# Crosstalk with renal proximal tubule cells drives acidosis-induced inflammatory response and dedifferentiation of fibroblasts via p38-singaling

**DOI:** 10.1186/s12964-024-01527-8

**Published:** 2024-02-24

**Authors:** Marie-Christin Schulz, Michael Kopf, Michael Gekle

**Affiliations:** Julius Bernstein Institute of Physiology, Magdeburger Straße 6, 06112 Halle (Saale), Germany

**Keywords:** Cellular crosstalk, Chronic kidney diseases, Extracellular acidosis, Inflammation, Dedifferentiation, MAPK-Signaling, JNK1/2, p38

## Abstract

**Background:**

Tubulointerstitial kidney disease associated microenvironmental dysregulation, like acidification, inflammation and fibrosis, affects tubule cells and fibroblasts. Micromilieu homeostasis influences intracellular signaling and intercellular crosstalk. Cell–cell communication in turn modulates the interstitial microenvironment. We assessed the impact of acidosis on inflammatory and fibrotic responses in proximal tubule cells and fibroblasts as a function of cellular crosstalk. Furthermore, cellular signaling pathways involved were identified.

**Methods:**

HK-2 (human proximal tubule) and CCD-1092Sk (human fibroblasts), in mono and coculture, were exposed to acidic or control media for 3 or 48 h. Protein expression of inflammation markers (TNF, TGF-ß and COX-2), dedifferentiation markers (N-cadherin, vinculin, ß-catenin and vimentin), fibrosis markers (collagen III and fibronectin) and phospho- as well as total MAPK levels were determined by western blot. Secreted collagen III and fibronectin were measured by ELISA. The impact of MAPK activation was assessed by pharmacological intervention. In addition, necrosis, apoptosis and epithelial permeability were determined.

**Results:**

Independent of culture conditions, acidosis caused a decrease of COX-2, vimentin and fibronectin expression in proximal tubule cells. Only in monoculture, ß-Catenin expression decreased and collagen III expression increased in tubule cells during acidosis. By contrast, in coculture collagen III protein expression of tubule cells was reduced. In fibroblasts acidosis led to an increase of TNF, COX-2, vimentin, vinculin, N-cadherin protein expression and a decrease of TGF-ß expression exclusively in coculture. In monoculture, expression of COX-2 and fibronectin was reduced. Collagen III expression of fibroblasts was reduced by acidosis independent of culture conditions.

In coculture, acidosis enhanced phosphorylation of ERK1/2, JNK1/2 and p38 transiently in proximal tubule cells. In fibroblasts, acidosis enhanced phosphorylation of p38 in a sustained and very strong manner. ERK1/2 and JNK1/2 were not affected in fibroblasts. Inhibition of JNK1/2 and p38 under coculture conditions reduced acidosis-induced changes in fibroblasts significantly.

**Conclusions:**

Our data show that the crosstalk between proximal tubule cells and fibroblasts is crucial for acidosis-induced dedifferentiation of fibroblasts into an inflammatory phenotype. This dedifferentiation is at least in part mediated by p38 and JNK1/2. Thus, cell–cell communication is essential for the pathophysiological impact of tubulointerstitial acidosis.

**Supplementary Information:**

The online version contains supplementary material available at 10.1186/s12964-024-01527-8.

## Introduction

**Fig. 1:**
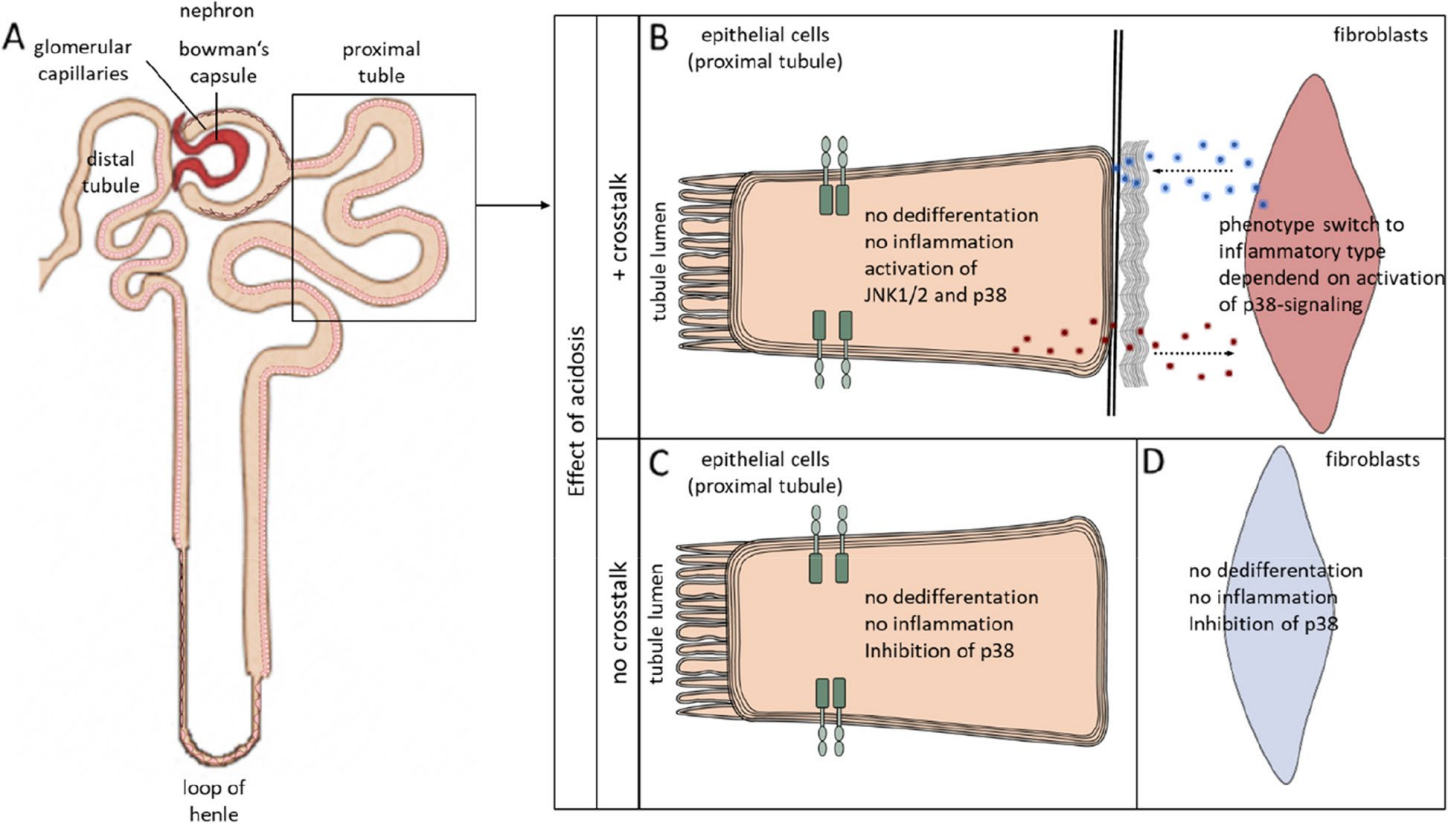
This sketch depicts a nephron (**A**), the smallest functional unit of the kidney. It consists of the bowman capsule and the tubule system. The tubule system can be divided in the proximal and distal tubule, the loope of Henle and the collecting duct. Panel B provides in an insight into the proximal tubule. The tubule epithelial cells are attached to the basement membrane, and behind it is the interstitial space, containing matrix proteins and fibroblasts. Panel **B** shows the effect of extracellular acidosis on proximal tubule cells and fibroblasts while enabling cellular crosstalk between these cells. Panels **C** and **D** show the impact of extracellular acidosis on proximal tubule cells (**C**) and fibroblasts (**D**) without cellular crosstalk

Approximately 10% of the world's population suffers from chronic kidney disease (CKD) from grade 3 and above [1]. Frequently, the development of CKD is accompanied by tubulointerstitial dyshomeostasis. The degree of functional decline closely correlates with the changes in the tubulointerstitial space. These changes comprise an increase of reactive oxygen and nitrogen species, hypoxia, acidosis, inflammation and fibrosis [2]. Inflammation and fibrosis are associated with non-respiratory acidification of the interstitial space [3–5]. Conversely, acidosis is described as a risk factor for an inflammatory response of tubulointerstitial cells that supports the formation of CKD, thereby forming a potential vicious cycle [2] (Fig. 1).

MAPK signaling mediates the cellular response to various extracellular stimuli, such as interstitial acidosis. ERK1/2, JNK1/2 and p38 are principal representatives of this signaling pathway. Earlier studies showed that extracellular acidosis causes a rapid, transient activation of MAPK [6, 7]. The rapid, transient activation mode mediates cellular responses like secretion of cytokines, thereby causing alterations in the micromilieu [8]. Moreover, MAPKs have a sustained activation mode for long-term response and chronic alterations, such as phenotype switches, as described for mesenchymal cells, including fibroblasts [9–11]. The activity of the MAPK pathway is strictly controlled by the orchestration of upstream regulators. On one hand upstream-kinases activate MAPKs, and on other hand various phosphatases like DUSPs (dual specificity phosphatase) lead to an inhibition of MAPKs [12, 13].

During pathophysiological processes, quiescent tissue fibroblasts can transdifferentiate into an inflammatory or fibrotic phenotype [14]. In the early phase of CKD, fibroblasts often show an inflammatory phenotype characterized by an increased secretion of inflammatory cytokines, such as TNF, IL-6, or COX metabolites, and a decreased secretion of matrix proteins. This shift in phenotype supports the development of a chronic interstitial inflammation [15]. In later stages of the disease, fibroblasts can switch to a fibrotic phenotype, contributing to the development of kidney failure.

Tubule and interstitial cells communicate via the secretion of soluble mediators, such as cytokines, prostaglandins or matrix proteins, thus contributing to physiological organ function [16, 17]. Micromilieu changes, such as acidosis, affect both tubule cells and fibroblasts thereby altering the tubulointerstitial crosstalk [18]. Subsequently, this may modify the response of fibroblasts to an extracellular stimulus, leading to inflammatory processes.

It must be emphasized that the renal pH landscape is complex and shows much larger spatiotemporal variations than the pH of other tissues. Thus, there is not “the” one physiological value and the usual threshold for transition into “the” pathological situation is most probably not applicable. Furthermore, the determination of this pH landscape is challenging and most of the data available were obtained in rodent kidneys. Nevertheless, pathological situations will lead to local acidification, and this drop in pH is supposed to be a pathogenic factor per se. The aim of our study was to investigate the impact of this pathogenic stimulus (drop in pH) on tubular cells and fibroblasts as a function of cellular crosstalk in a system with human cells. For this qualitative approach, a pH value out of the physiological range (pH 7.4) was compared with a value out of the pathophysiological range (pH 6.4). These values do not define the physiological and pathophysiological pH but are representatives of the two ranges. Interstitial pH around the late proximal tubule will be lower reaching values below 7.0 and the pathophysiological range starts below a threshold around pH 6.8. Due to its key role in micromilieu dyshomeostasis, the significance of tubulointerstitial crosstalk for acidosis-induced effects was a particular focus of the research. In this study we used human cell lines. In general, the use of immortalized cell lines goes along with limitations for the transferability to the in vivo situations. Yet, for the human situation with limited possibilities for controlled experimental interventions, appropriate cell culture allows the mechanistic investigations at the cellular level. HK-2 cells are derive from proximal tubule, they are characterized by a polarized morphology and expression of proximal tubule typical transporters such as sodium glucose symporter-2 (SGLT-2), and glucose transporter 1 (GLUT1) [19]. Moreover, it has been described that HK-2 cells respond to extracellular stimuli like drugs, or toxins and they are suitable to investigate the development of dedifferentiation [20, 21]. Based on these properties, HK-2 cells are an appropriate model to address our question.

CCD-1092Sk origin from skin. Nevertheless, based on studies of Korsunsky et al. and Büchler et al., which suggest that the microenvironment plays a more important role for fibroblast phenotype than their origin, CCD-1092Sk are suitable to extent our experimental setup to a coculture model [22, 23].

## Material and methods

If not stated otherwise, chemicals were purchased from Sigma-Aldrich, Munich, Germany.

### Cell culture

Normal human kidney epithelial cells (HK-2, CRL-2190™) and normal human fibroblasts (CCD-1092Sk, ATCC® CRL-2114™) were grown in DMEM medium supplemented with 10% fetal calf serum (FCS) and 2 g/l NaHCO_3_ at 37°C under a humidified 5% CO_2_ atmosphere and subcultivated once per week before confluence.

### Experimental setup

**Fig. 2:**
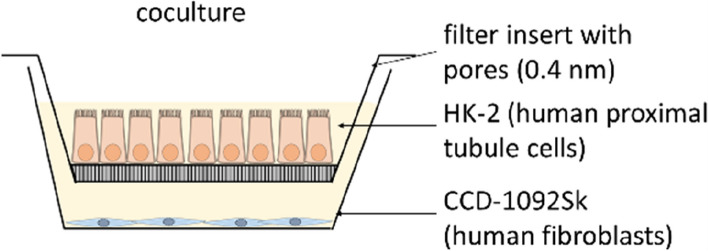
Scheme of indirect coculture

HK-2 cells were grown on permeable filter inserts (pore size 0.4 µm), and CCD-1092Sk cells were grown in 6-well plates. When cells were confluent on the filter inserts, containing HK-2 cells, they were transferred to the 6-well plates, containing CCD-1092Sk cells. This indirect coculture was used for the following experiments (Fig. 2). The coculture was transferred to medium without fetal calf serum.

(FCS) supplementation for 24 h and afterwards incubated with experimental pH conditions for 48 h. Control cells were exposed to media with a pH value of 7.4, while the acidosis group was incubated with media with a pH value of 6.4 or 6.0. Extracellular pH (pHe) was measured with a pH electrode InLab Solids Go-ISM (Mettler Toledo, Gießen, Germany). Only a minor reduction in pHe of the medium was observed during the chosen incubation periods (from 7.45 to 7.56 ± 0.02 for HK-2 and to 7.53 ± 0.02 for CCD-1092Sk). Therefore, the experiments could be performed under well-controlled conditions. In vivo epithelial cells attach to the substrate via their basolateral membrane, whereas the apical membrane faces towards the lumen. This substrate is the basement membrane, which separates the epithelial cell from the interstitial space that harbors fibroblasts. Thus, to properly mimic the in vivo situation, HK-2 cells were cultured in the inserts, attaching to the filter membrane, and the fibroblasts were cultivated on the bottom of the wells. 2.3. Assessment of cytosolic pH.

Cytosolic pH of HK-2 and CCD-1092Sk cells was determined by using the pH sensitive dye BCECF (2',7'-bis-(2-carboxyethyl)-5-(and-6)-carboxyfluorescein, acetoxymethyl ester, Invitrogen, Paisley, UK) as described before [24]. Cells were exposed for 15 min with 5 μmol/l BCECF-AM, diluted in media, in the incubator. Afterwards, cells were washed 2 × with corresponding Ringer solution, and transferred to the stage of an Axiovert 100 TV microscope (Zeiss, Oberkochen, Germany). The perfusate was controlled at 37 °C for each experiment. Intracellular fluorescence was determined in individual cells. Excitation wavelengths were 460 and 488 nm, and the emitted light was filtered through a band-pass filter (515–565 nm). Images were digitized by using video-imaging software (VisiView® Software, Visitron Systems, Puchheim, GER). Ratio images were sampled every 10 s. Calibration was performed after each experiment by the nigericin technique. Supplementary Fig. 5 shows a typical curve profile of a BCECF measurement and a calibrating curve.

### Western blot

Cells were lysed in 50 µl of ice-cold MOPS Triton buffer (see supplementary table for buffer composition). Subsequently, cells were centrifuged at 14,000 g for 10 min (4°C) and filled up with 16.6% of the total volume of 6 × Laemmli buffer (see supplementary table for buffer composition). Before loading the gel, samples were heated to 95°C for 5–10 min. For protein separation a 10% polyacrylamide gel was used. Proteins were separated by sodium dodecyl sulfate–polyacrylamide gel electrophoresis (SDS-PAGE) and transferred onto a nitrocellulose membrane. All proteins on the nitrocellulose membrane were stained with Ponceau S solution (AppliChem GmbH, Darmstadt, Germany). A picture of the stained membrane was taken with the Bio-Rad ChemiDoc™ XRS gel documentation system (Bio-Rad Laboratories GmbH, Feldkirchen, Germany). After staining, the membrane was blocked with 5% nonfat dry milk powder diluted in TRIS-buffered saline (TBS) (see supplementary table for buffer composition) and incubated with primary antibody (Table 1) diluted in 5% bovine serum (BSA) diluted in TBS Tween20 overnight. After removing the primary antibody and washing the membrane, a secondary antibody coupled to horseradish peroxidase was diluted 1:1000 in 5% nonfat dry milk powder in TBS Tween20 and added to the membrane. After removal of the secondary antibody solution, three wash steps in TBS Tween20 were performed. Finally, the membrane was incubated for 5 min with Clarity™ Western ECL Substrate (Bio-Rad, Munich, Germany), and the peroxidase activity-based light emission was recorded by an imaging system (Image Quant LAS4000, GE Healthcare, Buckinghamshire, GB or Bio-Rad ChemiDoc™ XRS gel documentation system). The density of the total protein, made visible with Ponceau S solution, and protein target bands were quantified using Image Lab Software 6.1 (Bio-Rad, Munich, Germany, 2020). The normalization was performed with the Ponceau S-stained total protein. When proteins have similar sizes, the membranes were incubated for 15 min at 65°C with Restore™ Western Blot Stripping Buffer (Thermo Fisher Scientific GmbH, Dreieich, Germany) to remove the antibodies. Afterward, the membranes were reused for detection.

**Table 1:** Collagen and fibronectin concentration under control conditions, mean ± s.e.m

	collagen III [ng/µg]	fibronectin [ng/µg]
HK-2 mono	9 ± 3	2748 ± 684
CCD-1092SK mono	2477 ± 730	5193 ± 1263
total co	3709 ± 1249	2011 ± 326

### Collagen direct ELISA

HK-2 and CCD-1092Sk cells in monoculture were seeded in 24-well-plates or respectively in coculture as described in 2.2. The cells were incubated with media enriched with 50 mg/l ascorbic acid to support collagen synthesis and 50 mg/l ß-aminoproprionitrile to avoid collagen polymerization, which improves collagen determination. Collagen and fibronectin concentration was measured in the media according to and normalized to cellular protein content [25]. Antibodies against collagen III (1:1000) and fibronectin 1 (1:2000) were obtained from Biomol. HRP-coupled secondary antibodies (1:5000) were obtained from Cell Signaling (see Table 1).

### Data analysis

All data are given as mean ± SEM. Statistical significance was determined by unpaired Student’s t-test or rank sum, as appropriate. Differences were considered statistically significant when *p* < 0.05.3

## Results

### Determination of maximum non-damaging acidic pH values

To determine the pH range that does not result in non-specific cell damage (maximum non-damaging acidotic pH values), HK-2 and CCD-1092Sk cells were exposed to various pH values (7.4; 7.0; 6.8; 6.6; 6.4; 6.2) over a period of 48 h. In supplementary figure S1 a, b, g, h the impact of acidic media on cell protein per petri dish (biomarker for cell loss) is shown. Supplementary figure S1 c, d, i, j shows the acidosis-effect on caspase-3-activity (biomarker for apoptosis) and S1 e, f, k shows the effect on LDH-release; biomarker for necrosis, i.e. membrane leakage. HK-2 and CCD-1092Sk cells showed signs of necrosis only at pH values lower than 6.4, in monoculture as well as in coculture.

### Extracellular acidosis had no impact on epithelial barrier function in coculture

Beside cell death, a reduced epithelial barrier function can be a sign for epithelial cell damage. Supplementary figure S1l shows a slight decrease of the epithelial barrier function in monoculture whilst S1m shows that the barrier function of HK-2 cells was not affected by exposure to acidic media (pH 6.4) in coculture.

### Extracellular acidosis reduced cytosolic pH values

Under control conditions (pHe = 7.4) intracellular pH in HK-2 cells was 7.53 ± 0.02 (*n* = 24) and under acidic conditions it was 6.66 ± 0.02 (*n* = 24) (pHe = 6.4). For CCD-1092Sk cells, the respective values were 7.48 ± 0.03 (*n* = 33) and 6.92 ± 0.04 (*n* = 33).

### Acidic media caused no inflammatory response in HK-2 cells

**Fig. 3:**
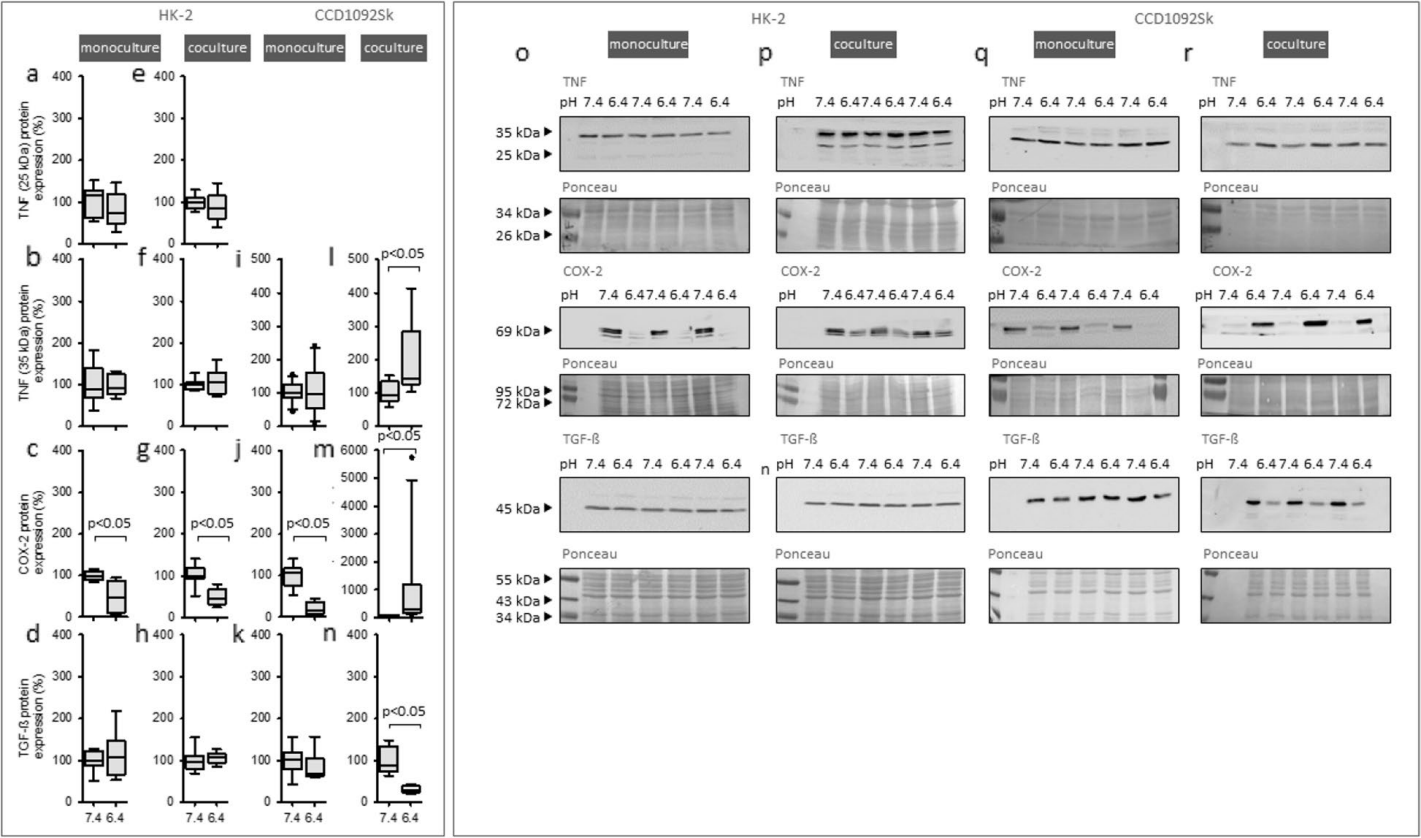
Effect of acidic media on inflammation markers in HK-2 and CCD-1092Sk cells in mono-and coculture. Protein expression changes of TNF, COX-2 and TGF-ß in HK-2 in mono (**a**-**d**), coculture (**e**–**h**) and CCD-1092Sk in mono (**i**-**k**) and coculture (**l**-**n**). Representative western blots of proteins isolated from cells exposed to acidosis (**o**-**r**), *n* = 6–9. Exposure time = 48 h

To assess whether the cells exhibit an inflammatory response, protein expression of the cytokines TNF (inflammatory) and TGF-ß (antiinflammatory), as well as the enzyme COX-2 (proinflammatory) was determined. Figure 3 a, b, d, e, f, h shows that acidosis had no impact on the expression of both cytokines in mono-and coculture, whereas COX-2 protein expression decreased in mono-and coculture (Fig. 3 c, g).

### In coculture, acidic media caused an inflammatory response in CCD-1092Sk cells

Figure 3i, k shows that the acidic milieu had no impact on the expression of TNF and TGF-ß in fibroblasts in monoculture, but led to a strong decrease of COX-2 protein expression (Fig. 3 j). In contrast, under coculture conditions, the acidic medium caused an increase of the TNF and COX-2 expression and a decrease of TGF-ß expression (Fig. 3l-n).

### Acidic media did not cause dedifferentiation of HK-2 cells

**Fig. 4:**
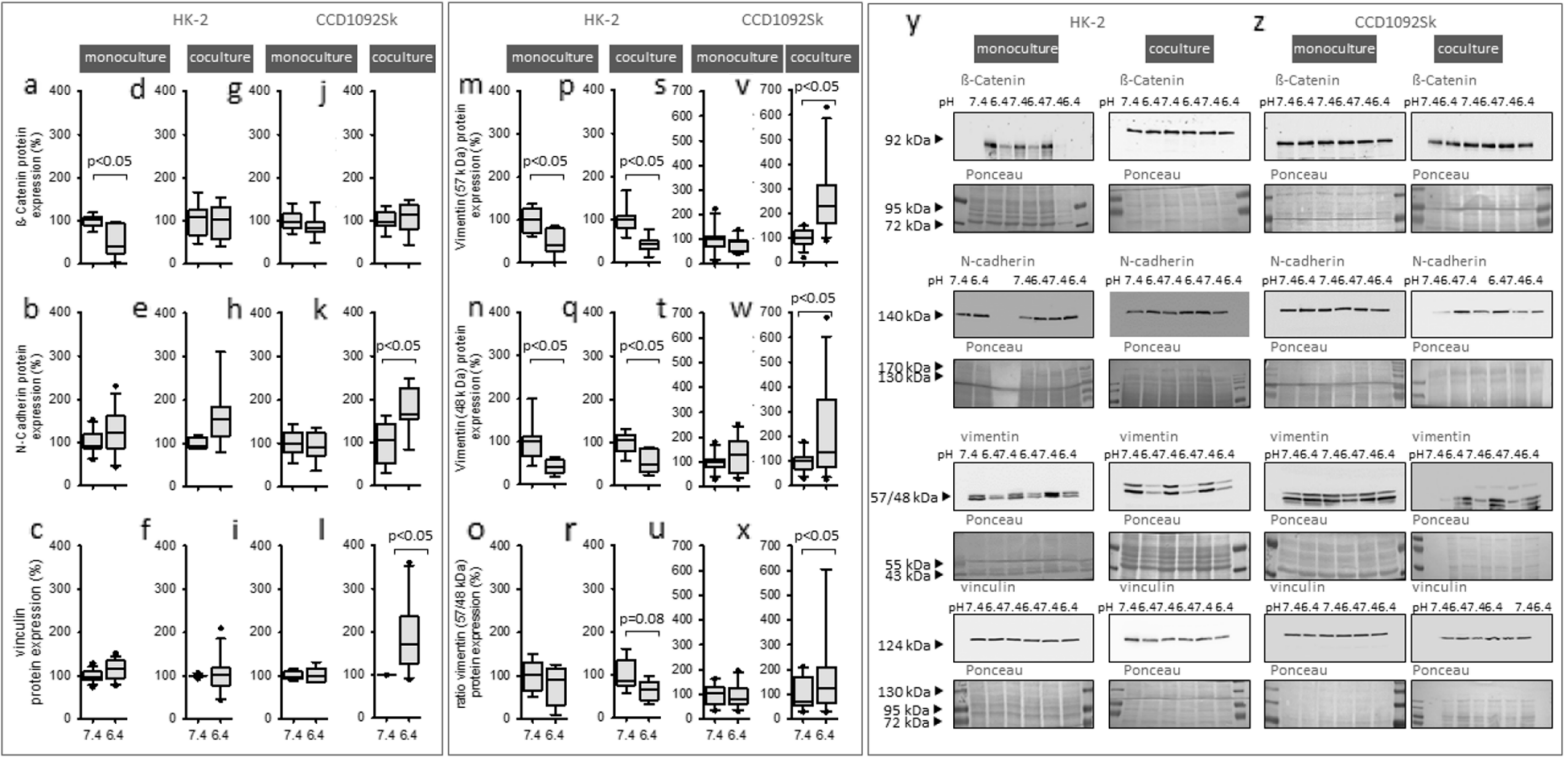
Effect of acidic media on differentiation markers in HK-2 and CCD-1092Sk cells in mono-and coculture. Protein expression changes of ß-catenin, N-cadherin, vinculin and vimentin in HK-2 in mono (**a**-**b**, **m**–**o**) coculture (**d**-**f**, **p**-**r**) and CCD-1092Sk in mono (**g**-**i**, **s**-**u**) and coculture (**j**-**l**, **v**-**x**). Representative western blots of proteins isolated from cells exposed to acidosis (**y**–**z**). n = 8—18. Exposure time = 48 h

As indicator of dedifferentiation, expression of cell contact proteins (ß-catenin, vinculin and N-cadherin) were determined. Additionally, expression of the mesenchymal cytoskeletal protein vimentin was assessed. In HK-2 cells in monoculture, acidosis led to a decreased expression of ß-catenin and full-length vimentin as well as the smaller 48 kDa vimentin-fragment (Fig. 4 a, m–o). Under coculture conditions, only vimentin expression was affected (Fig. 4 p-r). Figure 4r shows that the ratio of full-length vimentin (57 kDa) and the lower fragment (48 kDa) decreased in the acidosis group.

### Acidic media caused dedifferentiation of CCD-1092Sk cells

Figure 4 g-i, g, s-u shows that the exposure to acidic media had no impact on markers for dedifferentiation in fibroblasts in monoculture. In coculture, however acidosis induced an increased expression of N-cadherin, vinculin and vimentin in fibroblasts (Fig. 4 j-l, v-x).

### Impact of acidic media on fibrosis marker in HK-2 and CCD-1092Sk cells

**Fig. 5:**
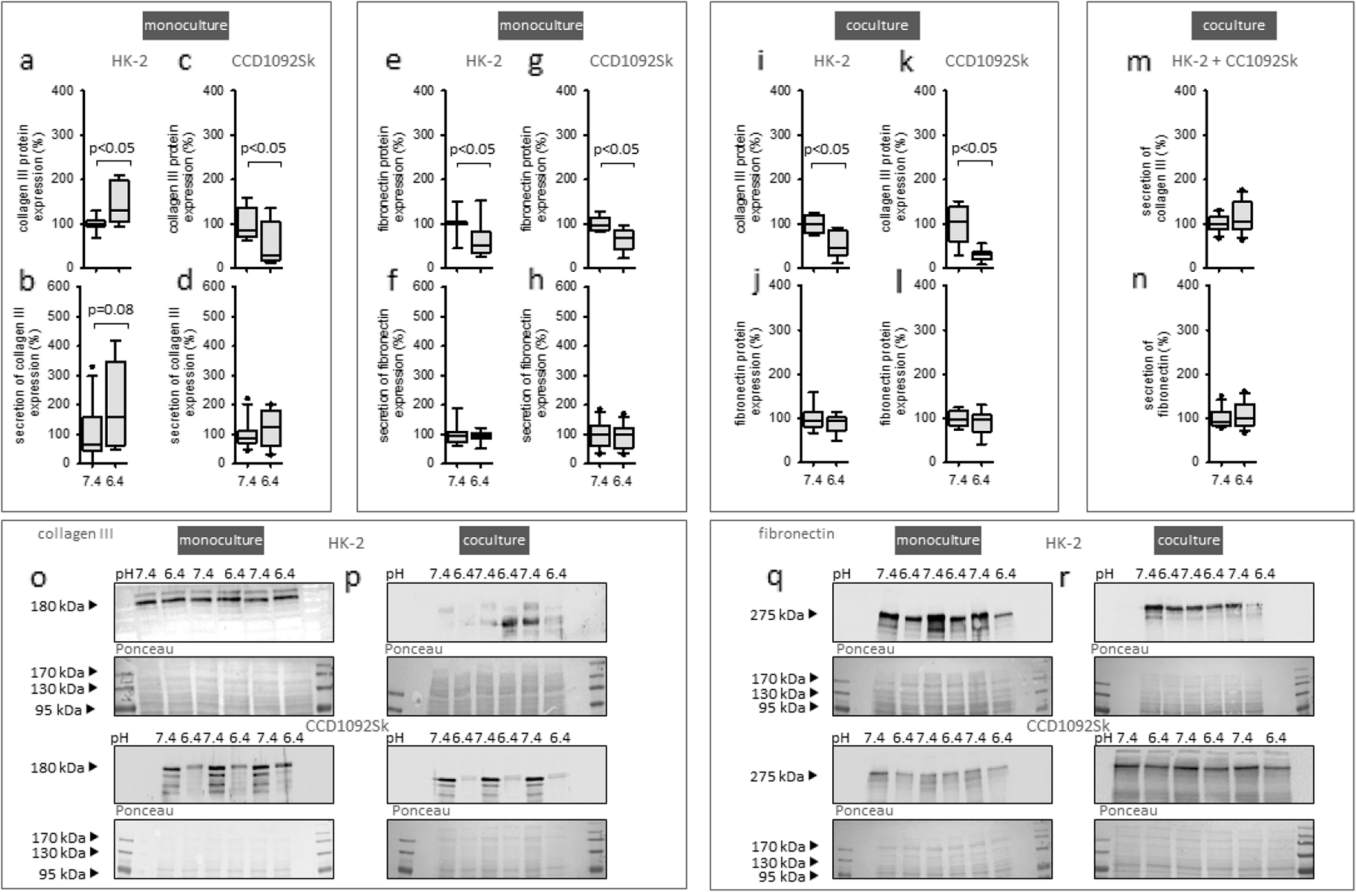
Effect of acidic media 48h on fibrosis markers in HK-2 and CCD1092Sk cells in mono-and coculture. Protein expression changes of intracellular and secreted collagen III and fibronectin in HK-2 in mono (**a**-**b**, **e**–**f**), coculture (**c**-**d**, **g**-**h**, **m**–**n**) and CCD-1092Sk in mono (**c**-**d**, **g**-**h**, **k**-**n**) and coculture (**l**-**n**). Representative western blots of proteins isolated from cells exposed to acidosis (**o**-**r**). *n* = 7—12. Exposure time = 48 h

Table 1 shows the concentration of secreted collagen III and fibronectin under control conditions. In HK-2 cells in monoculture exposure to acidic media led to an increase of secreted and intracellular collagen III (Fig. 5 a, b). Moreover, the secretion of fibronectin was unaltered, whereas the intracellular protein expression decreased (Fig. 5 e, f).

Acidosis had no impact on the secretion of collagen III and fibronectin in fibroblasts in monoculture, but the expression of intracellular collagen III and fibronectin was decreased (Fig. 5 c, d, g, h).

Because secreted proteins can pass the filter pores, it is not possible to assign protein changes in the media to one cell type. However, we measured the total amount of secreted collagen III and fibronectin. Figure 5 m, n shows that acidosis had no impact on the secreted collagen III and fibronectin in coculture. The same was shown for intracellular fibronectin in both cell types, whereas the expression of intracellular collagen III was decreased in both cell types in coculture (Fig. 5 i-l).

### Exposure to acidic media for 48 h causes caused sustained changes of MAPK-phosphorylation in HK-2 cells

**Fig. 6:**
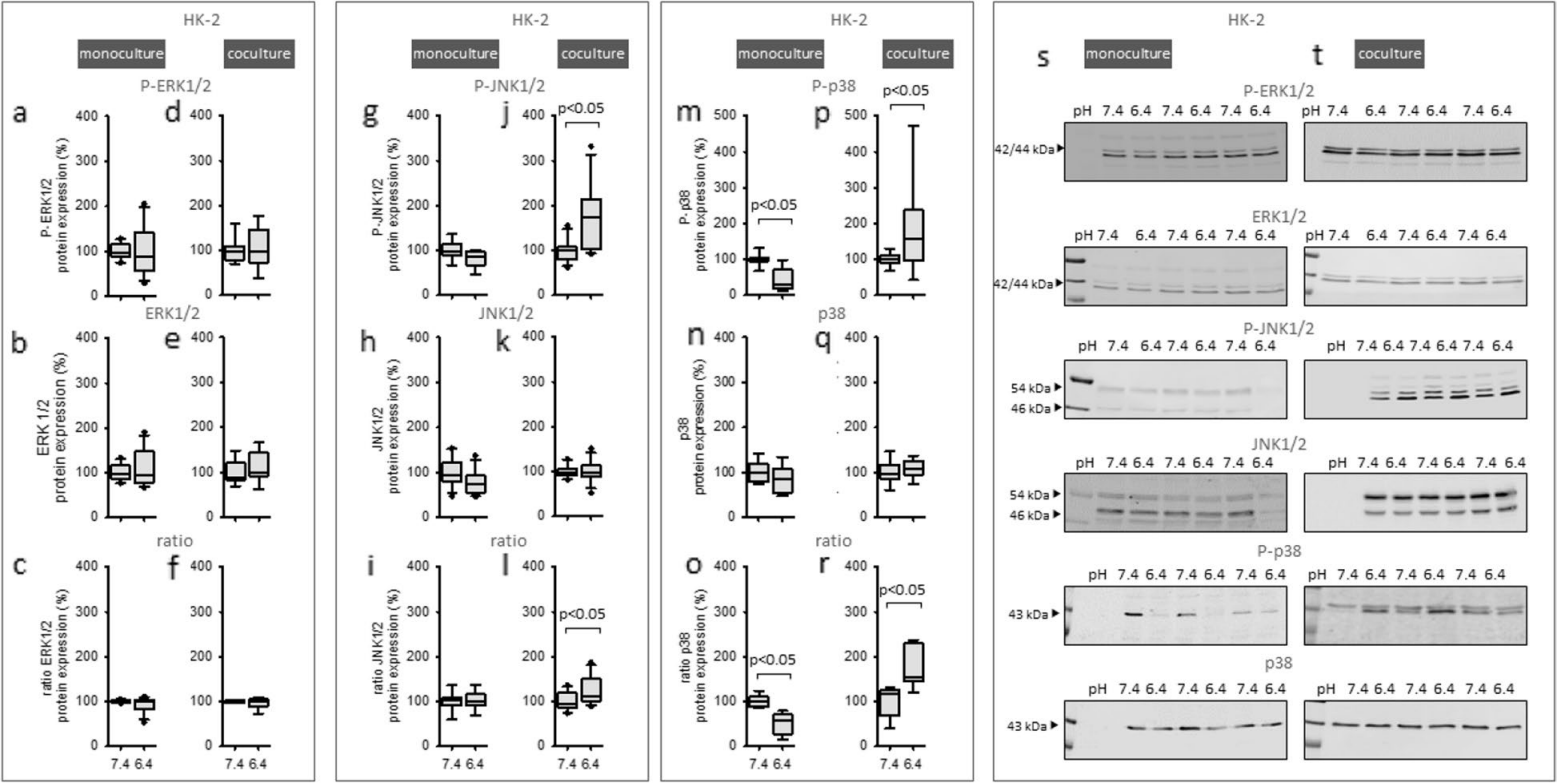
Long-time effect of acidic media on MAPKs in HK-2 cells in mono- and coculture. Protein expression changes of phosphorylated and total ERK1/2, JNK1/2 and p38 in monoculture (**a**-**c**, **g**-**i**, **m**–**o**) and coculture (**d**-**f**, **j**-**l**, **p**-**r**). Representative western blots of proteins isolated from cells exposed to acidosis (**s**-**t**). *n* = 6—9. Exposure time = 48 h

MAPK can mediate long-term effects through by a sustained activation mode. We assessed longterm effects. This was measured after an incubation time of 48 h, with acidic media. Figures 6 j-l, p-r shows that in HK-2 cells in coculture, the phosphorylation of the MAPK JNK1/2 and p38 was increased. In contrast, under monoculture conditions acidic media had no impact on JNK1/2 (Fig. 6 g- i) and led to a decreased phosphorylation of p38 (Fig. 6 m–o). Moreover, total as well as phosphorylated ERK1/2 was not changed under any of these conditions.

(Fig. 6 a-f). These results show that acidosis enhanced the sustained activation of JNK1/2 and p38 in HK-2 cells, only when a cellular crosstalk with fibroblasts is possible.

### Exposure to acidic media for 48 h caused changes of sustained phosphorylation and protein expression of MAPK in CCD-1092Sk cells

**Fig. 7:**
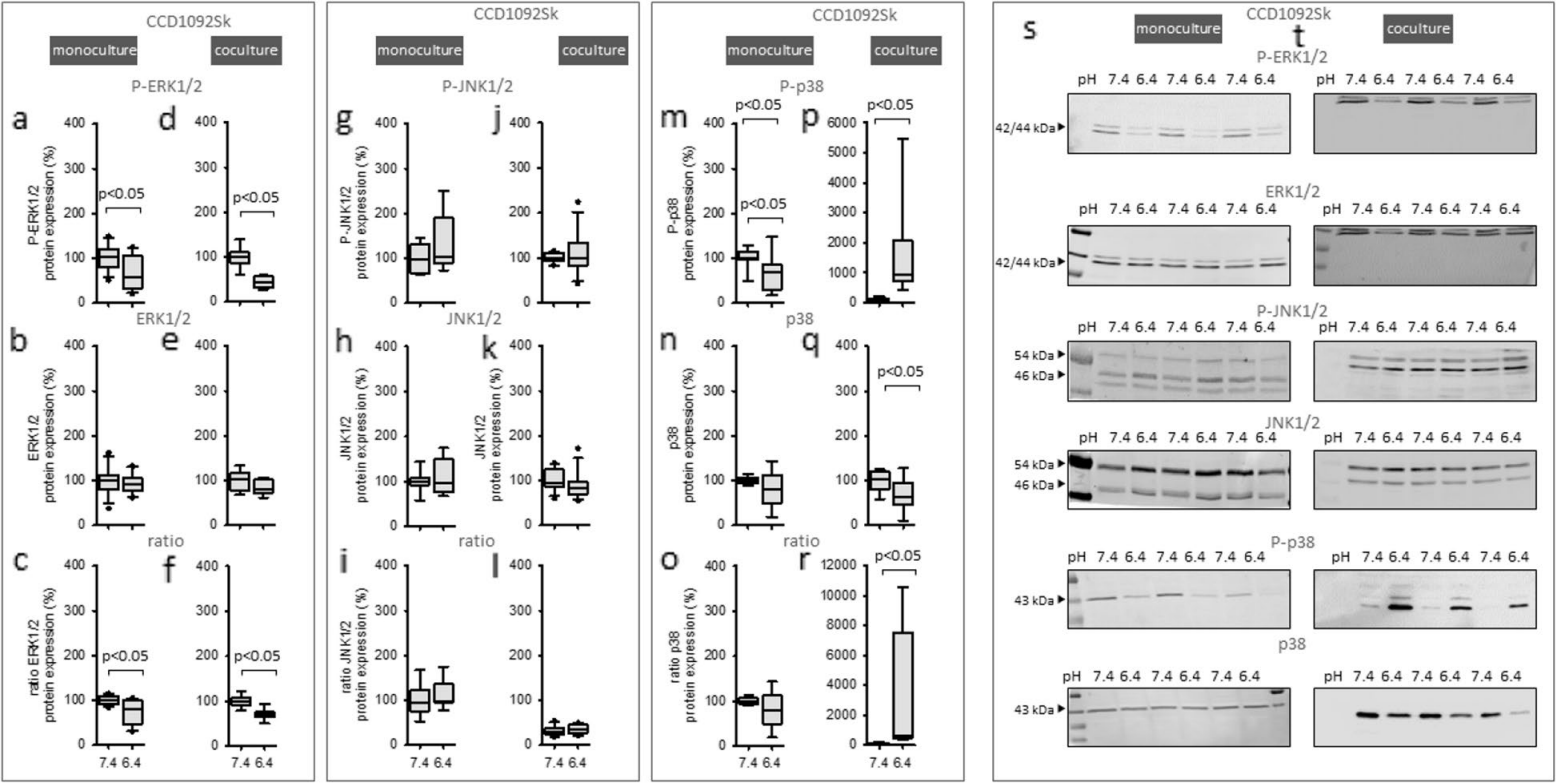
Long-time effect of acidic media on MAPKs in CCD-1092Sk cells in mono- and coculture. Protein expression changes of phosphorylated and total ERK1/2, JNK1/2 and p38 in monoculture (**a**-**c**, **g**-**i**, **m**–**o**) and coculture (**d**-**f**, **j**-**l**, **p**-**r**). Representative western blots of proteins isolated from cells exposed to acidosis (**s**-**t**). *n* = 6—9. Exposure time = 48 h

Figure 7 a-c, m–o depicts that exposure to acidic media resulted in a decreased phosphorylation of ERK1/2 and p38 in CCD1092Sk under monoculture conditions. In coculture, the acidosis-induced decrease of P-ERK1/2 (Fig. 7 d-f) remains, whereas the phosphorylation of p38 was highly upregulated (Fig. 7p-r). Phosphorylation and expression of JNK1/2 were unaltered (Fig. 7g-l). These data show that acidosis led to an eminent increase of p38 phosphorylation, only when cellular crosstalk occurs, i.e. there is a synergism of cellular crosstalk and acidosis.

### Short-time effect of acidic media on MAPK expression and phosphorylation

**Fig. 8:**
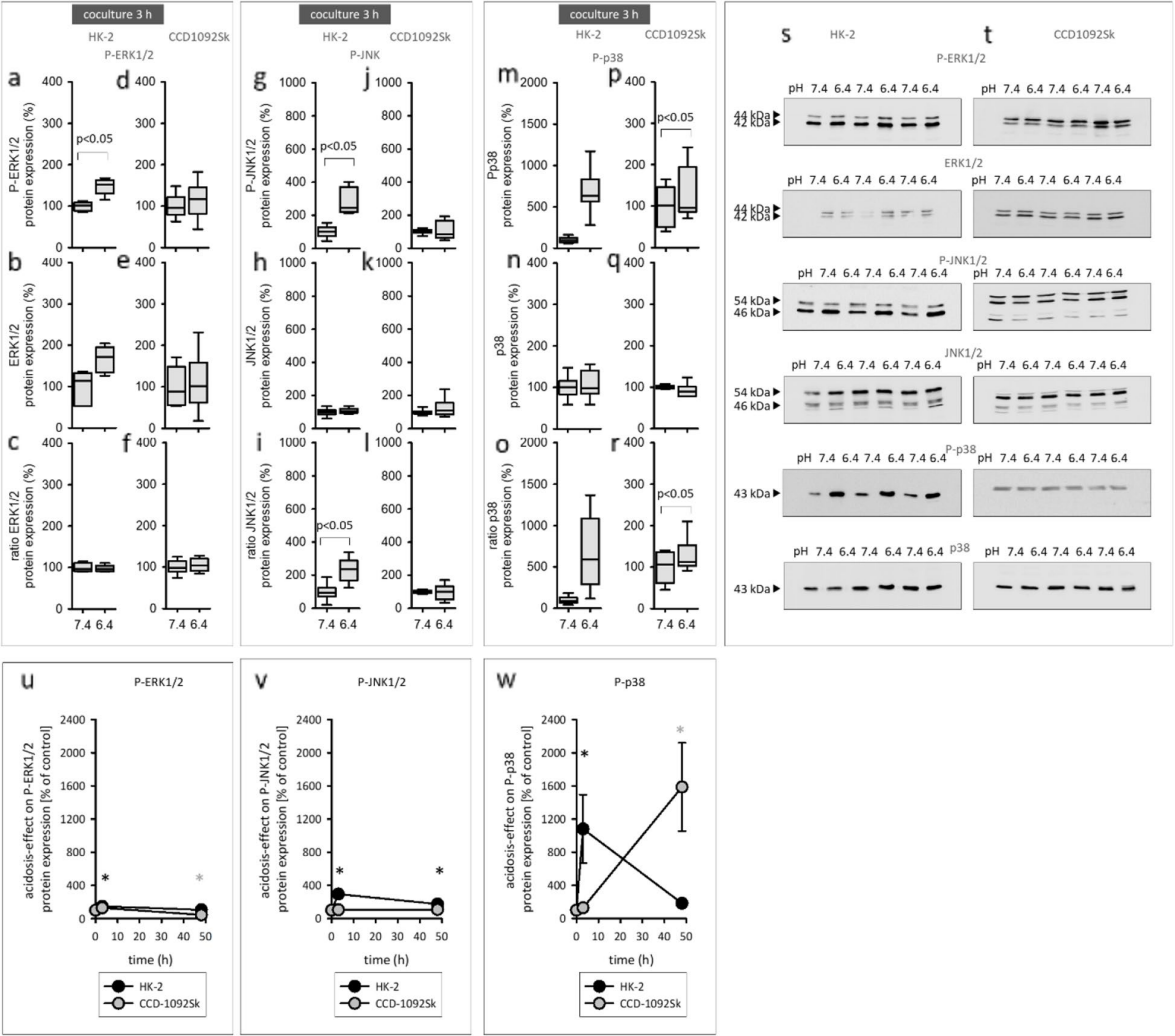
Short-time effect of acidic media on MAPK in HK-2 and CCD1092Sk cells in coculture. Protein expression changes of phosphorylated and total ERK1/2, JNK1/2 and p38 in HK-2 (**a**-**c**, **g**-**i**, **m**–**o**) and CCD-1092Sk (**d**-**f**, **j**-**l**, **p**-**r**). Representative western blots of proteins isolated from cells exposed to acidosis (**s**-**t**). Comparison of acidosis-effect in HK-2 and CCD-1092Sk on ERK1/2 (6**u**), JNK1/2 (6**v**) and p38 (6**w**). *n* = 6—9. exposure time = 3 h

The former data indicate that acidosis causes a long-lasting activation of MAPK only in coculture. Subsequently, it was tested if acidosis also causes a rapid activation of MAPK in coculture. Incubation of the cells in coculture for 3 h with acidic media led to an increased protein expression of phosphorylated ERK1/2, JNK1/2 and p38 in HK-2, whereas in CCD-1092Sk no changes were observed (Fig. 8). Figure 8 u-w shows the time course of MAPK-activation in HK-2 and CCD-1092Sk. This figure illustrates that acidosis induced by far the strongest activation of p38 in both cell lines. Moreover, Fig. 8w clearly shows that acidosis induced a rapid and transient activation of p38 in HK-2 cells, whereas in CCD-1092Sk the p38 activation was long-lasting.

### Acidosis-induced changes in CCD-1092Sk depend on p38 and JNK1/2 activity

**Fig. 9:**
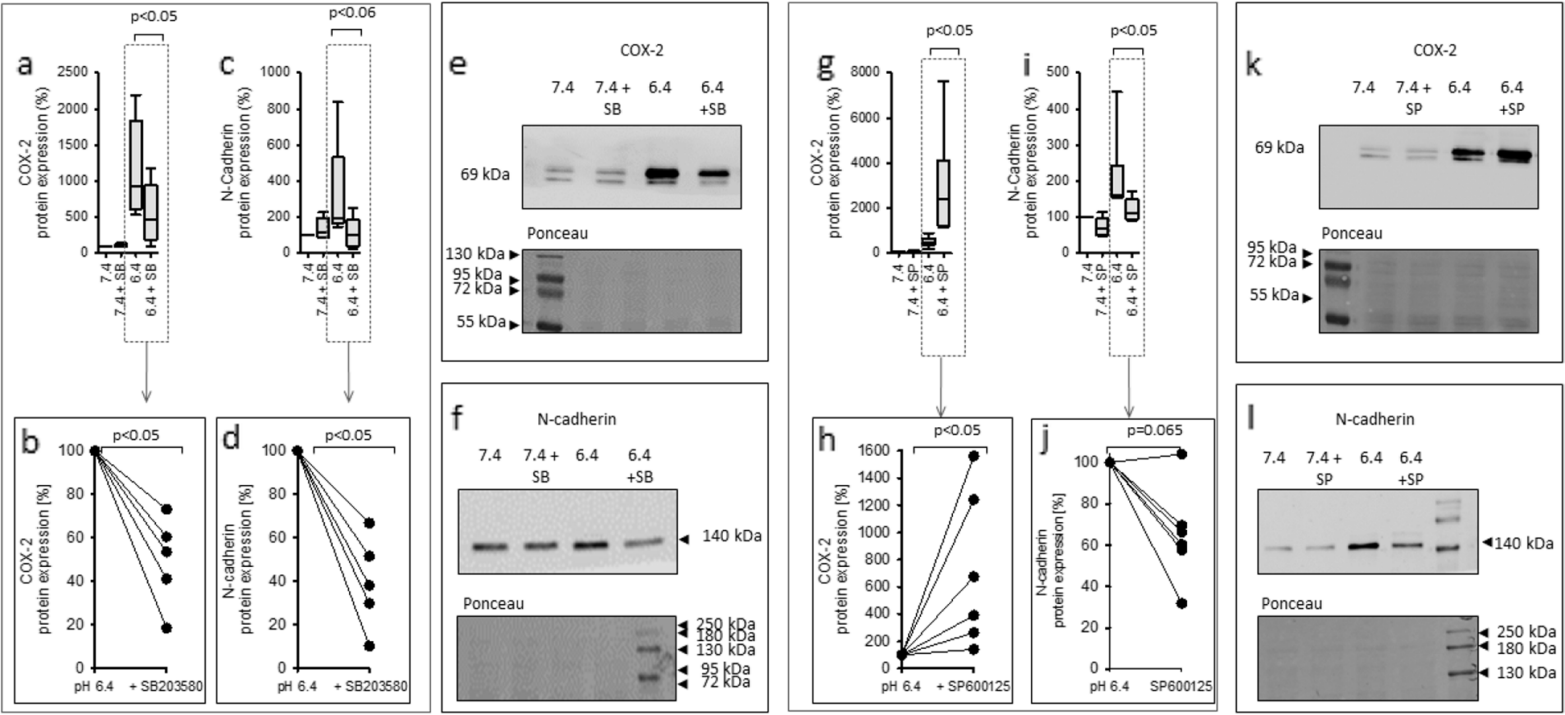
Impact of p38-inhibition (10 µM SB203580) and JNK1/2-inhibition (10 µM SP600125) on acidosis-induced changes in CCD-1092Sk cells coculture. Impact of p38-inhibition on acidosis-induced increase of COX-2 (**a**-**b**) and N-cadherin (**c**-**d**) and JNK1/2 inhibition on acidosis-induced increase of COX-2 (**g**-**h**) and N-cadherin (**i**-**j**). Representative western blots of proteins isolated from cells exposed to acidosis + 10 µM SB203580 or 10 µM SP600125 (**e**–**f**, **k**-**l**). *n* = 6. exposure time = 48 h

The effect of acidosis on MAPK is most pronounced for p38 phosphorylation, a pathway known to mediate inflammatory responses in cells. The data also show an activation of JNK1/2, which is also known to mediate inflammatory responses. Consequently, the involvement of p38 and JNK1/2 activity in the development of the inflammatory phenotype in CCD-1092Sk was investigated. For this purpose, the cells were exposed to acidic media for 48 h in coculture in the presence and absence of specific inhibitor of p38 (10 µM SB203580) or JNK1/2 (10 µM SP600125). Western blot analysis was performed on CCD-1092Sk to measure the protein expression change of the inflammatory marker COX-2 and the dedifferentiation marker N-cadherin. Figure 9 shows that the inhibitors itself had a negligible effect on the protein expression of N-cadherin and COX-2. Moreover, Fig. 9 shows that the acidosis-induced increase of N-cadherin was reduced after inhibiting p38 (Fig. 9c, d) and JNK1/2 (Fig. 9 i, j). Furthermore, the acidosis-induced increase of COX-2 was diminished when p38 (Fig. 9a, b) was inhibited but surprisingly enhanced after inhibiting JNK1/2 (Fig. 9g, h).

### Acidosis-effect on upstream regulators of p38

**Fig. 10:**
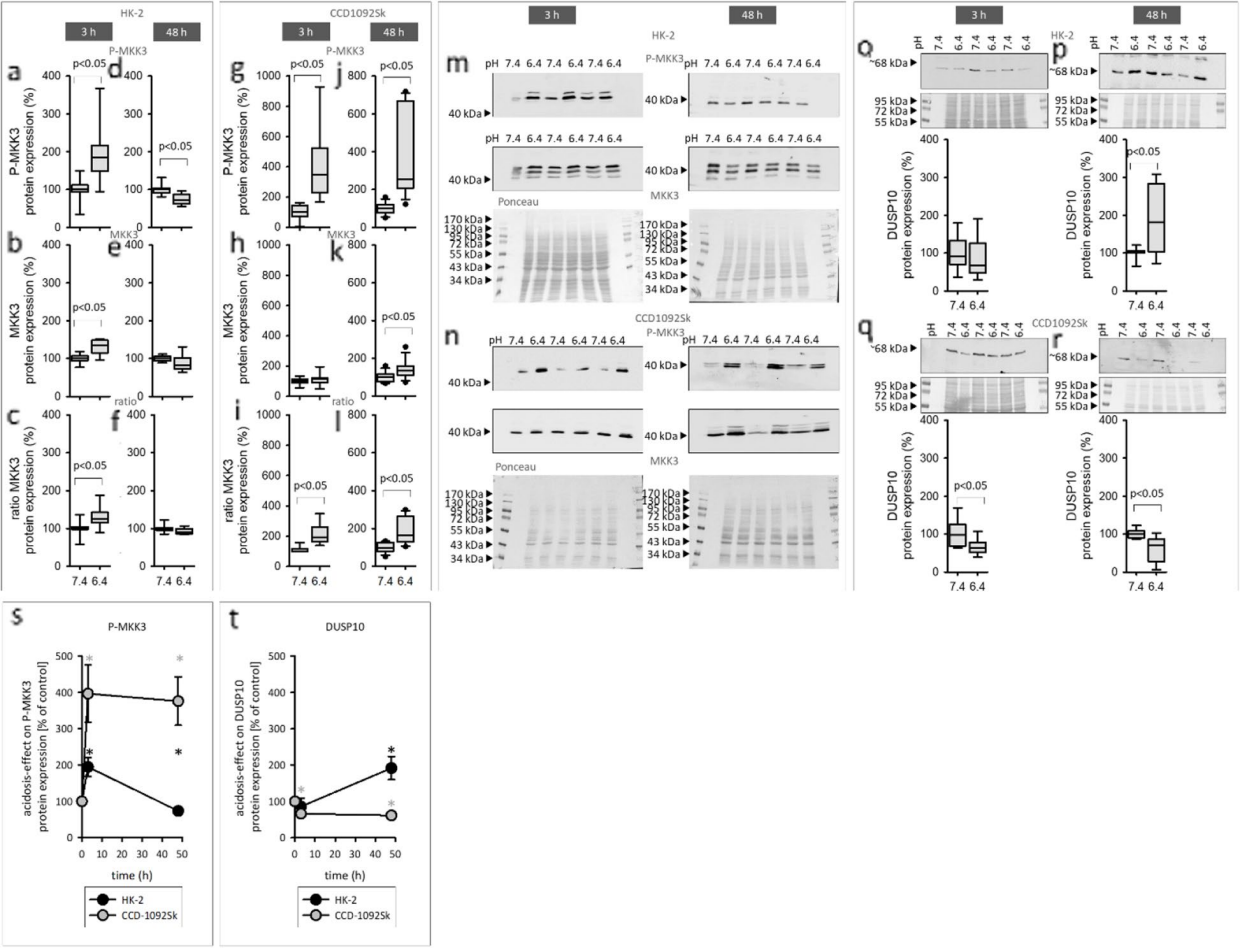
Effect of acidic media on the expression of MKK3 and DUSP10 in HK-2 and CCD-1092Sk cells in coculture. Impact of acidic media on the expression of MKK3 in HK-2 cells after 3 h (**a**-**c**) and after 48 h (**d**-**f**). Impact of acidic media on the expression of MKK3 in CCD-1092Sk cells after 3 h (**g**-**i**) and after 48 h (**j**-**l**). Effect of acidic media on DUSP10 expression HK-2 and CCD-1092Sk cells after 3 h (**o**, **q**) and after 48 h (**p**, **r**). Comparison of the acidosis-effect in HK-2 and CCD-1092Sk cells on MKK3 (8**s**) and DUSP10 (8**t**) Representative western blots of proteins isolated from cells exposed to acidosis (**m**, **n**) *n* = 6–9. exposure time = 3h or 48 h

Because, acidosis induced the strongest activation of p38 compared to ERK1/2 and JNK1/2 in both cell lines; its upstream-regulators were investigated. MKK3/6 are upstream kinases of p38, which induce the p38 -activity. Additionally, numerous phosphatases, that modulate p38 activity by dephosphorylation, existing. DUSP10 is a phosphatase with high affinity for p38. To determine which processes, lead to the observed increased p38 activity, the acidosis effect on MKK3 as upstream activator and DUSP10 as upstream inhibitor was measured. Figure 10 a-f shows that the phosphorylation of MKK3 was increased in HK-2 cells after 3 h incubation with acidic media; this change was not measured after 48 h. Moreover, the DUSP10 expression was increased after 48h in HK-2 cells, but unchanged after 3h (o-p). For CCD-1092Sk cells, the phosphorylation of MKK3 was increased after 3h as well as after 48h exposure to acidic media (Fig. 10 g-l). Besides that, the expression of DUSP10 was decreased after 3h and 48h in CCD-1092Sk (Fig. 10 q, r). Figure 10 s, t shows the time course of P-MKK3 and DUSP10 expression and compare the effect in HK-2 and CCD-1092Sk cells.

## Discussion

The present study demonstrates a synergistic interaction of epithelial-fibroblast crosstalk and extracellular acidosis, leading to an inflammatory phenotype in human fibroblasts (Fig. 1). This extends our knowledge concerning the role of cellular communication for acidosis-induced alterations.

Aim of the present study was to test the hypothesis that the impact of acidosis is essentially a function of renal cellular crosstalk also in human cells. To the best of our knowledge, the present study is the first to test the effect of extracellular acidosis in a human renal coculture model.

### Fibroblasts

Extracellular acidosis in monoculture did not elicit inflammatory or fibrotic alterations nor dedifferentiation in fibroblasts. By contrast, acidosis in combination with tubule cell-fibroblast crosstalk leads to an activation of fibroblasts, and dedifferentiation to inflammatory phenotype, with enhanced COX-2 and TNF expression and decreased TGF-ß expression. In addition, the increased expression of vinculin, N-cadherin, and both vimentin fragments indicates dedifferentiation to an inflammatory type of fibroblasts. However, there was no evidence for a synergistic action of coculture and acidosis concerning the fibrotic response.

We conclude that tubule cell-fibroblast crosstalk is necessary to generate a micromilieu changes, leading to the transition of fibroblasts to an inflammatory phenotype, which is prevalent during early stages of CKD [14].

### Proximal tubule cells

In HK-2 cells acidosis does not induce an inflammatory response or dedifferentiation, regardless of the culture conditions. The effects on matrix proteins under coculture conditions indicate a certain protective effect of the cellular crosstalk concerning fibrosis, which has also been demonstrated for rat proximal tubule cells [26].

In summary, proximal tubule derived HK-2 cells do not respond to acidosis with canonical pathophysiological changes such as inflammation, fibrosis, or dedifferentiation. Proximal tubule cells seem to play more the role of a mediator that triggers fibroblasts in a micromilieu dependent manner.

### Signaling pathways

Earlier studies revealed the MAPK signaling as pH sensitive, for example, in tumor cells, making the MAPK pathway a promising candidate to mediate the observed alterations [7, 27]. Our data show that acidosis induced an inhibition of the p38-signaling in both cells in monoculture. In contrast, in coculture, acidic media caused an eminent activation of p38 in both cell lines and JNK1/2 in HK-2. Additionally, acidosis led to a rapid increase in the expression of phosphorylated ERK1/2, JNK1/2 and p38 in HK-2 cells. Figure 8u-w compares the effect of acidosis in HK-2 and CCD-1092Sk as time course and shows that HK-2 cells react with a rapid and transient activation of MAPK, whereas CCD-1092Sk cells exhibit a long-lasting effect. It is conceivable that the activated MAPKs influence the secretome of HK-2 cells and thereby contribute to a modified microenvironment, which affects the pH sensitivity of fibroblasts. This hypothesis has to be tested in future experiments.

In our results, it is evident that acidosis, only in conjunction with cellular crosstalk, induces an inflammatory phenotype in fibroblasts. Moreover, Fig. 8 u-w illustrates that acidosis induced by far the strongest activation of p38 in both cell lines in coculture. Consequently, it was examined whether the p38 signaling pathway is involved in mediating the acidosis-induced phenotype switch in fibroblasts.

For this purpose, the expression of COX-2 (marker for inflammation) and N-cadherin (marker for dedifferentiation) was measured in fibroblasts under coculture conditions after exposure to acidic media with addition of a p38-inhibitor (10 µM SB203580).

The acidosis-induced increase of COX-2 and N-cadherin was diminished in fibroblasts after inhibition of p38 (10 µM SB203580). These results indicate that the acidosis-induced phenotype switch of fibroblasts is mediated by p38-signaling. To examine the p38-signaling in more detail, the expression of upstream regulators was measured. The upstream kinase MKK3 phosphorylates p38, leading to increased p38 activity [13]. Furthermore, the dual specific phosphatase DUSP10 dephosphorylates MAPKs with high affinity for p38, thus serves as a strong inhibitor of the p38 signaling pathway [12]. In fibroblasts, acidosis induced an increased expression of phosphorylated MKK3 and a decreased expression of DUSP10 after 3 h. This effect is stable after 48h, and this orchestration of upstream activators and inhibitors, leads to a long lasting activation of p38, but not to rapid response after 3h. In contrast, the expression of phosphorylated MKK3 increased after 3h in HK-2 cells, potentially causing the rapid activation of p38 in HK-2 cells, while the expression of DUSP10 was not changed. However, after 48h, the expression of phosphorylated MKK3 was decreased, while DUSP10 was increased. Since our results show that the acidosis-effect on expression of phosphorylated p38 was strongly attenuated in HK-2 cells after 48 h, these results indicate that p38 signaling in HK-2 cells is regulated through the activity of MKK3 and of DUSP10. The changes of MKK3 phosphorylation and DUSP10 expression after 48 h reflects a negative feedback loop. Furthermore, these findings demonstrate that acidosis in coculture has a particularly strong effect on the rapid activation of MAPK in HK-2 cells, while in fibroblasts the long-lasting effects are more important.

Besides p38, our data indicate an increased expression of phosphorylated JNK1/2 in HK-2 cells in coculture. JNK1/2 signaling is known to be involved in inflammatory response and dedifferentiation; therefore, its role in acidosis-induced inflammatory response and dedifferentiation in fibroblasts was assessed. Our data show that the acidosis-induced increase of N-cadherin was diminished in fibroblasts after inhibition of JNK1/2 (10 µM SP600125). This indicates that the acidosis-induced dedifferentiation is mediated partially by JNK1/2. In contrast, inhibition of JNK1/2 enhanced acidosis-induced COX-2 expression, excluding this kinase as mediator of the observed acidosis effects. It is known that MAPKs interact during feedback loops [28]. It is conceivable that the inhibition of JNK1/2 causes an enhanced activation of p38, explaining the measured stronger acidosis-induced elevation of COX-2. An alternative explanation for the observed effect is that JNK1/2 play a more protective role against acidosis-induced inflammatory response. In this study, the basic relevance of the interplay of acidosis and cellular crosstalk has been established. In future studies pH values between 7.4 and 6.4 have to be added to the experimental protocol to describe in more detail the shape of pH dependency.

Furthermore, we are testing a model with a transepithelial pH gradient (apical pH more acidic than basolateral pH) to approximate the situation in the tubule tissue.

Concerning the pH values applied in our study, it must be kept in mind that there exits not “the” physiological pH value nor “the” pathophysiological value but there are pH ranges under physiological and pathophysiological conditions. In the present study we chose an exemplary pH value (7.4) from the physiological and one from the pathophysiological range (6.4). This approach allowed us to identify the role of cellular crosstalk for the impact of acidosis as a matter of principle.

As there are no in vivo pH measurements of the tubulointerstitial space in humans, the precise threshold between the physiological and pathophysiological range is not settled, limiting the precise imitation of the in vivo situation in a cell culture model.

Because proximal tubular pH can decrease to values around 6.8 under physiological conditions, at least in the S3 segment, it seems valid to assume that a threshold for the transition from low physiological to pathophysiological pH is also in this range, and therefore the pH value of 6.4 represents a pathological micromilieu. This interpretation is supported by pH measurements under pathophysiological conditions (inflammation, ischemia, hypoxia) in other tissues [29–33]. Unfortunately, comparable data do not exist for human renal tissue. Therefore, we had to follow the findings obtained in other tissue types for our experimental setting. We believe this is a rational approach, and injured renal tissue will display a similar degree of acidification. Of note, the choice of the acidotic pH value was also chosen under the consideration of cell viability (“non-damaging acidotic pH value”), because a sudden necrotic or apoptotic cell death would not reflect the in vivo situation to be aimed at.

Investigation of pathophysiologically relevant crosstalk between cells of human origin, especially for the complex renal situation, is accompanied by certain limitations that result from the availability of suitable cells and from the limited information regarding the in vivo conditions in human tissue. In the present study, we have set up a polarized coculture system of an established human proximal tubule cell line together with a human fibroblast cell line, which mimics in a certain simplified manner the tubulointerstitial space. Of course, the cells used are not fully differentiated when compared with their human counterpart in vivo, although various important traits are expressed [34–37]. Comparing the results of the current study with previous studies using cells derived from rat kidney [26, 38], where the importance of cell–cell communication for the impact of microenvironmental acidosis, shows that cellular crosstalk is of major principal importance for acidosis impact on cell signalling and phenotype. However, the data also show that detailed cellular outcomes depend on the species. Of course, our findings have to be confirmed in the future. For this purpose, a coculture model with human primary proximal tubule cells could be a suitable alternative.

## Conclusions

Our data indicate that interstitial acidosis induces an inflammatory fibroblast phenotype only in conjunction with cellular crosstalk. This effect is partially mediated by the p38 and JNK1/2 MAPK-pathways. Moreover, we show that proximal tubule cells do not response with classical nephropathic changes, but rapid changes of signaling pathways. Thus, it can be assumed that proximal tubule cells act as modifier of the dyshomeostasis of the tubulointerstitial milieu. If these data also hold true in vivo, they demonstrate the key role of tubular-fibroblast crosstalk for milieu acidosis-induced inflammation during CKD development.

### Supplementary Information

**Supplementary Material 1.**

**Supplementary Material 2.**

## Data Availability

The datasets used and/or analysed during the current study are available from the corresponding author on reasonable request.

## Abbreviations

BSA: bovine serum
CDK: chronic kidney diseases
COX-2: cyclooxygenase
DUSP: dual specificity phosphatase
ECM: extracellular matrix
ELISA: enzyme-linked immunosorbent assay
ERK: extracellular signal-regulated kinase
FCS: fetal calf serum
IL: interleukin
JNK: c-Jun N-terminal kinase
LDH: lactate dehydrogenase
MAPK: mitogen-activated protein kinase
MMP: matrix metalloproteinases
MKK3: mitogen-activated protein kinase kinase 3
pHe: extracellular pH value
SDS-PAGE: sodium dodecyl sulfate–polyacrylamide gel electrophoresis
SEM: standard error of the mean
TBS: TRIS-buffered saline
TGF: transforming growth factor
TNF: tumor necrosis factor

## References

[1] Kovesdy CP (2022). Epidemiology of chronic kidney disease: an update 2022. Kidney Int Suppl.

[2] Schnaper HW (2017). The tubulointerstitial pathophysiology of progressive kidney disease. Adv Chronic Kidney Dis.

[3] Dong L (2013). Acidosis activation of the proton-sensing GPR4 receptor stimulates vascular endothelial cell inflammatory responses revealed by transcriptome analysis. PLoS ONE.

[4] Kellum JA, Song M, Li J (2004). Science review: extracellular acidosis and the immune response: clinical and physiologic implications. Crit Care.

[5] Wesson DE, Buysse JM, Bushinsky DA (2020). Mechanisms of metabolic acidosis-induced kidney injury in chronic kidney disease. J Am Soc Nephrol.

[6] Riemann A (2015). Acidic environment activates inflammatory programs in fibroblasts via a cAMP-MAPK pathway. Biochim Biophys Acta.

[7] Riemann A (2011). Acidic environment leads to ROS-induced MAPK signaling in cancer cells. PLoS ONE.

[8] Lue H (2006). Rapid and transient activation of the ERK MAPK signalling pathway by macrophage migration inhibitory factor (MIF) and dependence on JAB1/CSN5 and Src kinase activity. Cell Signal.

[9] Wang XY (2022). c-Myc-driven glycolysis polarizes functional regulatory B cells that trigger pathogenic inflammatory responses. Signal Transduct Target Ther.

[10] Kim HS, Asmis R (2017). Mitogen-activated protein kinase phosphatase 1 (MKP-1) in macrophage biology and cardiovascular disease. A redox-regulated master controller of monocyte function and macrophage phenotype. Free Radic Biol Med.

[11] Bretherton R (2020). Regulators of cardiac fibroblast cell state. Matrix Biol.

[12] Chen, H.F., H.C. Chuang, and T.H. Tan, Regulation of Dual-Specificity Phosphatase (DUSP) Ubiquitination and Protein Stability. Int J Mol Sci, 2019. 20(11). 10.3390/ijms20112668 PMC6600639 31151270

[13] Zarubin T, Han J (2005). Activation and signaling of the p38 MAP kinase pathway. Cell Res.

[14] Meran S, Steadman R (2011). Fibroblasts and myofibroblasts in renal fibrosis. Int J Exp Pathol.

[15] Sulikowska B (2015). The role of interstitial changes in the progression of chronic kidney disease. Postepy Hig Med Dosw (Online).

[16] Magno AL (2019). Current knowledge of IL-6 cytokine family members in acute and chronic kidney disease. Biomedicines.

[17] Ferenbach DA, Bonventre JV (2016). Kidney tubules: intertubular, vascular, and glomerular cross-talk. Curr Opin Nephrol Hypertens.

[18] Prunotto M (2012). Epithelial-mesenchymal crosstalk alteration in kidney fibrosis. J Pathol.

[19] Ryan MJ (1994). HK-2: an immortalized proximal tubule epithelial cell line from normal adult human kidney. Kidney Int.

[20] Gunness P (2010). Comparison of the novel HK-2 human renal proximal tubular cell line with the standard LLC-PK1 cell line in studying drug-induced nephrotoxicity. Can J Physiol Pharmacol.

[21] Guo Y (2014). Parathyroid hormone induces epithelial-to-mesenchymal transition via the Wnt/beta-catenin signaling pathway in human renal proximal tubular cells. Int J Clin Exp Pathol.

[22] Korsunsky I (2022). Cross-tissue, single-cell stromal atlas identifies shared pathological fibroblast phenotypes in four chronic inflammatory diseases. Med.

[23] Buechler MB (2021). Cross-tissue organization of the fibroblast lineage. Nature.

[24] Gekle M (2002). NHE3 serves as a molecular tool for cAMP-mediated regulation of receptor-mediated endocytosis. Am J Physiol Renal Physiol.

[25] Schwerdt G (2007). Long-term effects of ochratoxin A on fibrosis and cell death in human proximal tubule or fibroblast cells in primary culture. Toxicology.

[26] Schulz MC (2022). Epithelial-fibroblast crosstalk protects against acidosis-induced inflammatory and fibrotic alterations. Biomedicines.

[27] Riemann A (2016). Impact of the tumor microenvironment on the expression of inflammatory mediators in cancer cells. Adv Exp Med Biol.

[28] Cheung PC (2003). Feedback control of the protein kinase TAK1 by SAPK2a/p38alpha. EMBO J.

[29] Hunt JF (2000). Endogenous airway acidification. Implications for asthma pathophysiology. Am J Respir Crit Care Med.

[30] Ward TT, Steigbigel RT (1978). Acidosis of synovial fluid correlates with synovial fluid leukocytosis. Am J Med.

[31] Farr M (1985). Significance of the hydrogen ion concentration in synovial fluid in rheumatoid arthritis. Clin Exp Rheumatol.

[32] Cartwright IM (2021). Mucosal acidosis elicits a unique molecular signature in epithelia and intestinal tissue mediated by GPR31-induced CREB phosphorylation. Proc Natl Acad Sci U S A.

[33] Ferrara A (1990). Hypothermia and acidosis worsen coagulopathy in the patient requiring massive transfusion. Am J Surg.

[34] Chonlaket P, Wongwan T, Soodvilai S (2018). Liver X receptor activation inhibits SGLT2-mediated glucose transport in human renal proximal tubular cells. Exp Physiol.

[35] Chang CY (2014). FXYD2c plays a potential role in modulating Na(+)/K (+)-ATPase activity in HK-2 cells upon hypertonic challenge. J Membr Biol.

[36] Wang Q, Lu Y, Morris ME (2007). Monocarboxylate transporter (MCT) mediates the transport of gamma-hydroxybutyrate in human kidney HK-2 cells. Pharm Res.

[37] Wu Y (2016). Oxidative stress-activated NHE1 Is involved in high glucose-induced apoptosis in renal tubular epithelial cells. Yonsei Med J.

[38] Schulz MC (2023). Acidosis activates the Nrf2 pathway in renal proximal tubule-derived cells through a crosstalk with renal fibroblasts. Antioxidants (Basel).

