# Supplementary material for: Crosstalk with renal proximal tubule cells drives acidosis-induced inflammatory response and dedifferentiation of fibroblasts via p38-singaling
Source: Cell Commun Signal. 2024 Feb 24;22:148. doi: 10.1186/s12964-024-01527-8 (PMC10893741; doi:10.1186/s12964-024-01527-8)
Supplement: Supplementary file 1 — Supplementary Material 1. [file 12964_2024_1527_MOESM1_ESM.docx]

**Supplementary material table of content**

Supplementary figure 1 (S1): Determination of acidosis resilience for HK-2 and CCD-1092Sk in mono-and coculture.

Supplementary figure 2 (S2): Impact of extracellular pH 6.0 on inflammation markers in HK-2 and CCD-1092Sk cells in mono-and coculture.

Supplementary figure 3 (S3): Impact of extracellular pH 6.0 on differentiation marker proteins in HK-2 and CCD-1092Sk cells in mono-and coculture.

Supplementary figure 4 (S4): Impact of extracellular pH 6.0 on fibrosis markers in HK-2 and CCD-1092Sk cells in mono- and coculture

Supplementary figure 5 (S5): Original tracing curves for BCECF-measurement.

Supplementary Table 1 Summary of acidosis (6.4) effects

Supplementary Table 2 Summary of acidosis (6.0) effects

Supplementary Table 3 Antibodies

Supplementary table 4 buffer composition

**S1**

**
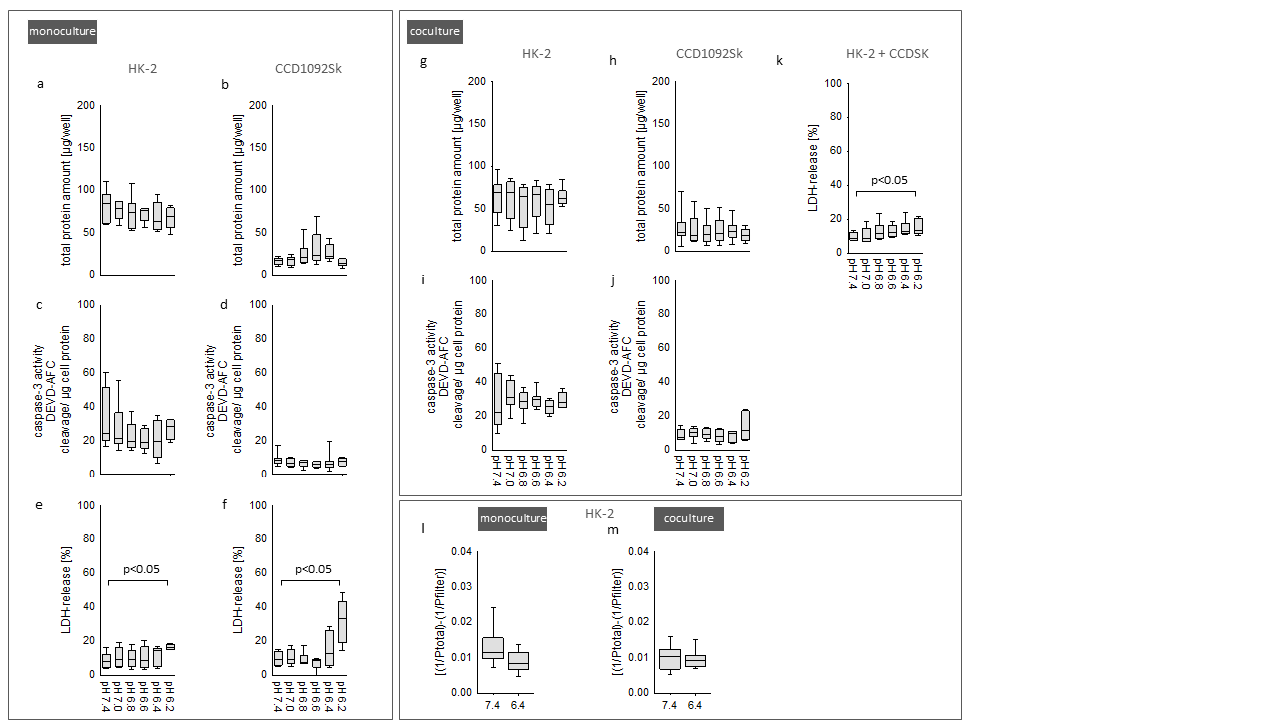
**

Supplementary figure 1 Determination of acidosis resilience for HK-2 and CCD-1092Sk in mono-and coculture. Effect of acidosis on total protein amount (a, b, g, h), caspase-3 activity (c, d, i, j), LDH release (e, f, k), diffusion of FITC-Dextran (l, m). n = 6-10.

S1 methods

S1.1 Caspase-3 activity assay

Protein fractions of the cells were obtained after 30 min incubation on ice with 100 µl cell lysis buffer and used to determine caspase-3 activity. 60 µl of the sample was incubated with 60 µl of Caspase-reaction buffer and 42 µM DEVD-AFC (end concentration) for 90 min at 37°C. Fluorescence of the cleaved product AFC was measured with a plate reader (Infinite M200, Tecan) at 400 nm excitation and 505 nm emission wavelengths. Cleaved AFC was quantified using a calibration curve with known AFC concentrations and normalized to the total amount of protein contained in the sample (determined by BCA assay).

S1.2 Lactate dehydrogenase (LDH) assay

LDH activity was measured after a standard protocol according to Bergmeyer et al. 1974 (Bergmeyer, H.U. and Bernt, E. (1974) Methoden der enzymatischen Analyse. Verlag Chemie, Weinheim.). Cell media and cell lysates were incubated with LDH substrate buffer, and the turnover of LDH substrates was measured at 334 nm (NADH) for 30 minutes. The relative LDH release was calculated as LDH activity in the media divided by the total LDH activity (=media + lysate) and used as surrogate marker for necrosis.

S1.3 FITC-Dextran diffusion.

HK-2 cells were seeded on the filter inserts (Falcon, Tewksbury, USA) in a 24-well plate. 300 µl of media was added to the apical compartment, and 500 µl was added to the basolateral compartment. After incubation with acidic media for 24 h (pH 7.4-6.2), 1 g/L FITC-dextran (70 kDa) was applied to the apical compartment for an additional 24 h. Six hours and 24 h after adding FITC-dextran, 25 µl media aliquots were sampled from the basolateral and apical compartments. Before the measurement, 100 µl HEPES-Ringer buffer was added to each sample. Fluorescence of FITC-Dextran was measured at 400 nm excitation and 505 nm emission wavelength using a multiwell reader (Infinite® M200, Tecan, Germany). Afterwards the slope according to time was calculated and the reciprocal value was used as marker for cellular resistance.

**S2**

**
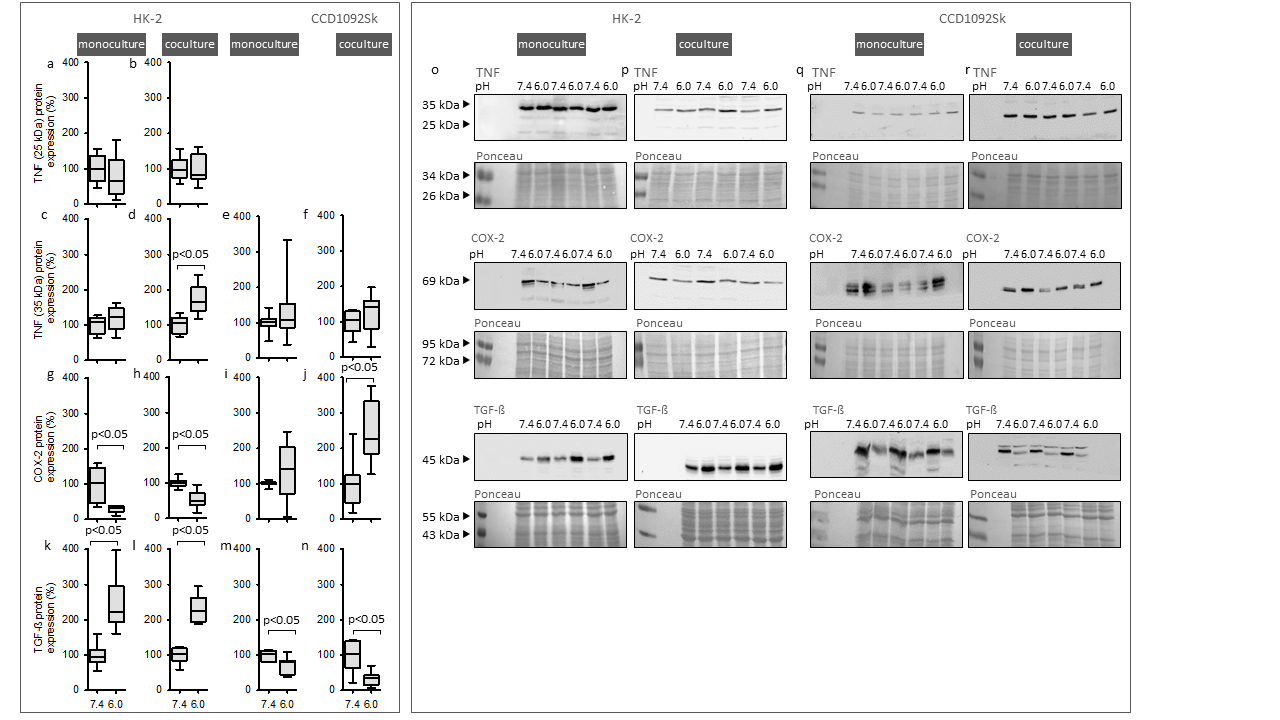
**

Supplementary figure 2 Effect of acidic media on inflammation markers in HK-2 and CCD-1092Sk cells in mono-and coculture. Protein expression changes of TNF (a, b, e, f, i, j), COX-2 (c, g, k, l) and TGF-ß (d, h, m, n). Representative western blots of proteins isolated from cells exposed to acidosis (o-z), n = 6-9. exposure time = 48 h, pH 6.0.

S2 Impact of media with pH 6.0 on inflammation markers in HK-2 and CCD-1092Sk cells.

The results so far show that the proximal tubular cells react little to the maximum non-damaging acidic pH value. To test whether a lower pH favors inflammatory response or dedifferentiation, we lowered the pH to 6.0.

S2 shows that an exposure to media containing of pH 6.0 causes a decrease of COX-2 and an increase of TGF-ß expression in HK-2 cells (S2 c, d). Under co-culture conditions, an additional increase of TNF protein expression was observed (S2 f-h). In fibroblasts, exposure to media, with pH 6.0, led to an increase of COX-2 protein expression and a decrease of TGF-ß protein expression in co-culture (S2 l, n).

**S3**

**
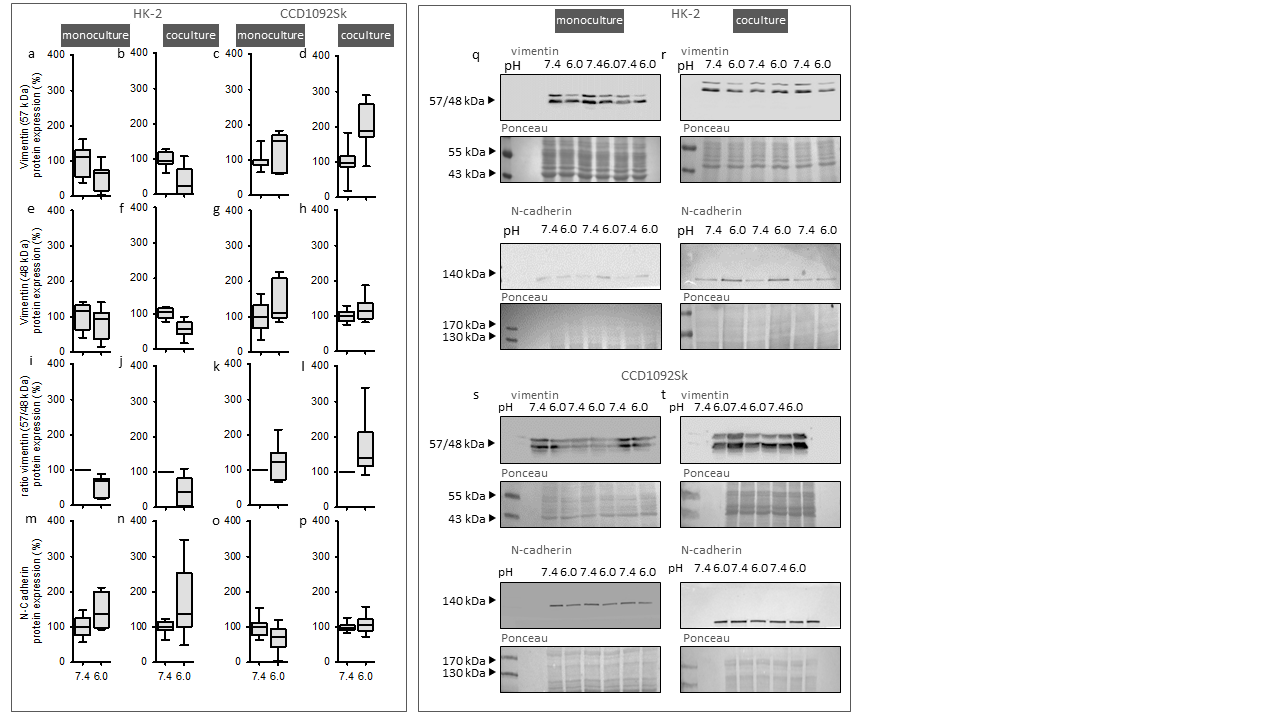
**

Supplementary figure 3 Effect of acidic media on differentiation marker proteins in HK-2 and CCD-1092Sk cells in mono-and coculture. Protein expression changes of vimentin (a-d, i-l) and N-cadherin (d, n). Representative western blots of proteins isolated from cells exposed to acidosis (q-x). n = 6 - 12. exposure time = 48 h, pH 6.0.

S3 Influence of extracellular pH 6.0 on markers for differentiation in HK-2 and CCD-1092Sk cells.

HK-2 cells in monoculture responded with decreased expression of full-length vimentin as well as and the vimentin ratio (S3 a, c). Under coculture conditions, acidosis led to a decrease of both vimentin fragments and the vimentin ratio. Furthermore, the expression of N-cadherin was unaltered (S3 e-h). In fibroblasts in monoculture, the acidic media induced a decreased protein expression of N-cadherin (S3 l). Under co-culture conditions, the exposure to acidic media led to an increase of the full-length vimentin and the vimentin ratio (S3 m, n).

S4 Impact of media with pH 6.0 on markers for fibrosis in HK-2 and CCD-1092Sk cells.

S4 shows, that HK-2 cells in monoculture respond with a decrease of intracellular collagen III and fibronectin expression as well as fibronection secretion, whilst the secretion of collagen III is unchanged (S3 a, b, q, r). Under co-culture conditions, the acidosis-effect on fibronectin but not on collagen III remains (S3 c, d). In fibroblasts in monoculture, acidic media led to a decreased expression of intracellular collagen III but not of fibronectin or the secretion of collagen III and fibronectin (S3 s, t). Under co-culture conditions intracellular fibronectin but not collagen III was increased (S3 i, l). S4 u and v shows that acidosis induced a decreased secretion of collagen III and fibronectin in co-culture.

**S4**

**
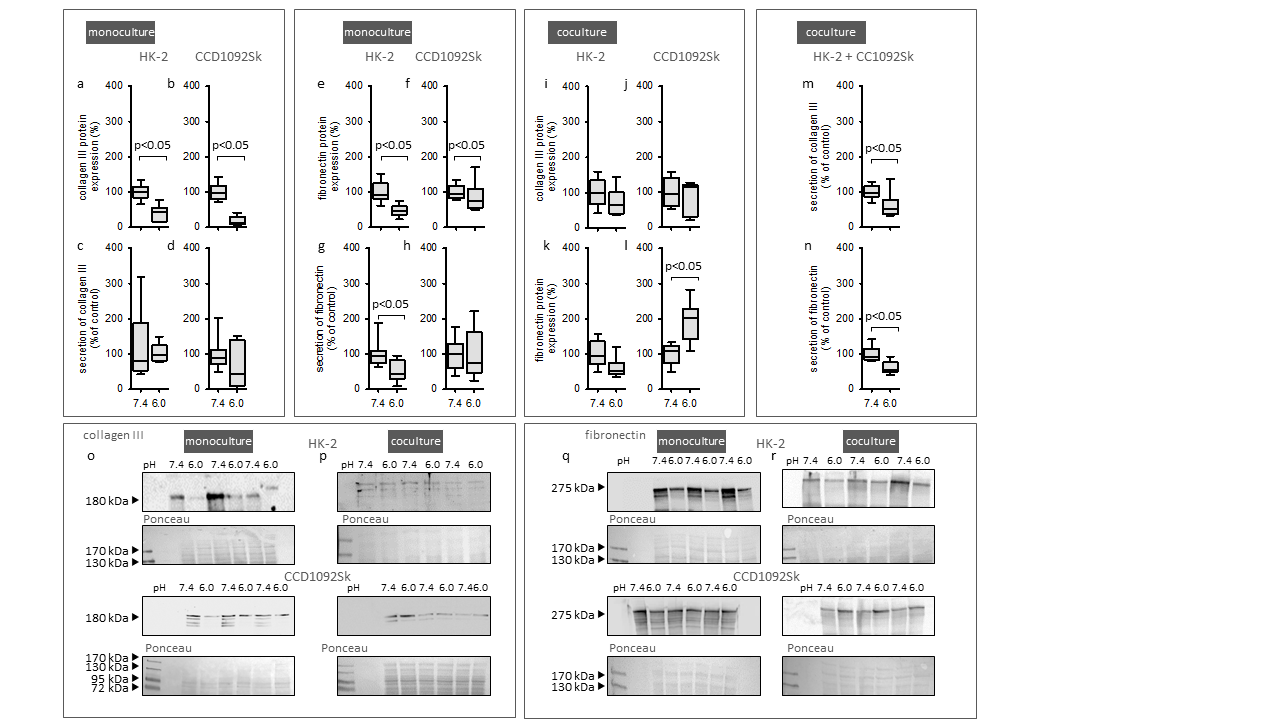
**

Supplementary figure 4 Effect of acidic media on fibrosis markers in HK-2 and CCD-1092Sk cells in mono- and coculture. Protein expression changes of intracellular and secreted collagen III (a, c, i, k) and fibronectin (b, d, j, l). Representative western blots of proteins isolated from cells exposed to acidosis (e-h, m-p). n = 7 - 12. exposure time = 48 h, pH 6.0.


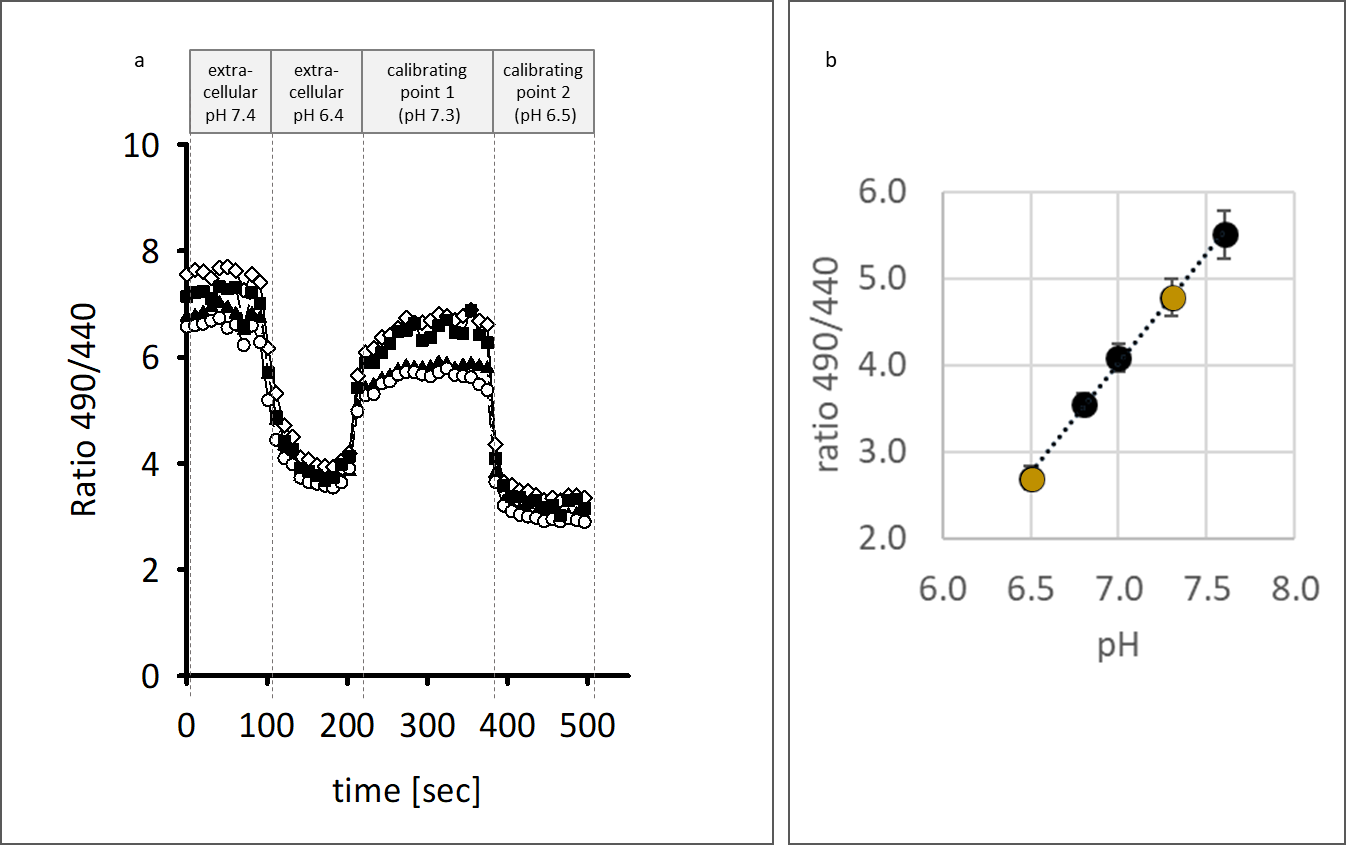


Supplementary figure 5 Original tracing curves for the BCECF-method (a) and for the calibration with the nigericin-method (b). Change of intracellular fluorescence ratio over time. The cells were incubated with ringer, containing the experimental pH values of first 7.4 (first plateau) and, 6.4 (second plateau). Afterwards, cells were incubated with calibration solution, containing nigericin and a pH value of 7.3 (third plateau) and 6.5 (forth plateau) (a). B depicts a representative detailed calibration curve. The yellow points depict the actual calibration measurements of the used two-point-calibration.

Supplementary table 1 Summary of acidosis (pH 6.4) effects.

|  | **HK-2 pH 6.4** | | **CCD-1092Sk pH 6.4** | |
| --- | --- | --- | --- | --- |
| **Target** | **monoculture** | **coculture** | **monoculture** | **coculture** |
| **Viability** | Ø | Ø | Ø | Ø |
| **Marker for inflammation** | | | | |
| **TNF** | Ø | Ø | Ø | **↑** |
| **COX-2** | **↓** | **↓** | **↓** | **↑** |
| **TGF-ß** |  |  |  | **↓** |
| **Marker for differentiation** | | | | |
| **Vimentin** | **↓** | **↓** | Ø | **↑** |
| **N-cadherin** | Ø | Ø | Ø | **↑** |
| **ß-Catenin** | **↓** | **↓** | Ø | Ø |
| **Vinculin** | Ø | Ø | Ø | **↑** |
| **Epithelial barrier** | Ø | Ø | Ø | Ø |
| **Marker for fibrosis** | | | | |
| **Collagen III (IC)** | **↑** | **↓** | **↓** | **↓** |
| **Fibronectin (IC)** | **↓** | Ø | **↓** | Ø |
| **secreted collagen III** | **↑** | n.d. | Ø | n.d. |
| **secreted fibronectin** | Ø | n.d. | Ø | n.d. |
| **MAPK-Signaling** | | | | |
| **P-ERK1/2** | Ø | Ø | **↓** | **↓** |
| **ERK1/2** | Ø | Ø | Ø | Ø |
| **p-JNK1/2** | Ø | **↑** | Ø | Ø |
| **JNK1/2** | Ø | Ø | Ø | Ø |
| **P-p38** | Ø | **↑** | **↓** | **↑** |
| **p38** | Ø | Ø | Ø | **↓** |
| **DUSP1** | n.d. | Ø | n.d. | **↑** |
| **DUSP4** | n.d. | Ø | n.d. | **↓** |

↑ - upregulated ↓ - downregulated Ø – not altered n.d. – not detected

Supplementary table 2 Summary of acidosis (6.0) effects.

|  | **HK-2 pH 6.0** | | **CCD-1092Sk pH 6.0** | |
| --- | --- | --- | --- | --- |
| **Target** | **monoculture** | **coculture** | **monoculture** | **coculture** |
| **Viability** | **↓** | **↓** | **↓** | **↓** |
| **Marker for inflammation** | | | | |
| **TNF** | Ø | **↑** | Ø | Ø |
| **COX-2** | **↓** | **↓** | Ø | **↑** |
| **TGF-ß** | **↑** | **↑** | **↓** | **↓** |
| **Marker for differentiation** | | | | |
| **Vimentin** | **↓** | **↓** | Ø | **↑** |
| **N-cadherin** | Ø | **↑** | **↓** | Ø |
| **Marker for fibrosis** | | | | |
| **Collagen III (IC)** | **↓** | Ø | **↓** | Ø |
| **Fibronectin (IC)** | **↓** | **↓** | Ø | **↑** |
| **secreted collagen III** | Ø | n.d. | Ø | n.d. |
| **secreted fibronectin** | **↓** | n.d. | Ø | n.d. |

↑ - upregulated ↓ - downregulated Ø – not altered n.d. – not detected

Supplementary table 3 Antibodies, order number, host and dilutions used.

| **Target** | **Company** | **Order number** | **Host** | **Dilution** |
| --- | --- | --- | --- | --- |
| N-Cadherin | Cell Signaling, Danvers, USA | 14215 | Mouse | 1:500 |
| ß-Catenin | Cell Signaling, Danvers, USA |  | Rabbit | 1:1000 |
| COX-2 | Abcam, Cambridge, UK | ab52237 | Rabbit | 1:500 |
| Collagen, Type III | Biomol, Hamburg; Ger | 600-401-105 | Rabbit | 1:500 |
| DUSP10 | Cell Signaling, Danvers, USA | 3483 | Rabbit | 1:500 |
| ERK 1/2 (MAPK, p44/42-, (L34F12) | Cell Signaling, Danvers, USA | 4696 | Mouse | 1:1000 |
| P-ERK1/2 (Phospho-p44/p42-) | Cell Signaling, Danvers, USA | 9101 | Rabbit | 1:1000 |
| Fibronectin | Biomol, Hamburg; Ger | 600-401-117 | Rabbit | 1:500 |
| JNK 1/2 | Cell Signaling, Danvers, USA | 9252 | Rabbit | 1:1000 |
| P-JNK 1/2 | Cell Signaling, Danvers, USA | 9251 | Rabbit | 1:1000 |
| MKK3 | Cell Signaling, Danvers, USA | 5674 | Rabbit | 1:1000 |
| P-MKK3 | Cell Signaling, Danvers, USA | 12280 | Rabbit | 1:1000 |
| P38 | Cell Signaling, Danvers, USA | 9212 | Rabbit | 1:1000 |
| P-p38 | Cell Signaling, Danvers, USA | 9216 | Rabbit | 1:1000 |
| TGF-ß | Cell Signaling, Danvers, USA | 3711 | Rabbit | 1:1000 |
| TNF-α | Cell Signaling, Danvers, USA | 6945 | Rabbit | 1:500 |
| Vimentin | Cell Signaling, Danvers, USA | 5741 | Rabbit | 1:1000 |
| Vinculin | Cell Signaling, Danvers, USA | 13901 | Rabbit | 1:1000 |
| Anti-Mouse IgG HRP | Cell Signaling, Danvers, USA | 7076 | horse | 1:2000 |
| Anti-Rabbit IgG HRP | Cell Signaling, Danvers, USA | 7074 | goat | 1:2000 |

Abbreviations: COX-2 cyclooxygenase 2,DUSP Dual-specificity phosphatase, ERK extracellular signal-regulated kinases, HRP horseradish peroxidase, JNK c-Jun N-terminal kinases, MAPK Mitogen-activated protein kinases, TGF-ß transforming growth factor-ß, TNF tumor necrosis factor.

Supplementary table 4 Buffer composition

| **Buffer** | **Composition** |
| --- | --- |
| Calibration Solution (for intracellular pH) | - 132 mM KCl - 1 mM CaCl_2_ - 1 mM MgCl_2_ - 10 mM HEPES - 0.01 mM Nigericin |
| caspase-3 reaction buffer | - 10 mM PIPES - 2 mM EDTA - 0.1 % CHAPS - 1 % DTT (freshly added) - pH 7.5 |
| Calibration Solution for cytosolic pH measurements | - 132 mM KCl - 1 mM CaCl_2_ - 1 mM MgCl_2_ - 10 mM HEPES - 0.01 mM Nigericin |
| EDTA buffer | - 136.8 mM NaCl - 2.68 mM KCl - 8.1 mM Na_2_HPO_4_ - 2 mM H_2_PO_4_^-^ - 0,7 mM EDTA - pH 7,2 |
| HEPES buffer | - 122.5 mM NaCl - 5.5 mM KCl - 0.8 mM MgCl_2_ x 6H_2_O - 1.2 mM CaCl_2_ x 2H_2_O - 1 mM NaH_2_PO_4_ x H_2_0 - 10 mM HEPES - pH 7.4 |
| HRP-Substrate | - 0.015 % (v/v) H_2_O_2_ (30%) - 0.46 mM ortho-phenylendiamine - 6.6 mM Na_2_HPO_4_ · 2H_2_O - 7.8 mM citric acid |
| MOPS-Triton buffer | - 10 mM TRIS base - 20 mM MOPS - 100 mM NaCl - 1 mM EDTA - 0.01 % Triton X-100 - pH adjusted to 7.5 |
| 1x PBS-TWEEN | - 1370 mM NaCl - 27 mM KCl - 81 mM Na_2_HPO_4_ * 2 H_2_O - 15 mM KH_2_PO_4_ - 1 % TWEEN 20 - pH 7.4 |
| 6 x Redmix/Lämmli buffer | - 124.8 mM TRIS HCl - 6 % SDS - 1,42 mM ß-Mercaptoethanol - 4,7 mM Glycerol - 155.2 mM Bromphenol Blue - pH 6.8 |
| Running buffer | - 25 mM TRIS - 3,5 mM SDS - 192 mM glycine |
| Ringer solution | - 24 mM NaHCO_3_ - 0.8 mM Na_2_HPO_4_ - 0.2 mM NaH_2_PO_4_ - 87 mM NaCl - 5.4 mM KCl - 1.2 mM CaCl_2_ - 0.8 mM MgCl_2_ - 25 mM HEPES - 5.5 mM Glucose |
| 1×Turbo-Transferbuffer | - 20 % 5× Transferbuffer (Bio-Rad, Feldkirchen, Ger) - 20 % ethanol - 60 % pure water |
| Trypsin solution | - 154 mM NaCl - 2.7 mM KCl - 8.2 mM Na_2_HPO_4_ - 1.5 mM KH_2_PO_4_ - 0,7 mM EDTA - 0,2 mM streptomycin - 0,18 mM penicillin - 0.02 mM trypsin - pH 7,1-7,3 |
| 1xTBS TWEEN | - 3 mM TRIS base - 140 mM NaCl - 0.17 mM TRIS-HCl - 1 % TWEEN 20 - pH 7.4 |
| Vollers buffer | - 10.2 mM Na_2_CO_3_ - 34.9 mM NaHCO_3_ |
