# Supplementary material for: Crosstalk with renal proximal tubule cells drives acidosis-induced inflammatory response and dedifferentiation of fibroblasts via p38-singaling
Source: Cell Commun Signal. 2024 Feb 24;22:148. doi: 10.1186/s12964-024-01527-8 (PMC10893741; doi:10.1186/s12964-024-01527-8)
Supplement: Supplementary file 2 — Supplementary Material 2. [file 12964_2024_1527_MOESM2_ESM.pptx]

## Slide 1
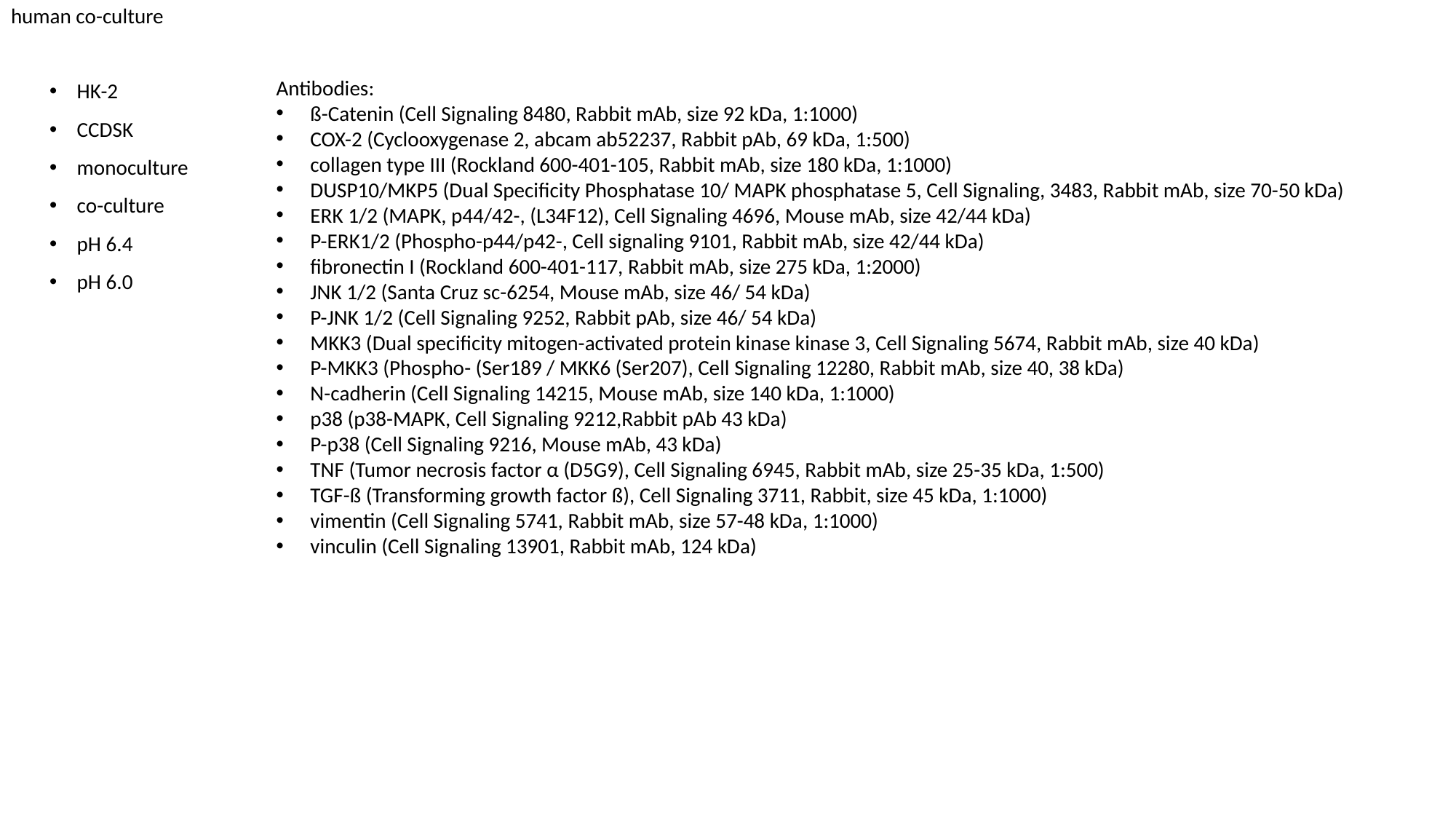

# human co-culture
Antibodies:
ß-Catenin (Cell Signaling 8480, Rabbit mAb, size 92 kDa, 1:1000)
COX-2 (Cyclooxygenase 2, abcam ab52237, Rabbit pAb, 69 kDa, 1:500)
collagen type III (Rockland 600-401-105, Rabbit mAb, size 180 kDa, 1:1000)
DUSP10/MKP5 (Dual Specificity Phosphatase 10/ MAPK phosphatase 5, Cell Signaling, 3483, Rabbit mAb, size 70-50 kDa)
ERK 1/2 (MAPK, p44/42-, (L34F12), Cell Signaling 4696, Mouse mAb, size 42/44 kDa)
P-ERK1/2 (Phospho-p44/p42-, Cell signaling 9101, Rabbit mAb, size 42/44 kDa)
fibronectin I (Rockland 600-401-117, Rabbit mAb, size 275 kDa, 1:2000)
JNK 1/2 (Santa Cruz sc-6254, Mouse mAb, size 46/ 54 kDa)
P-JNK 1/2 (Cell Signaling 9252, Rabbit pAb, size 46/ 54 kDa)
MKK3 (Dual specificity mitogen-activated protein kinase kinase 3, Cell Signaling 5674, Rabbit mAb, size 40 kDa)
P-MKK3 (Phospho- (Ser189 / MKK6 (Ser207), Cell Signaling 12280, Rabbit mAb, size 40, 38 kDa)
N-cadherin (Cell Signaling 14215, Mouse mAb, size 140 kDa, 1:1000)
p38 (p38-MAPK, Cell Signaling 9212,Rabbit pAb 43 kDa)
P-p38 (Cell Signaling 9216, Mouse mAb, 43 kDa)
TNF (Tumor necrosis factor α (D5G9), Cell Signaling 6945, Rabbit mAb, size 25-35 kDa, 1:500)
TGF-ß (Transforming growth factor ß), Cell Signaling 3711, Rabbit, size 45 kDa, 1:1000)
vimentin (Cell Signaling 5741, Rabbit mAb, size 57-48 kDa, 1:1000)
vinculin (Cell Signaling 13901, Rabbit mAb, 124 kDa)
HK-2
CCDSK
monoculture
co-culture
pH 6.4
pH 6.0

## Slide 2
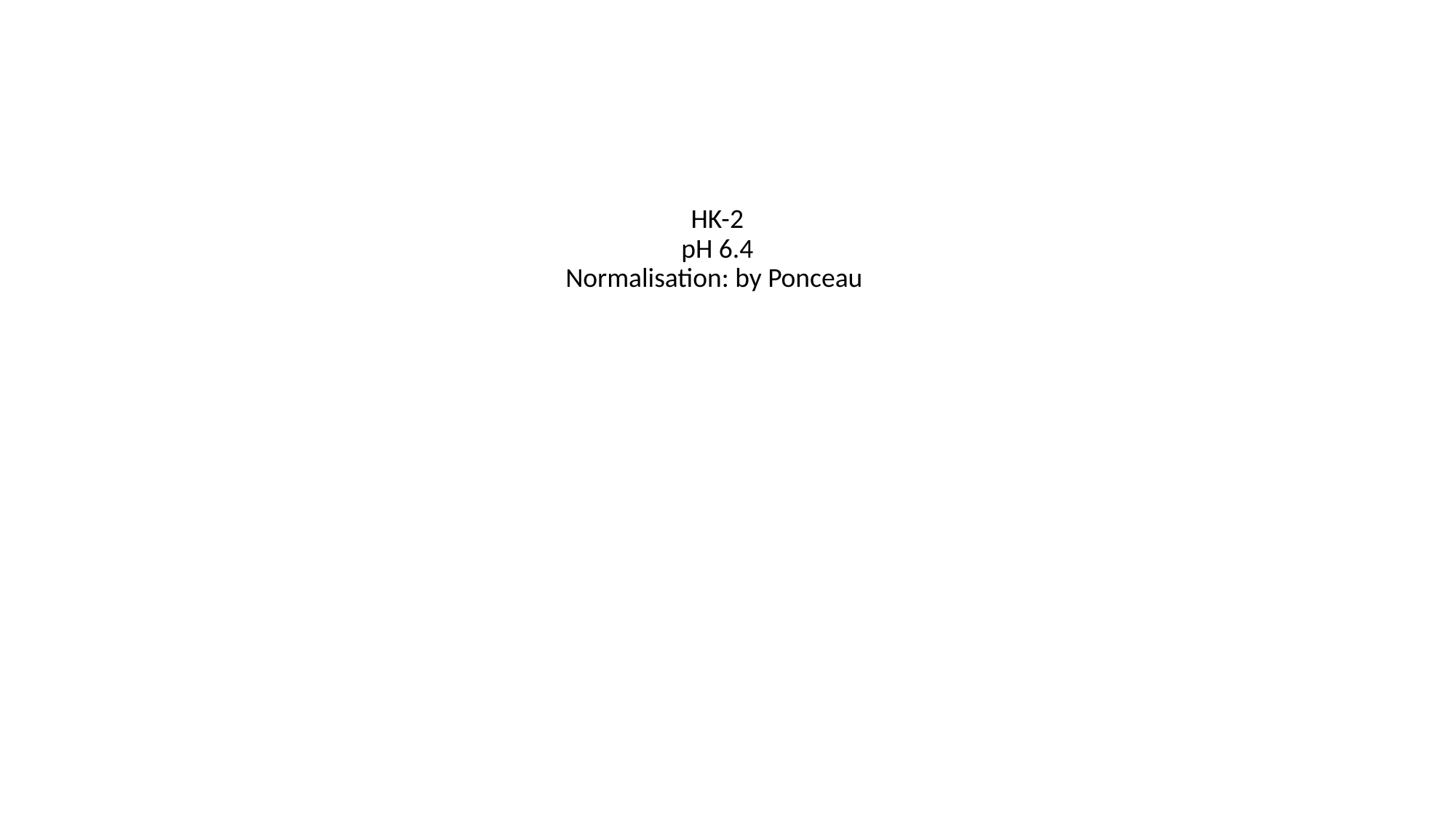

# HK-2 pH 6.4 Normalisation: by Ponceau

## Slide 3
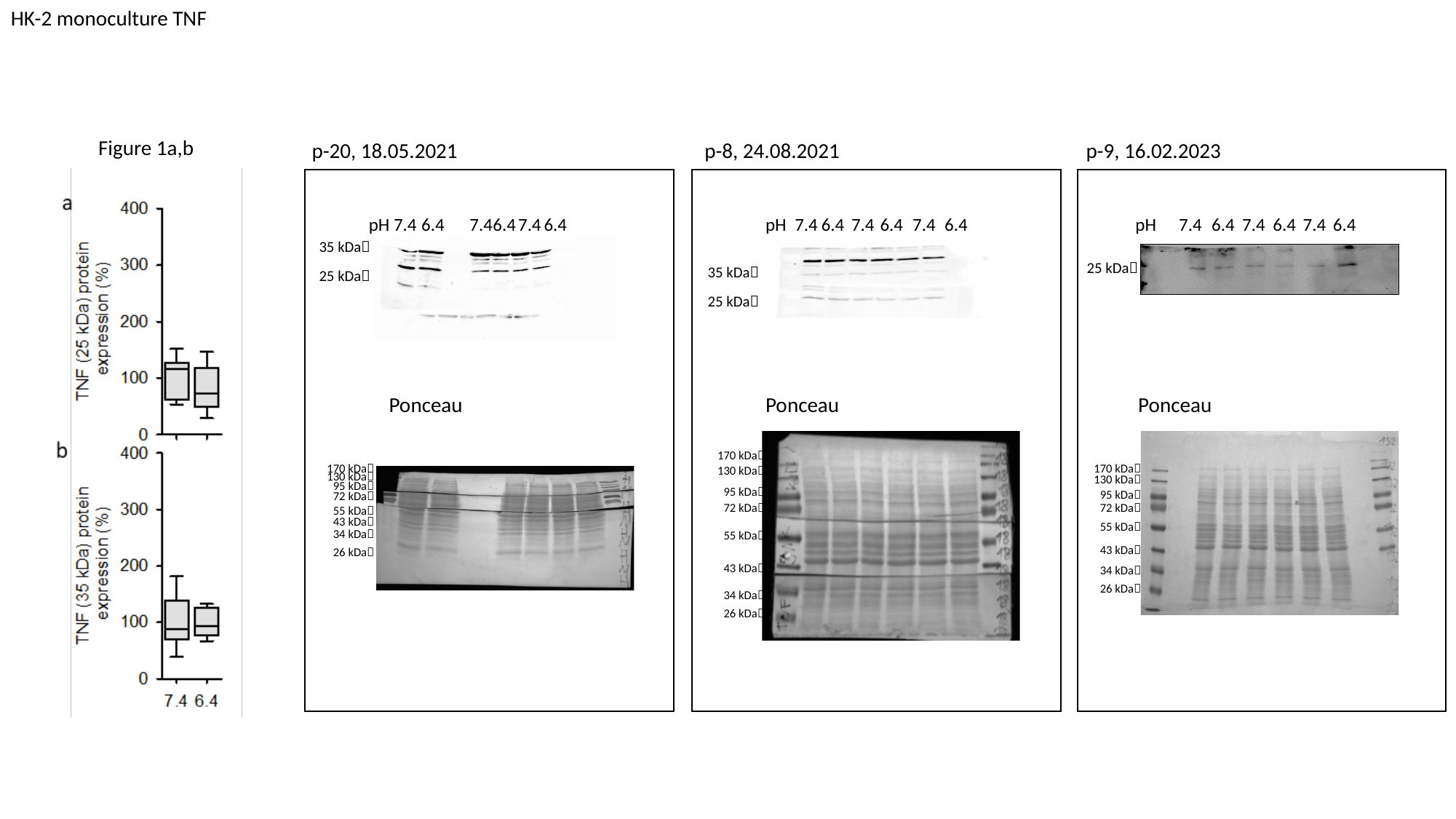

HK-2 monoculture TNF
Figure 1a,b
p-20, 18.05.2021
p-8, 24.08.2021
p-9, 16.02.2023
pH
7.4
6.4
7.4
6.4
7.4
6.4
pH
7.4
6.4
7.4
6.4
7.4
6.4
pH
7.4
6.4
7.4
6.4
7.4
6.4
35 kDa
25 kDa
35 kDa
25 kDa
25 kDa
Ponceau
Ponceau
Ponceau
170 kDa
130 kDa
95 kDa
72 kDa
55 kDa
43 kDa
34 kDa
26 kDa
170 kDa
130 kDa
95 kDa
72 kDa
55 kDa
43 kDa
34 kDa
26 kDa
170 kDa
130 kDa
95 kDa
72 kDa
55 kDa
43 kDa
34 kDa
26 kDa

## Slide 4
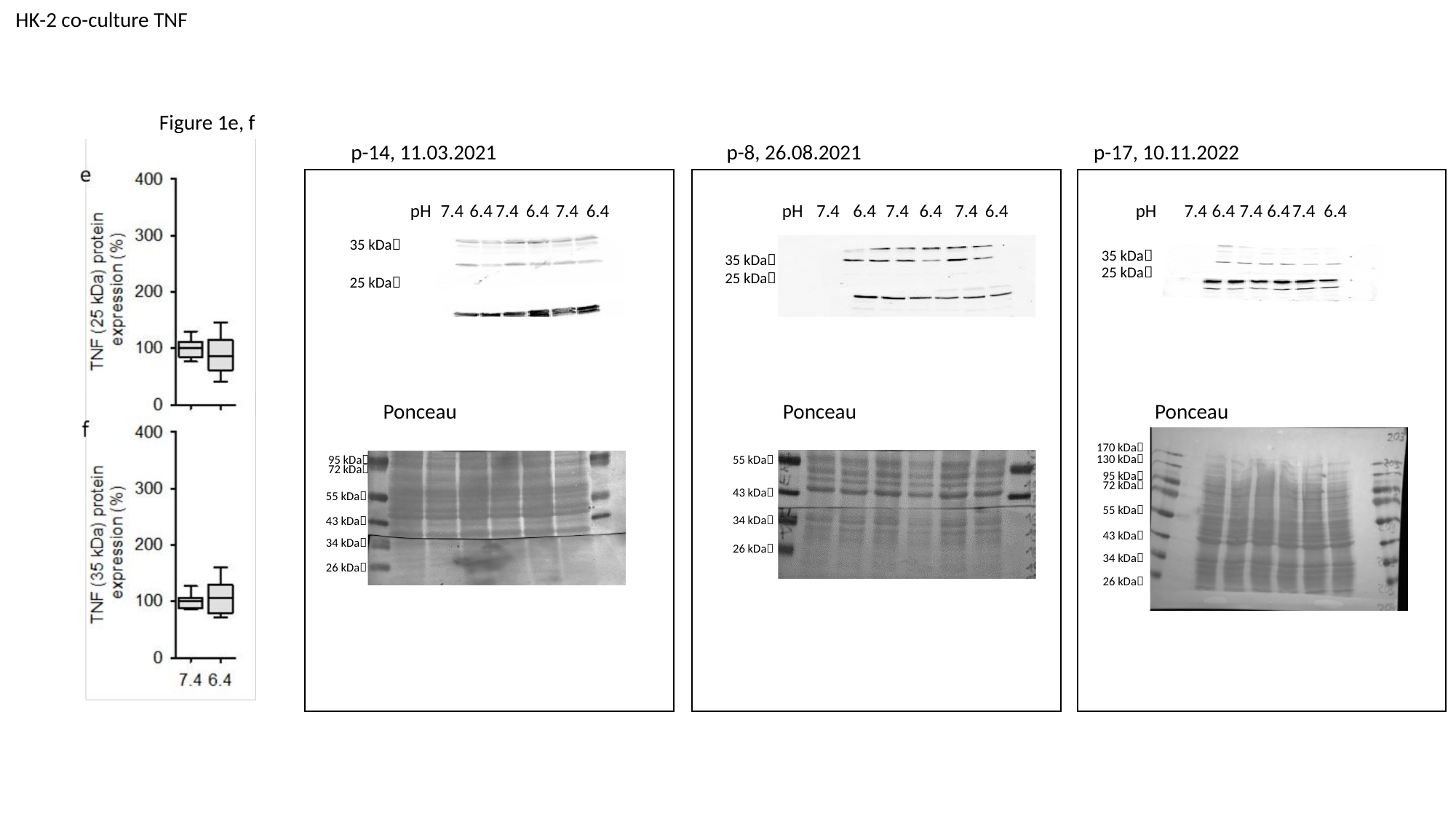

HK-2 co-culture TNF
Figure 1e, f
p-14, 11.03.2021
p-8, 26.08.2021
p-17, 10.11.2022
pH
7.4
6.4
7.4
6.4
7.4
6.4
pH
7.4
6.4
7.4
6.4
7.4
6.4
pH
7.4
6.4
7.4
6.4
7.4
6.4
35 kDa
35 kDa
35 kDa
25 kDa
25 kDa
25 kDa
Ponceau
Ponceau
Ponceau
170 kDa
130 kDa
95 kDa
72 kDa
55 kDa
43 kDa
34 kDa
26 kDa
55 kDa
43 kDa
34 kDa
26 kDa
95 kDa
72 kDa
55 kDa
43 kDa
34 kDa
26 kDa

## Slide 5
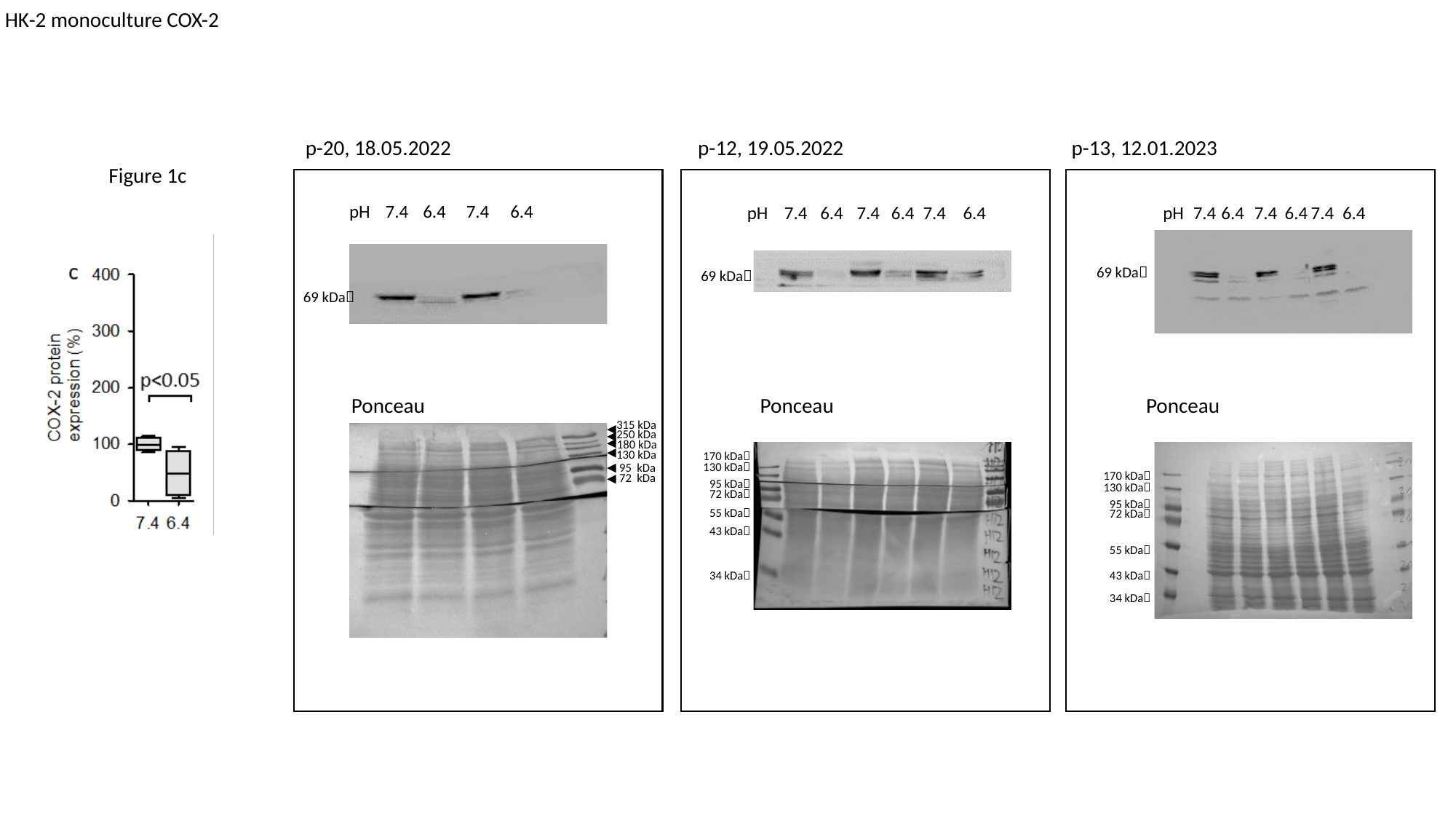

HK-2 monoculture COX-2
p-20, 18.05.2022
p-12, 19.05.2022
p-13, 12.01.2023
Figure 1c
pH
7.4
6.4
7.4
6.4
pH
7.4
6.4
7.4
6.4
7.4
6.4
pH
7.4
6.4
7.4
6.4
7.4
6.4
69 kDa
69 kDa
69 kDa
Ponceau
Ponceau
Ponceau
315 kDa
250 kDa
180 kDa
130 kDa
170 kDa
130 kDa
95 kDa
72 kDa
55 kDa
43 kDa
34 kDa
 95 kDa
170 kDa
130 kDa
95 kDa
72 kDa
55 kDa
43 kDa
34 kDa
 72 kDa

## Slide 6
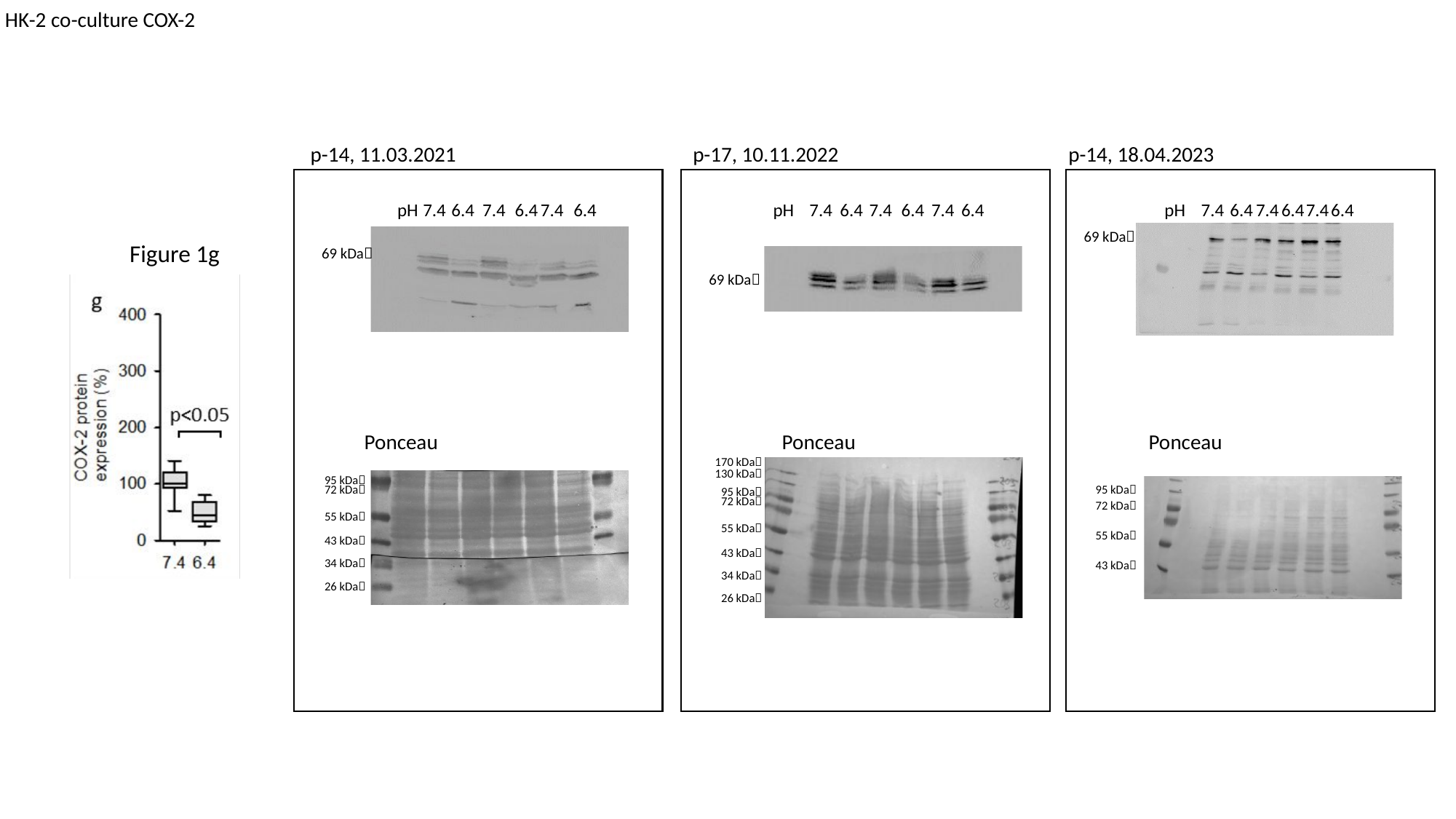

HK-2 co-culture COX-2
p-14, 11.03.2021
p-17, 10.11.2022
p-14, 18.04.2023
pH
7.4
6.4
7.4
6.4
7.4
6.4
pH
7.4
6.4
7.4
6.4
7.4
6.4
pH
7.4
6.4
7.4
6.4
7.4
6.4
69 kDa
Figure 1g
69 kDa
69 kDa
Ponceau
Ponceau
Ponceau
170 kDa
130 kDa
95 kDa
72 kDa
55 kDa
43 kDa
34 kDa
26 kDa
95 kDa
72 kDa
55 kDa
43 kDa
34 kDa
26 kDa
95 kDa
72 kDa
55 kDa
43 kDa

## Slide 7
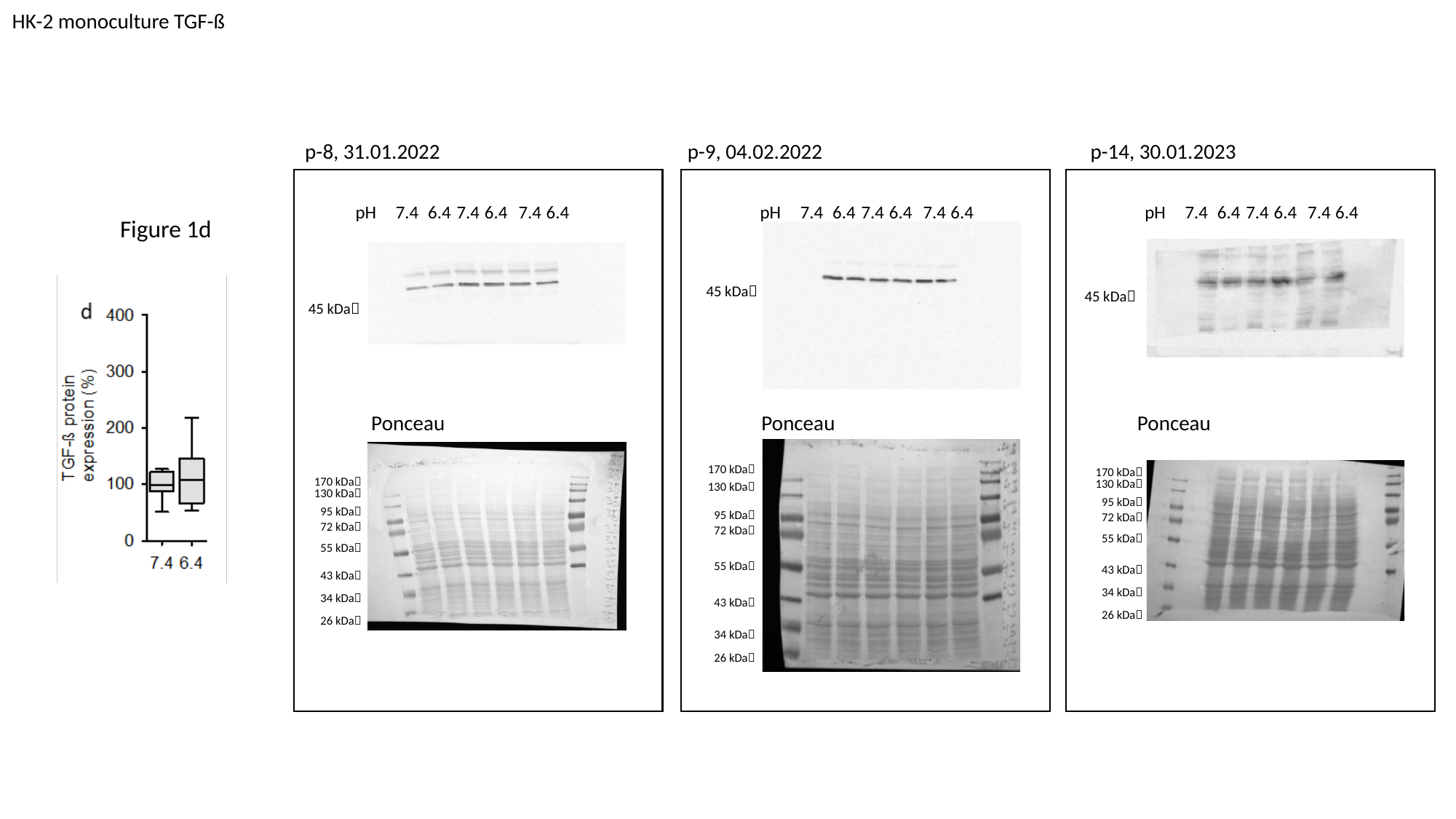

HK-2 monoculture TGF-ß
p-8, 31.01.2022
p-9, 04.02.2022
p-14, 30.01.2023
pH
7.4
6.4
7.4
6.4
7.4
6.4
pH
7.4
6.4
7.4
6.4
7.4
6.4
pH
7.4
6.4
7.4
6.4
7.4
6.4
Figure 1d
45 kDa
45 kDa
45 kDa
Ponceau
Ponceau
Ponceau
170 kDa
130 kDa
95 kDa
72 kDa
55 kDa
43 kDa
34 kDa
26 kDa
170 kDa
130 kDa
95 kDa
72 kDa
55 kDa
43 kDa
34 kDa
26 kDa
170 kDa
130 kDa
95 kDa
72 kDa
55 kDa
43 kDa
34 kDa
26 kDa

## Slide 8
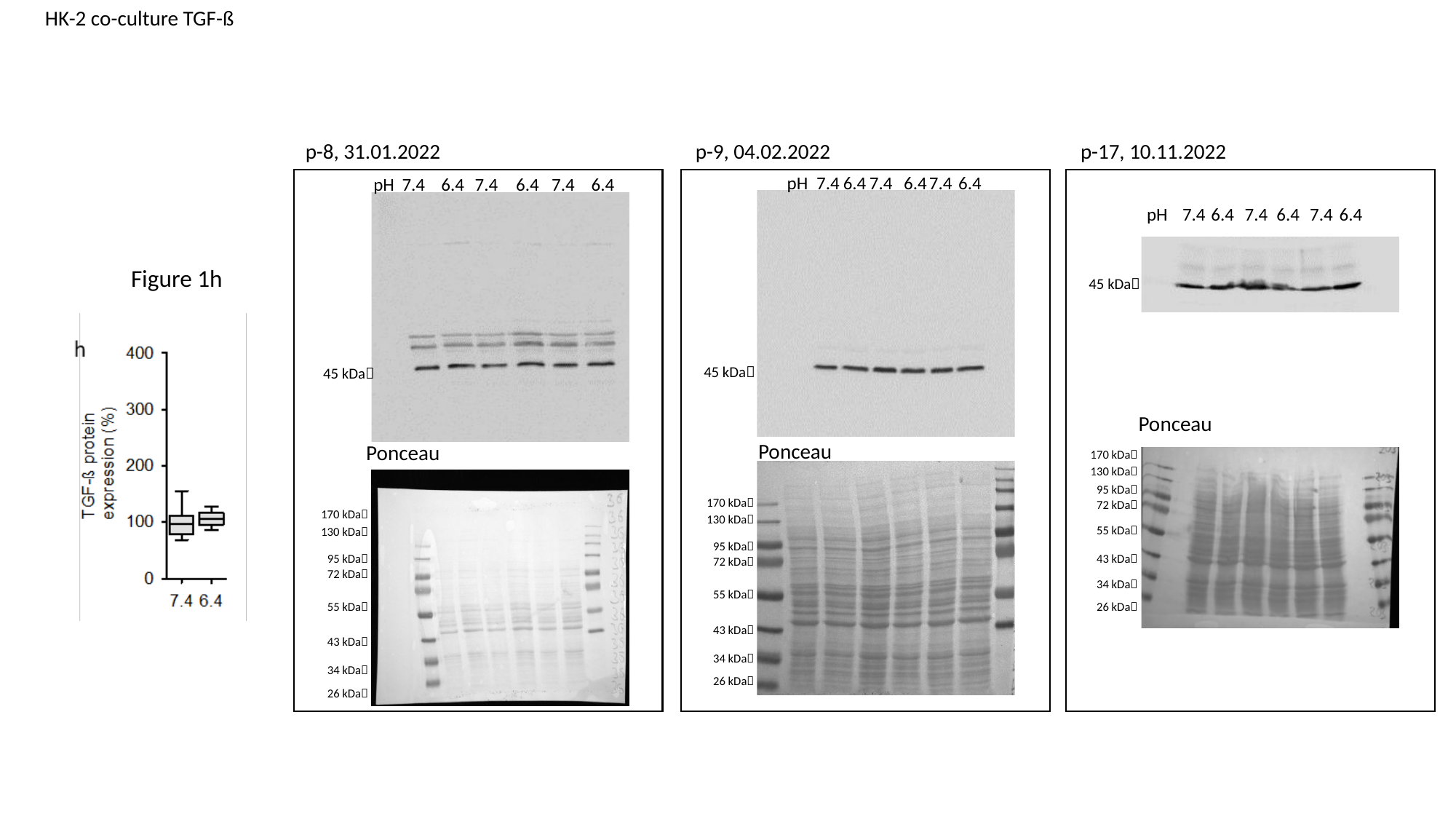

HK-2 co-culture TGF-ß
p-8, 31.01.2022
p-9, 04.02.2022
p-17, 10.11.2022
pH
7.4
6.4
7.4
6.4
7.4
6.4
pH
7.4
6.4
7.4
6.4
7.4
6.4
pH
7.4
6.4
7.4
6.4
7.4
6.4
Figure 1h
45 kDa
45 kDa
45 kDa
Ponceau
Ponceau
Ponceau
170 kDa
130 kDa
95 kDa
72 kDa
55 kDa
43 kDa
34 kDa
26 kDa
170 kDa
130 kDa
95 kDa
72 kDa
55 kDa
43 kDa
34 kDa
26 kDa
170 kDa
130 kDa
95 kDa
72 kDa
55 kDa
43 kDa
34 kDa
26 kDa

## Slide 9
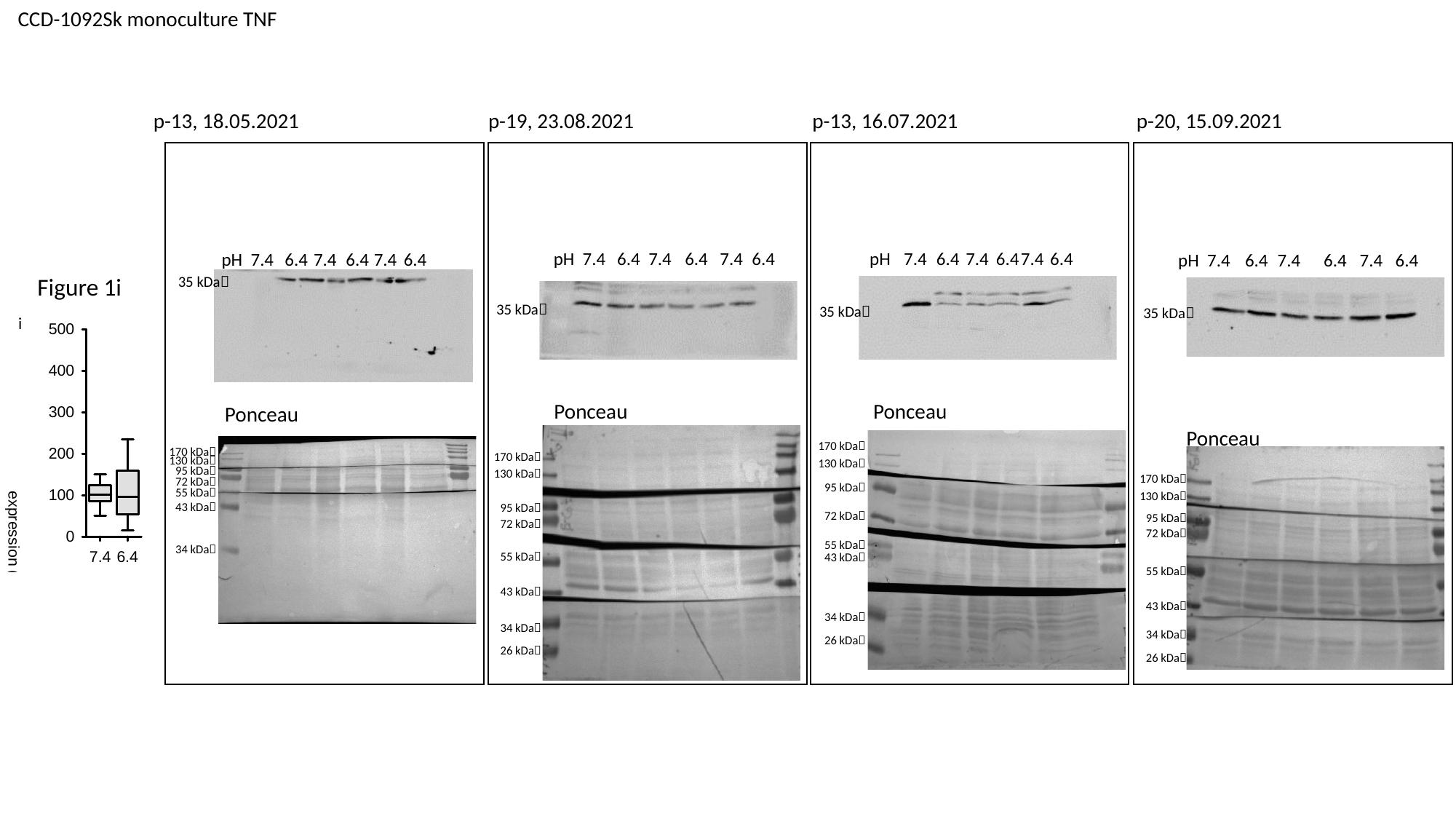

CCD-1092Sk monoculture TNF
p-13, 18.05.2021
p-19, 23.08.2021
p-13, 16.07.2021
p-20, 15.09.2021
pH
7.4
6.4
7.4
6.4
7.4
6.4
pH
7.4
6.4
7.4
6.4
7.4
6.4
pH
7.4
6.4
7.4
6.4
7.4
6.4
pH
7.4
6.4
7.4
6.4
7.4
6.4
Figure 1i
35 kDa
35 kDa
35 kDa
35 kDa
i
Ponceau
Ponceau
Ponceau
Ponceau
170 kDa
130 kDa
95 kDa
72 kDa
55 kDa
43 kDa
34 kDa
26 kDa
170 kDa
130 kDa
95 kDa
72 kDa
55 kDa
43 kDa
34 kDa
170 kDa
130 kDa
95 kDa
72 kDa
55 kDa
43 kDa
34 kDa
26 kDa
170 kDa
130 kDa
95 kDa
72 kDa
55 kDa
43 kDa
34 kDa
26 kDa

## Slide 10
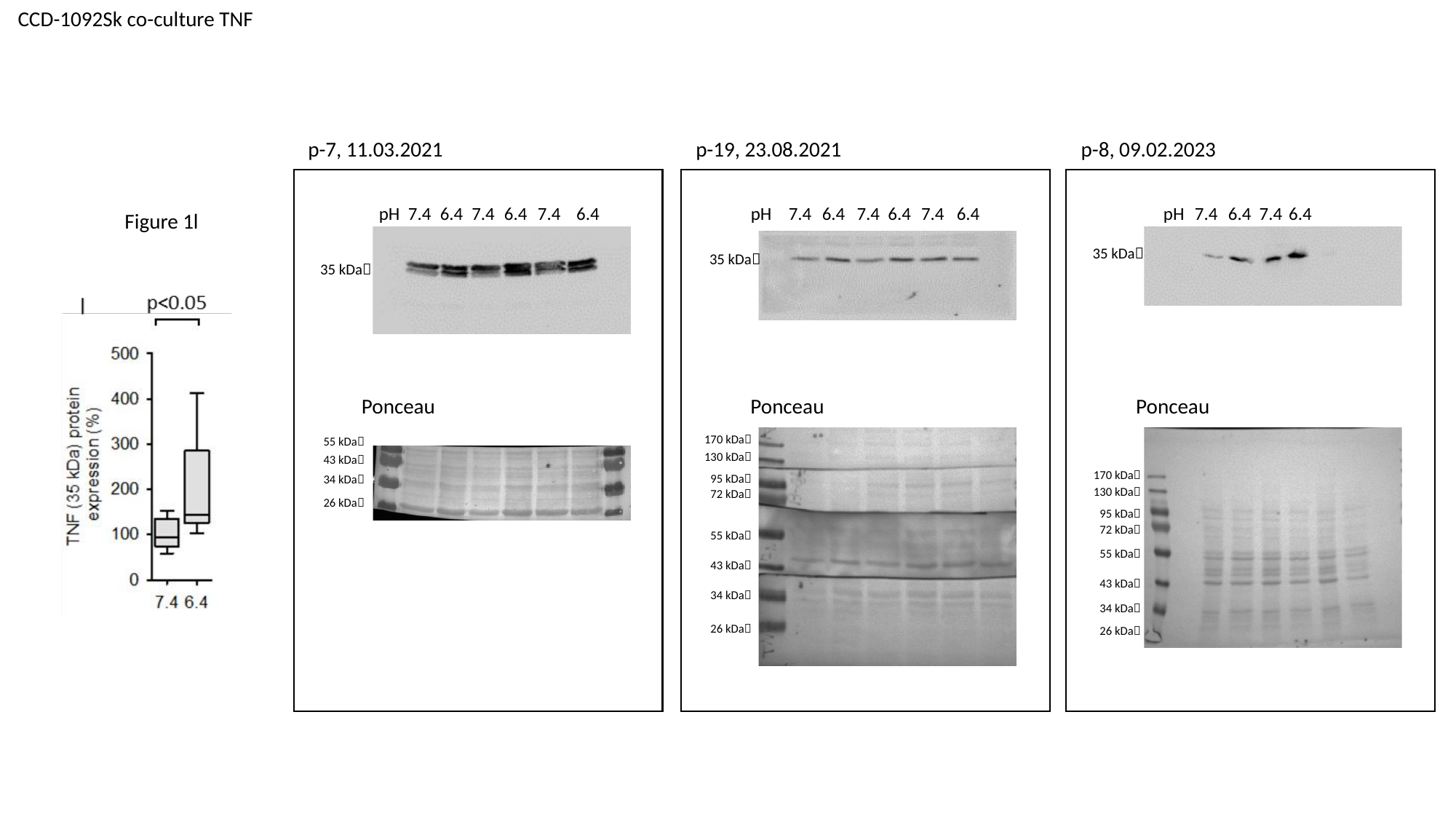

CCD-1092Sk co-culture TNF
p-7, 11.03.2021
p-19, 23.08.2021
p-8, 09.02.2023
pH
7.4
6.4
7.4
6.4
7.4
6.4
pH
7.4
6.4
7.4
6.4
pH
7.4
6.4
7.4
6.4
7.4
6.4
Figure 1l
35 kDa
35 kDa
35 kDa
Ponceau
Ponceau
Ponceau
170 kDa
130 kDa
95 kDa
72 kDa
55 kDa
43 kDa
34 kDa
26 kDa
55 kDa
43 kDa
34 kDa
26 kDa
170 kDa
130 kDa
95 kDa
72 kDa
55 kDa
43 kDa
34 kDa
26 kDa

## Slide 11
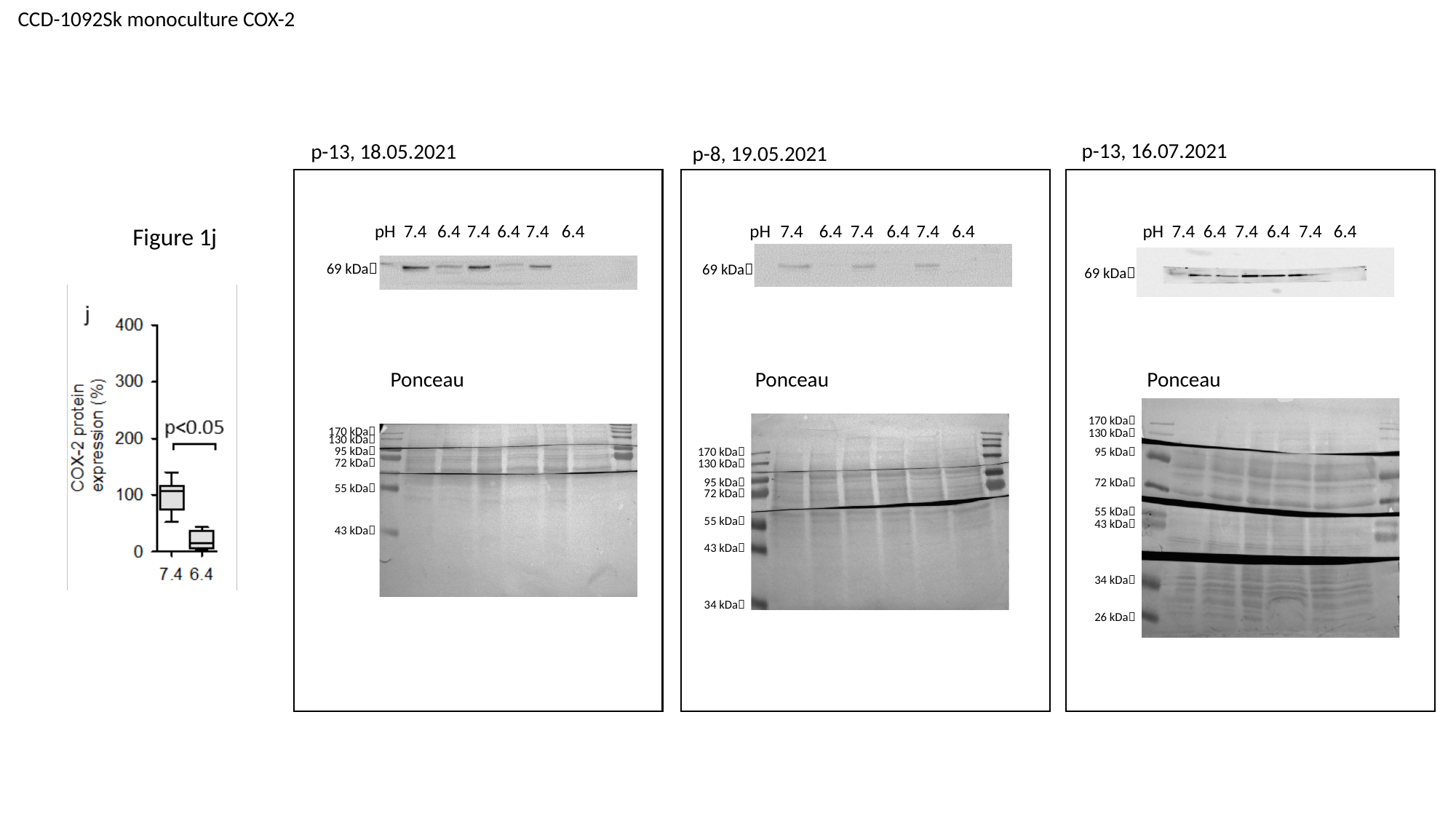

CCD-1092Sk monoculture COX-2
p-13, 16.07.2021
p-13, 18.05.2021
p-8, 19.05.2021
pH
7.4
6.4
7.4
6.4
7.4
6.4
pH
7.4
6.4
7.4
7.4
6.4
6.4
pH
7.4
6.4
7.4
6.4
7.4
6.4
Figure 1j
69 kDa
69 kDa
69 kDa
Ponceau
Ponceau
Ponceau
170 kDa
130 kDa
95 kDa
72 kDa
55 kDa
43 kDa
34 kDa
26 kDa
170 kDa
130 kDa
95 kDa
72 kDa
55 kDa
43 kDa
170 kDa
130 kDa
95 kDa
72 kDa
55 kDa
43 kDa
34 kDa

## Slide 12
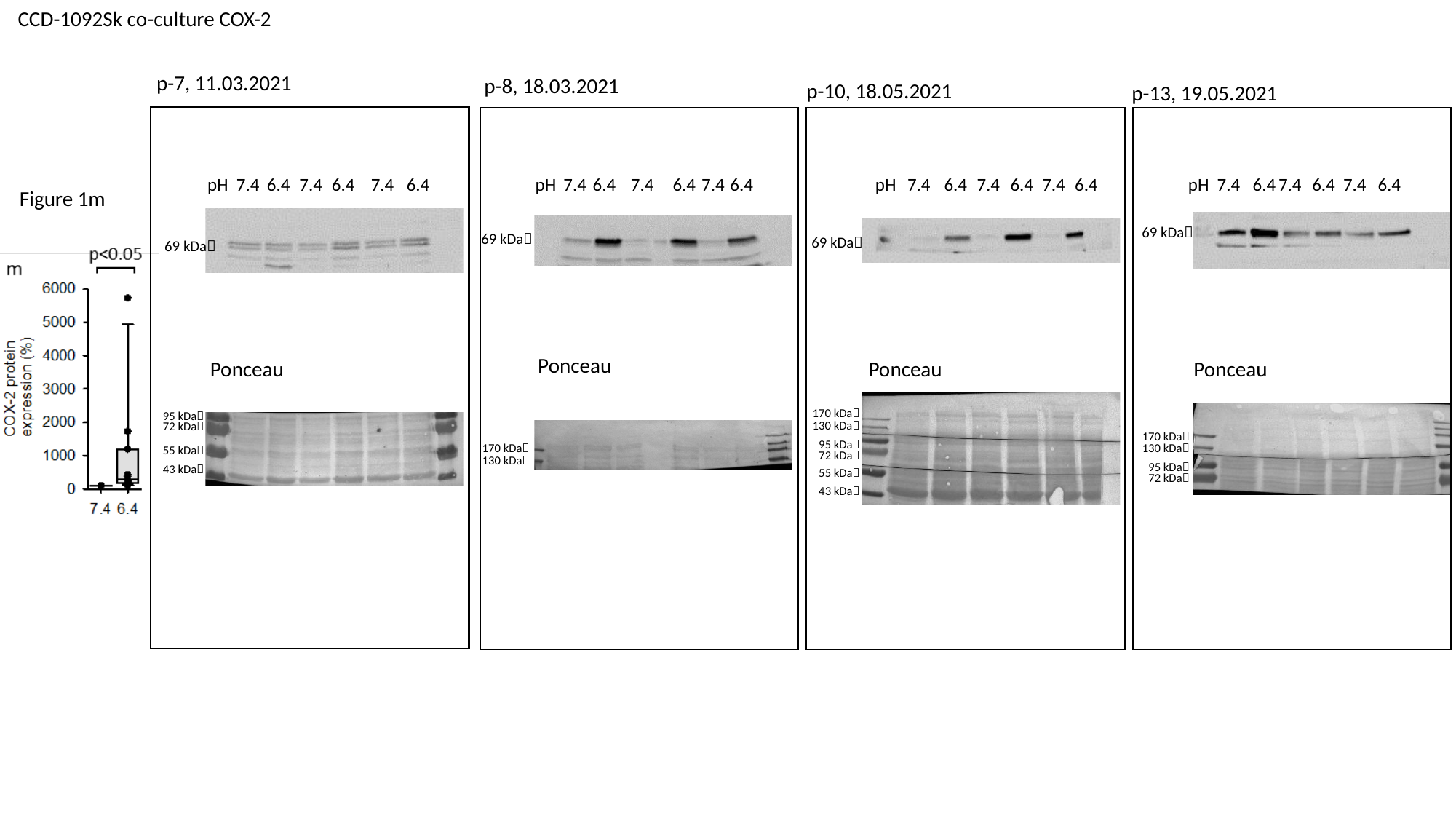

CCD-1092Sk co-culture COX-2
p-7, 11.03.2021
p-8, 18.03.2021
p-10, 18.05.2021
p-13, 19.05.2021
pH
7.4
6.4
7.4
6.4
7.4
6.4
pH
7.4
6.4
7.4
6.4
7.4
6.4
pH
7.4
6.4
7.4
6.4
7.4
6.4
pH
7.4
6.4
7.4
6.4
7.4
6.4
Figure 1m
69 kDa
69 kDa
69 kDa
69 kDa
Ponceau
Ponceau
Ponceau
Ponceau
170 kDa
130 kDa
95 kDa
72 kDa
55 kDa
43 kDa
95 kDa
72 kDa
55 kDa
43 kDa
170 kDa
130 kDa
95 kDa
72 kDa
170 kDa
130 kDa

## Slide 13
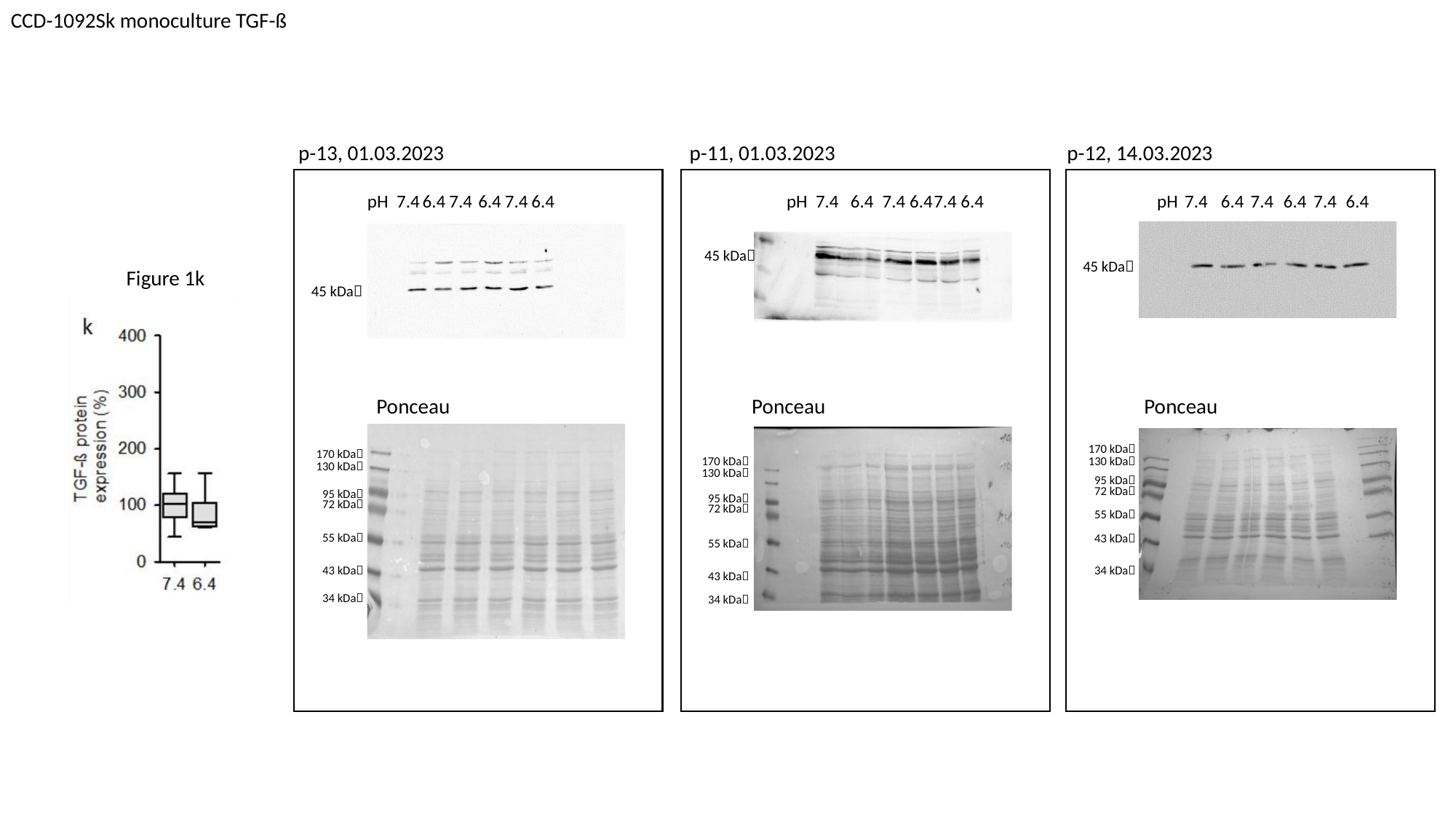

CCD-1092Sk monoculture TGF-ß
p-13, 01.03.2023
p-11, 01.03.2023
p-12, 14.03.2023
pH
7.4
6.4
7.4
6.4
7.4
6.4
pH
7.4
6.4
7.4
6.4
7.4
6.4
pH
7.4
6.4
7.4
6.4
7.4
6.4
45 kDa
45 kDa
Figure 1k
45 kDa
Ponceau
Ponceau
Ponceau
170 kDa
130 kDa
95 kDa
72 kDa
55 kDa
43 kDa
34 kDa
170 kDa
130 kDa
95 kDa
72 kDa
55 kDa
43 kDa
34 kDa
170 kDa
130 kDa
95 kDa
72 kDa
55 kDa
43 kDa
34 kDa

## Slide 14
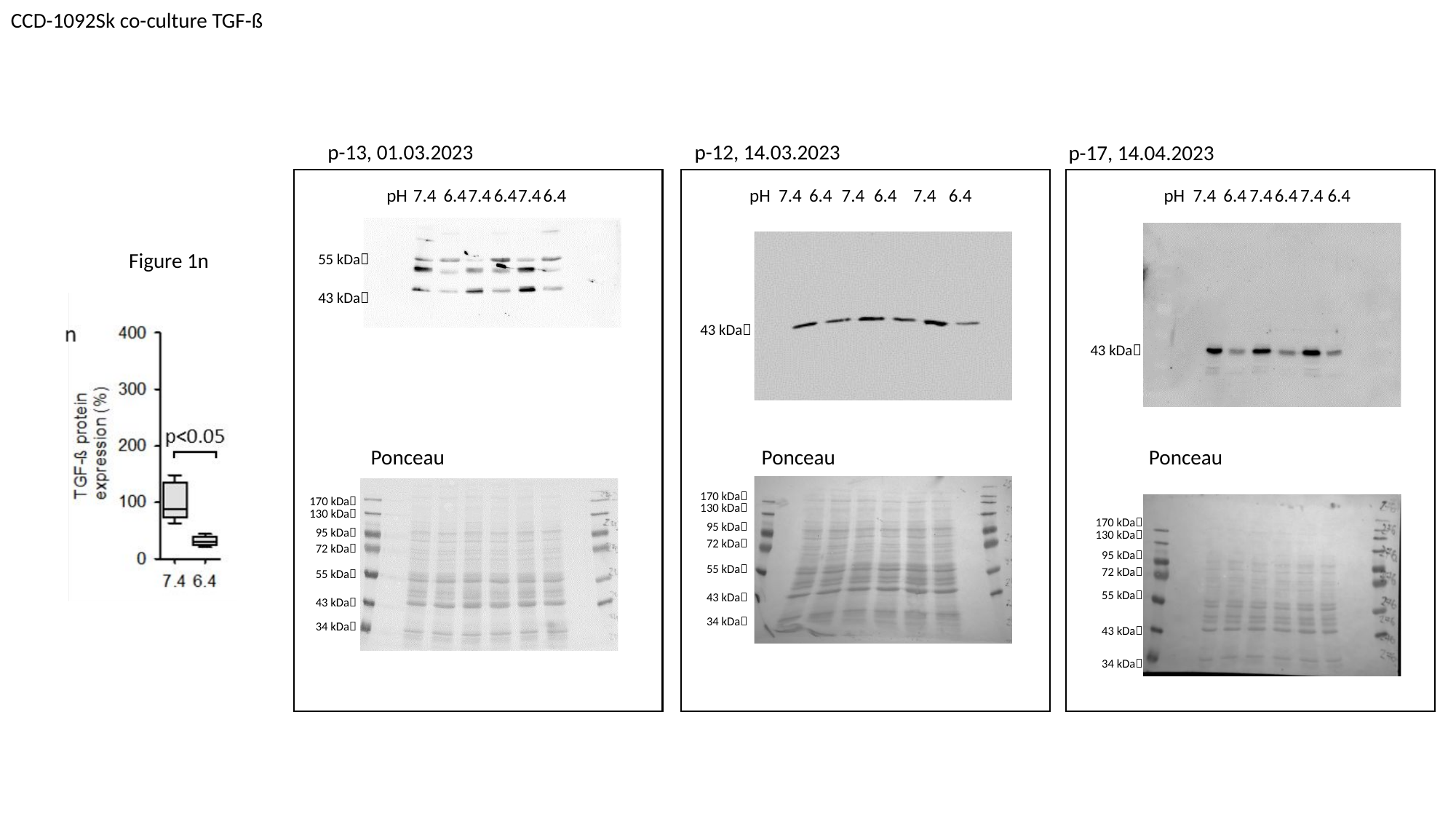

CCD-1092Sk co-culture TGF-ß
p-12, 14.03.2023
p-13, 01.03.2023
p-17, 14.04.2023
pH
7.4
6.4
7.4
6.4
7.4
6.4
pH
7.4
6.4
7.4
6.4
7.4
6.4
pH
7.4
6.4
7.4
6.4
7.4
6.4
Figure 1n
55 kDa
43 kDa
43 kDa
43 kDa
Ponceau
Ponceau
Ponceau
170 kDa
130 kDa
95 kDa
72 kDa
55 kDa
43 kDa
34 kDa
170 kDa
130 kDa
95 kDa
72 kDa
55 kDa
43 kDa
34 kDa
170 kDa
130 kDa
95 kDa
72 kDa
55 kDa
43 kDa
34 kDa

## Slide 15
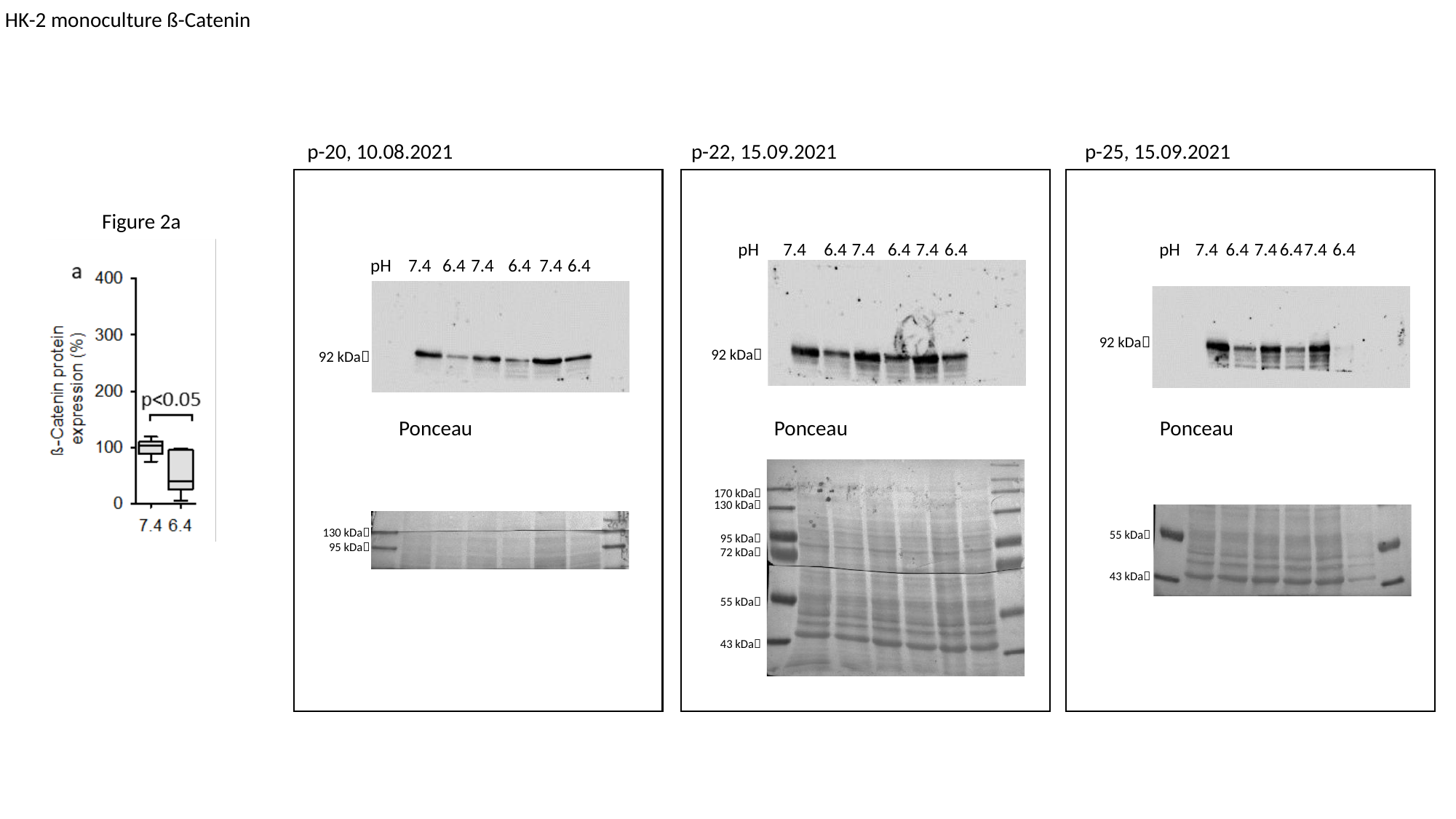

HK-2 monoculture ß-Catenin
p-20, 10.08.2021
p-22, 15.09.2021
p-25, 15.09.2021
Figure 2a
pH
7.4
6.4
7.4
6.4
7.4
6.4
pH
7.4
6.4
7.4
6.4
7.4
6.4
pH
7.4
6.4
7.4
6.4
7.4
6.4
92 kDa
92 kDa
92 kDa
Ponceau
Ponceau
Ponceau
170 kDa
130 kDa
95 kDa
72 kDa
55 kDa
43 kDa
130 kDa
95 kDa
55 kDa
43 kDa

## Slide 16
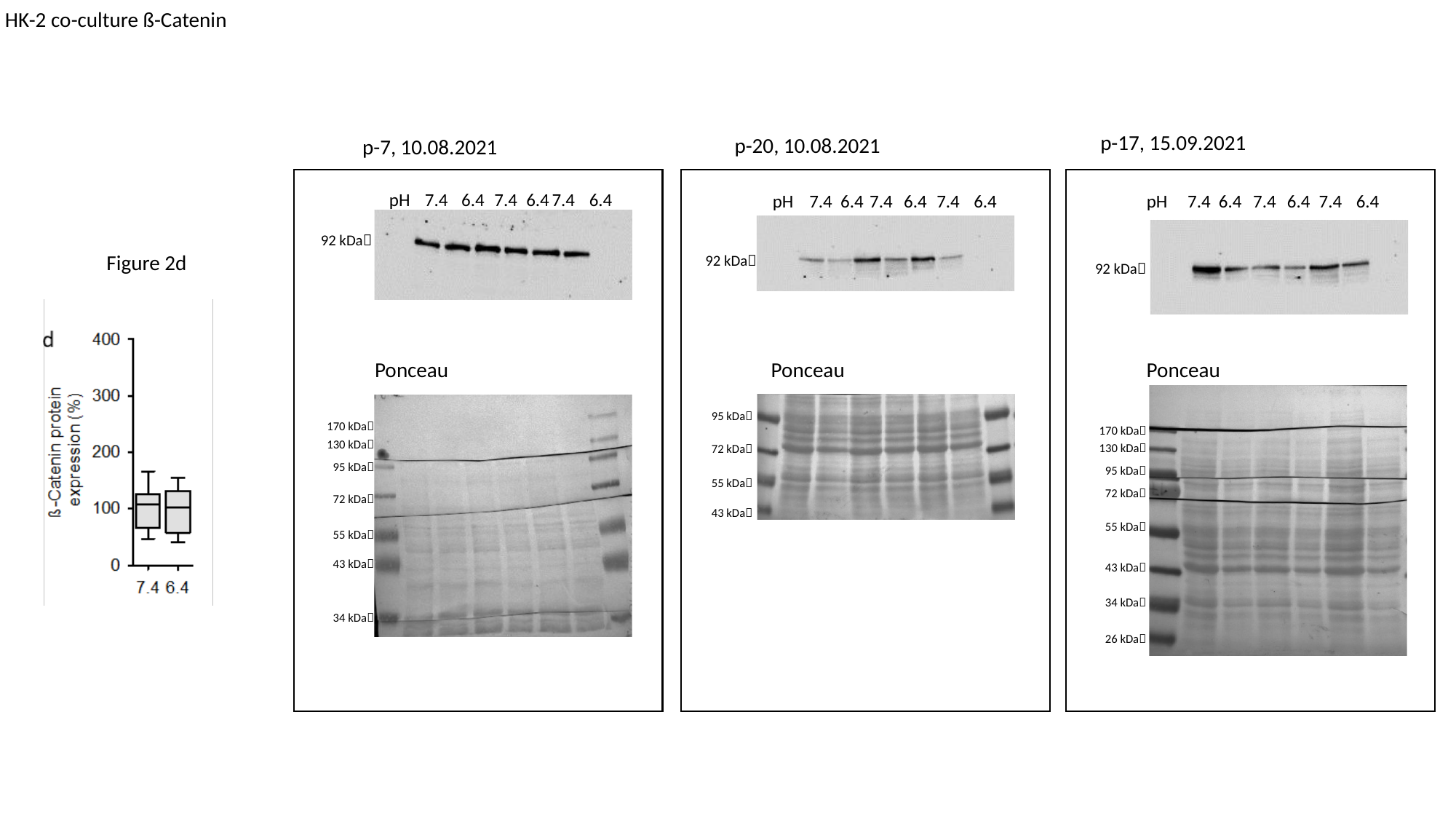

HK-2 co-culture ß-Catenin
p-17, 15.09.2021
p-20, 10.08.2021
p-7, 10.08.2021
pH
7.4
6.4
7.4
6.4
7.4
6.4
pH
7.4
6.4
7.4
6.4
7.4
6.4
pH
7.4
6.4
7.4
6.4
7.4
6.4
92 kDa
Figure 2d
92 kDa
92 kDa
Ponceau
Ponceau
Ponceau
95 kDa
72 kDa
55 kDa
43 kDa
170 kDa
130 kDa
95 kDa
72 kDa
55 kDa
43 kDa
34 kDa
170 kDa
130 kDa
95 kDa
72 kDa
55 kDa
43 kDa
34 kDa
26 kDa

## Slide 17
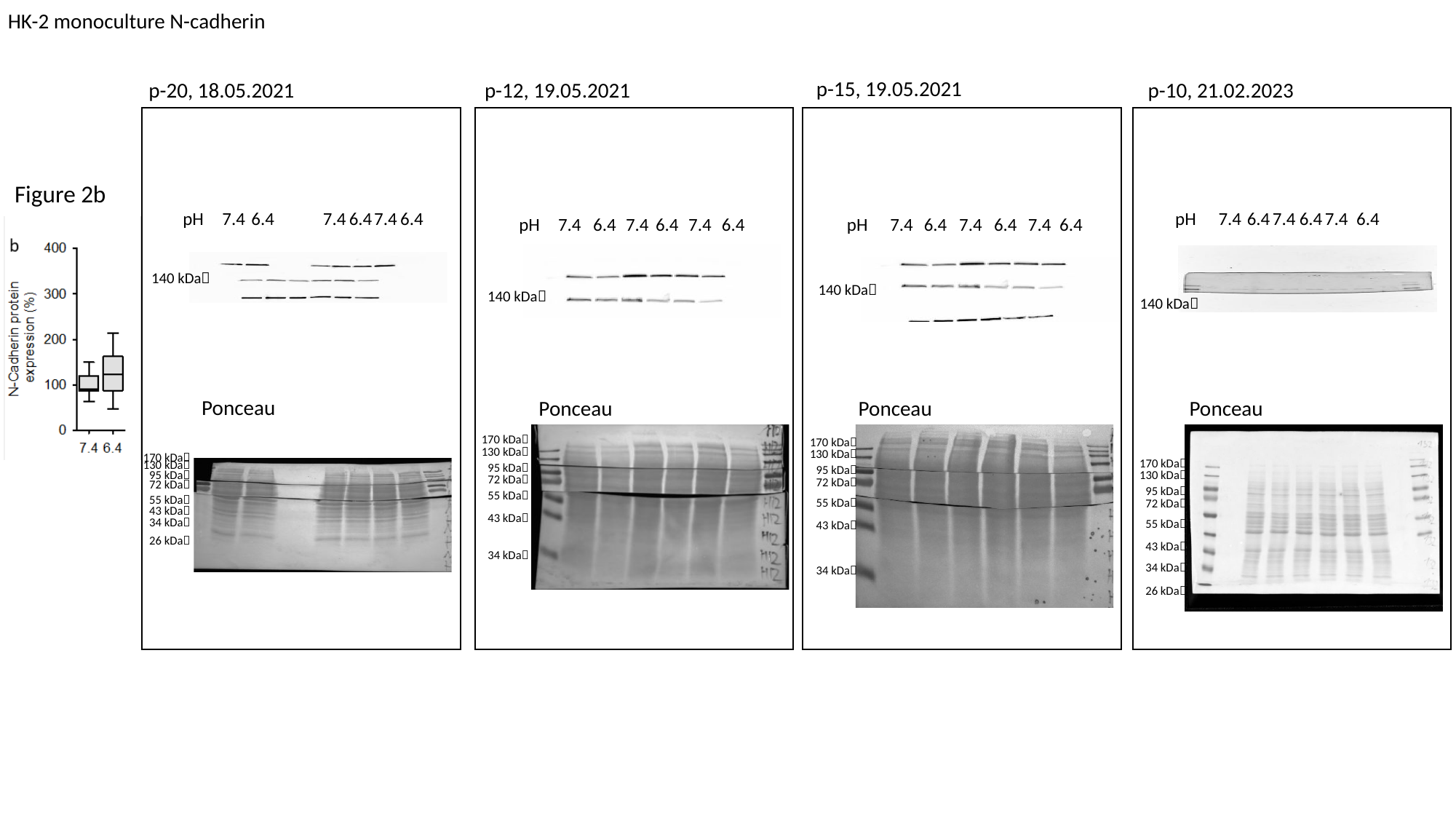

HK-2 monoculture N-cadherin
p-15, 19.05.2021
p-20, 18.05.2021
p-12, 19.05.2021
p-10, 21.02.2023
Figure 2b
pH
7.4
6.4
7.4
6.4
7.4
6.4
pH
7.4
6.4
7.4
6.4
7.4
6.4
pH
7.4
6.4
7.4
6.4
7.4
6.4
pH
7.4
6.4
7.4
6.4
7.4
6.4
140 kDa
140 kDa
140 kDa
140 kDa
Ponceau
Ponceau
Ponceau
Ponceau
170 kDa
130 kDa
95 kDa
72 kDa
55 kDa
43 kDa
34 kDa
170 kDa
130 kDa
95 kDa
72 kDa
55 kDa
43 kDa
34 kDa
170 kDa
130 kDa
95 kDa
72 kDa
55 kDa
43 kDa
34 kDa
26 kDa
170 kDa
130 kDa
95 kDa
72 kDa
55 kDa
43 kDa
34 kDa
26 kDa

## Slide 18
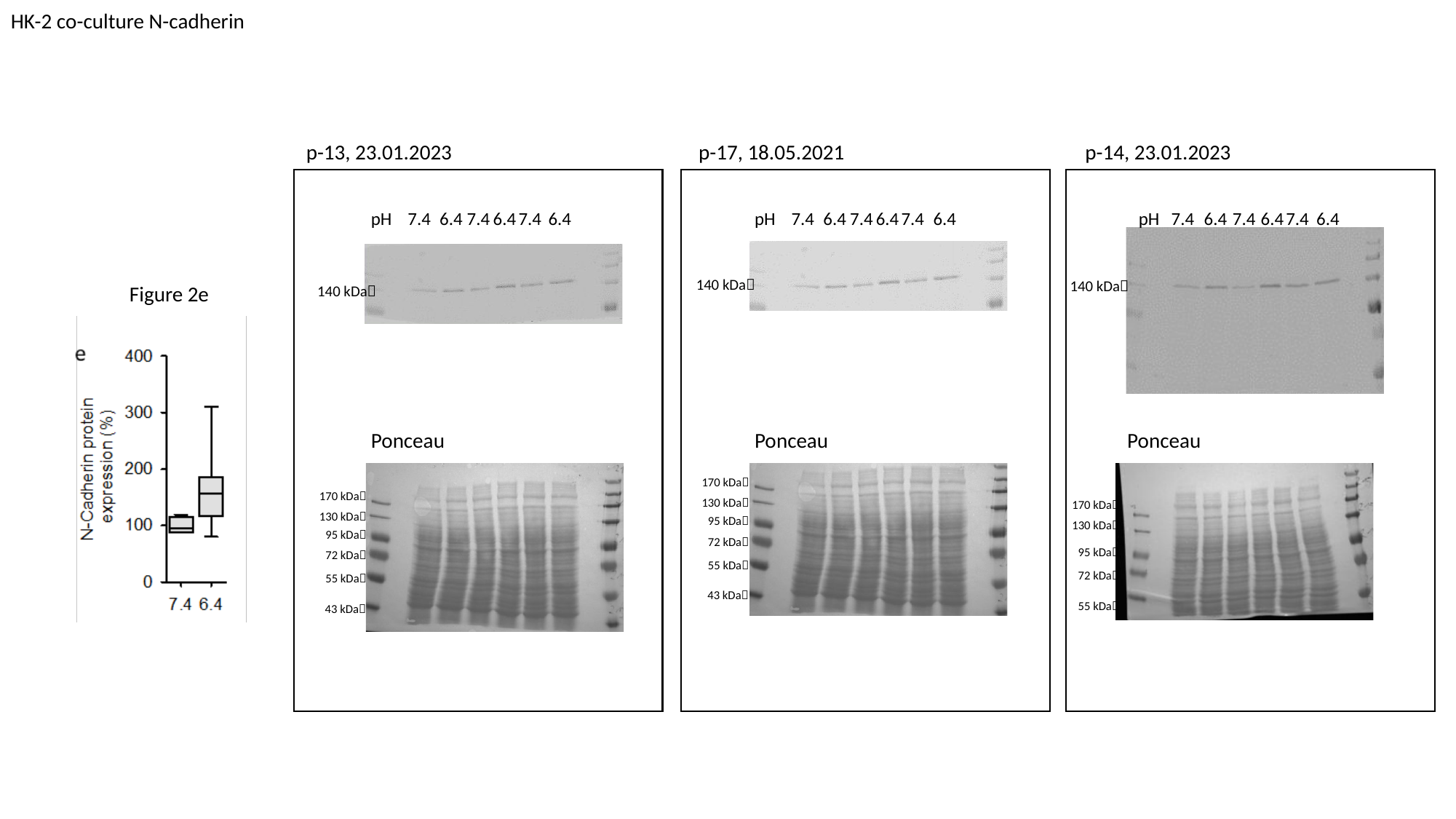

HK-2 co-culture N-cadherin
p-13, 23.01.2023
p-17, 18.05.2021
p-14, 23.01.2023
pH
7.4
6.4
7.4
6.4
7.4
6.4
pH
7.4
6.4
7.4
6.4
7.4
6.4
pH
7.4
6.4
7.4
6.4
7.4
6.4
140 kDa
140 kDa
140 kDa
Figure 2e
Ponceau
Ponceau
Ponceau
170 kDa
130 kDa
95 kDa
72 kDa
55 kDa
43 kDa
170 kDa
130 kDa
95 kDa
72 kDa
55 kDa
43 kDa
170 kDa
130 kDa
95 kDa
72 kDa
55 kDa

## Slide 19
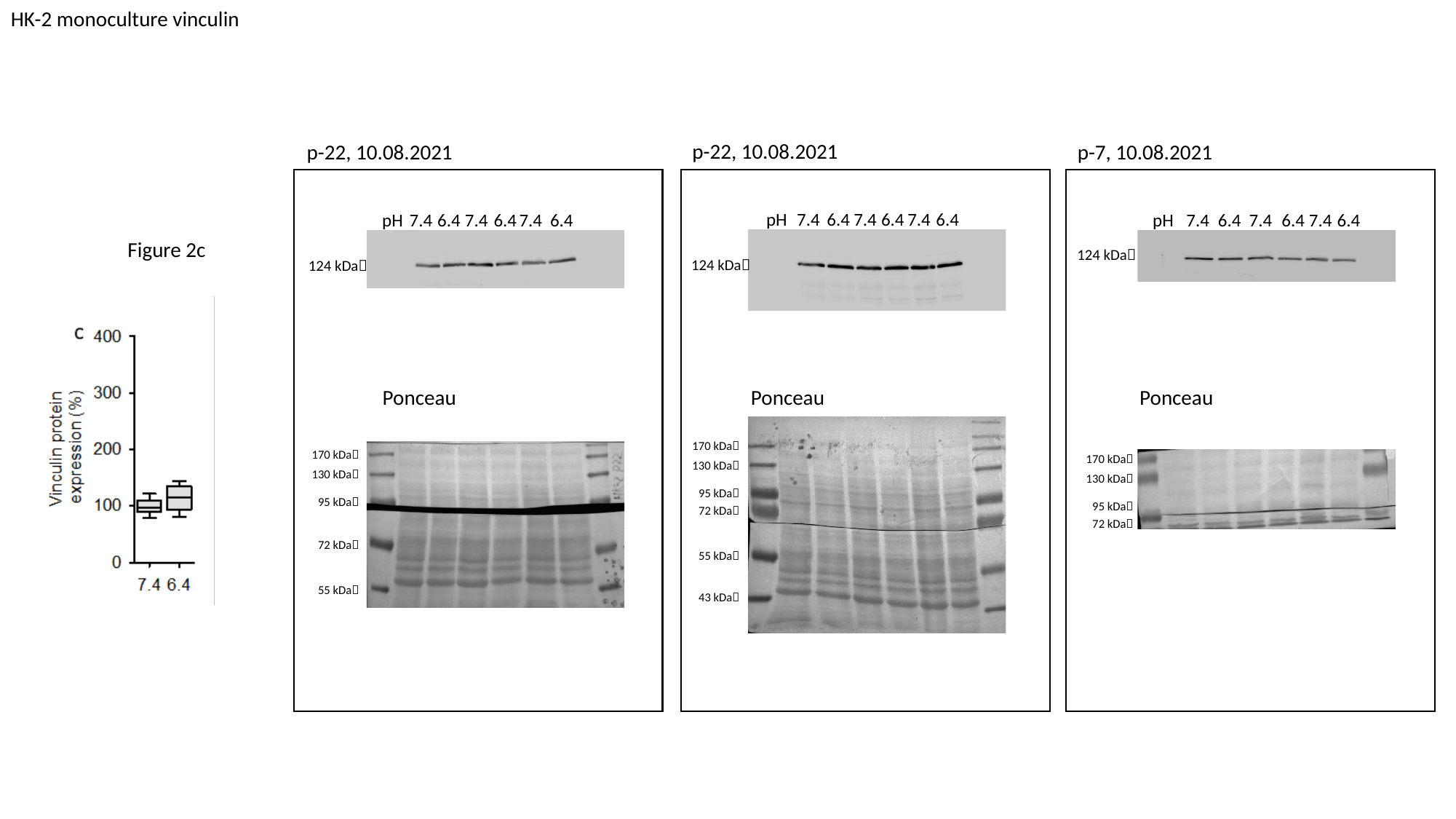

HK-2 monoculture vinculin
p-22, 10.08.2021
p-22, 10.08.2021
p-7, 10.08.2021
pH
7.4
6.4
7.4
6.4
7.4
6.4
pH
7.4
6.4
7.4
6.4
7.4
6.4
pH
7.4
6.4
7.4
6.4
7.4
6.4
Figure 2c
124 kDa
124 kDa
124 kDa
Ponceau
Ponceau
Ponceau
170 kDa
130 kDa
95 kDa
72 kDa
55 kDa
43 kDa
170 kDa
130 kDa
95 kDa
72 kDa
55 kDa
170 kDa
130 kDa
95 kDa
72 kDa

## Slide 20
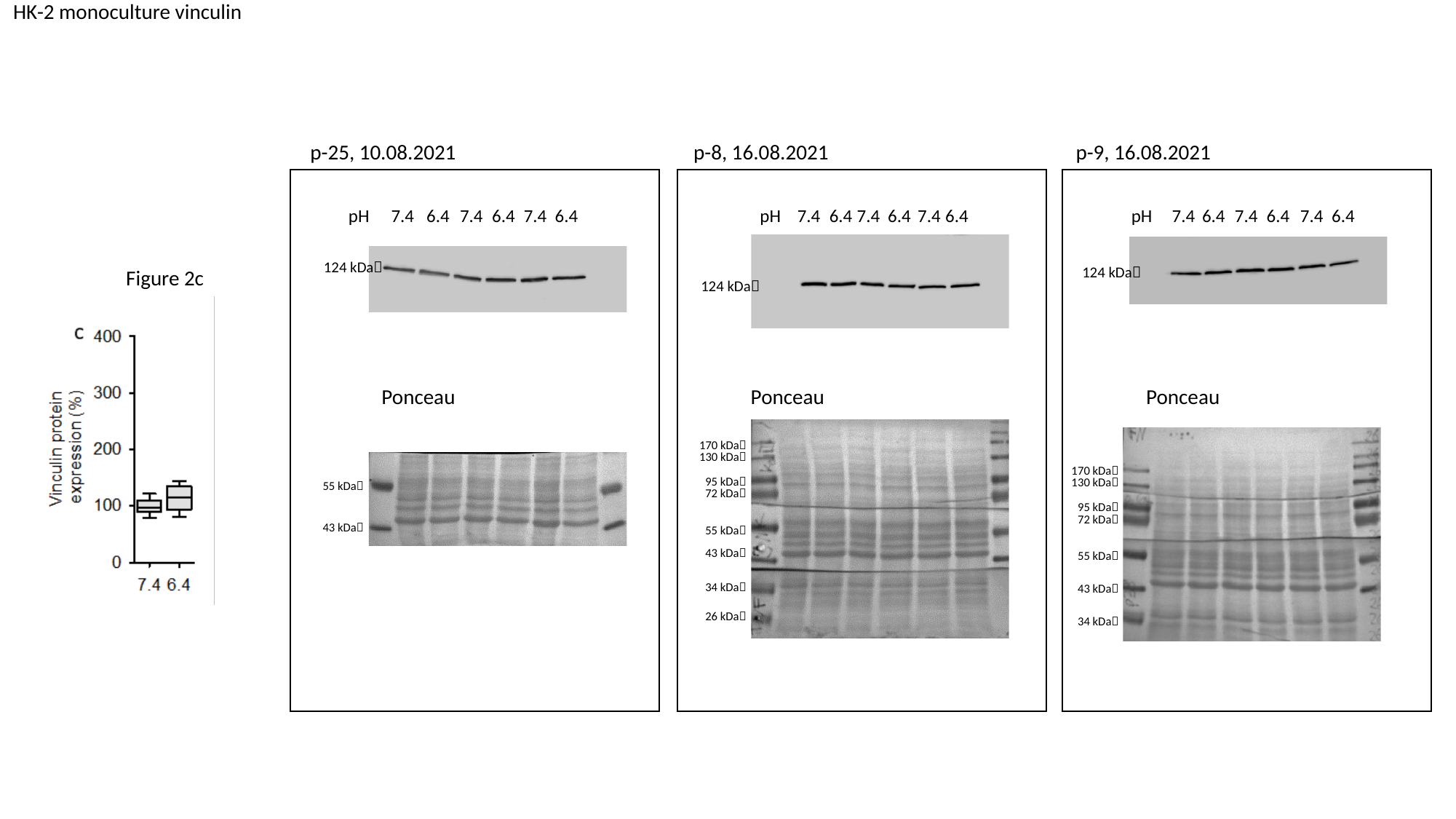

HK-2 monoculture vinculin
p-25, 10.08.2021
p-8, 16.08.2021
p-9, 16.08.2021
pH
7.4
6.4
7.4
6.4
7.4
6.4
pH
7.4
6.4
7.4
6.4
7.4
6.4
pH
7.4
6.4
7.4
6.4
7.4
6.4
124 kDa
124 kDa
Figure 2c
124 kDa
Ponceau
Ponceau
Ponceau
170 kDa
130 kDa
95 kDa
72 kDa
55 kDa
43 kDa
34 kDa
26 kDa
170 kDa
130 kDa
95 kDa
72 kDa
55 kDa
43 kDa
34 kDa
55 kDa
43 kDa

## Slide 21
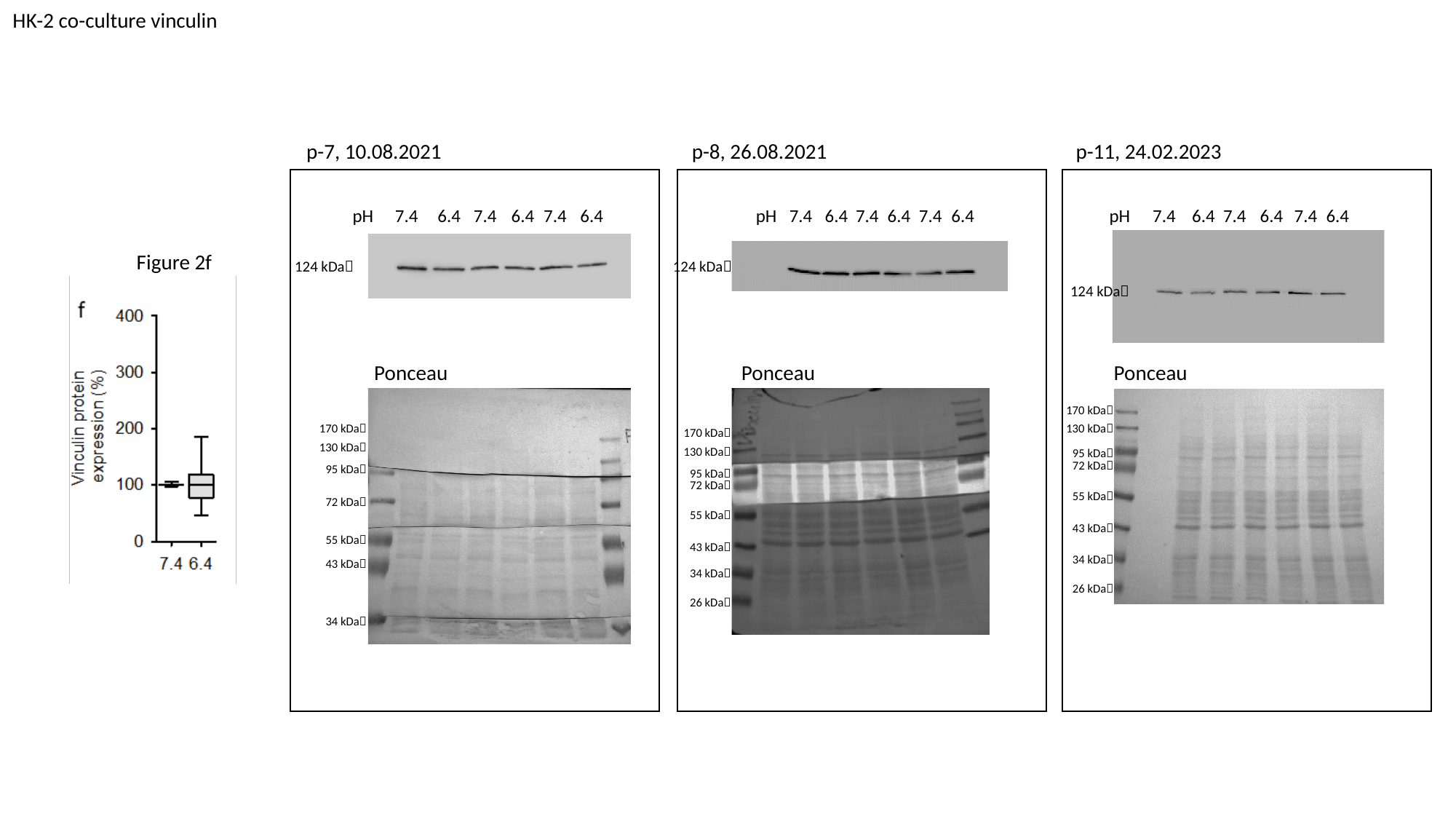

HK-2 co-culture vinculin
p-7, 10.08.2021
p-8, 26.08.2021
p-11, 24.02.2023
pH
7.4
6.4
7.4
6.4
7.4
6.4
pH
7.4
6.4
7.4
6.4
7.4
6.4
pH
7.4
6.4
7.4
6.4
7.4
6.4
Figure 2f
124 kDa
124 kDa
124 kDa
Ponceau
Ponceau
Ponceau
170 kDa
130 kDa
95 kDa
72 kDa
55 kDa
43 kDa
34 kDa
26 kDa
170 kDa
130 kDa
95 kDa
72 kDa
55 kDa
43 kDa
34 kDa
170 kDa
130 kDa
95 kDa
72 kDa
55 kDa
43 kDa
34 kDa
26 kDa

## Slide 22
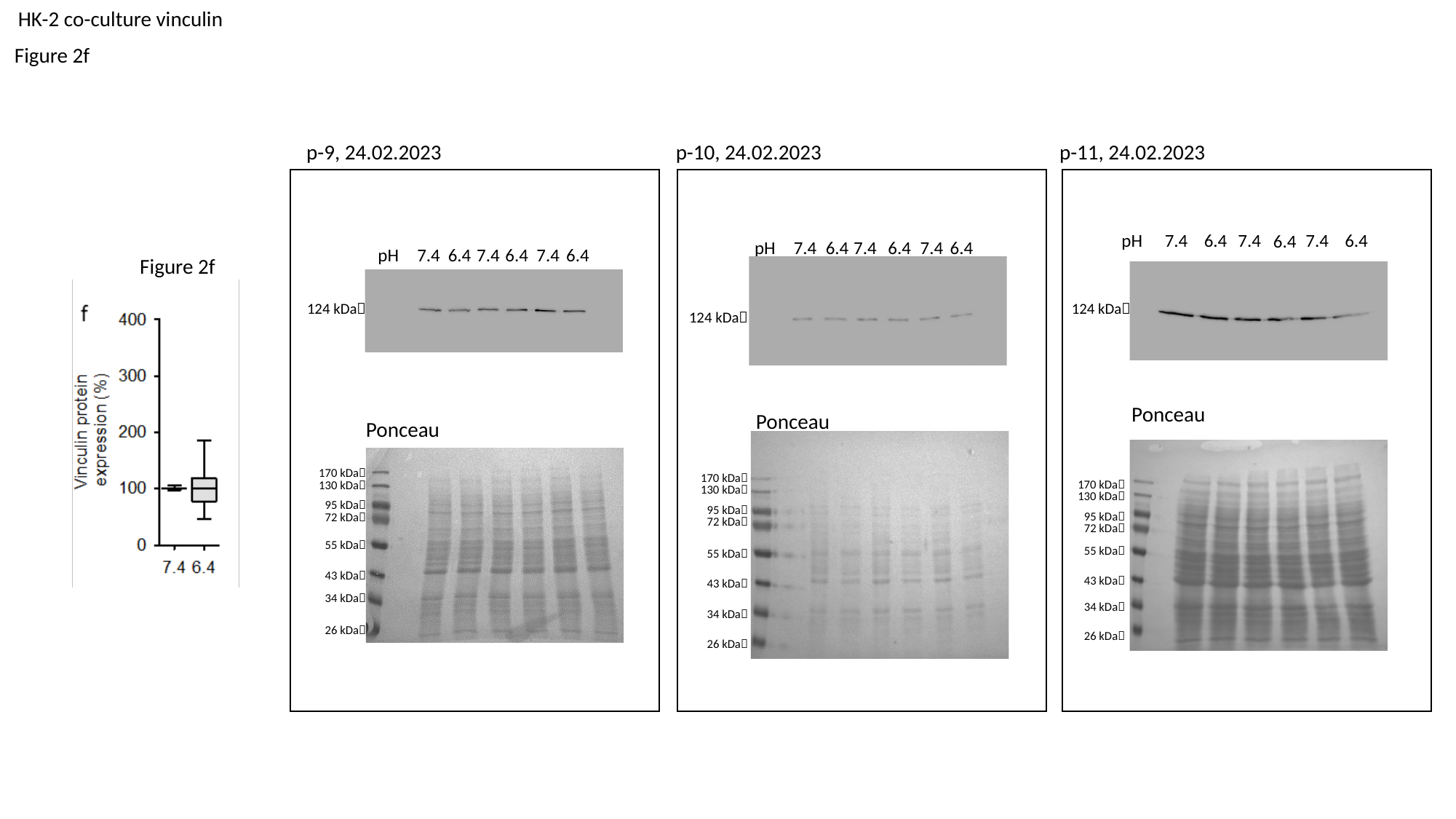

HK-2 co-culture vinculin
Figure 2f
p-9, 24.02.2023
p-10, 24.02.2023
p-11, 24.02.2023
pH
7.4
6.4
7.4
7.4
6.4
6.4
pH
7.4
6.4
7.4
6.4
7.4
6.4
pH
7.4
6.4
7.4
6.4
7.4
6.4
Figure 2f
124 kDa
124 kDa
124 kDa
Ponceau
Ponceau
Ponceau
170 kDa
130 kDa
95 kDa
72 kDa
55 kDa
43 kDa
34 kDa
26 kDa
170 kDa
130 kDa
95 kDa
72 kDa
55 kDa
43 kDa
34 kDa
26 kDa
170 kDa
130 kDa
95 kDa
72 kDa
55 kDa
43 kDa
34 kDa
26 kDa

## Slide 23
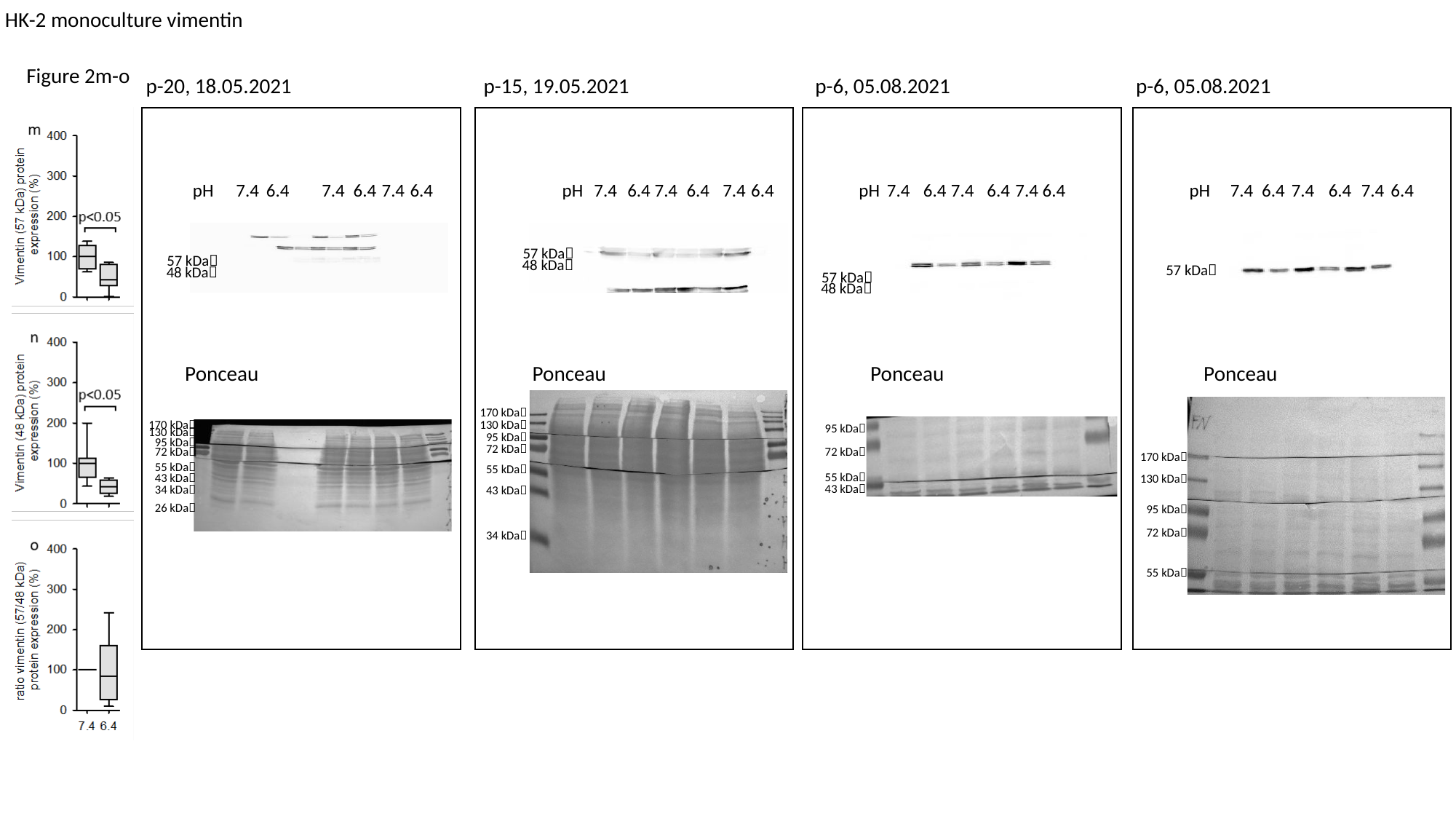

HK-2 monoculture vimentin
Figure 2m-o
p-20, 18.05.2021
p-15, 19.05.2021
p-6, 05.08.2021
p-6, 05.08.2021
pH
7.4
6.4
7.4
6.4
7.4
6.4
pH
7.4
6.4
7.4
6.4
7.4
6.4
pH
7.4
6.4
7.4
6.4
7.4
6.4
pH
7.4
6.4
7.4
6.4
7.4
6.4
57 kDa
57 kDa
48 kDa
57 kDa
48 kDa
57 kDa
48 kDa
Ponceau
Ponceau
Ponceau
Ponceau
170 kDa
130 kDa
95 kDa
72 kDa
55 kDa
43 kDa
34 kDa
170 kDa
130 kDa
95 kDa
72 kDa
55 kDa
43 kDa
34 kDa
26 kDa
95 kDa
72 kDa
55 kDa
170 kDa
130 kDa
95 kDa
72 kDa
55 kDa
43 kDa

## Slide 24
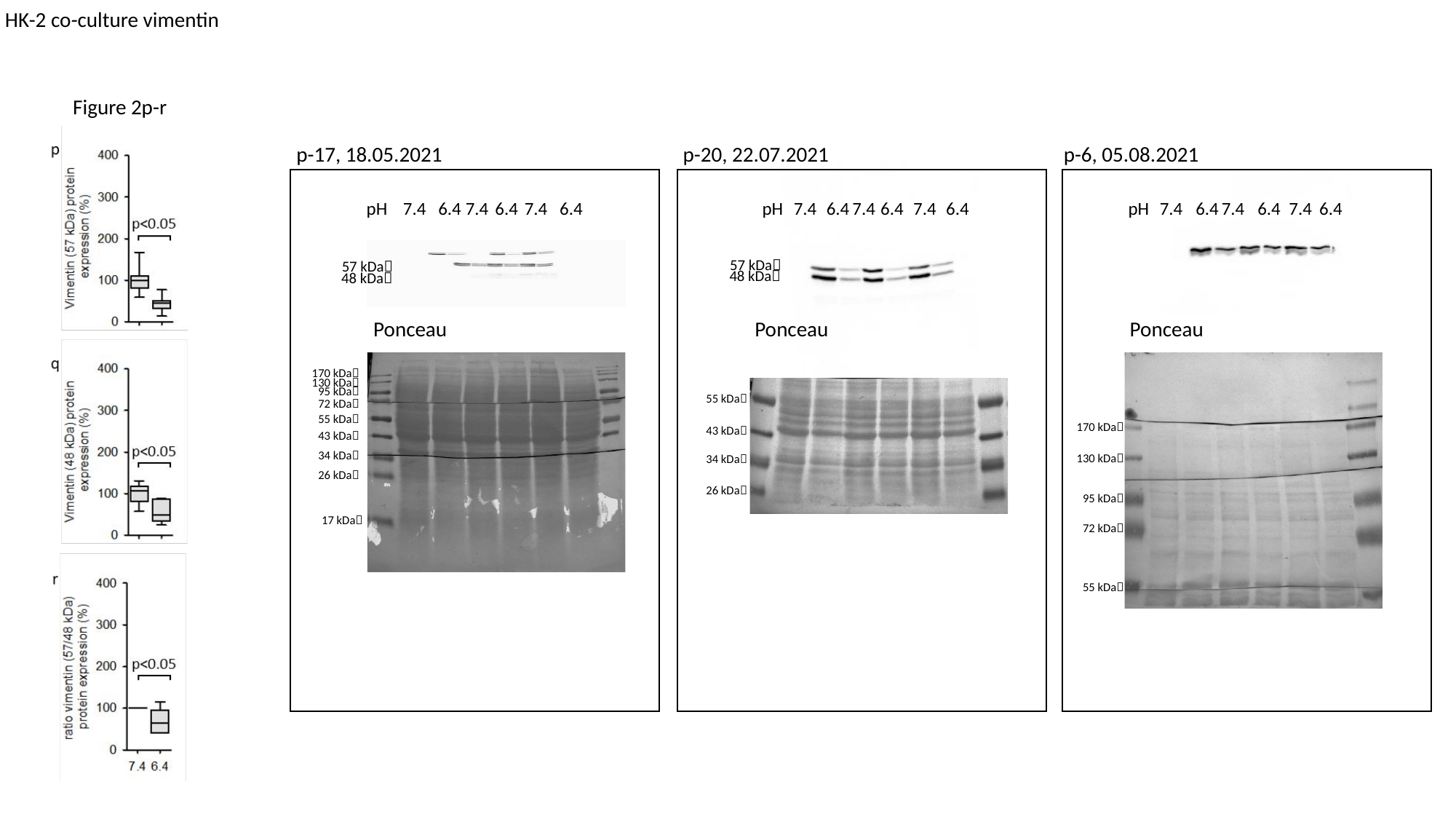

HK-2 co-culture vimentin
Figure 2p-r
p-17, 18.05.2021
p-20, 22.07.2021
p-6, 05.08.2021
pH
7.4
6.4
7.4
6.4
7.4
6.4
pH
7.4
6.4
7.4
6.4
7.4
6.4
pH
7.4
6.4
7.4
6.4
7.4
6.4
57 kDa
57 kDa
48 kDa
48 kDa
Ponceau
Ponceau
Ponceau
170 kDa
130 kDa
95 kDa
72 kDa
55 kDa
43 kDa
34 kDa
26 kDa
17 kDa
55 kDa
43 kDa
34 kDa
26 kDa
170 kDa
130 kDa
95 kDa
72 kDa
55 kDa

## Slide 25
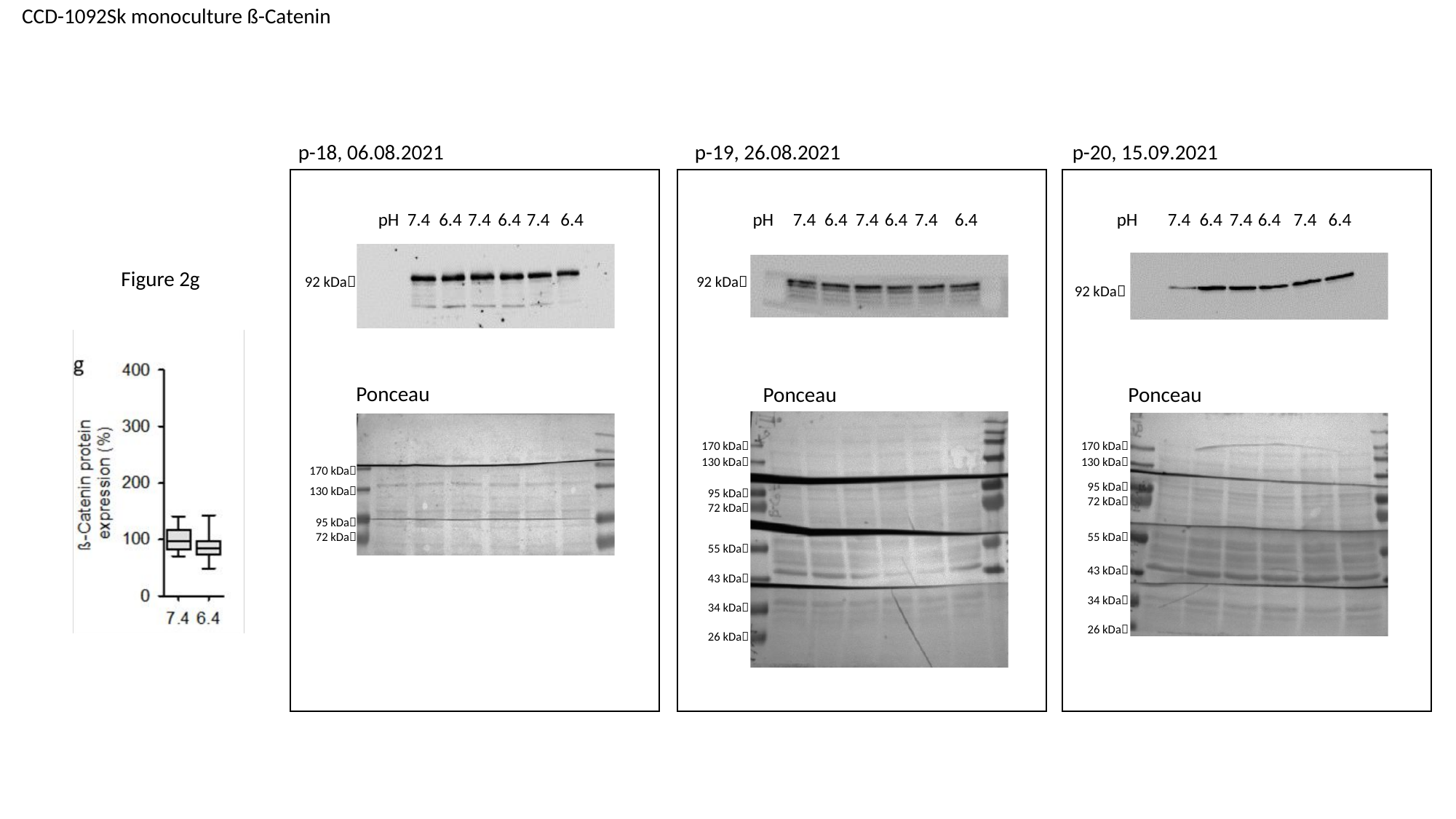

CCD-1092Sk monoculture ß-Catenin
p-18, 06.08.2021
p-19, 26.08.2021
p-20, 15.09.2021
pH
7.4
6.4
7.4
6.4
7.4
6.4
pH
7.4
6.4
7.4
6.4
7.4
6.4
pH
7.4
6.4
7.4
6.4
7.4
6.4
Figure 2g
92 kDa
92 kDa
92 kDa
Ponceau
Ponceau
Ponceau
170 kDa
130 kDa
95 kDa
72 kDa
55 kDa
43 kDa
34 kDa
26 kDa
170 kDa
130 kDa
95 kDa
72 kDa
55 kDa
43 kDa
34 kDa
26 kDa
170 kDa
130 kDa
95 kDa
72 kDa

## Slide 26
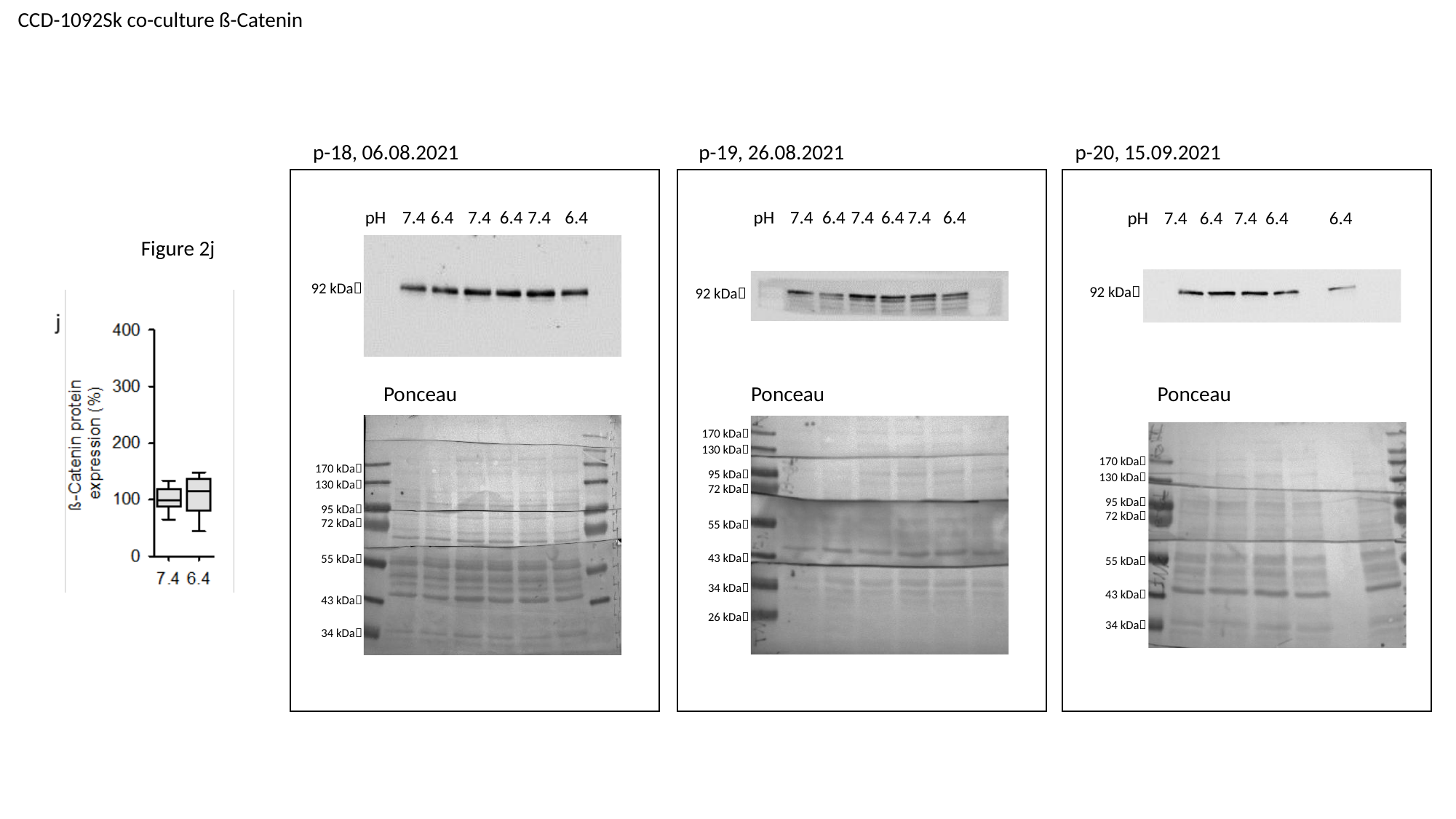

CCD-1092Sk co-culture ß-Catenin
p-20, 15.09.2021
p-18, 06.08.2021
p-19, 26.08.2021
pH
7.4
6.4
7.4
6.4
7.4
6.4
pH
7.4
6.4
7.4
6.4
7.4
6.4
pH
7.4
6.4
7.4
6.4
6.4
Figure 2j
92 kDa
92 kDa
92 kDa
Ponceau
Ponceau
Ponceau
170 kDa
130 kDa
95 kDa
72 kDa
55 kDa
43 kDa
34 kDa
26 kDa
170 kDa
130 kDa
95 kDa
72 kDa
55 kDa
43 kDa
34 kDa
170 kDa
130 kDa
95 kDa
72 kDa
55 kDa
43 kDa
34 kDa

## Slide 27
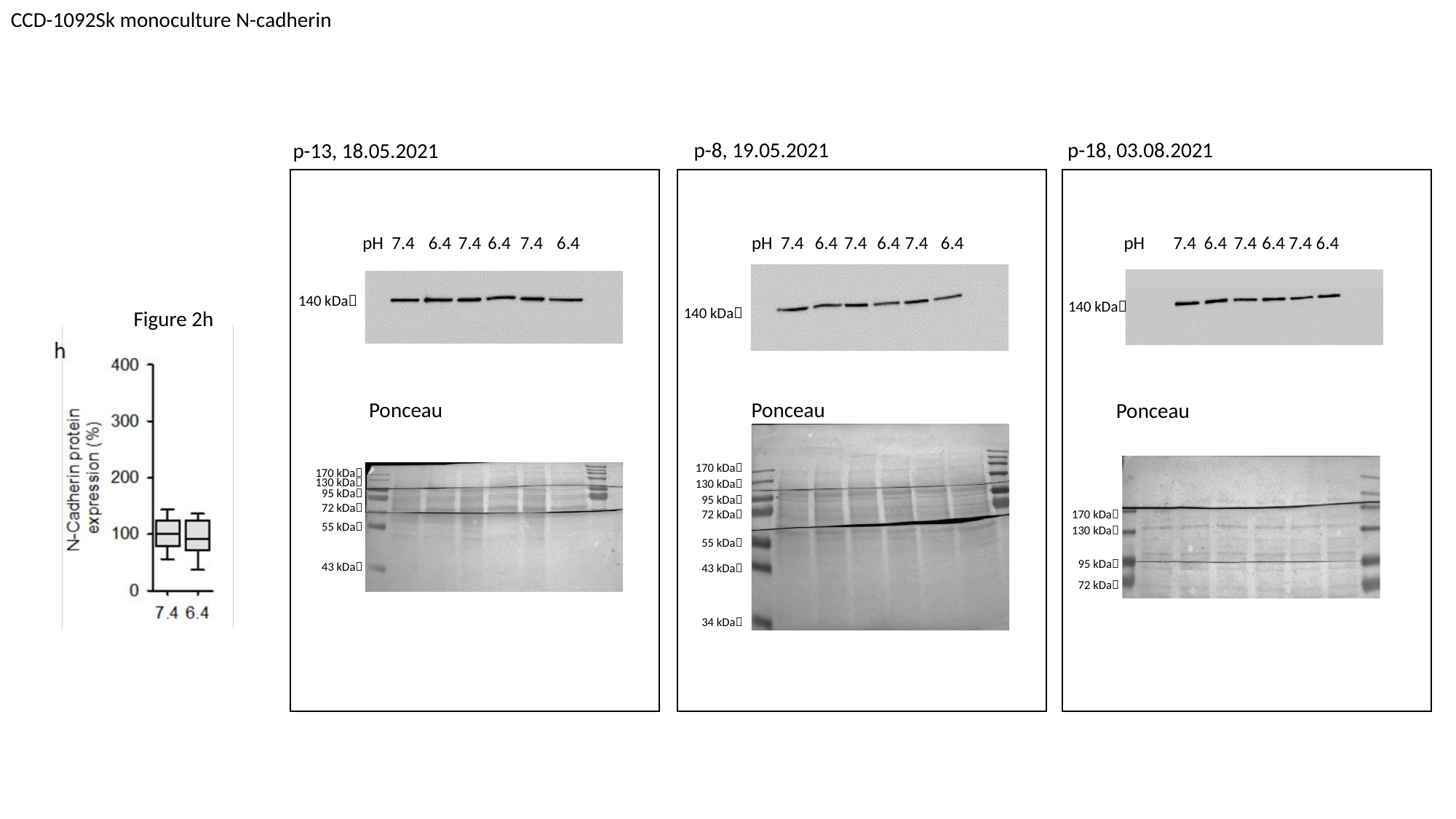

CCD-1092Sk monoculture N-cadherin
p-8, 19.05.2021
p-18, 03.08.2021
p-13, 18.05.2021
pH
7.4
6.4
7.4
6.4
7.4
6.4
pH
7.4
6.4
7.4
6.4
7.4
6.4
pH
7.4
6.4
7.4
6.4
7.4
6.4
140 kDa
140 kDa
140 kDa
Figure 2h
Ponceau
Ponceau
Ponceau
170 kDa
130 kDa
95 kDa
72 kDa
55 kDa
43 kDa
34 kDa
170 kDa
130 kDa
95 kDa
72 kDa
55 kDa
43 kDa
170 kDa
130 kDa
95 kDa
72 kDa

## Slide 28
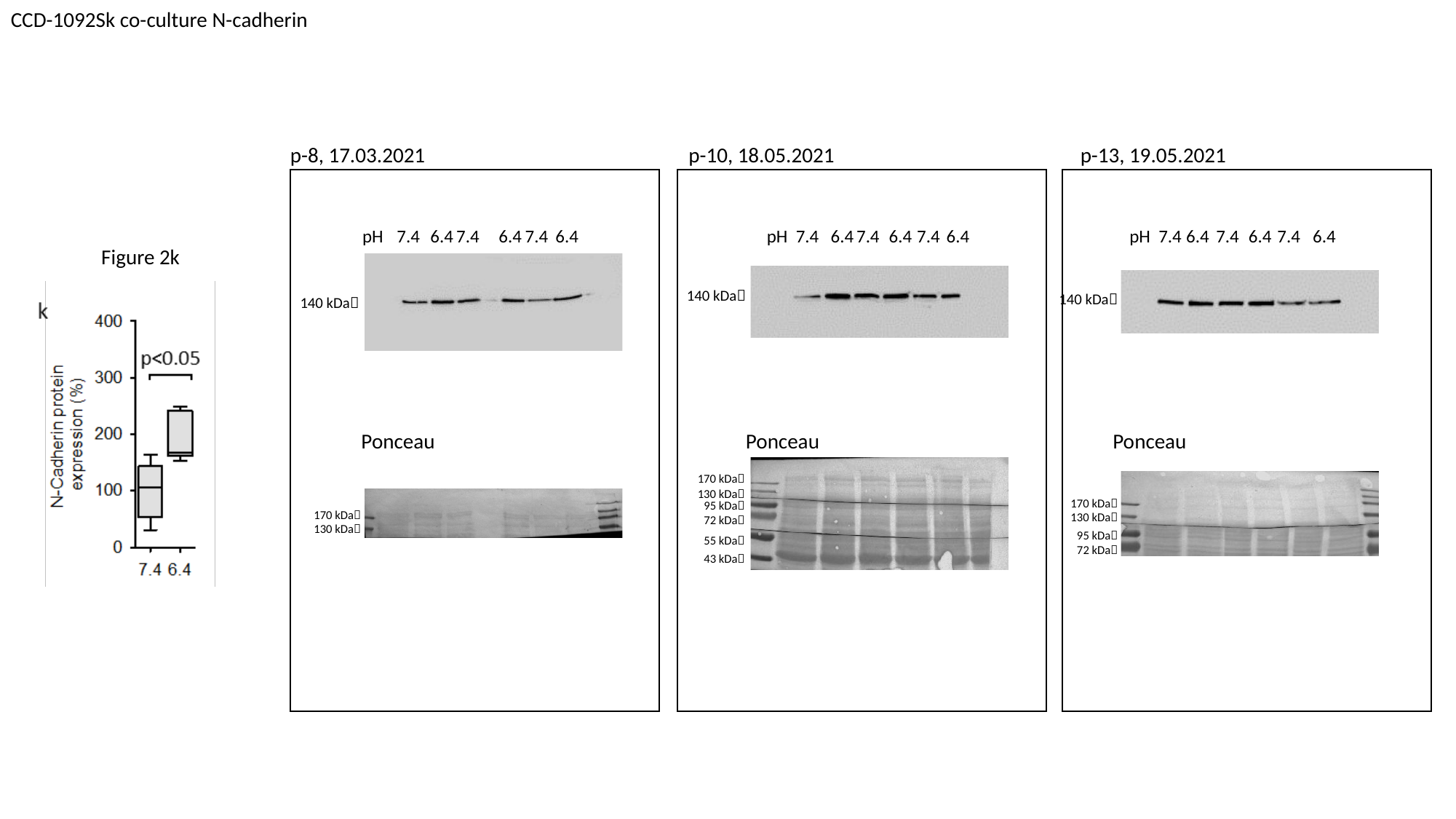

CCD-1092Sk co-culture N-cadherin
p-10, 18.05.2021
p-13, 19.05.2021
p-8, 17.03.2021
pH
7.4
6.4
7.4
6.4
7.4
6.4
pH
7.4
6.4
7.4
6.4
7.4
6.4
pH
7.4
6.4
7.4
6.4
7.4
6.4
Figure 2k
140 kDa
140 kDa
140 kDa
Ponceau
Ponceau
Ponceau
170 kDa
130 kDa
95 kDa
72 kDa
55 kDa
43 kDa
170 kDa
130 kDa
95 kDa
72 kDa
170 kDa
130 kDa

## Slide 29
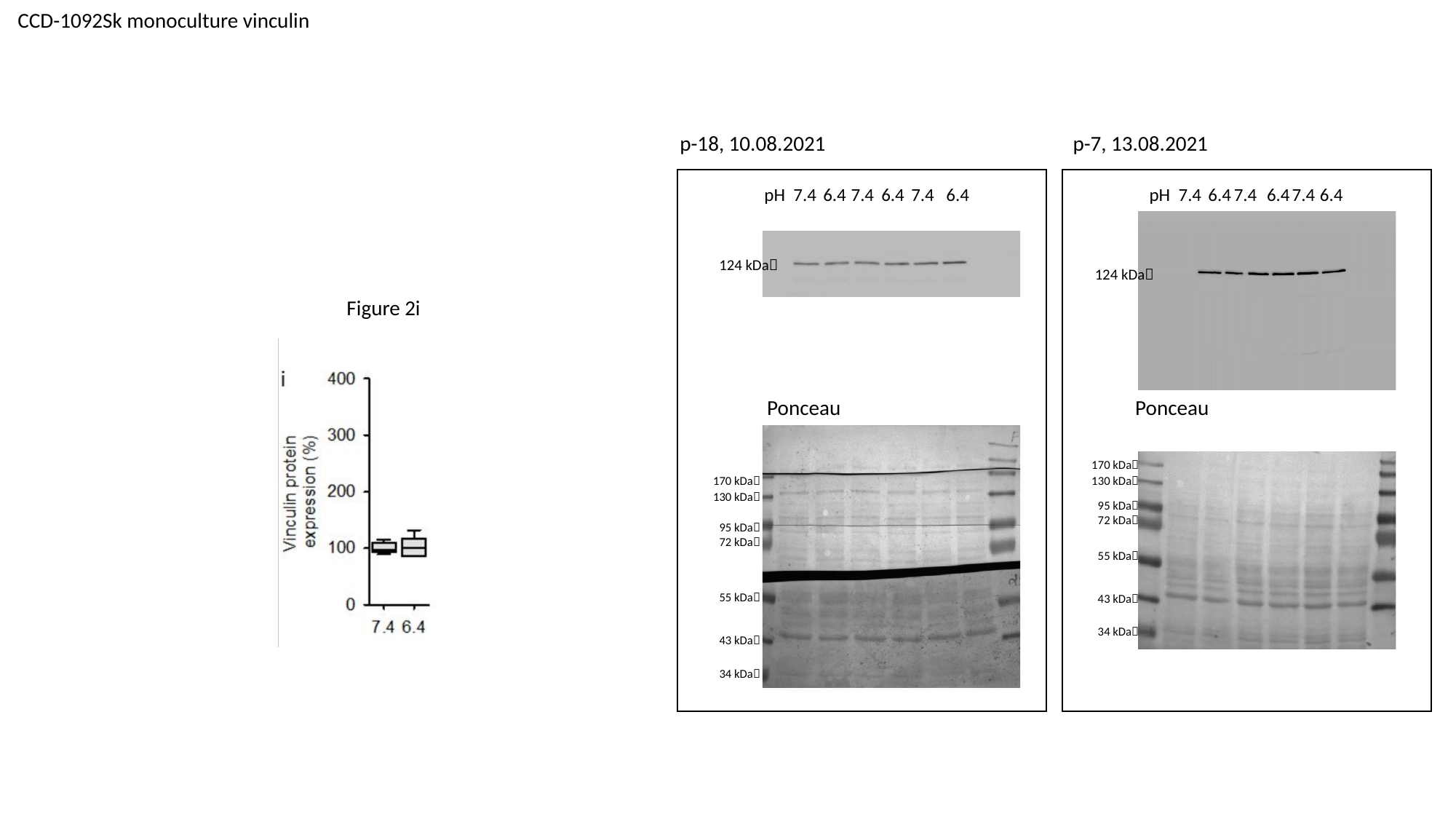

CCD-1092Sk monoculture vinculin
p-18, 10.08.2021
p-7, 13.08.2021
pH
7.4
6.4
7.4
6.4
7.4
6.4
pH
7.4
6.4
7.4
6.4
7.4
6.4
124 kDa
124 kDa
Figure 2i
Ponceau
Ponceau
170 kDa
130 kDa
95 kDa
72 kDa
55 kDa
43 kDa
34 kDa
170 kDa
130 kDa
95 kDa
72 kDa
55 kDa
43 kDa
34 kDa

## Slide 30
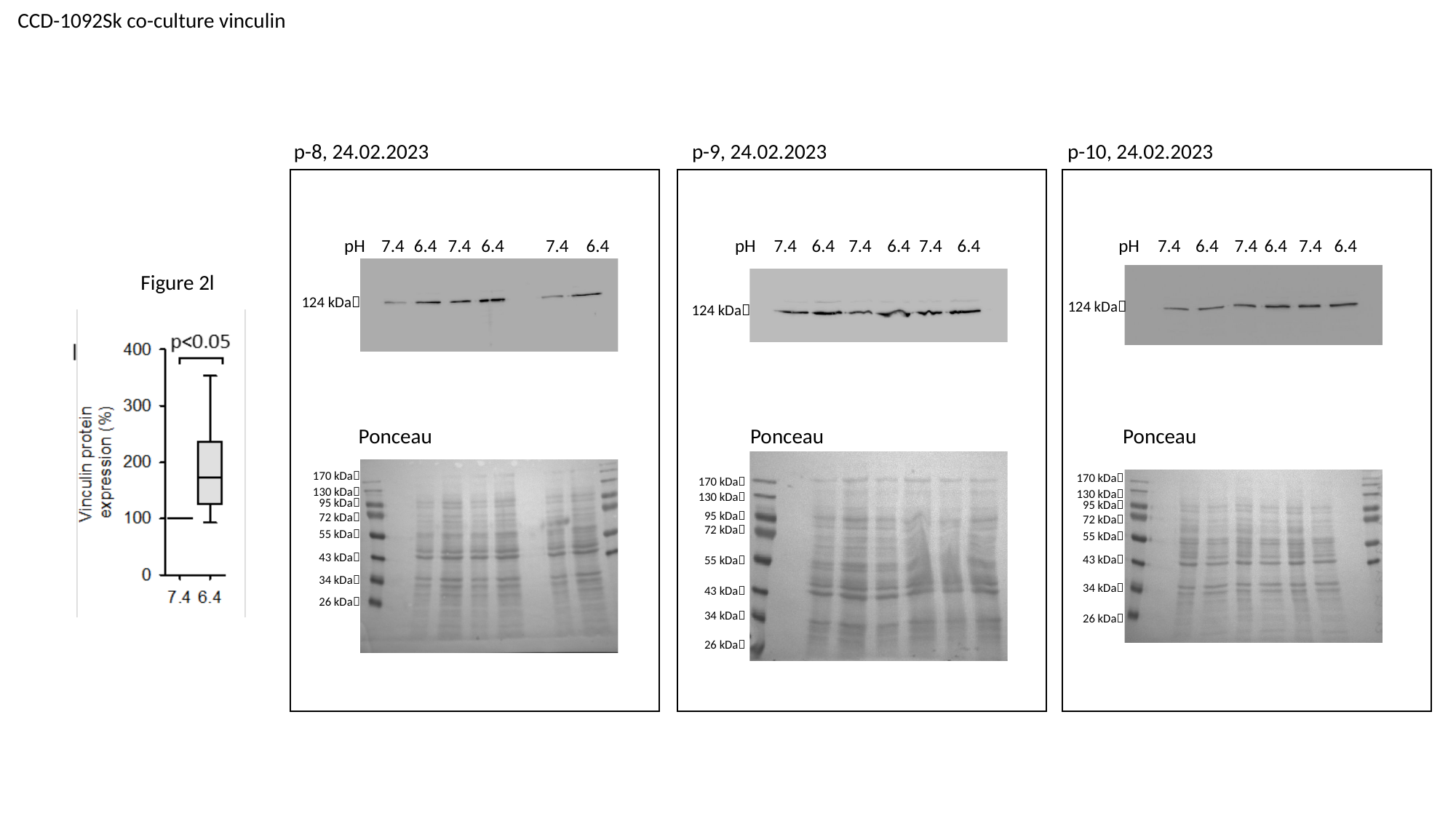

CCD-1092Sk co-culture vinculin
p-8, 24.02.2023
p-9, 24.02.2023
p-10, 24.02.2023
pH
7.4
6.4
7.4
6.4
7.4
6.4
pH
7.4
6.4
7.4
6.4
7.4
6.4
pH
7.4
6.4
7.4
6.4
7.4
6.4
Figure 2l
124 kDa
124 kDa
124 kDa
Ponceau
Ponceau
Ponceau
170 kDa
130 kDa
95 kDa
72 kDa
55 kDa
43 kDa
34 kDa
26 kDa
170 kDa
130 kDa
95 kDa
72 kDa
55 kDa
43 kDa
34 kDa
26 kDa
170 kDa
130 kDa
95 kDa
72 kDa
55 kDa
43 kDa
34 kDa
26 kDa

## Slide 31
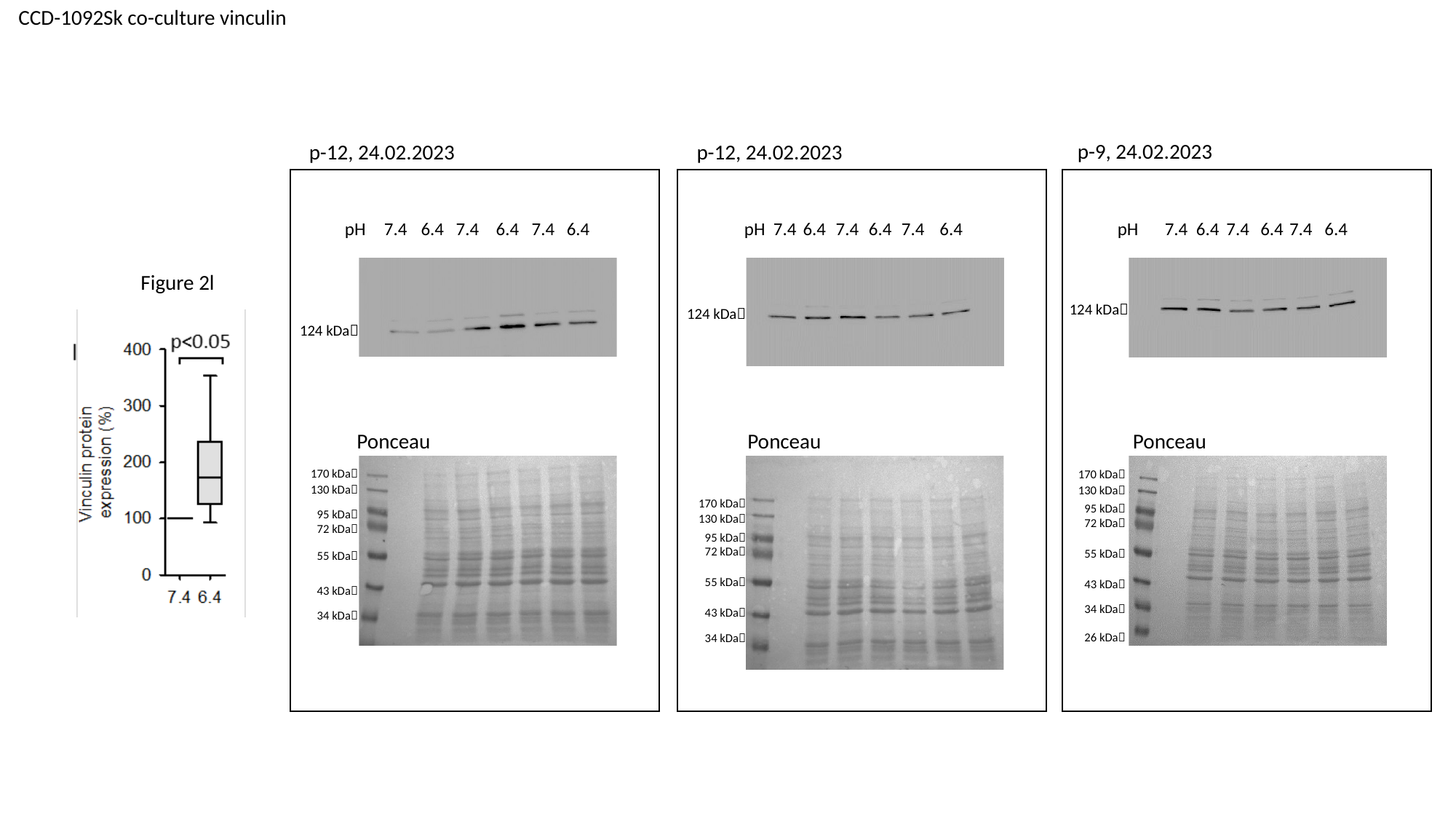

CCD-1092Sk co-culture vinculin
p-9, 24.02.2023
p-12, 24.02.2023
p-12, 24.02.2023
pH
7.4
6.4
7.4
6.4
7.4
6.4
pH
7.4
6.4
7.4
6.4
7.4
6.4
pH
7.4
6.4
7.4
6.4
7.4
6.4
Figure 2l
124 kDa
124 kDa
124 kDa
Ponceau
Ponceau
Ponceau
170 kDa
130 kDa
95 kDa
72 kDa
55 kDa
43 kDa
34 kDa
170 kDa
130 kDa
95 kDa
72 kDa
55 kDa
43 kDa
34 kDa
26 kDa
170 kDa
130 kDa
95 kDa
72 kDa
55 kDa
43 kDa
34 kDa

## Slide 32
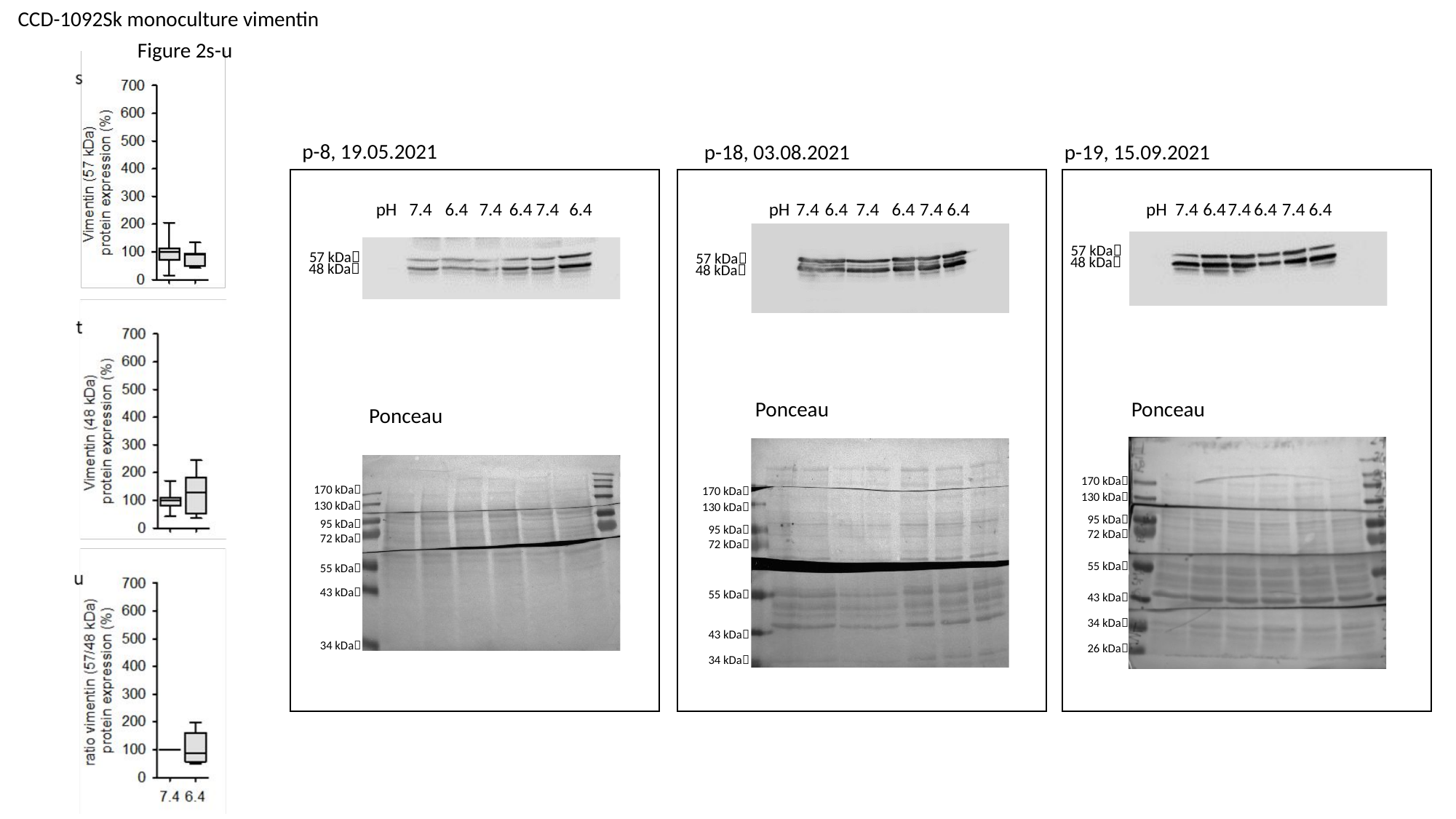

CCD-1092Sk monoculture vimentin
Figure 2s-u
p-8, 19.05.2021
p-18, 03.08.2021
p-19, 15.09.2021
pH
7.4
6.4
7.4
6.4
7.4
6.4
pH
7.4
6.4
7.4
6.4
7.4
6.4
pH
7.4
6.4
7.4
6.4
7.4
6.4
57 kDa
57 kDa
57 kDa
48 kDa
48 kDa
48 kDa
Ponceau
Ponceau
Ponceau
170 kDa
130 kDa
95 kDa
72 kDa
55 kDa
43 kDa
34 kDa
26 kDa
170 kDa
130 kDa
95 kDa
72 kDa
55 kDa
43 kDa
34 kDa
170 kDa
130 kDa
95 kDa
72 kDa
55 kDa
43 kDa
34 kDa

## Slide 33
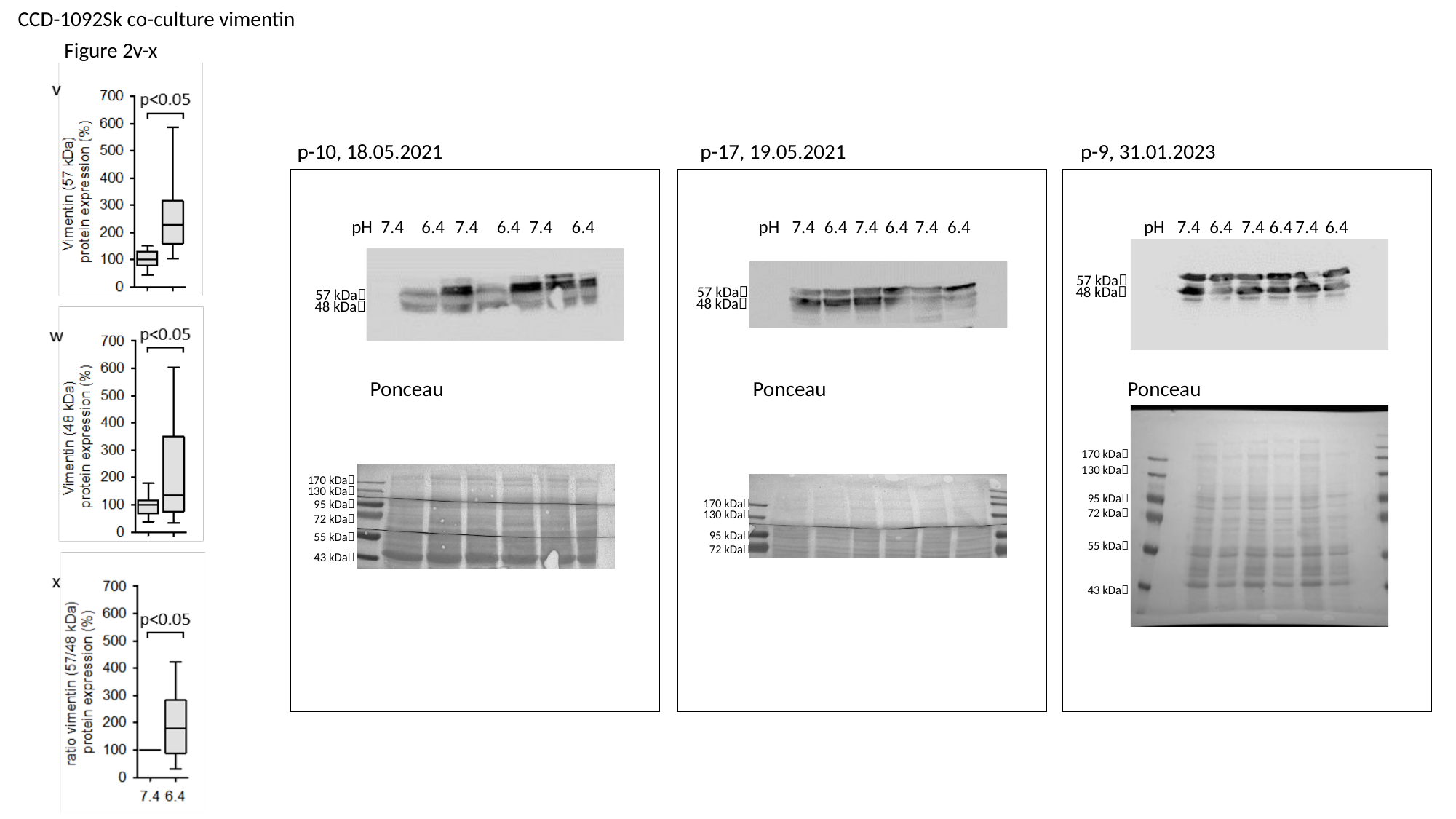

CCD-1092Sk co-culture vimentin
Figure 2v-x
p-10, 18.05.2021
p-17, 19.05.2021
p-9, 31.01.2023
pH
7.4
6.4
7.4
6.4
7.4
6.4
pH
7.4
6.4
7.4
6.4
7.4
6.4
pH
7.4
6.4
7.4
6.4
7.4
6.4
57 kDa
57 kDa
48 kDa
57 kDa
48 kDa
48 kDa
Ponceau
Ponceau
Ponceau
170 kDa
130 kDa
95 kDa
72 kDa
55 kDa
43 kDa
170 kDa
130 kDa
95 kDa
72 kDa
55 kDa
43 kDa
170 kDa
130 kDa
95 kDa
72 kDa

## Slide 34
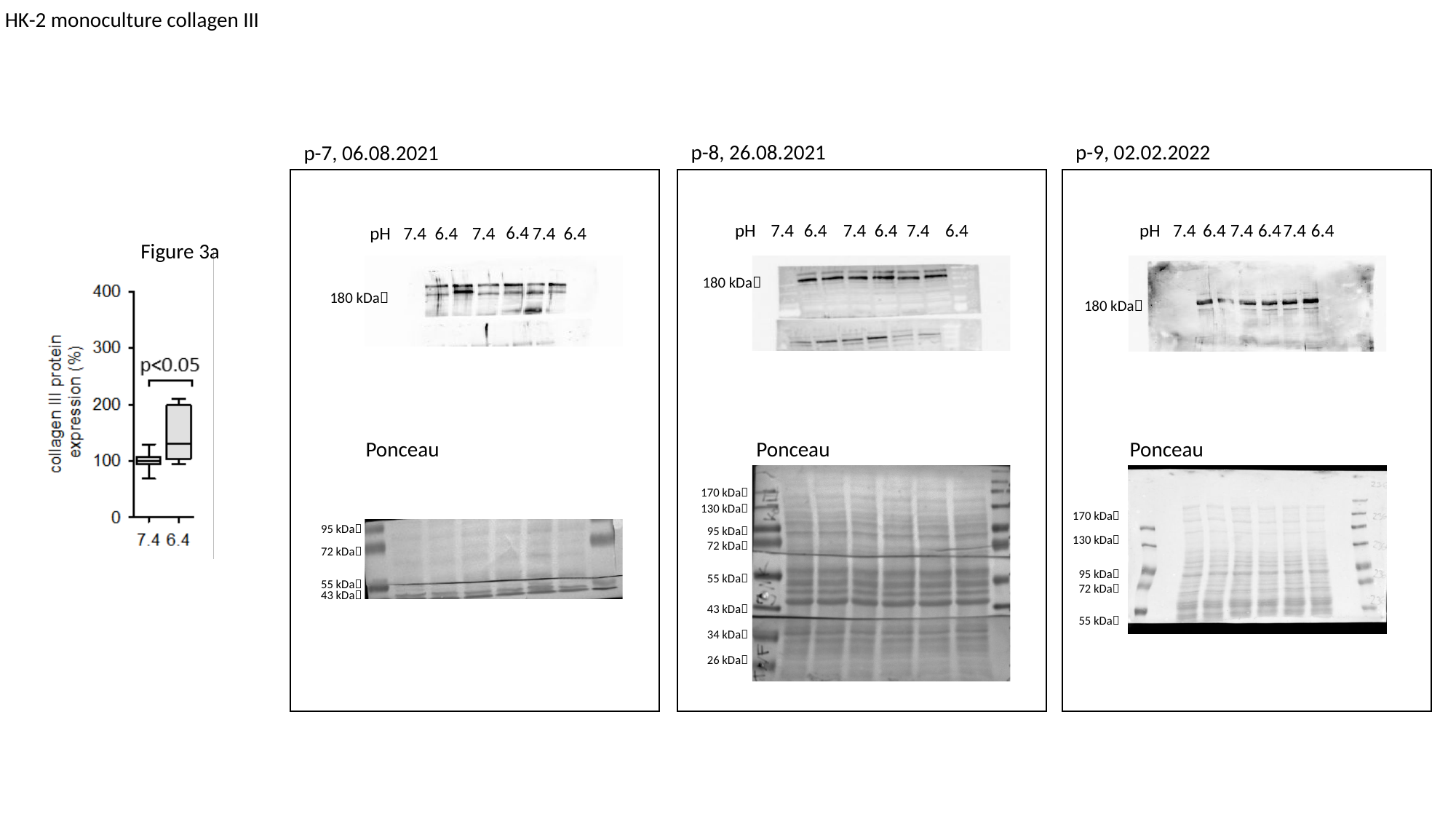

HK-2 monoculture collagen III
p-9, 02.02.2022
p-8, 26.08.2021
p-7, 06.08.2021
pH
7.4
6.4
7.4
6.4
7.4
6.4
pH
7.4
6.4
7.4
6.4
7.4
6.4
6.4
pH
7.4
6.4
7.4
7.4
6.4
Figure 3a
180 kDa
180 kDa
180 kDa
Ponceau
Ponceau
Ponceau
170 kDa
130 kDa
95 kDa
72 kDa
55 kDa
43 kDa
34 kDa
26 kDa
170 kDa
130 kDa
95 kDa
72 kDa
55 kDa
95 kDa
72 kDa
55 kDa
43 kDa

## Slide 35
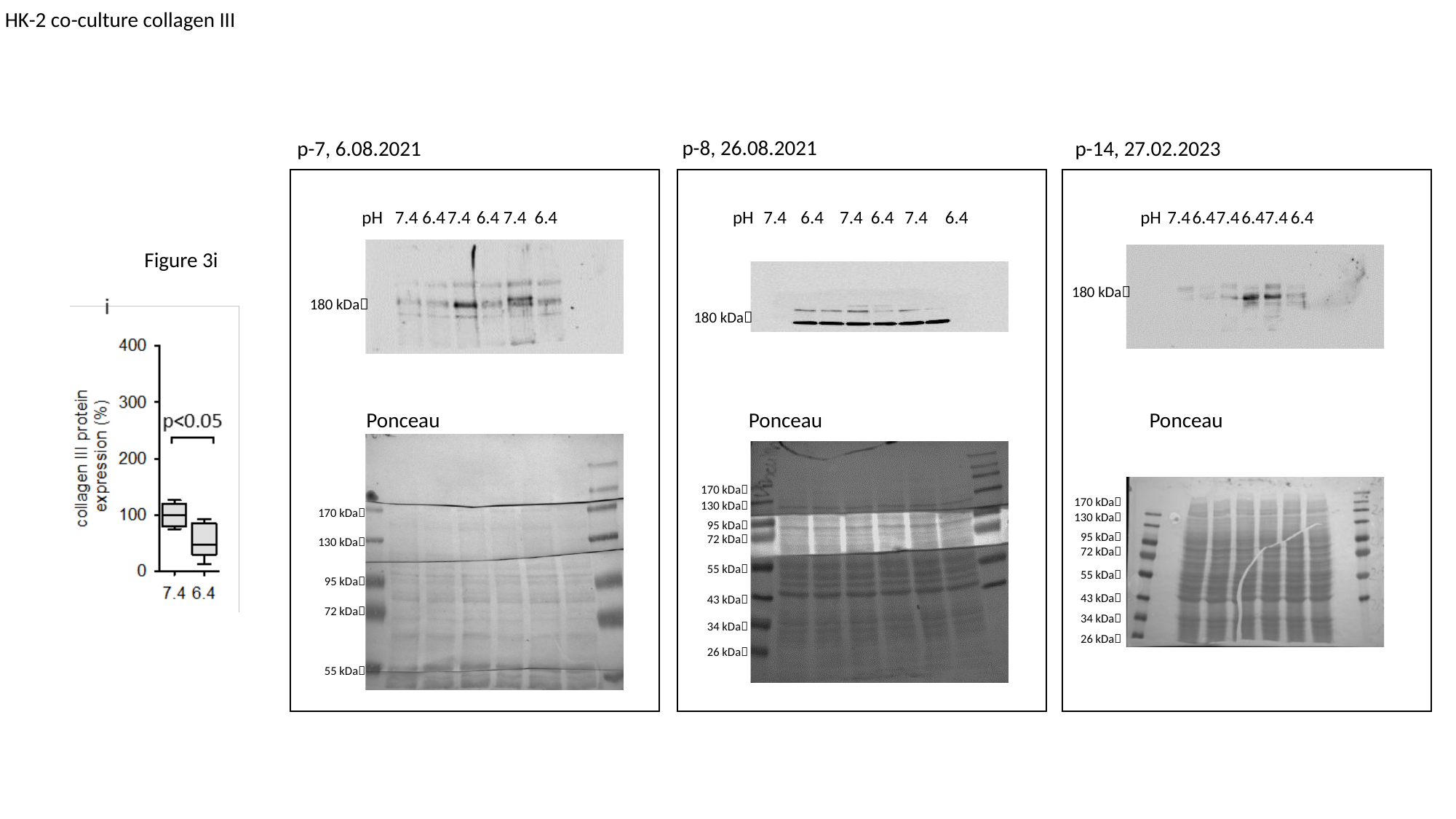

HK-2 co-culture collagen III
p-8, 26.08.2021
p-7, 6.08.2021
p-14, 27.02.2023
pH
7.4
6.4
7.4
6.4
7.4
6.4
pH
7.4
6.4
7.4
6.4
7.4
6.4
pH
7.4
6.4
7.4
6.4
7.4
6.4
Figure 3i
180 kDa
180 kDa
180 kDa
Ponceau
Ponceau
Ponceau
170 kDa
130 kDa
95 kDa
72 kDa
55 kDa
43 kDa
34 kDa
26 kDa
170 kDa
130 kDa
95 kDa
72 kDa
55 kDa
43 kDa
34 kDa
26 kDa
170 kDa
130 kDa
95 kDa
72 kDa
55 kDa

## Slide 36
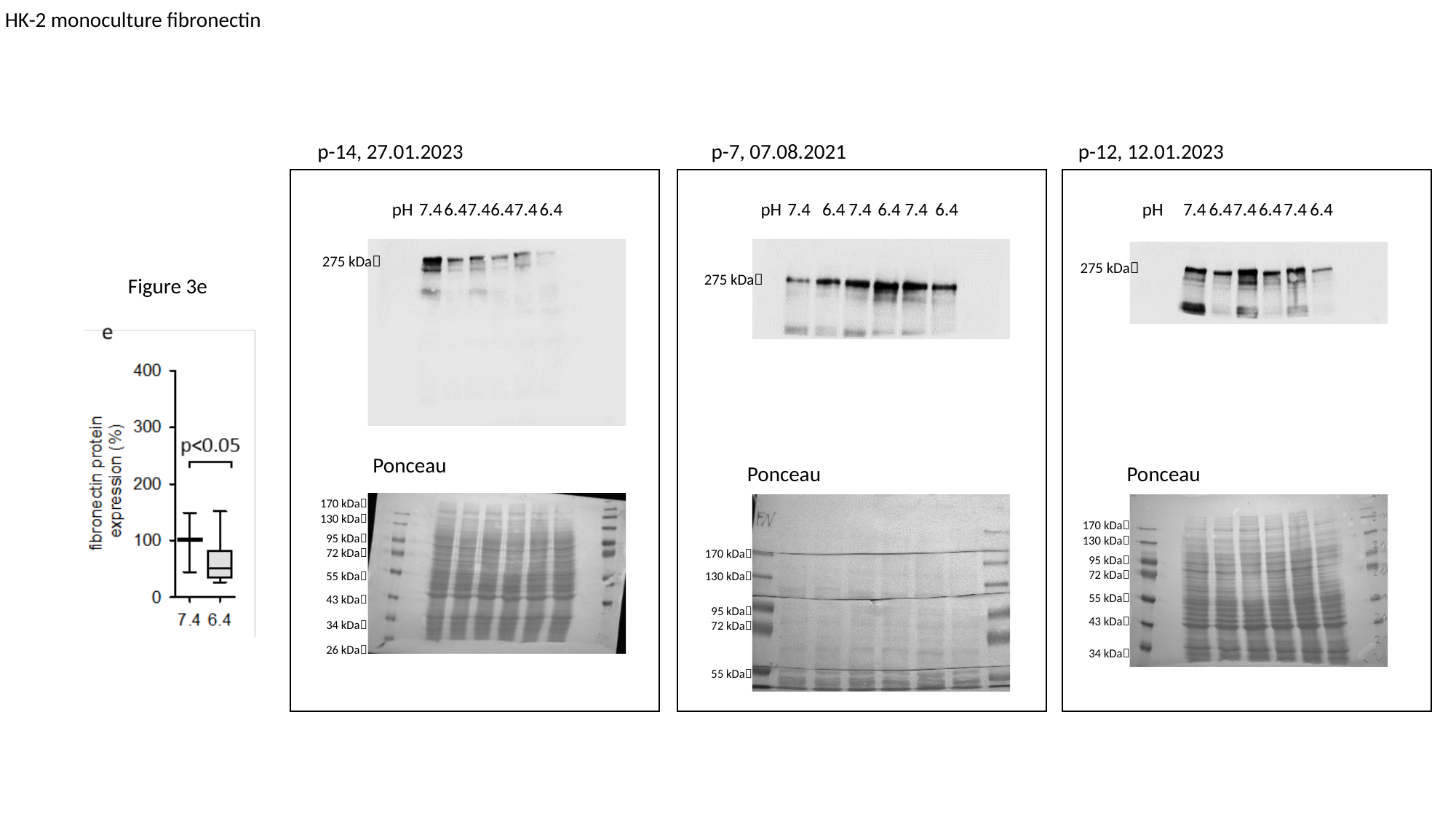

HK-2 monoculture fibronectin
p-7, 07.08.2021
p-12, 12.01.2023
p-14, 27.01.2023
pH
7.4
6.4
7.4
6.4
7.4
6.4
pH
7.4
6.4
7.4
6.4
7.4
6.4
pH
7.4
6.4
7.4
6.4
7.4
6.4
275 kDa
275 kDa
275 kDa
Figure 3e
Ponceau
Ponceau
Ponceau
170 kDa
130 kDa
95 kDa
72 kDa
55 kDa
43 kDa
34 kDa
26 kDa
170 kDa
130 kDa
95 kDa
72 kDa
55 kDa
43 kDa
34 kDa
170 kDa
130 kDa
95 kDa
72 kDa
55 kDa

## Slide 37
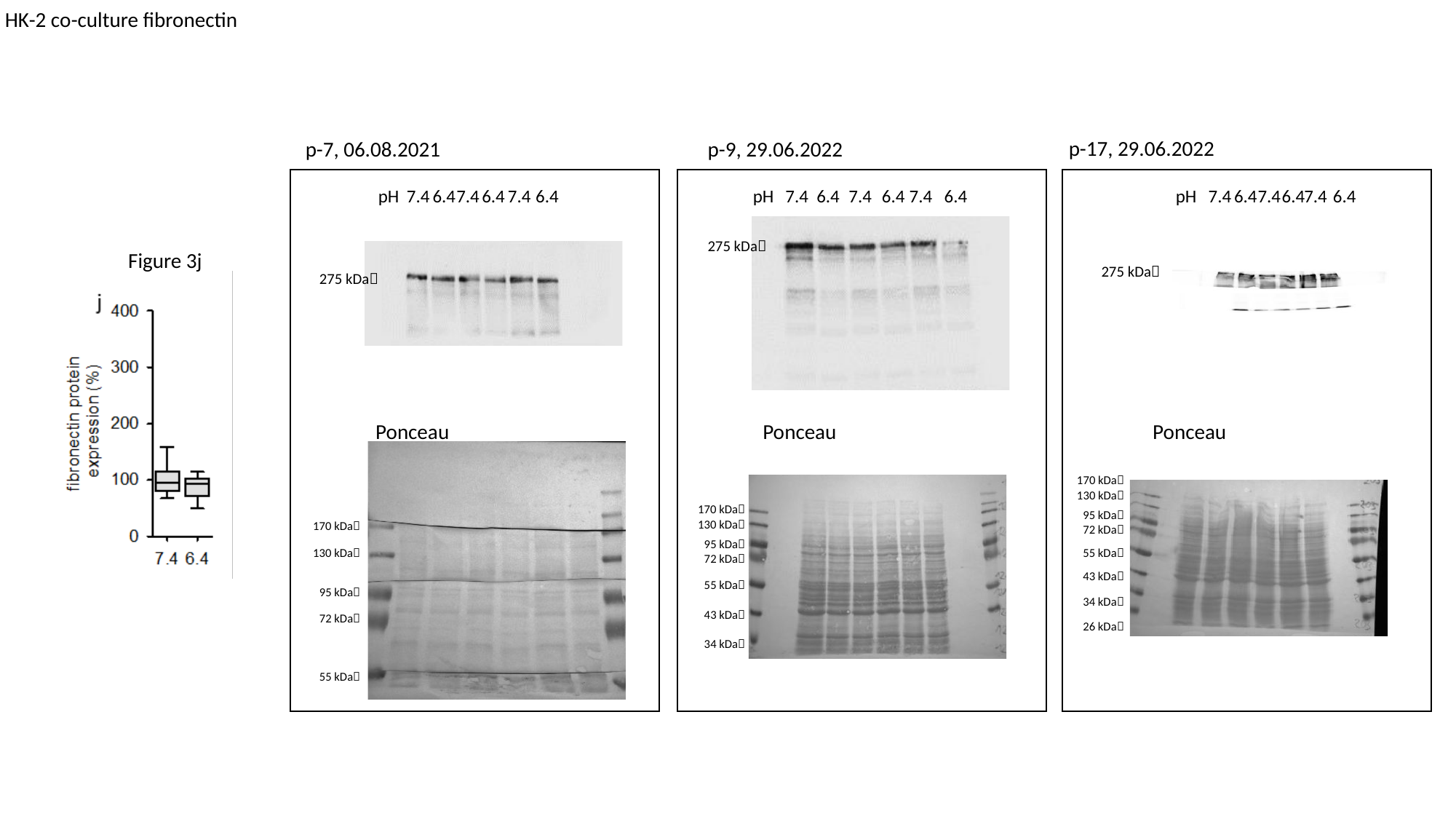

HK-2 co-culture fibronectin
p-17, 29.06.2022
p-9, 29.06.2022
p-7, 06.08.2021
pH
7.4
6.4
7.4
6.4
7.4
6.4
pH
7.4
6.4
7.4
6.4
7.4
6.4
pH
7.4
6.4
7.4
6.4
7.4
6.4
275 kDa
Figure 3j
275 kDa
275 kDa
Ponceau
Ponceau
Ponceau
170 kDa
130 kDa
95 kDa
72 kDa
55 kDa
43 kDa
34 kDa
26 kDa
170 kDa
130 kDa
95 kDa
72 kDa
55 kDa
43 kDa
34 kDa
170 kDa
130 kDa
95 kDa
72 kDa
55 kDa

## Slide 38
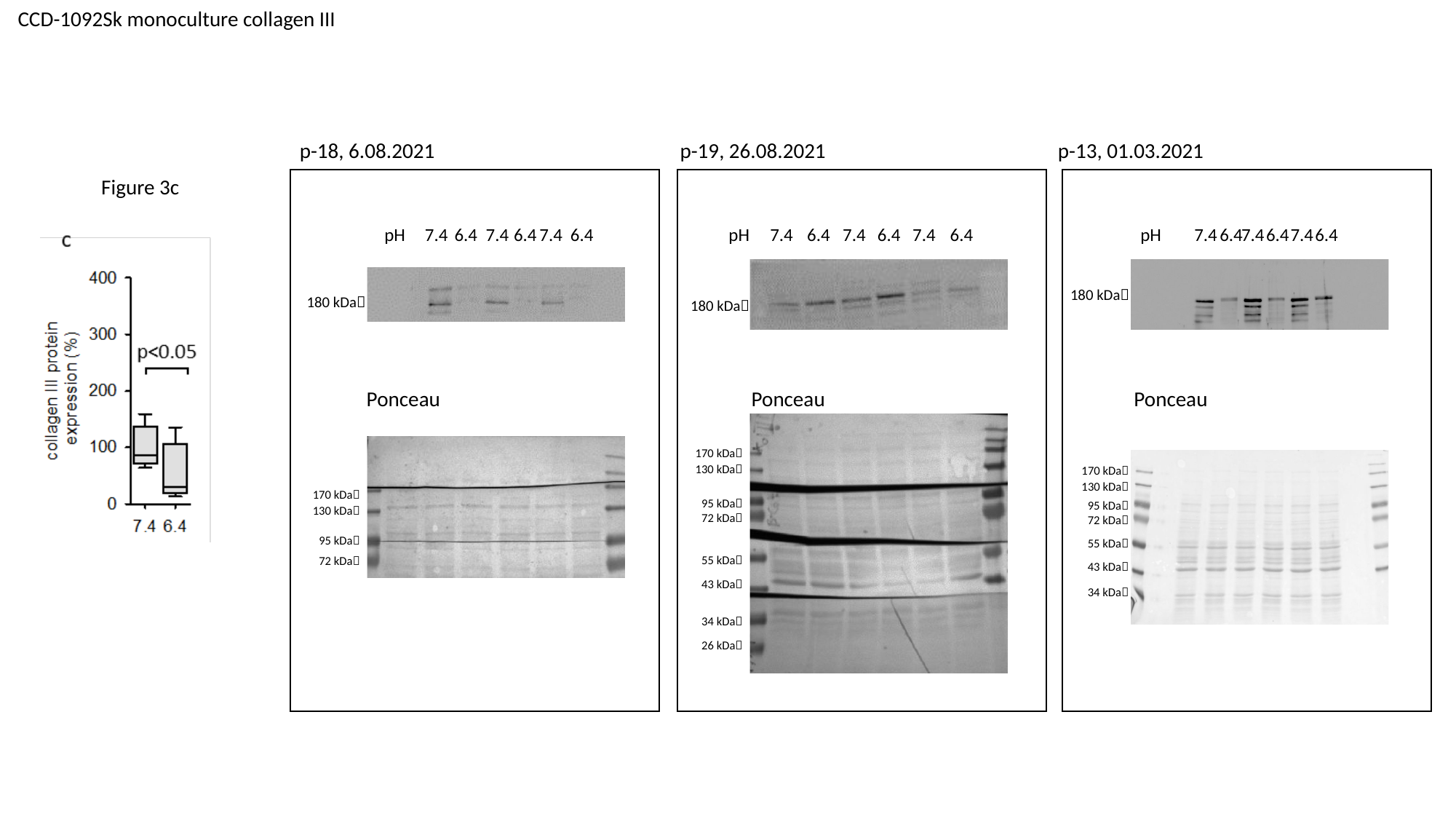

CCD-1092Sk monoculture collagen III
p-18, 6.08.2021
p-19, 26.08.2021
p-13, 01.03.2021
Figure 3c
pH
7.4
6.4
7.4
6.4
7.4
6.4
pH
7.4
6.4
7.4
6.4
7.4
6.4
pH
7.4
6.4
7.4
6.4
7.4
6.4
180 kDa
180 kDa
180 kDa
Ponceau
Ponceau
Ponceau
170 kDa
130 kDa
95 kDa
72 kDa
55 kDa
43 kDa
34 kDa
26 kDa
170 kDa
130 kDa
95 kDa
72 kDa
55 kDa
43 kDa
34 kDa
170 kDa
130 kDa
95 kDa
72 kDa

## Slide 39
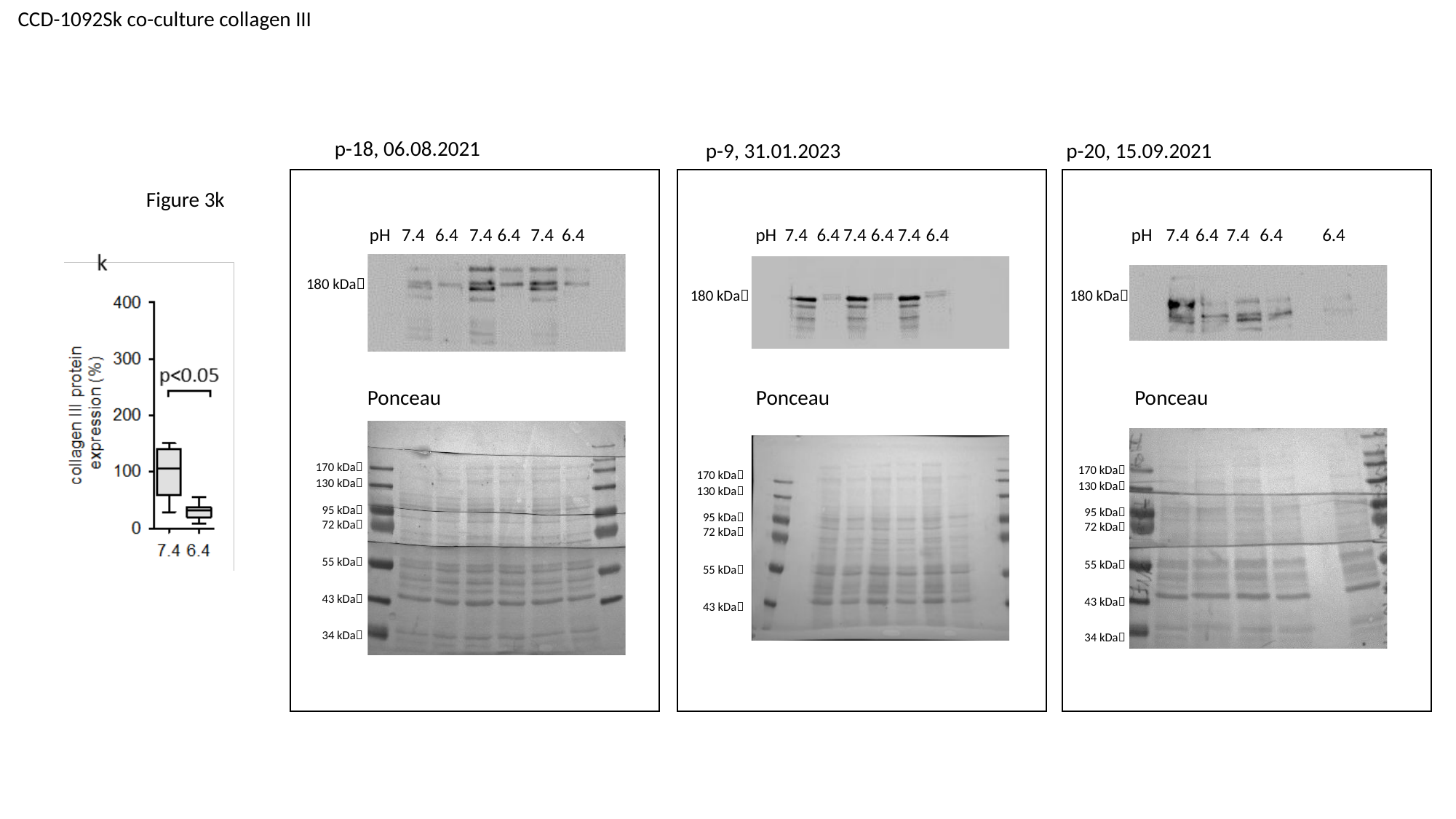

CCD-1092Sk co-culture collagen III
p-18, 06.08.2021
p-20, 15.09.2021
p-9, 31.01.2023
Figure 3k
pH
7.4
6.4
7.4
6.4
7.4
6.4
pH
7.4
6.4
7.4
6.4
7.4
6.4
pH
7.4
6.4
7.4
6.4
6.4
180 kDa
180 kDa
180 kDa
Ponceau
Ponceau
Ponceau
170 kDa
130 kDa
95 kDa
72 kDa
55 kDa
43 kDa
34 kDa
170 kDa
130 kDa
95 kDa
72 kDa
55 kDa
43 kDa
34 kDa
170 kDa
130 kDa
95 kDa
72 kDa
55 kDa
43 kDa

## Slide 40
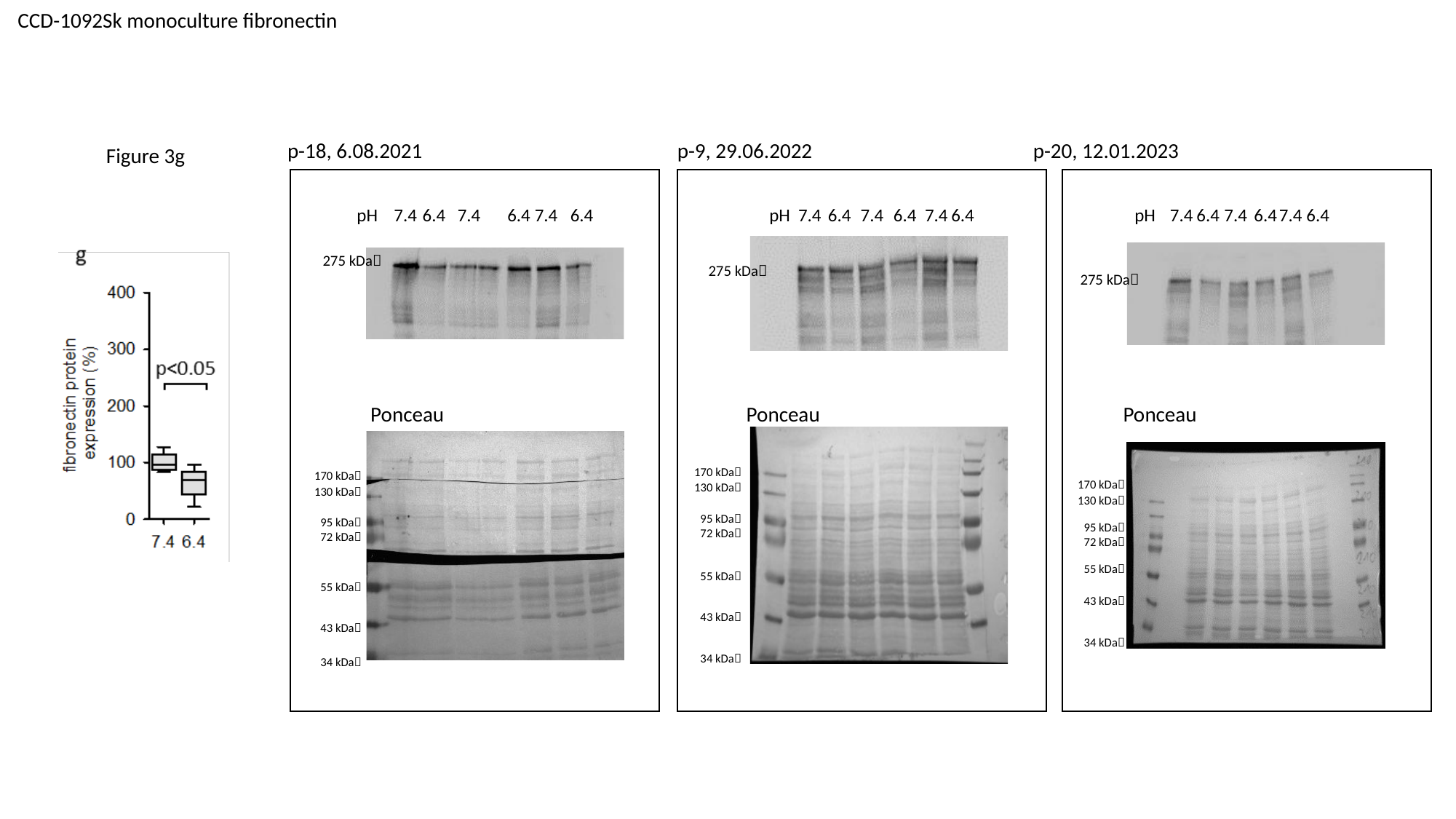

CCD-1092Sk monoculture fibronectin
p-18, 6.08.2021
p-9, 29.06.2022
p-20, 12.01.2023
Figure 3g
pH
7.4
6.4
7.4
6.4
7.4
6.4
pH
7.4
6.4
7.4
6.4
7.4
6.4
pH
7.4
6.4
7.4
6.4
7.4
6.4
275 kDa
275 kDa
275 kDa
Ponceau
Ponceau
Ponceau
170 kDa
130 kDa
95 kDa
72 kDa
55 kDa
43 kDa
34 kDa
170 kDa
130 kDa
95 kDa
72 kDa
55 kDa
43 kDa
34 kDa
170 kDa
130 kDa
95 kDa
72 kDa
55 kDa
43 kDa
34 kDa

## Slide 41
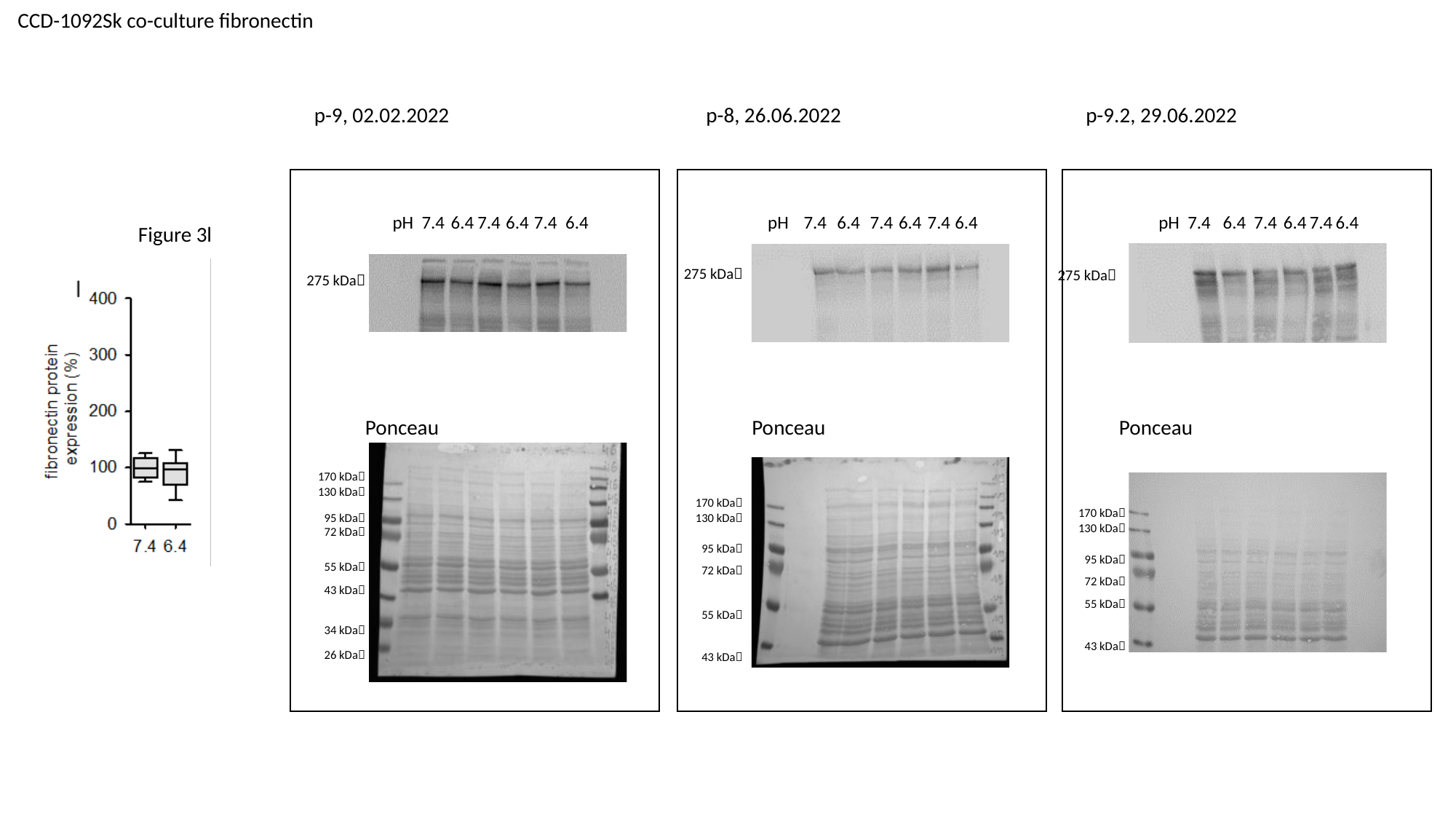

CCD-1092Sk co-culture fibronectin
p-9, 02.02.2022
p-8, 26.06.2022
p-9.2, 29.06.2022
pH
7.4
6.4
7.4
6.4
7.4
6.4
pH
7.4
6.4
7.4
6.4
7.4
6.4
pH
7.4
6.4
7.4
6.4
7.4
6.4
Figure 3l
275 kDa
275 kDa
275 kDa
Ponceau
Ponceau
Ponceau
170 kDa
130 kDa
95 kDa
72 kDa
55 kDa
43 kDa
34 kDa
26 kDa
170 kDa
130 kDa
95 kDa
72 kDa
55 kDa
43 kDa
170 kDa
130 kDa
95 kDa
72 kDa
55 kDa
43 kDa

## Slide 42
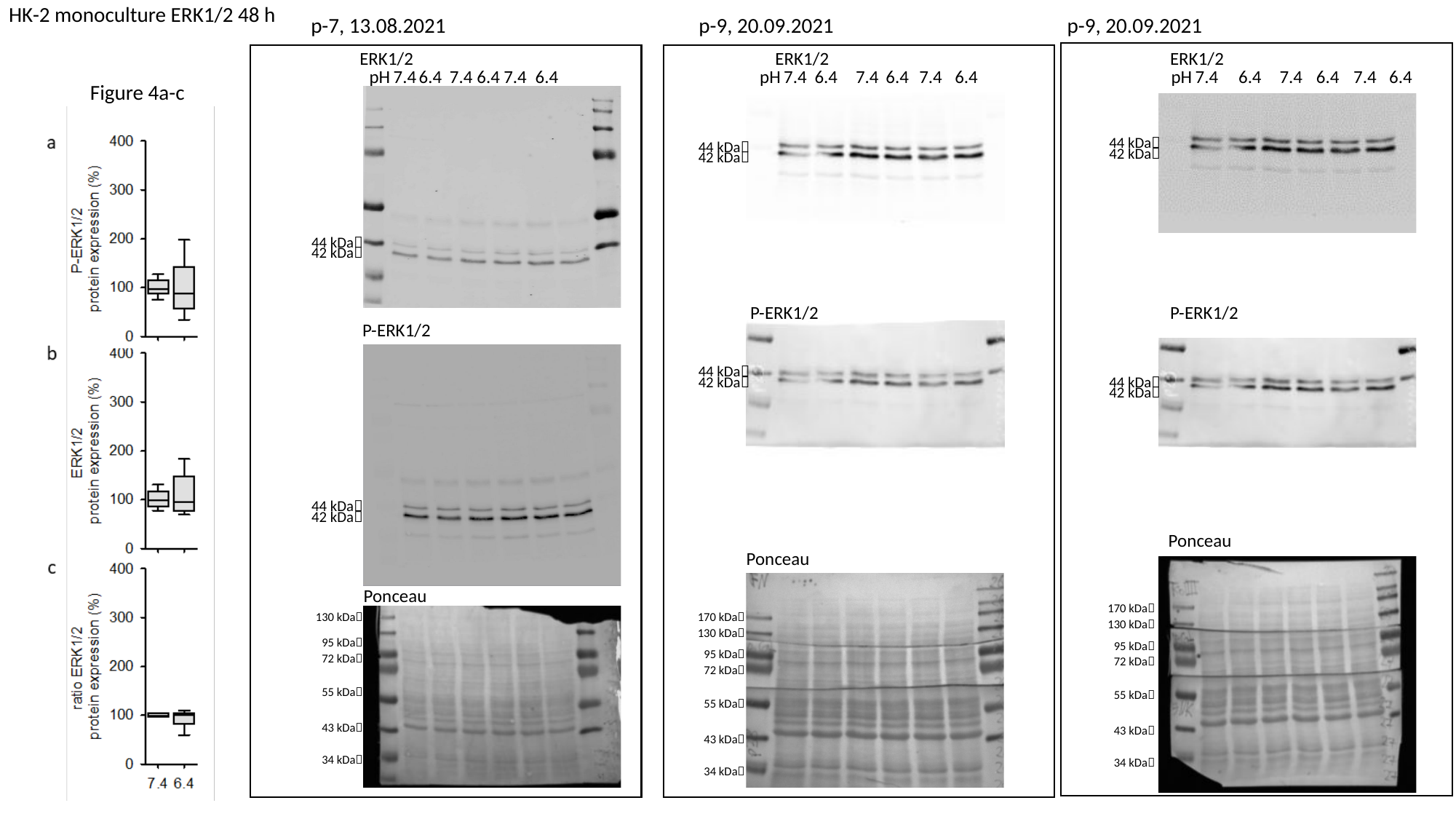

HK-2 monoculture ERK1/2 48 h
p-7, 13.08.2021
p-9, 20.09.2021
p-9, 20.09.2021
ERK1/2
ERK1/2
ERK1/2
pH
7.4
6.4
7.4
6.4
7.4
6.4
pH
7.4
6.4
7.4
6.4
7.4
6.4
pH
7.4
6.4
7.4
6.4
7.4
6.4
Figure 4a-c
44 kDa
44 kDa
42 kDa
42 kDa
44 kDa
42 kDa
P-ERK1/2
P-ERK1/2
P-ERK1/2
44 kDa
42 kDa
44 kDa
42 kDa
44 kDa
42 kDa
Ponceau
Ponceau
Ponceau
170 kDa
130 kDa
95 kDa
72 kDa
55 kDa
43 kDa
34 kDa
170 kDa
130 kDa
95 kDa
72 kDa
55 kDa
43 kDa
34 kDa
130 kDa
95 kDa
72 kDa
55 kDa
43 kDa
34 kDa

## Slide 43
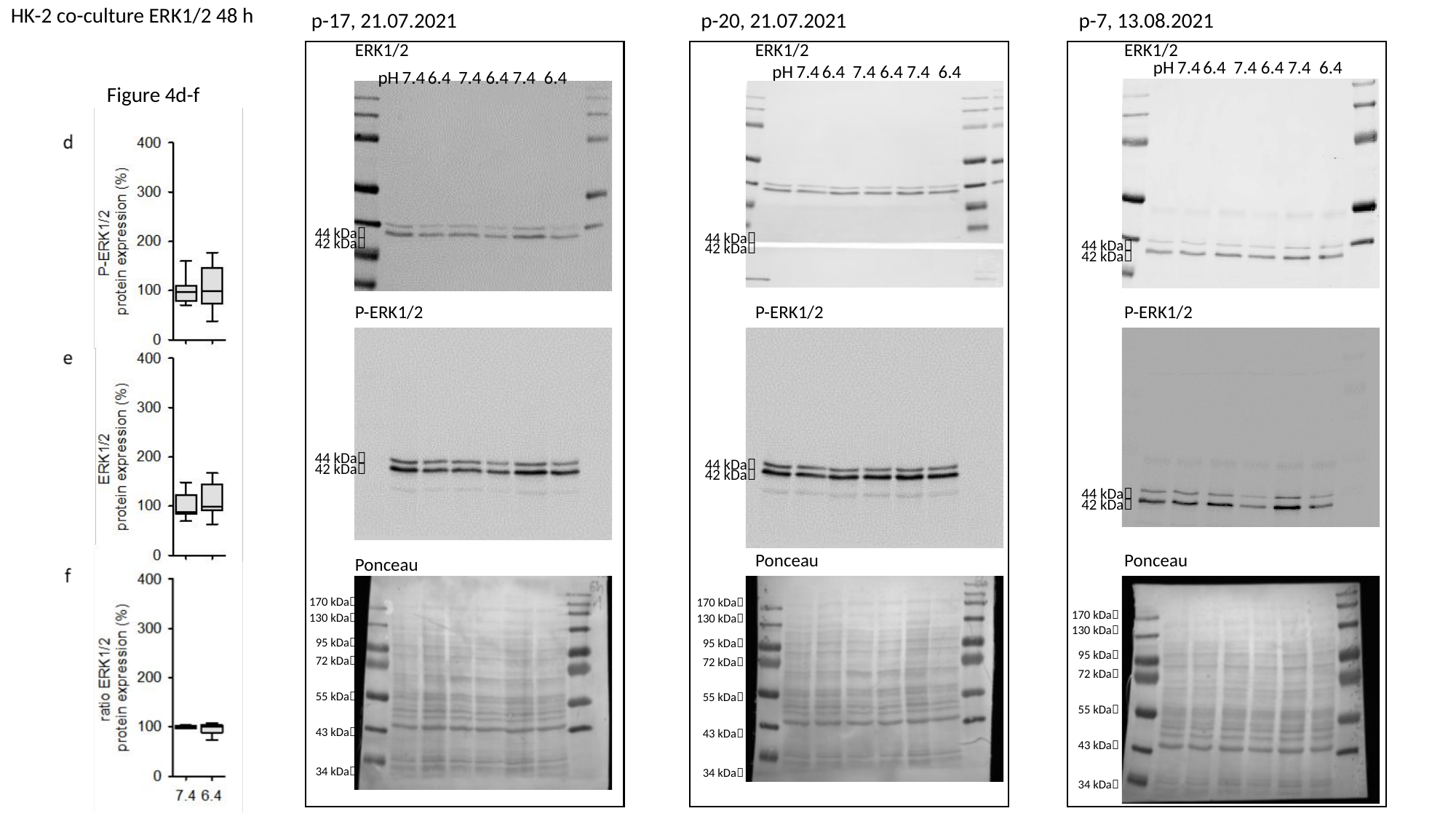

HK-2 co-culture ERK1/2 48 h
p-17, 21.07.2021
p-20, 21.07.2021
p-7, 13.08.2021
ERK1/2
ERK1/2
ERK1/2
pH
7.4
6.4
7.4
6.4
7.4
6.4
pH
7.4
6.4
7.4
6.4
7.4
6.4
pH
7.4
6.4
7.4
6.4
7.4
6.4
Figure 4d-f
44 kDa
44 kDa
42 kDa
44 kDa
42 kDa
42 kDa
P-ERK1/2
P-ERK1/2
P-ERK1/2
44 kDa
44 kDa
42 kDa
42 kDa
44 kDa
42 kDa
Ponceau
Ponceau
Ponceau
170 kDa
130 kDa
95 kDa
72 kDa
55 kDa
43 kDa
34 kDa
170 kDa
130 kDa
95 kDa
72 kDa
55 kDa
43 kDa
34 kDa
170 kDa
130 kDa
95 kDa
72 kDa
55 kDa
43 kDa
34 kDa

## Slide 44
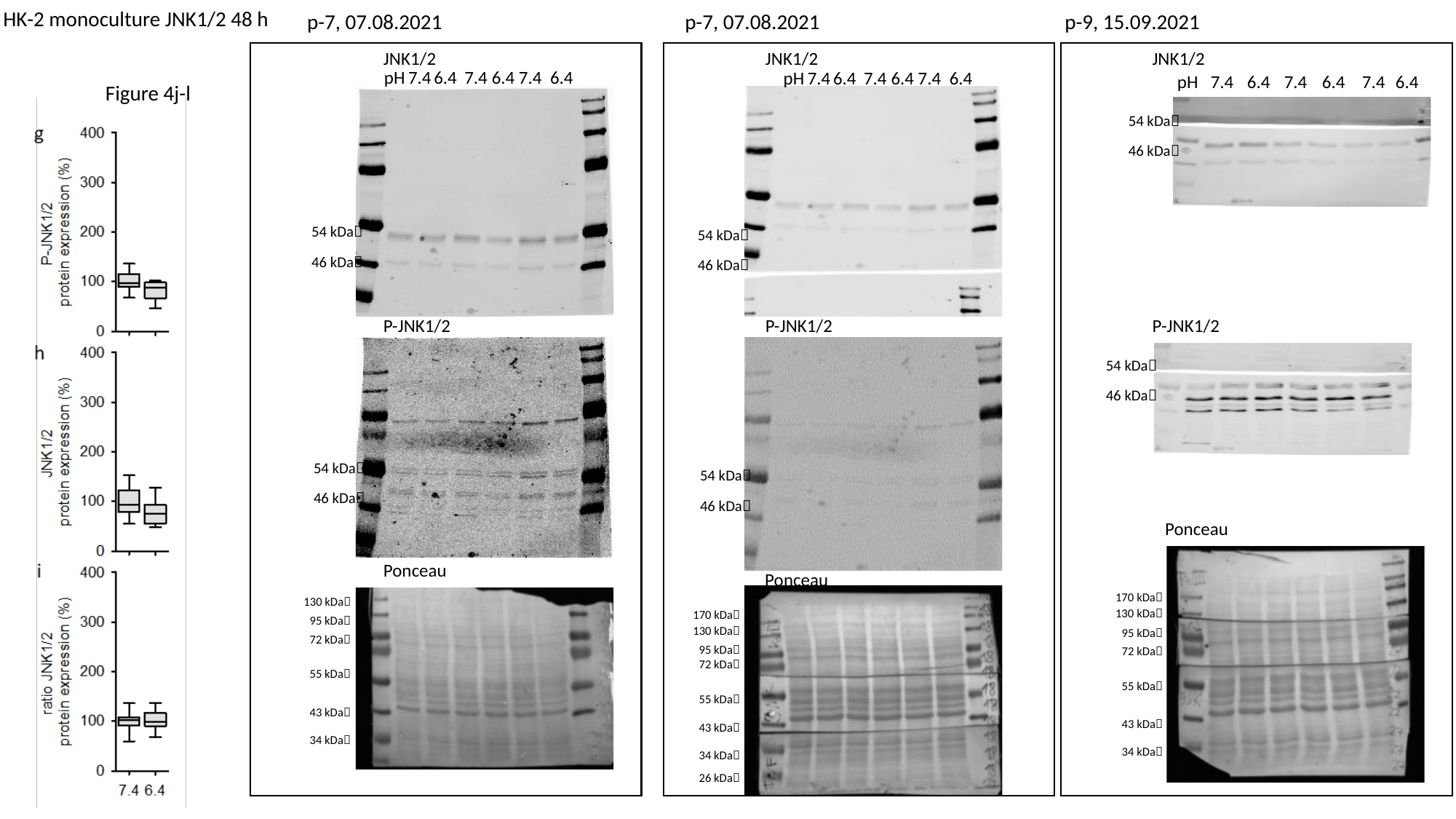

HK-2 monoculture JNK1/2 48 h
p-7, 07.08.2021
p-7, 07.08.2021
p-9, 15.09.2021
JNK1/2
JNK1/2
JNK1/2
pH
7.4
6.4
7.4
6.4
7.4
6.4
pH
7.4
6.4
7.4
6.4
7.4
6.4
pH
7.4
6.4
7.4
6.4
7.4
6.4
Figure 4j-l
54 kDa
46 kDa
54 kDa
54 kDa
46 kDa
46 kDa
P-JNK1/2
P-JNK1/2
P-JNK1/2
54 kDa
46 kDa
54 kDa
54 kDa
46 kDa
46 kDa
Ponceau
Ponceau
Ponceau
170 kDa
130 kDa
95 kDa
72 kDa
55 kDa
43 kDa
34 kDa
130 kDa
95 kDa
72 kDa
55 kDa
43 kDa
34 kDa
170 kDa
130 kDa
95 kDa
72 kDa
55 kDa
43 kDa
34 kDa
26 kDa

## Slide 45
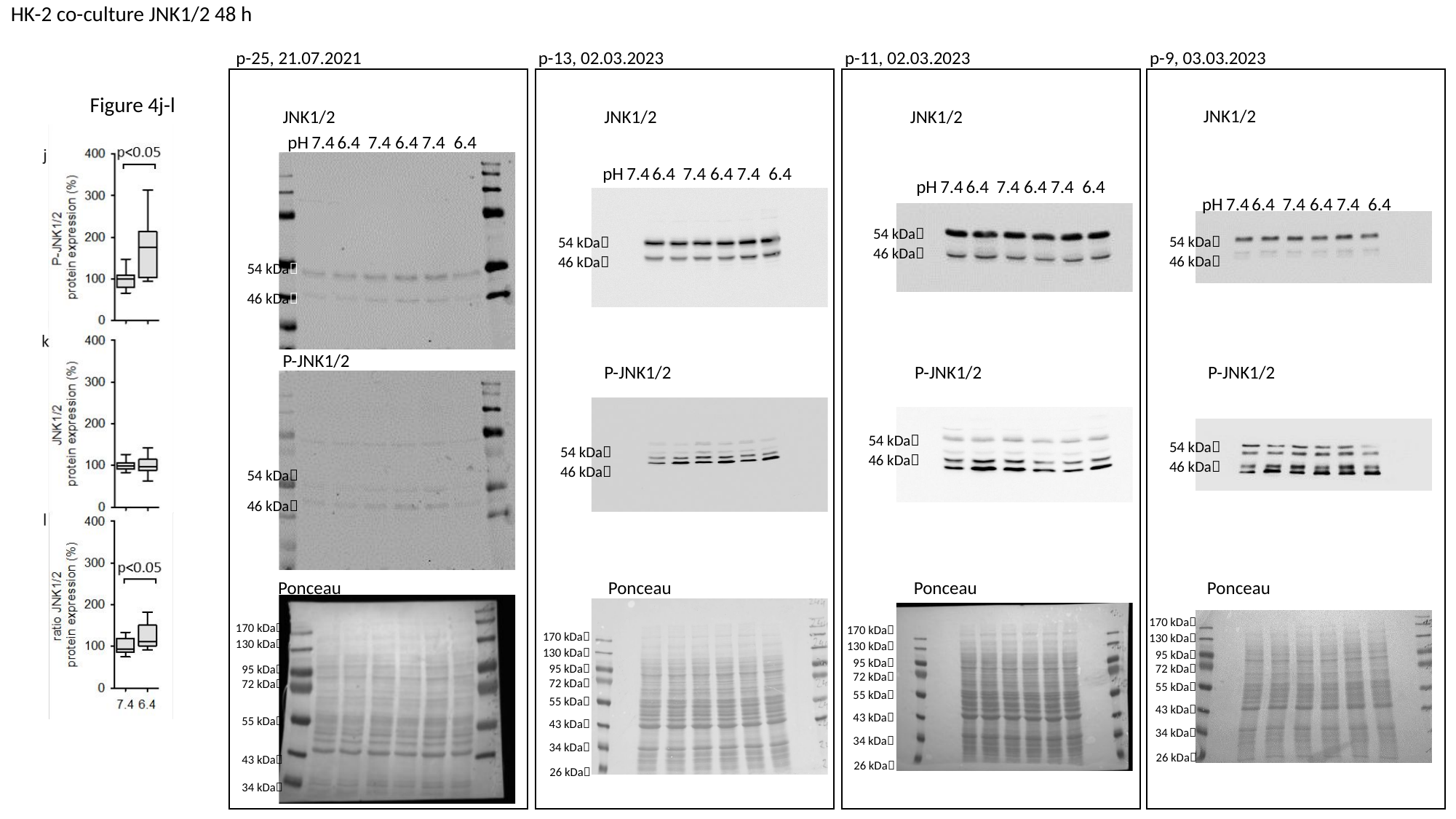

HK-2 co-culture JNK1/2 48 h
p-25, 21.07.2021
p-13, 02.03.2023
p-11, 02.03.2023
p-9, 03.03.2023
Figure 4j-l
JNK1/2
JNK1/2
JNK1/2
JNK1/2
pH
7.4
6.4
7.4
6.4
7.4
6.4
pH
7.4
6.4
7.4
6.4
7.4
6.4
pH
7.4
6.4
7.4
6.4
7.4
6.4
pH
7.4
6.4
7.4
6.4
7.4
6.4
54 kDa
54 kDa
54 kDa
46 kDa
46 kDa
46 kDa
54 kDa
46 kDa
P-JNK1/2
P-JNK1/2
P-JNK1/2
P-JNK1/2
54 kDa
54 kDa
54 kDa
46 kDa
46 kDa
46 kDa
54 kDa
46 kDa
Ponceau
Ponceau
Ponceau
Ponceau
170 kDa
130 kDa
95 kDa
72 kDa
55 kDa
43 kDa
34 kDa
26 kDa
170 kDa
130 kDa
95 kDa
72 kDa
55 kDa
43 kDa
34 kDa
170 kDa
130 kDa
95 kDa
72 kDa
55 kDa
43 kDa
34 kDa
26 kDa
170 kDa
130 kDa
95 kDa
72 kDa
55 kDa
43 kDa
34 kDa
26 kDa

## Slide 46
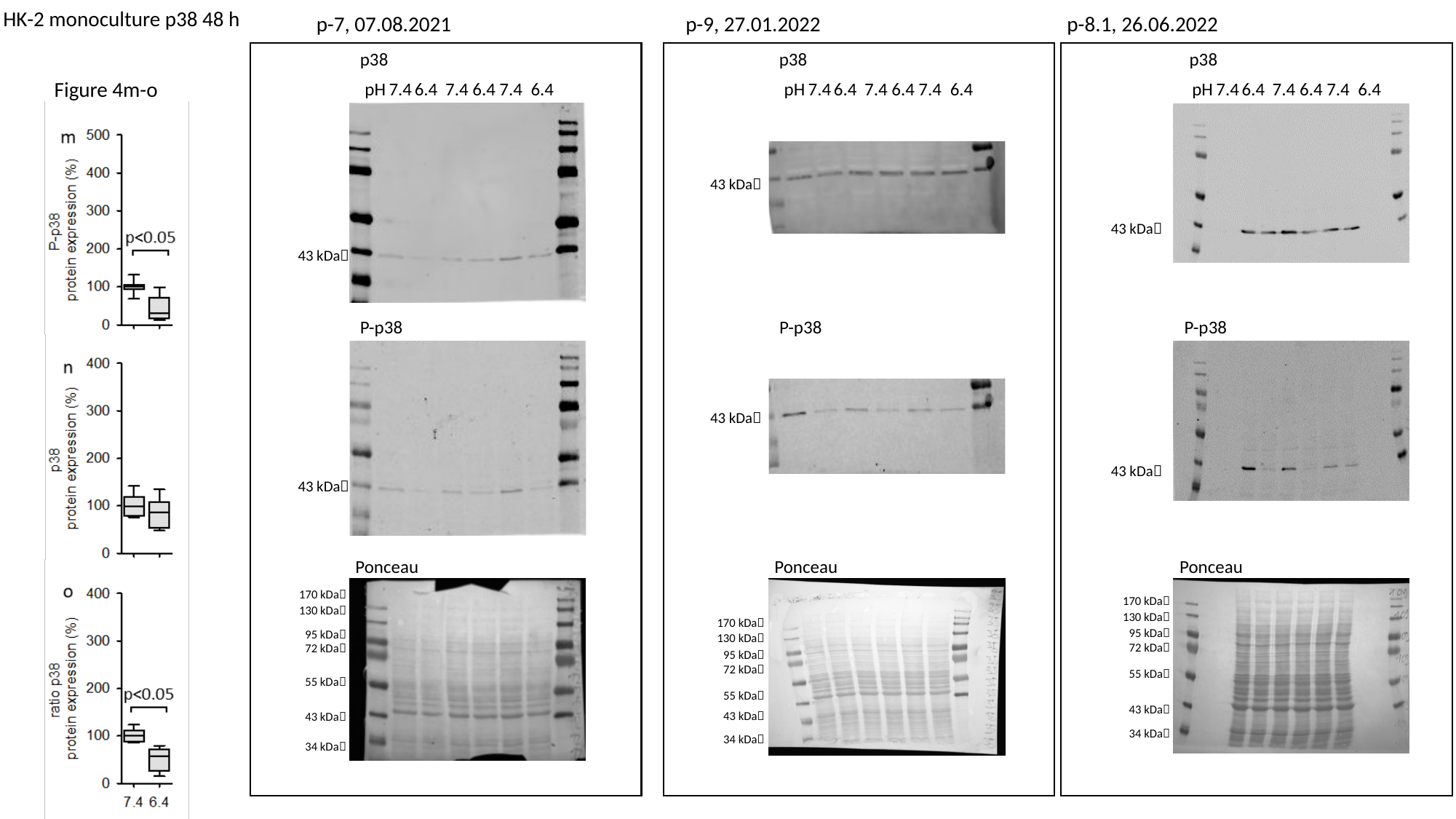

HK-2 monoculture p38 48 h
p-7, 07.08.2021
p-9, 27.01.2022
p-8.1, 26.06.2022
p38
p38
p38
Figure 4m-o
pH
7.4
6.4
7.4
6.4
7.4
6.4
pH
7.4
6.4
7.4
6.4
7.4
6.4
pH
7.4
6.4
7.4
6.4
7.4
6.4
43 kDa
43 kDa
43 kDa
P-p38
P-p38
P-p38
43 kDa
43 kDa
43 kDa
Ponceau
Ponceau
Ponceau
170 kDa
130 kDa
95 kDa
72 kDa
55 kDa
43 kDa
34 kDa
170 kDa
130 kDa
95 kDa
72 kDa
55 kDa
43 kDa
34 kDa
170 kDa
130 kDa
95 kDa
72 kDa
55 kDa
43 kDa
34 kDa

## Slide 47
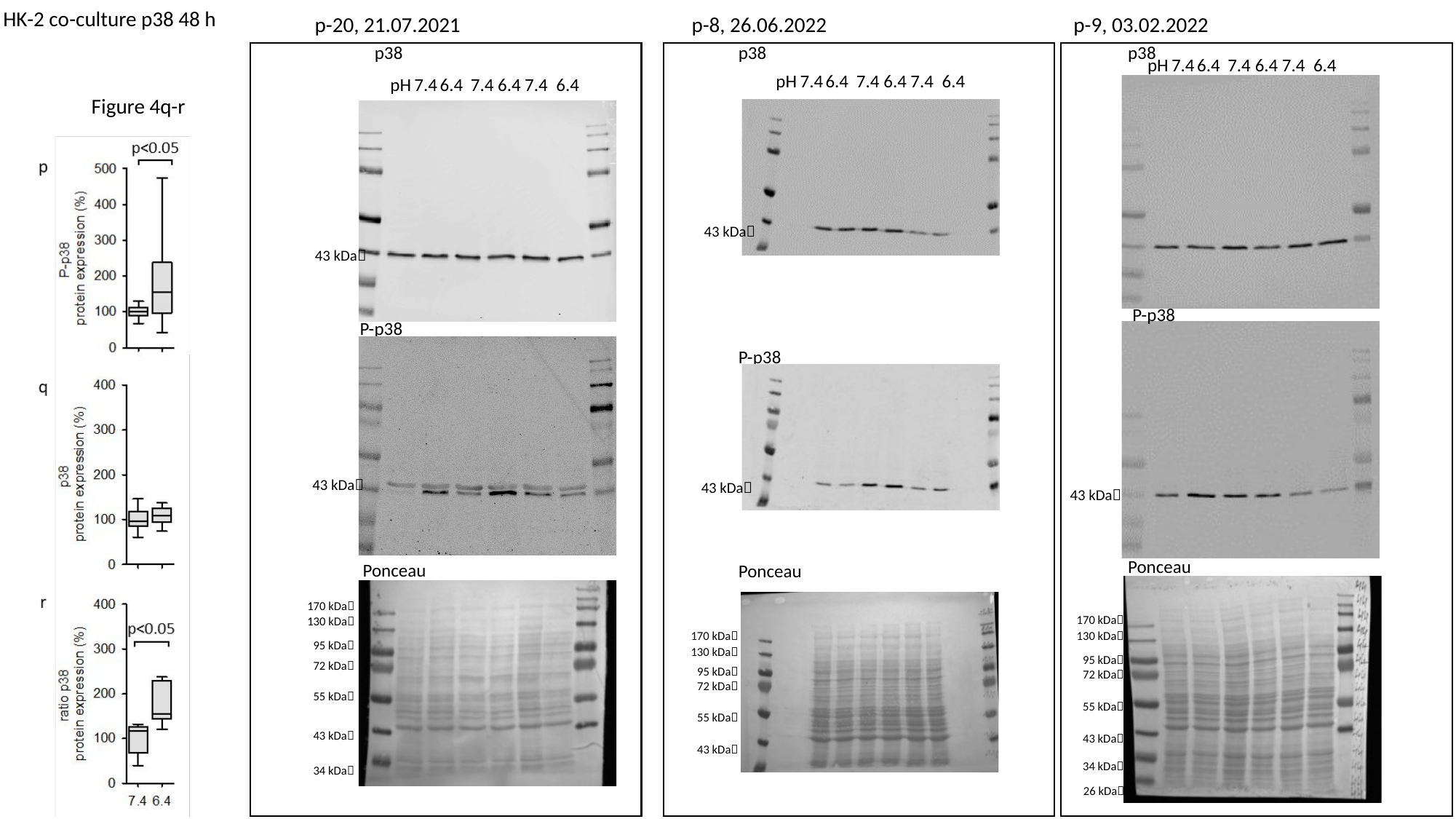

HK-2 co-culture p38 48 h
p-20, 21.07.2021
p-8, 26.06.2022
p-9, 03.02.2022
p38
p38
p38
pH
7.4
6.4
7.4
6.4
7.4
6.4
pH
7.4
6.4
7.4
6.4
7.4
6.4
pH
7.4
6.4
7.4
6.4
7.4
6.4
Figure 4q-r
43 kDa
43 kDa
43 kDa
P-p38
P-p38
P-p38
43 kDa
43 kDa
43 kDa
43 kDa
Ponceau
Ponceau
Ponceau
170 kDa
130 kDa
95 kDa
72 kDa
55 kDa
43 kDa
34 kDa
170 kDa
130 kDa
95 kDa
72 kDa
55 kDa
43 kDa
34 kDa
26 kDa
170 kDa
130 kDa
95 kDa
72 kDa
55 kDa
43 kDa

## Slide 48
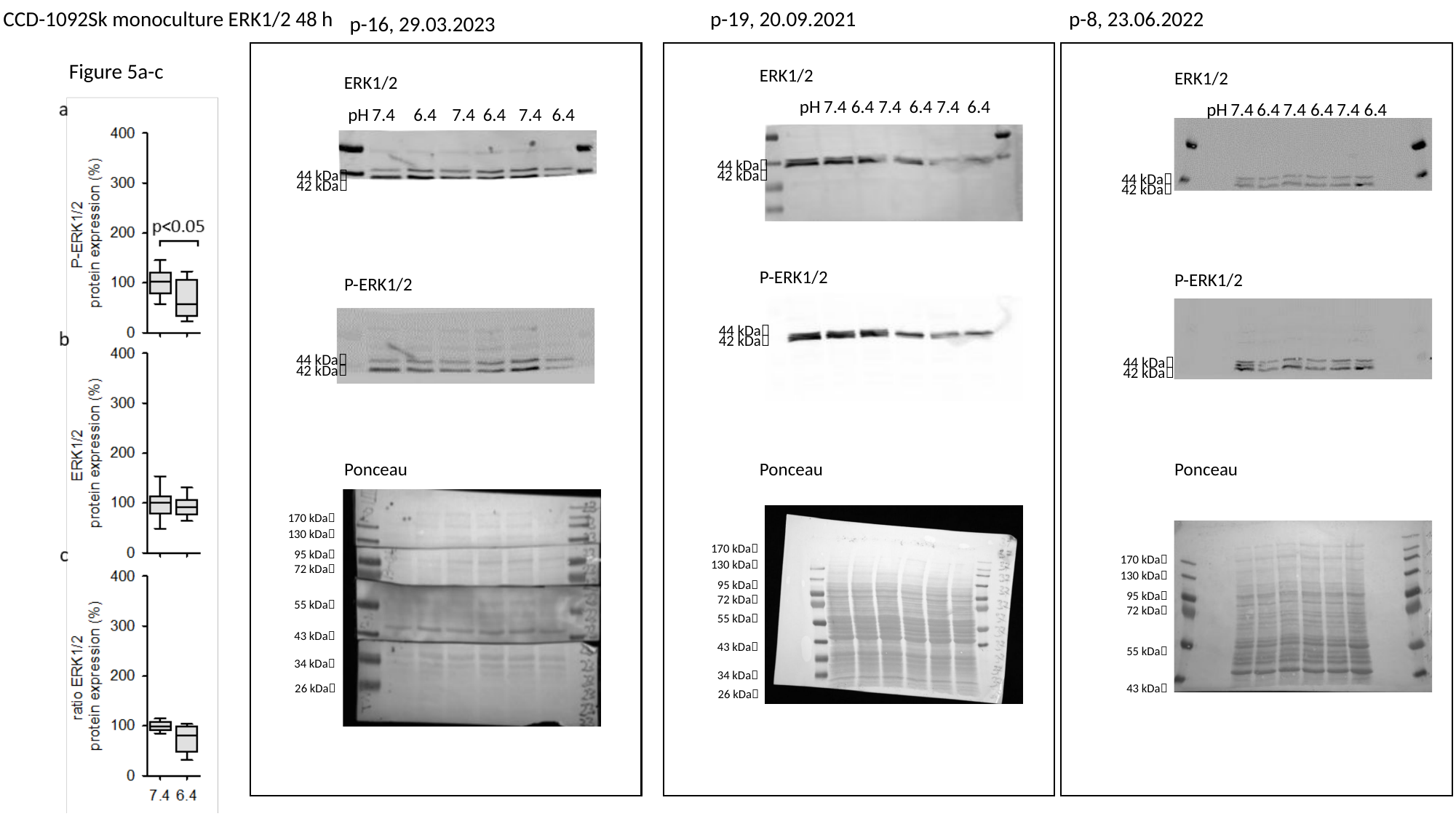

CCD-1092Sk monoculture ERK1/2 48 h
p-19, 20.09.2021
p-8, 23.06.2022
p-16, 29.03.2023
Figure 5a-c
ERK1/2
ERK1/2
ERK1/2
pH
7.4
6.4
7.4
6.4
7.4
6.4
pH
7.4
6.4
7.4
6.4
7.4
6.4
pH
7.4
6.4
7.4
6.4
7.4
6.4
44 kDa
44 kDa
42 kDa
44 kDa
42 kDa
42 kDa
P-ERK1/2
P-ERK1/2
P-ERK1/2
44 kDa
42 kDa
44 kDa
44 kDa
42 kDa
42 kDa
Ponceau
Ponceau
Ponceau
170 kDa
130 kDa
95 kDa
72 kDa
55 kDa
43 kDa
34 kDa
26 kDa
170 kDa
130 kDa
95 kDa
72 kDa
55 kDa
43 kDa
34 kDa
26 kDa
170 kDa
130 kDa
95 kDa
72 kDa
55 kDa
43 kDa

## Slide 49
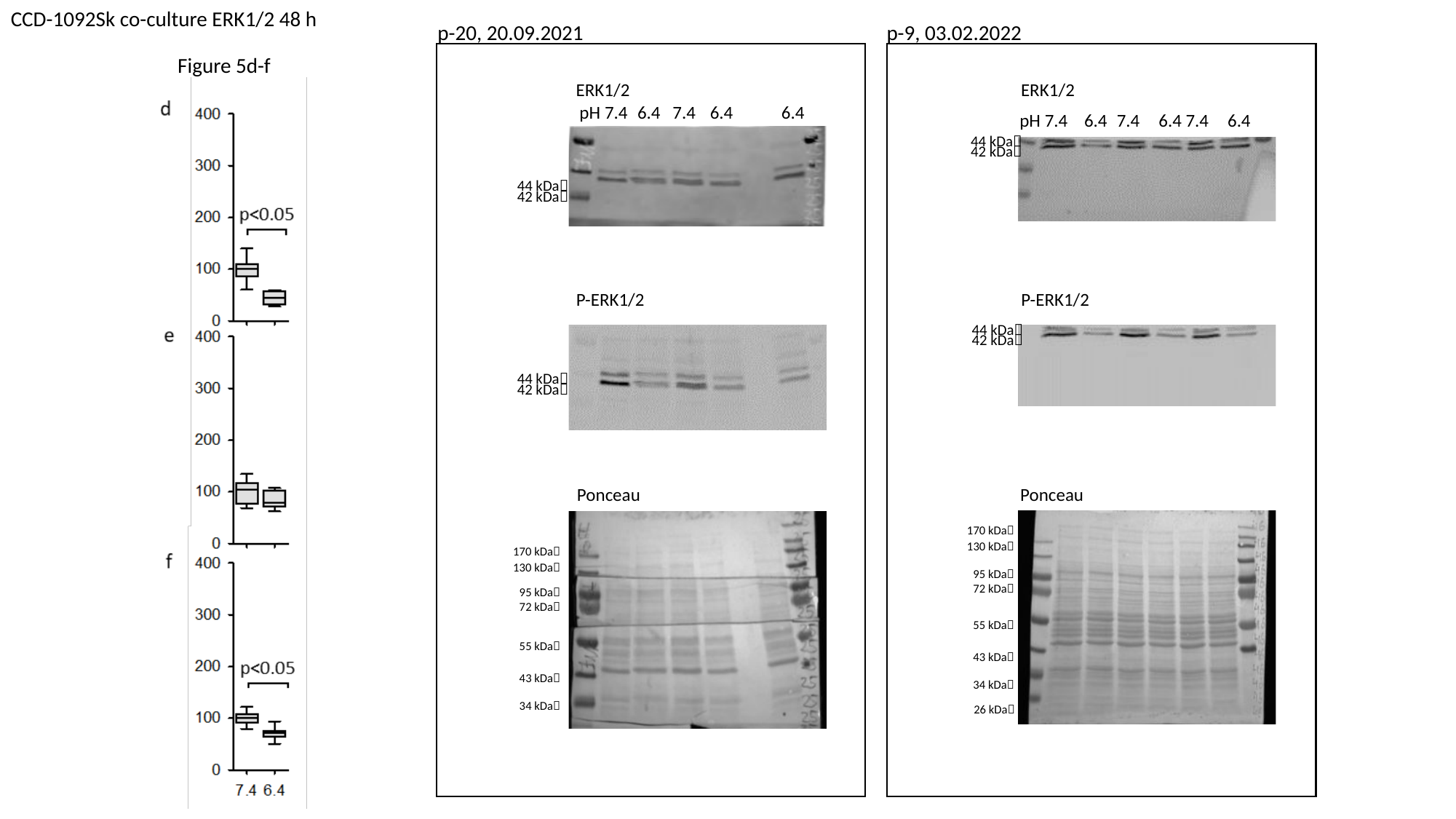

CCD-1092Sk co-culture ERK1/2 48 h
p-20, 20.09.2021
p-9, 03.02.2022
Figure 5d-f
ERK1/2
ERK1/2
pH
7.4
6.4
7.4
6.4
6.4
pH
7.4
6.4
7.4
6.4
7.4
6.4
44 kDa
42 kDa
44 kDa
42 kDa
P-ERK1/2
P-ERK1/2
44 kDa
42 kDa
44 kDa
42 kDa
Ponceau
Ponceau
170 kDa
130 kDa
95 kDa
72 kDa
55 kDa
43 kDa
34 kDa
26 kDa
170 kDa
130 kDa
95 kDa
72 kDa
55 kDa
43 kDa
34 kDa

## Slide 50
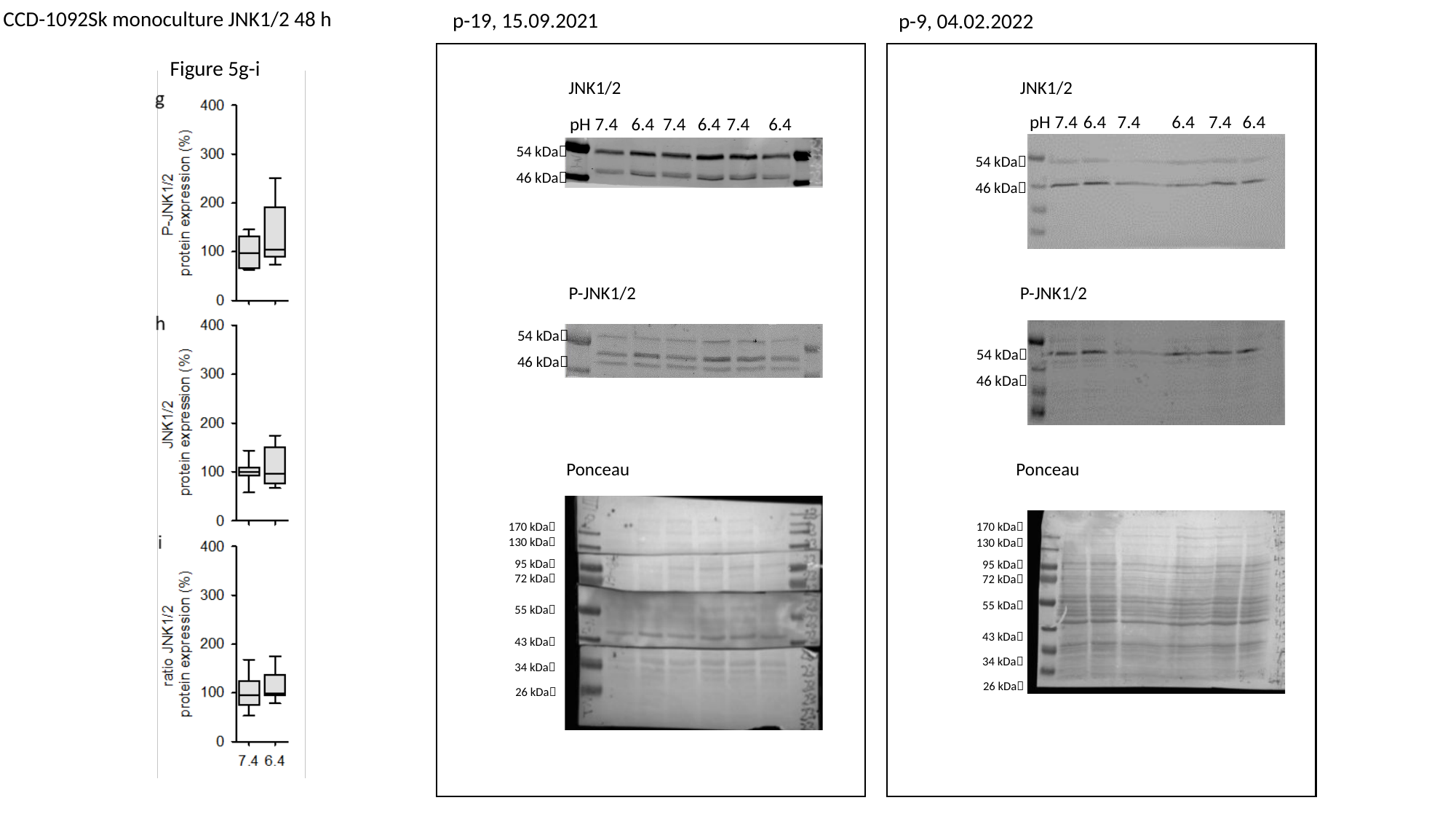

CCD-1092Sk monoculture JNK1/2 48 h
p-19, 15.09.2021
p-9, 04.02.2022
Figure 5g-i
JNK1/2
JNK1/2
pH
7.4
6.4
7.4
6.4
7.4
6.4
pH
7.4
6.4
7.4
6.4
7.4
6.4
54 kDa
54 kDa
46 kDa
46 kDa
P-JNK1/2
P-JNK1/2
54 kDa
54 kDa
46 kDa
46 kDa
Ponceau
Ponceau
170 kDa
130 kDa
95 kDa
72 kDa
55 kDa
43 kDa
34 kDa
26 kDa
170 kDa
130 kDa
95 kDa
72 kDa
55 kDa
43 kDa
34 kDa
26 kDa

## Slide 51
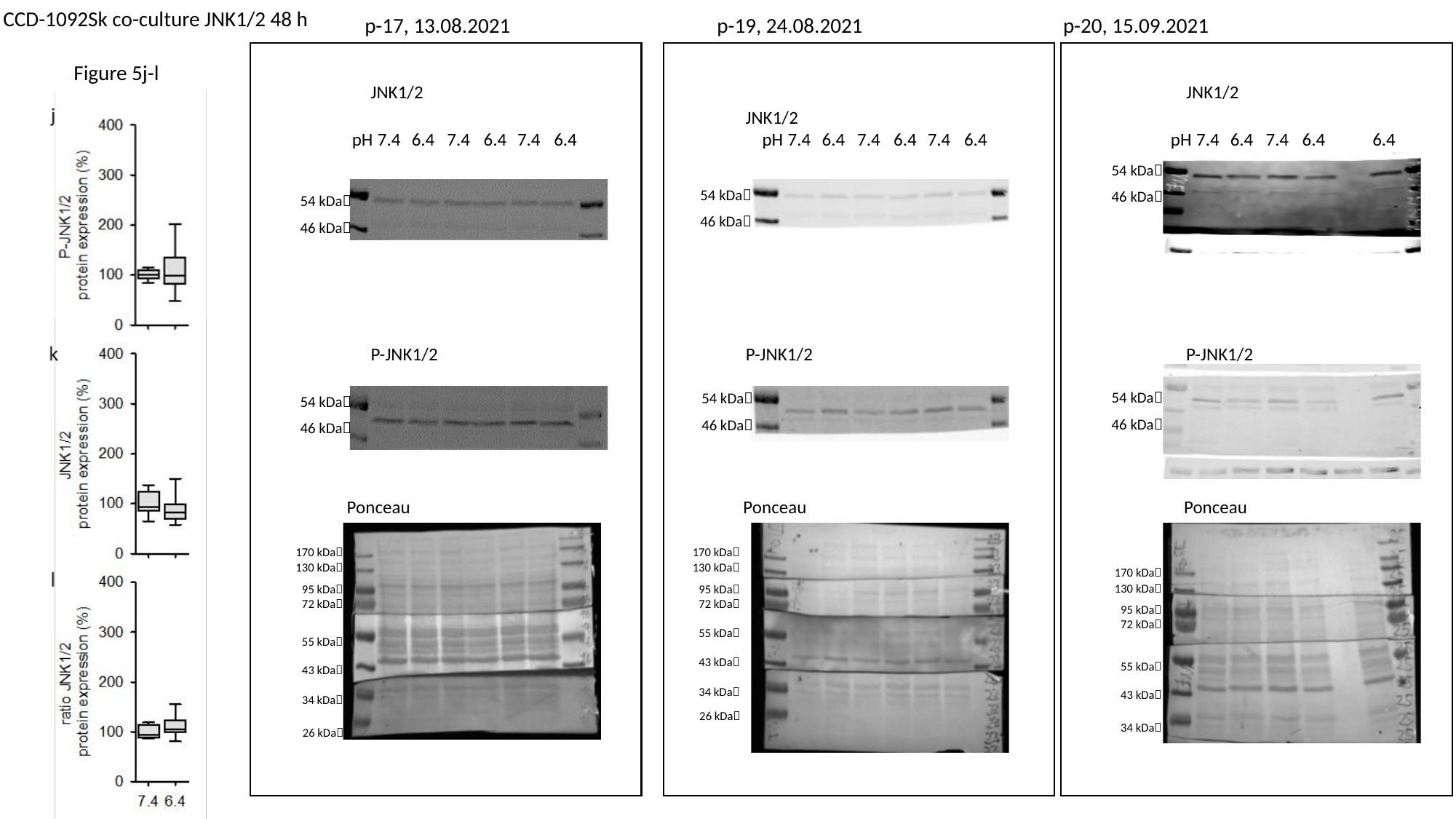

CCD-1092Sk co-culture JNK1/2 48 h
p-17, 13.08.2021
p-19, 24.08.2021
p-20, 15.09.2021
Figure 5j-l
JNK1/2
JNK1/2
JNK1/2
pH
7.4
6.4
7.4
6.4
7.4
6.4
pH
7.4
6.4
7.4
6.4
7.4
6.4
pH
7.4
6.4
7.4
6.4
6.4
54 kDa
54 kDa
46 kDa
54 kDa
46 kDa
46 kDa
P-JNK1/2
P-JNK1/2
P-JNK1/2
54 kDa
54 kDa
54 kDa
46 kDa
46 kDa
46 kDa
Ponceau
Ponceau
Ponceau
170 kDa
130 kDa
95 kDa
72 kDa
55 kDa
43 kDa
34 kDa
26 kDa
170 kDa
130 kDa
95 kDa
72 kDa
55 kDa
43 kDa
34 kDa
26 kDa
170 kDa
130 kDa
95 kDa
72 kDa
55 kDa
43 kDa
34 kDa

## Slide 52
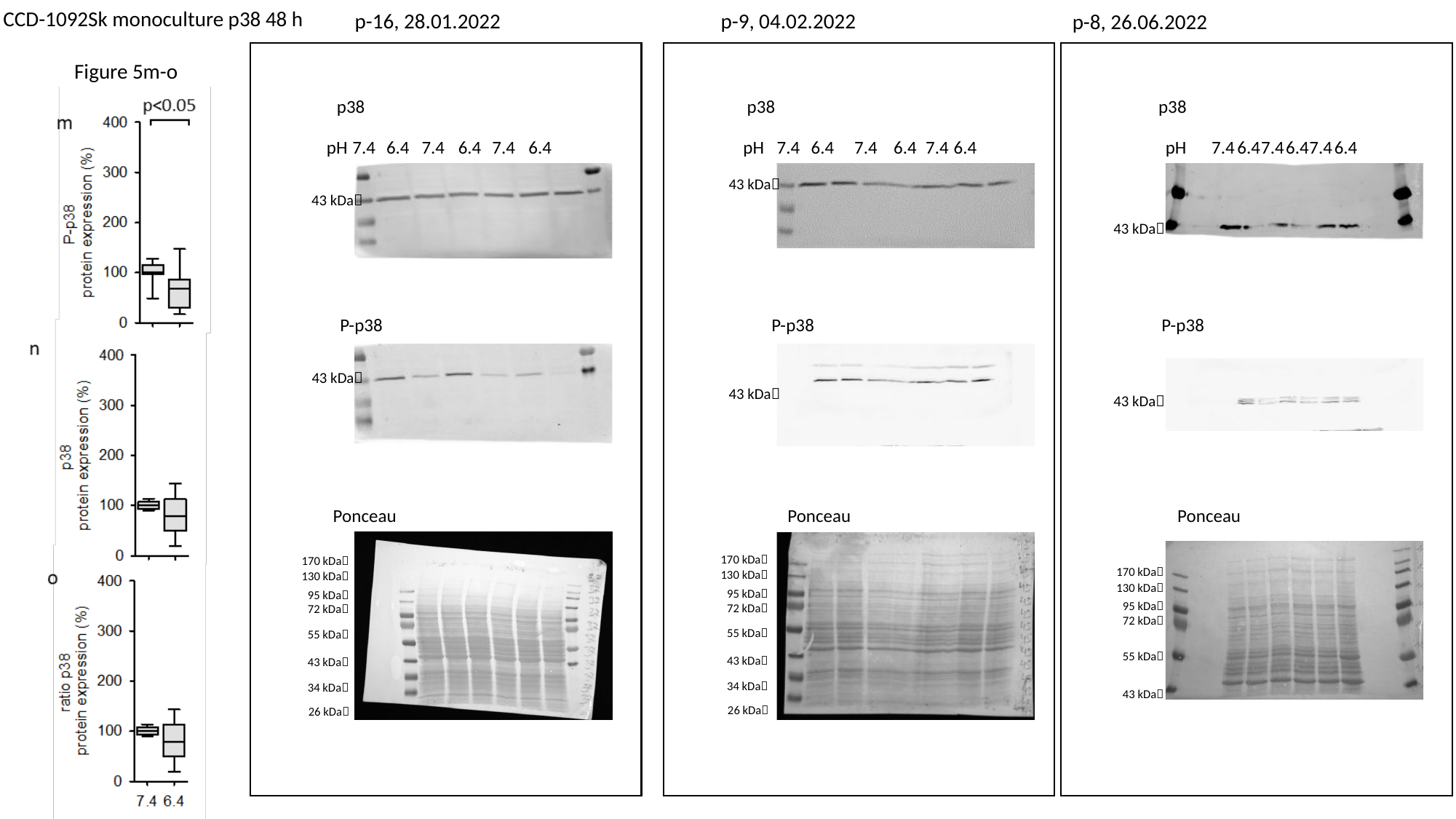

CCD-1092Sk monoculture p38 48 h
p-16, 28.01.2022
p-9, 04.02.2022
p-8, 26.06.2022
Figure 5m-o
p38
p38
p38
pH
7.4
6.4
7.4
6.4
7.4
6.4
pH
7.4
6.4
7.4
6.4
7.4
6.4
pH
7.4
6.4
7.4
6.4
7.4
6.4
43 kDa
43 kDa
43 kDa
P-p38
P-p38
P-p38
43 kDa
43 kDa
43 kDa
Ponceau
Ponceau
Ponceau
170 kDa
130 kDa
95 kDa
72 kDa
55 kDa
43 kDa
34 kDa
26 kDa
170 kDa
130 kDa
95 kDa
72 kDa
55 kDa
43 kDa
34 kDa
26 kDa
170 kDa
130 kDa
95 kDa
72 kDa
55 kDa
43 kDa

## Slide 53
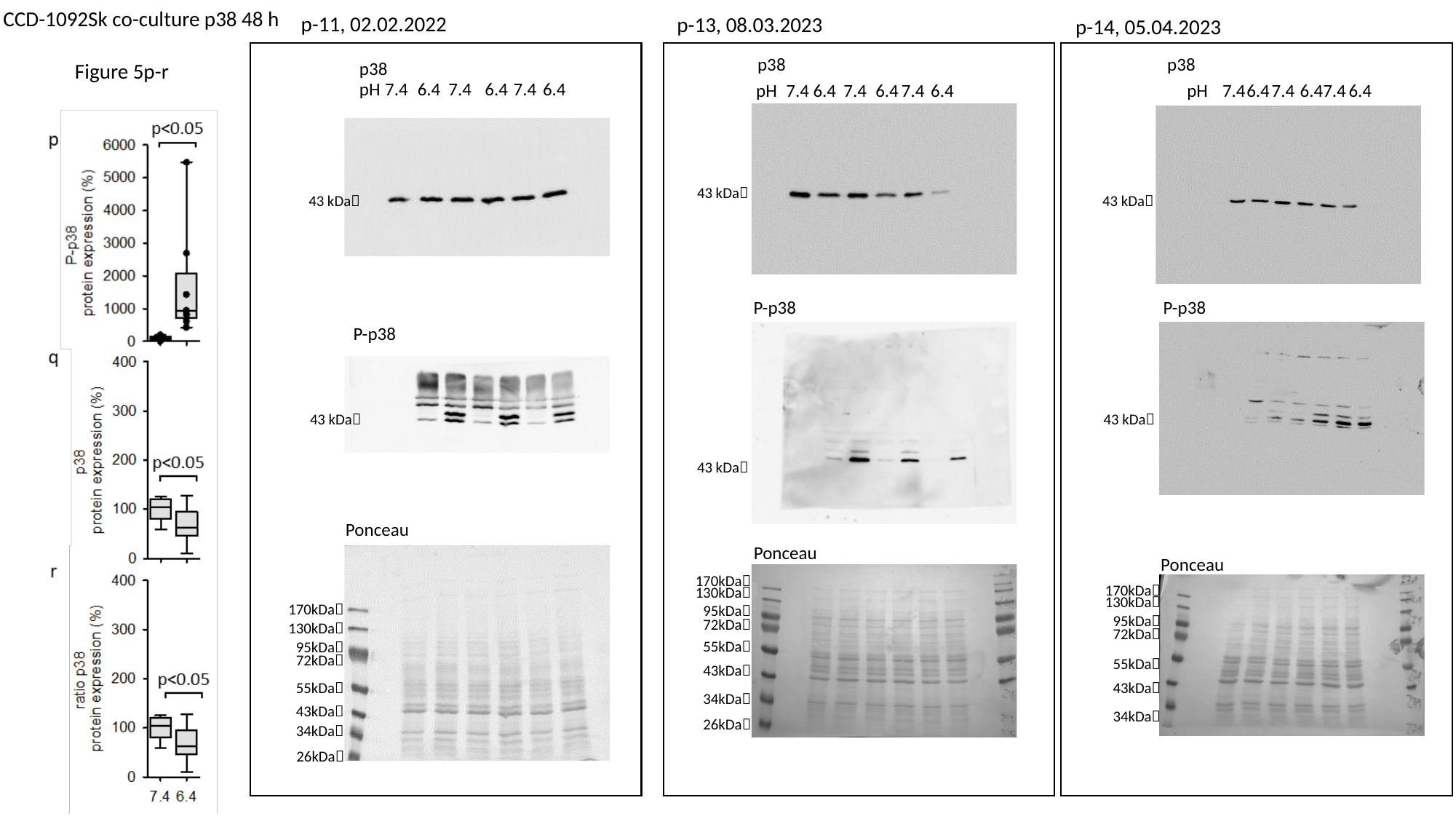

CCD-1092Sk co-culture p38 48 h
p-11, 02.02.2022
p-13, 08.03.2023
p-14, 05.04.2023
p38
p38
p38
Figure 5p-r
pH
7.4
6.4
7.4
6.4
7.4
6.4
pH
7.4
6.4
7.4
6.4
7.4
6.4
pH
7.4
6.4
7.4
6.4
7.4
6.4
43 kDa
43 kDa
43 kDa
P-p38
P-p38
P-p38
43 kDa
43 kDa
43 kDa
Ponceau
Ponceau
Ponceau
170kDa
130kDa
95kDa
72kDa
55kDa
43kDa
34kDa
26kDa
170kDa
130kDa
95kDa
72kDa
55kDa
43kDa
34kDa
170kDa
130kDa
95kDa
72kDa
55kDa
43kDa
34kDa
26kDa

## Slide 54
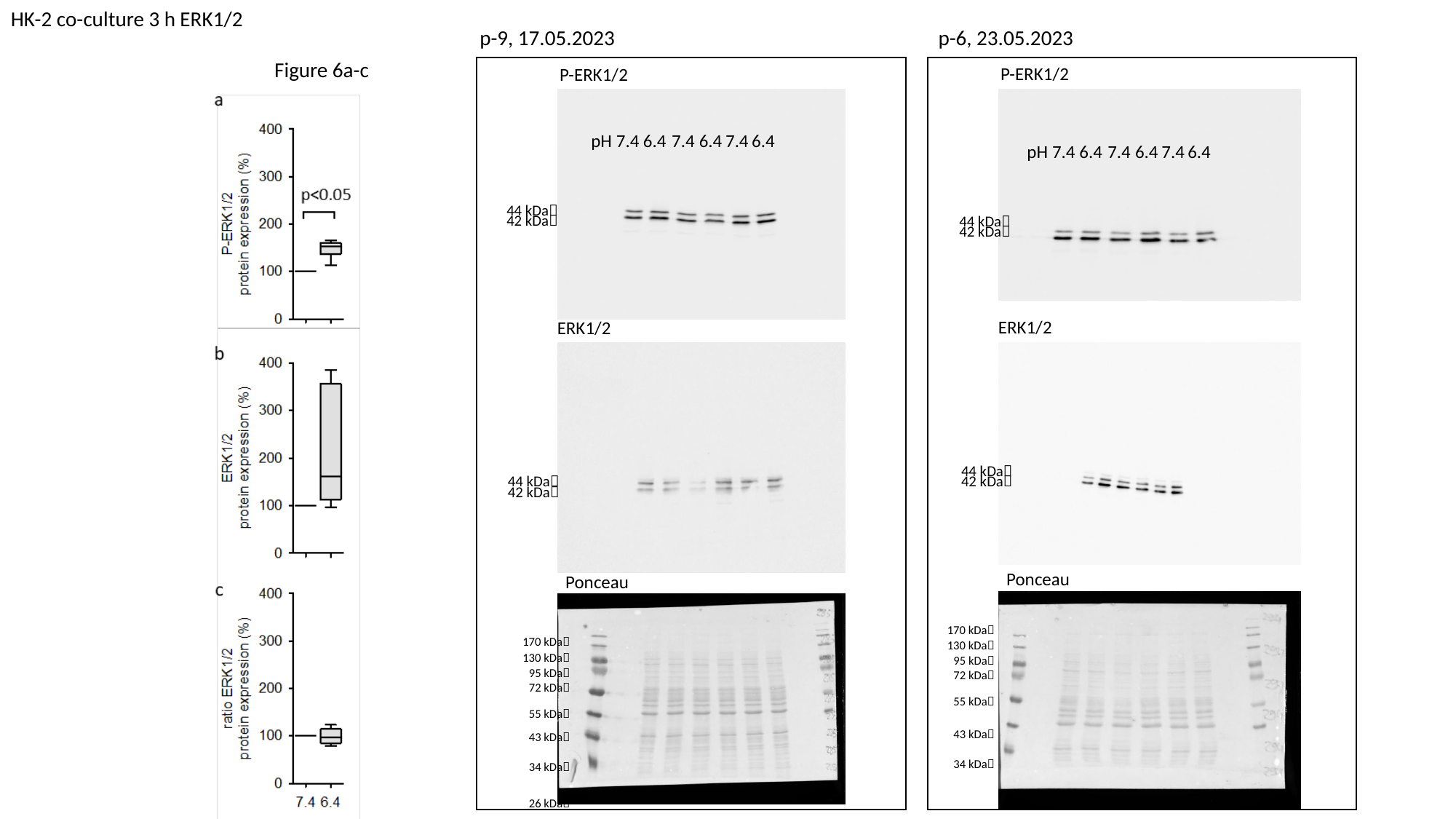

HK-2 co-culture 3 h ERK1/2
p-6, 23.05.2023
p-9, 17.05.2023
Figure 6a-c
P-ERK1/2
P-ERK1/2
pH
7.4
6.4
7.4
6.4
7.4
6.4
pH
7.4
6.4
7.4
6.4
7.4
6.4
44 kDa
42 kDa
44 kDa
42 kDa
ERK1/2
ERK1/2
44 kDa
44 kDa
42 kDa
42 kDa
Ponceau
Ponceau
170 kDa
130 kDa
95 kDa
72 kDa
55 kDa
43 kDa
34 kDa
170 kDa
130 kDa
95 kDa
72 kDa
55 kDa
43 kDa
34 kDa
26 kDa

## Slide 55
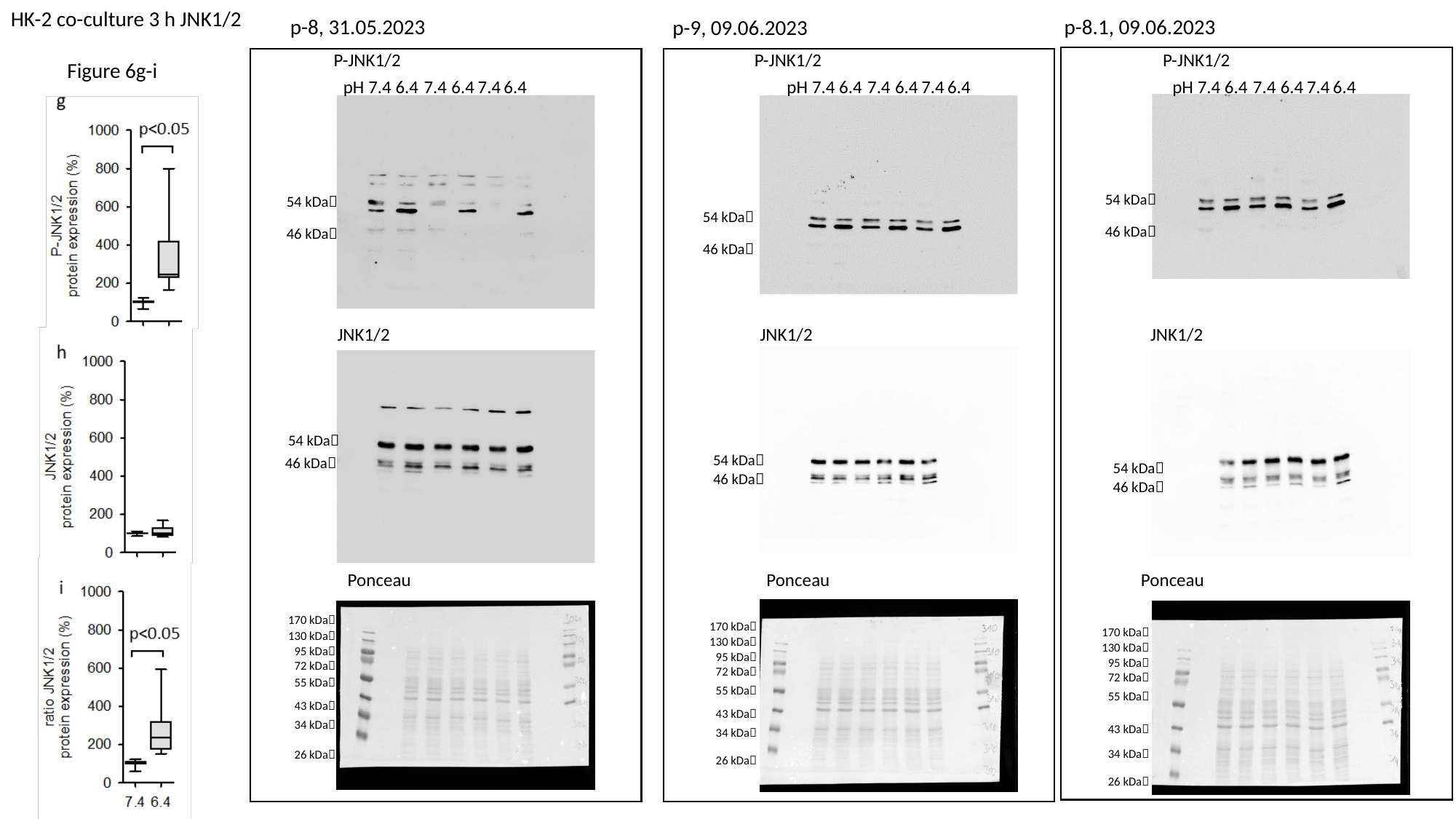

HK-2 co-culture 3 h JNK1/2
p-8, 31.05.2023
p-8.1, 09.06.2023
p-9, 09.06.2023
P-JNK1/2
P-JNK1/2
P-JNK1/2
Figure 6g-i
pH
7.4
6.4
7.4
6.4
7.4
6.4
pH
7.4
6.4
7.4
6.4
7.4
6.4
pH
7.4
6.4
7.4
6.4
7.4
6.4
54 kDa
54 kDa
54 kDa
46 kDa
46 kDa
46 kDa
JNK1/2
JNK1/2
JNK1/2
54 kDa
54 kDa
46 kDa
54 kDa
46 kDa
46 kDa
Ponceau
Ponceau
Ponceau
170 kDa
130 kDa
95 kDa
72 kDa
55 kDa
43 kDa
34 kDa
26 kDa
170 kDa
130 kDa
95 kDa
72 kDa
55 kDa
43 kDa
34 kDa
26 kDa
170 kDa
130 kDa
95 kDa
72 kDa
55 kDa
43 kDa
34 kDa
26 kDa

## Slide 56
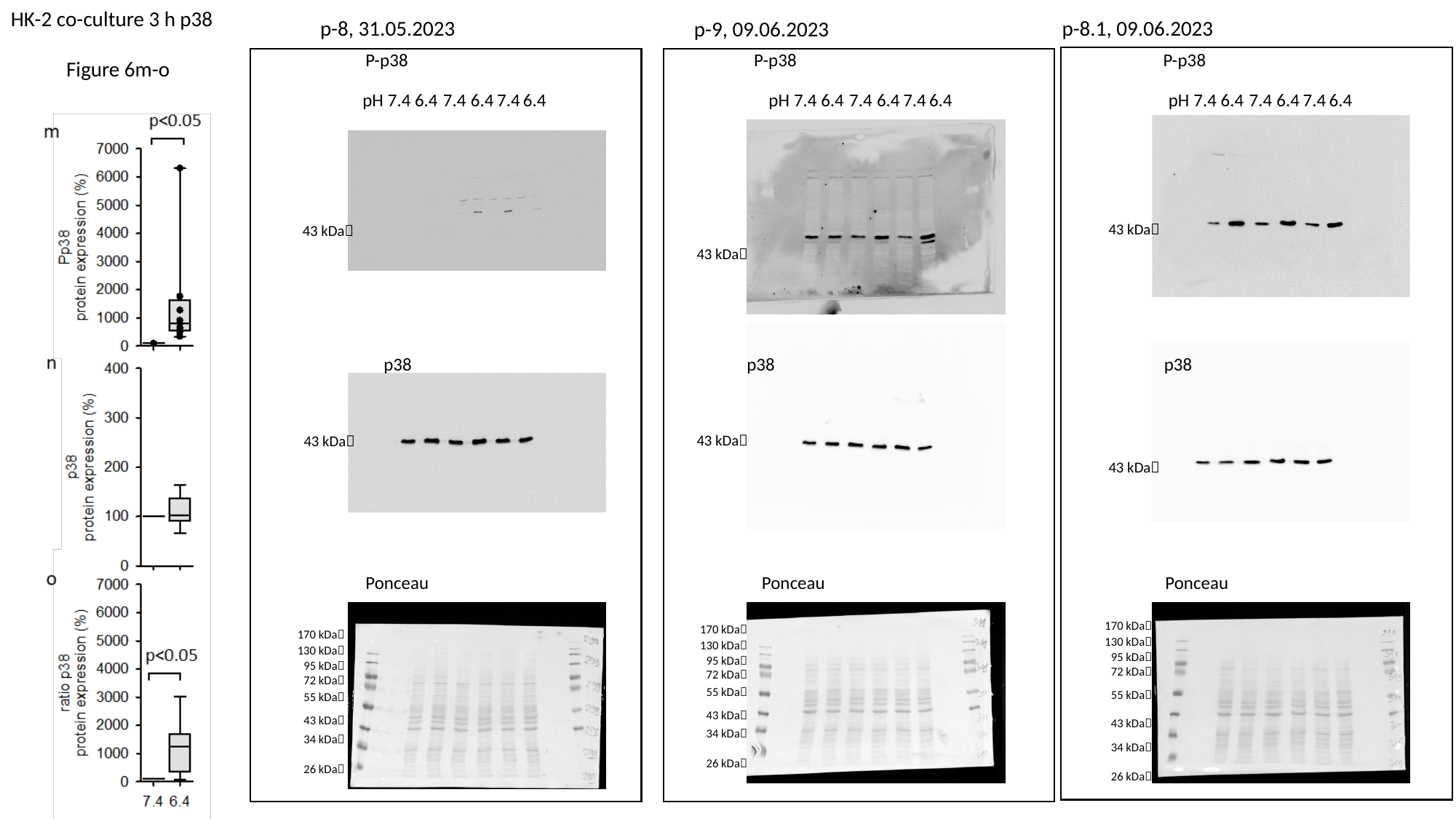

HK-2 co-culture 3 h p38
p-8, 31.05.2023
p-8.1, 09.06.2023
p-9, 09.06.2023
P-p38
P-p38
P-p38
Figure 6m-o
pH
7.4
6.4
7.4
6.4
7.4
6.4
pH
7.4
6.4
7.4
6.4
7.4
6.4
pH
7.4
6.4
7.4
6.4
7.4
6.4
43 kDa
43 kDa
43 kDa
p38
p38
p38
43 kDa
43 kDa
43 kDa
Ponceau
Ponceau
Ponceau
170 kDa
130 kDa
95 kDa
72 kDa
55 kDa
43 kDa
34 kDa
26 kDa
170 kDa
130 kDa
95 kDa
72 kDa
55 kDa
43 kDa
34 kDa
26 kDa
170 kDa
130 kDa
95 kDa
72 kDa
55 kDa
43 kDa
34 kDa
26 kDa

## Slide 57
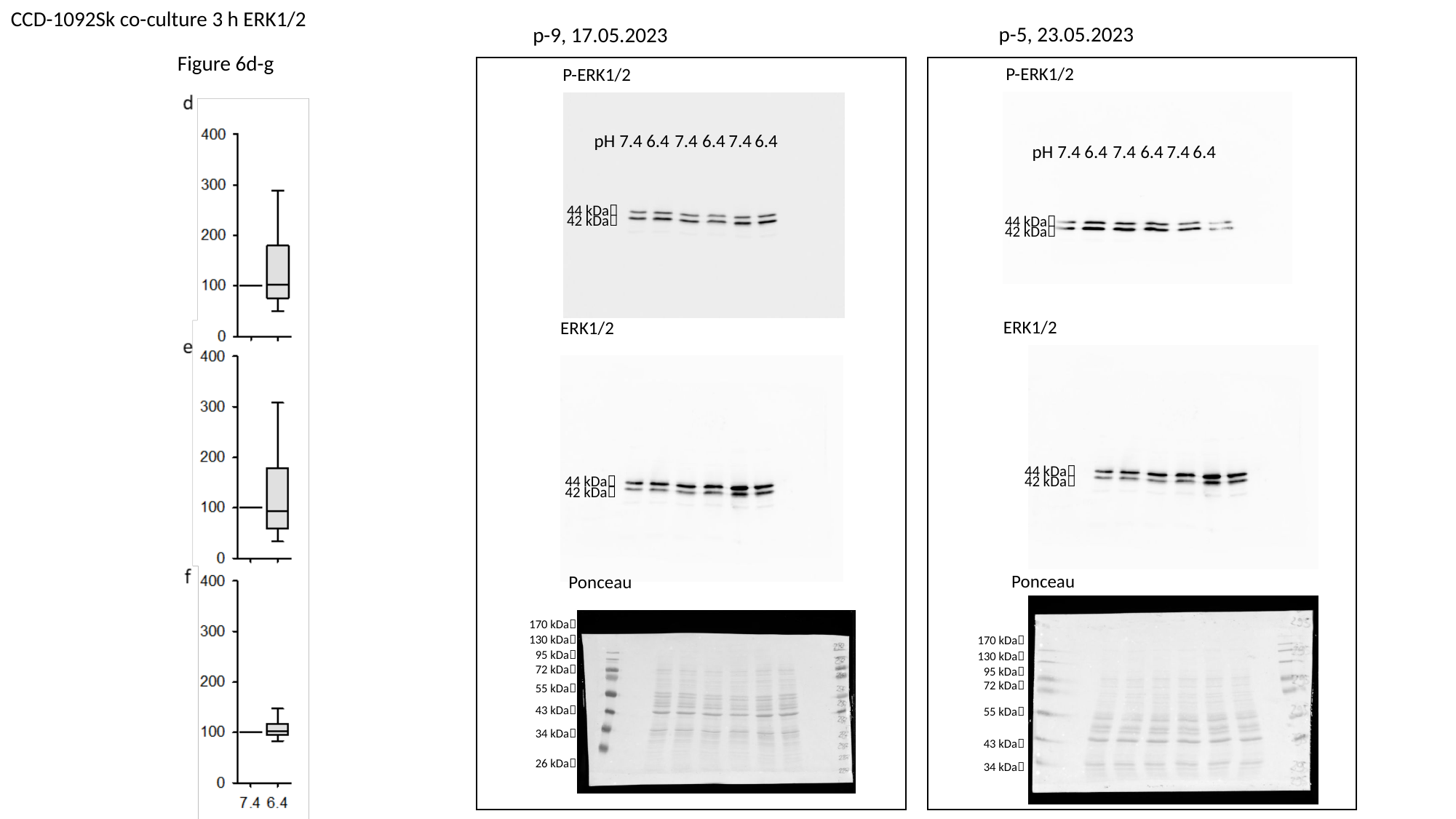

CCD-1092Sk co-culture 3 h ERK1/2
p-5, 23.05.2023
p-9, 17.05.2023
Figure 6d-g
P-ERK1/2
P-ERK1/2
pH
7.4
6.4
7.4
6.4
7.4
6.4
pH
7.4
6.4
7.4
6.4
7.4
6.4
44 kDa
42 kDa
44 kDa
42 kDa
ERK1/2
ERK1/2
44 kDa
44 kDa
42 kDa
42 kDa
Ponceau
Ponceau
170 kDa
130 kDa
95 kDa
72 kDa
55 kDa
43 kDa
34 kDa
26 kDa
170 kDa
130 kDa
95 kDa
72 kDa
55 kDa
43 kDa
34 kDa

## Slide 58
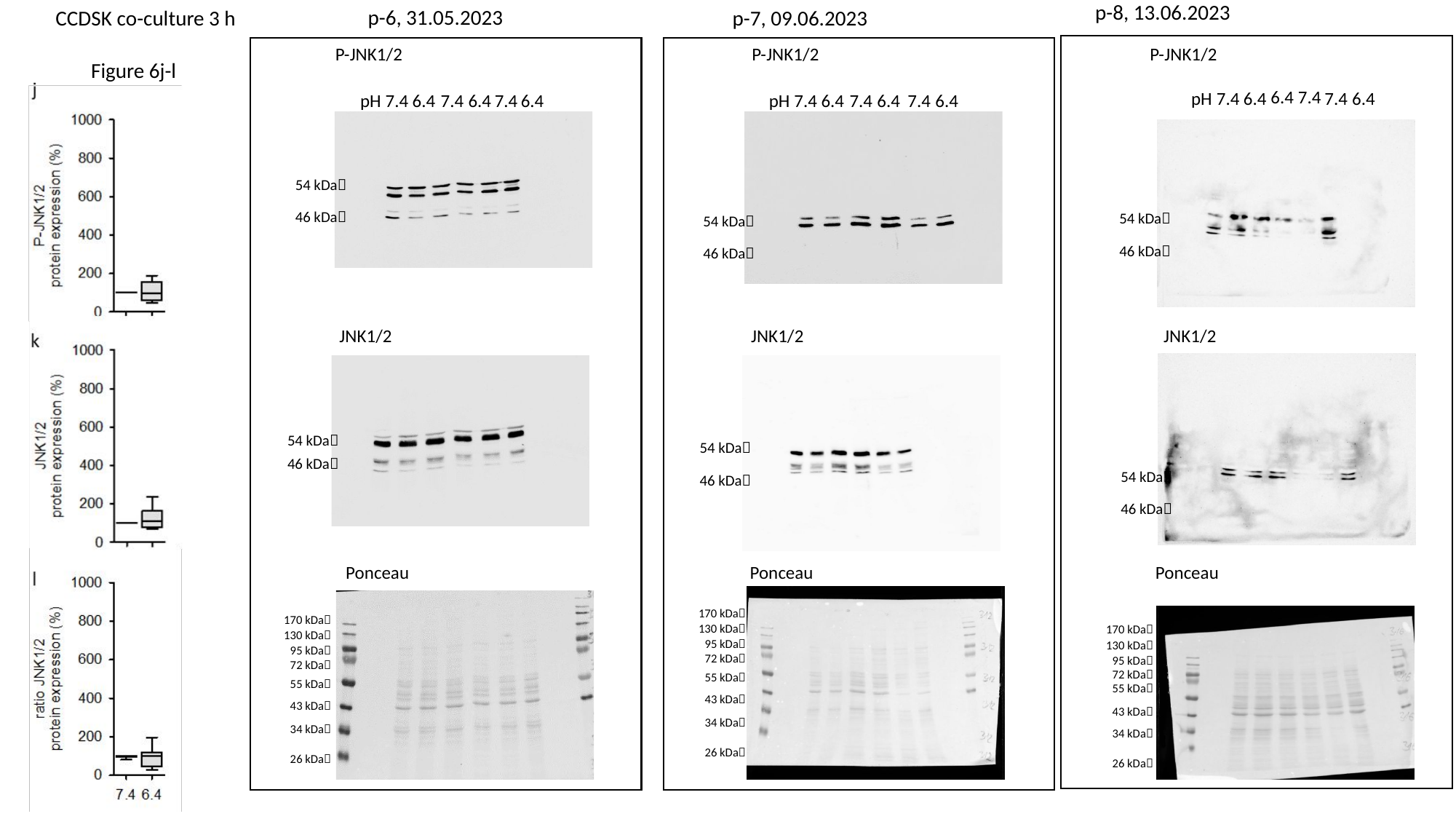

p-8, 13.06.2023
p-6, 31.05.2023
p-7, 09.06.2023
CCDSK co-culture 3 h
P-JNK1/2
P-JNK1/2
P-JNK1/2
Figure 6j-l
7.4
6.4
pH
7.4
6.4
7.4
6.4
pH
7.4
6.4
7.4
6.4
7.4
6.4
pH
7.4
6.4
7.4
6.4
7.4
6.4
54 kDa
46 kDa
54 kDa
54 kDa
46 kDa
46 kDa
JNK1/2
JNK1/2
JNK1/2
54 kDa
54 kDa
46 kDa
54 kDa
46 kDa
46 kDa
Ponceau
Ponceau
Ponceau
170 kDa
130 kDa
95 kDa
72 kDa
55 kDa
43 kDa
34 kDa
26 kDa
170 kDa
130 kDa
95 kDa
72 kDa
55 kDa
43 kDa
34 kDa
26 kDa
170 kDa
130 kDa
95 kDa
72 kDa
55 kDa
43 kDa
34 kDa
26 kDa

## Slide 59
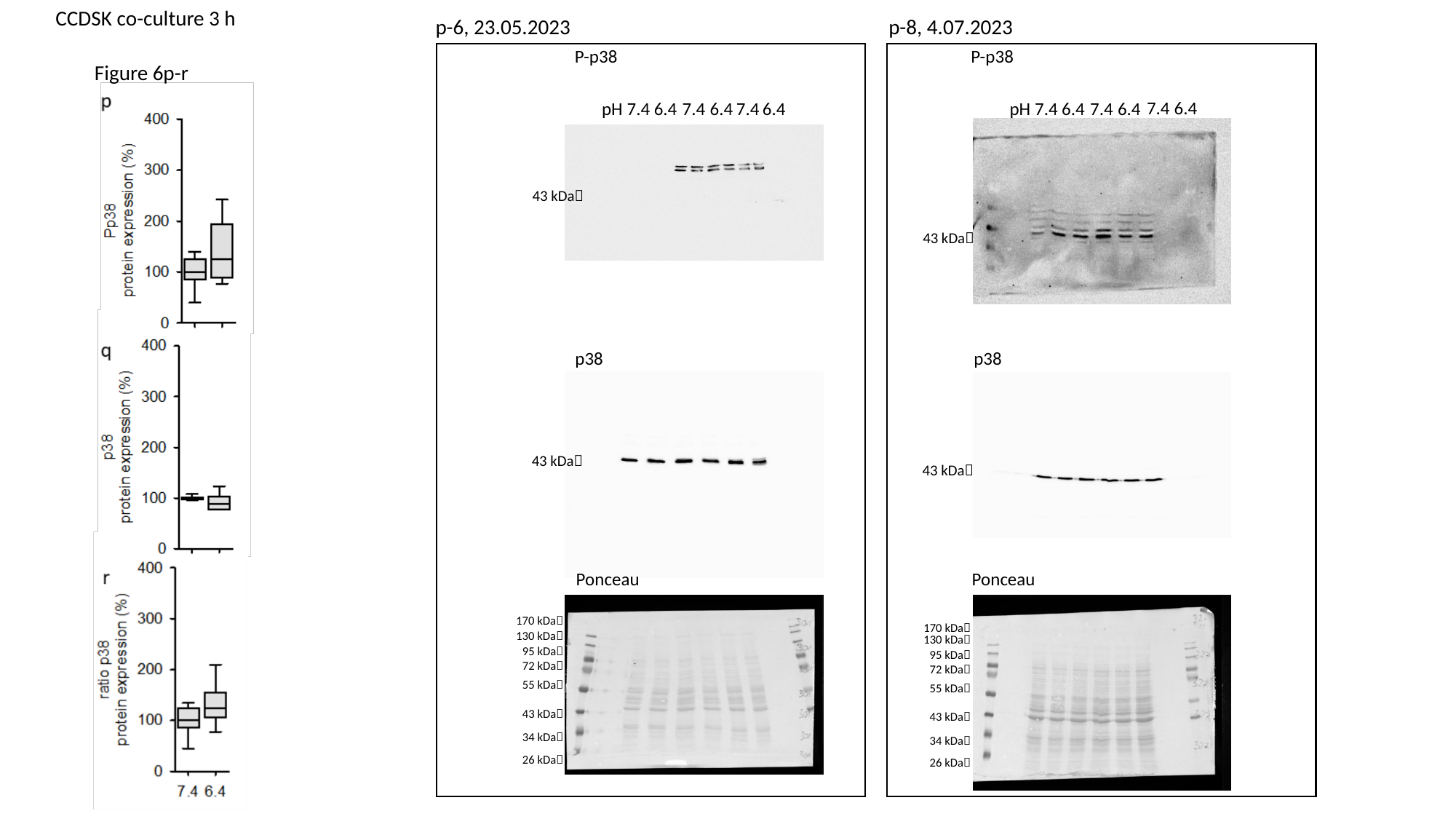

CCDSK co-culture 3 h
p-6, 23.05.2023
p-8, 4.07.2023
P-p38
P-p38
Figure 6p-r
7.4
6.4
pH
7.4
6.4
7.4
6.4
pH
7.4
6.4
7.4
6.4
7.4
6.4
43 kDa
43 kDa
p38
p38
43 kDa
43 kDa
Ponceau
Ponceau
170 kDa
130 kDa
95 kDa
72 kDa
55 kDa
43 kDa
34 kDa
26 kDa
170 kDa
130 kDa
95 kDa
72 kDa
55 kDa
43 kDa
34 kDa
26 kDa

## Slide 60
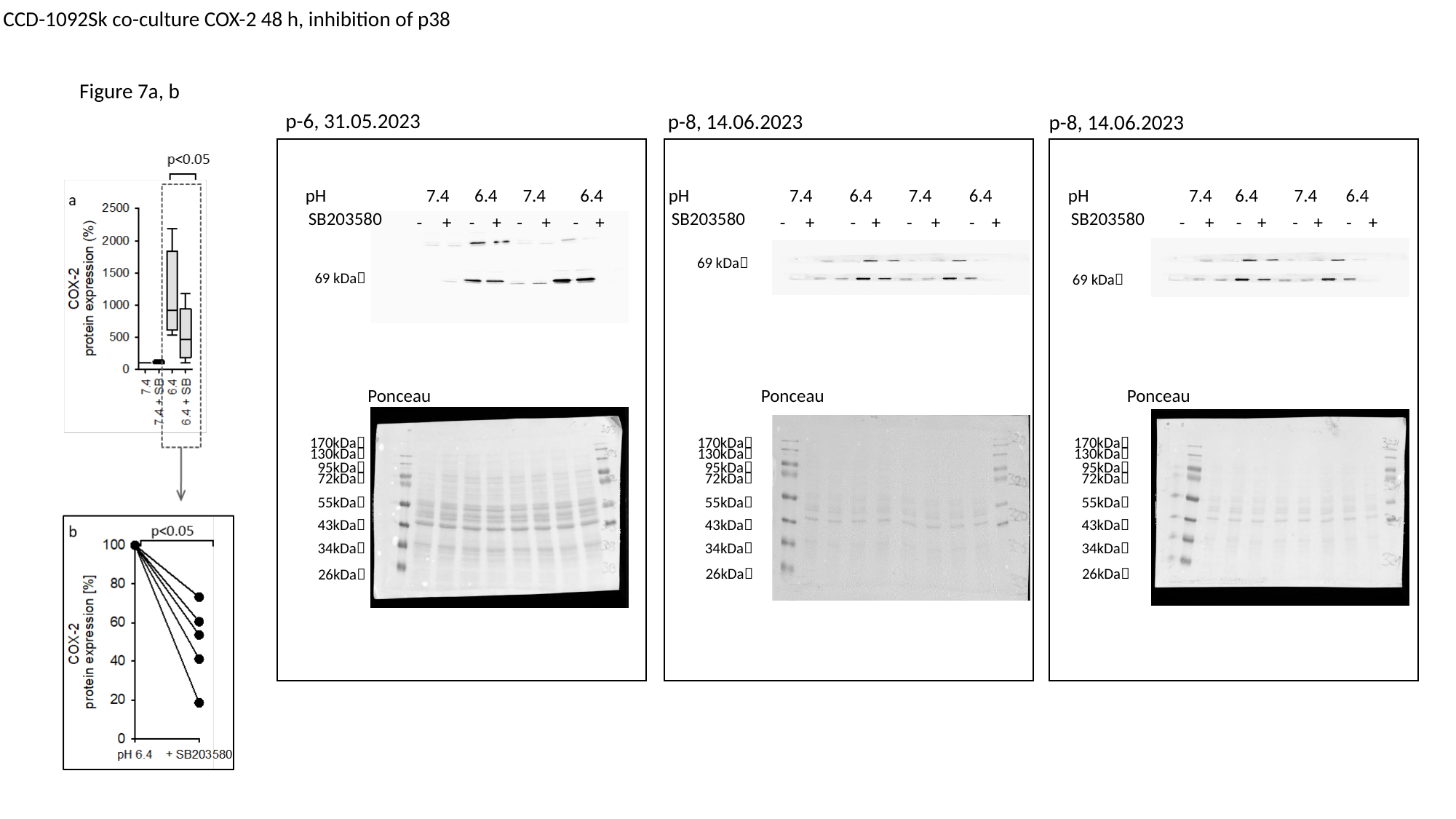

CCD-1092Sk co-culture COX-2 48 h, inhibition of p38
Figure 7a, b
p-6, 31.05.2023
p-8, 14.06.2023
p-8, 14.06.2023
pH
7.4
6.4
7.4
6.4
SB203580
+
-
+
-
+
-
+
-
pH
7.4
6.4
7.4
6.4
SB203580
+
-
+
-
+
-
+
-
pH
7.4
6.4
7.4
6.4
SB203580
+
-
+
-
+
-
+
-
69 kDa
69 kDa
69 kDa
Ponceau
Ponceau
Ponceau
170kDa
130kDa
95kDa
72kDa
55kDa
43kDa
34kDa
26kDa
170kDa
130kDa
95kDa
72kDa
55kDa
43kDa
34kDa
26kDa
170kDa
130kDa
95kDa
72kDa
55kDa
43kDa
34kDa
26kDa

## Slide 61
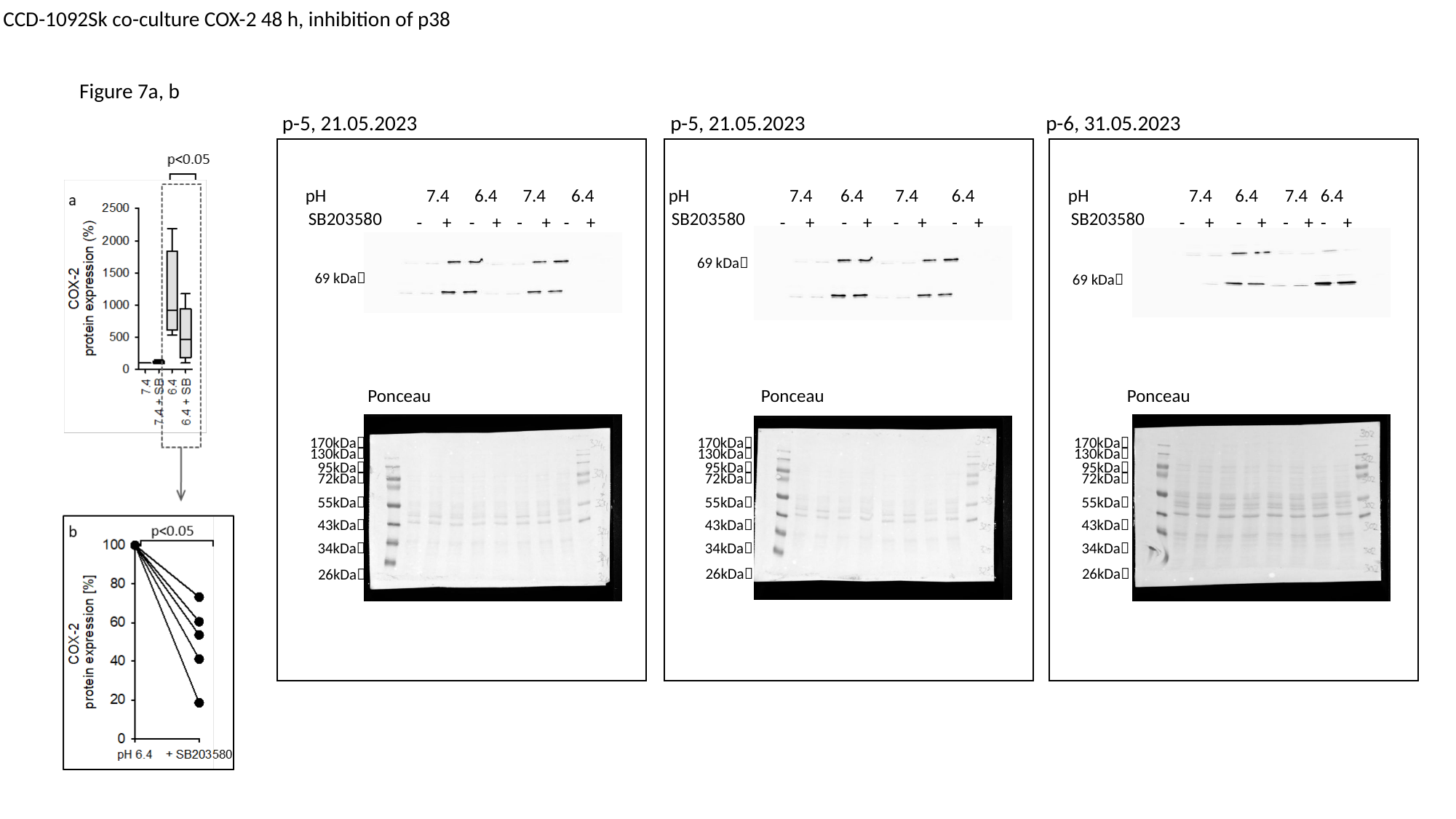

CCD-1092Sk co-culture COX-2 48 h, inhibition of p38
Figure 7a, b
p-6, 31.05.2023
p-5, 21.05.2023
p-5, 21.05.2023
pH
7.4
6.4
7.4
6.4
SB203580
+
-
+
-
+
-
+
-
pH
7.4
6.4
7.4
6.4
SB203580
+
-
+
-
+
-
+
-
pH
7.4
6.4
7.4
6.4
SB203580
+
-
+
-
+
-
+
-
69 kDa
69 kDa
69 kDa
Ponceau
Ponceau
Ponceau
170kDa
130kDa
95kDa
72kDa
55kDa
43kDa
34kDa
26kDa
170kDa
130kDa
95kDa
72kDa
55kDa
43kDa
34kDa
26kDa
170kDa
130kDa
95kDa
72kDa
55kDa
43kDa
34kDa
26kDa

## Slide 62
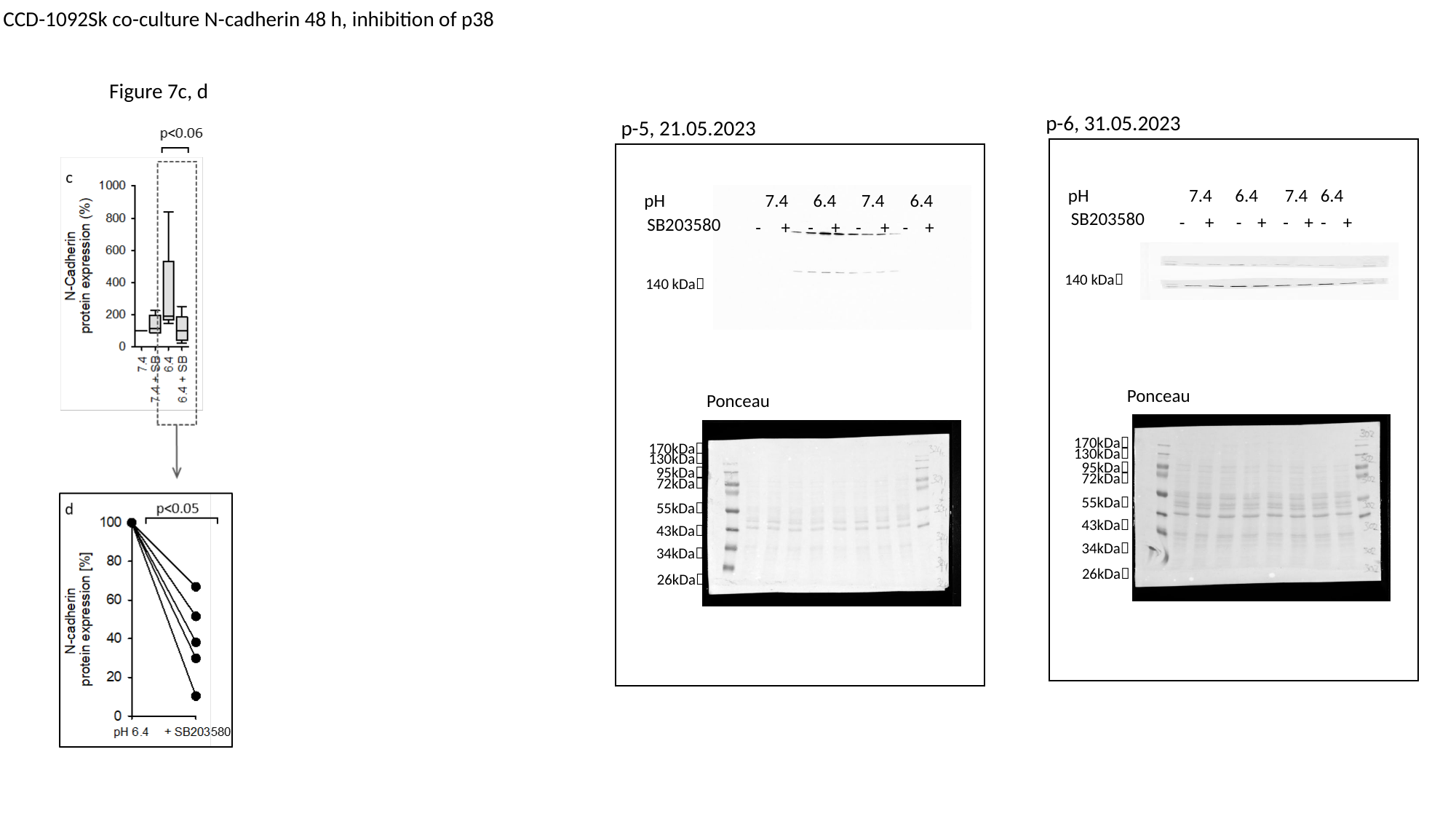

CCD-1092Sk co-culture N-cadherin 48 h, inhibition of p38
Figure 7c, d
p-6, 31.05.2023
p-5, 21.05.2023
pH
7.4
6.4
7.4
6.4
SB203580
+
-
+
-
+
-
+
-
pH
7.4
6.4
7.4
6.4
SB203580
+
-
+
-
+
-
+
-
140 kDa
140 kDa
Ponceau
Ponceau
170kDa
130kDa
95kDa
72kDa
55kDa
43kDa
34kDa
26kDa
170kDa
130kDa
95kDa
72kDa
55kDa
43kDa
34kDa
26kDa

## Slide 63
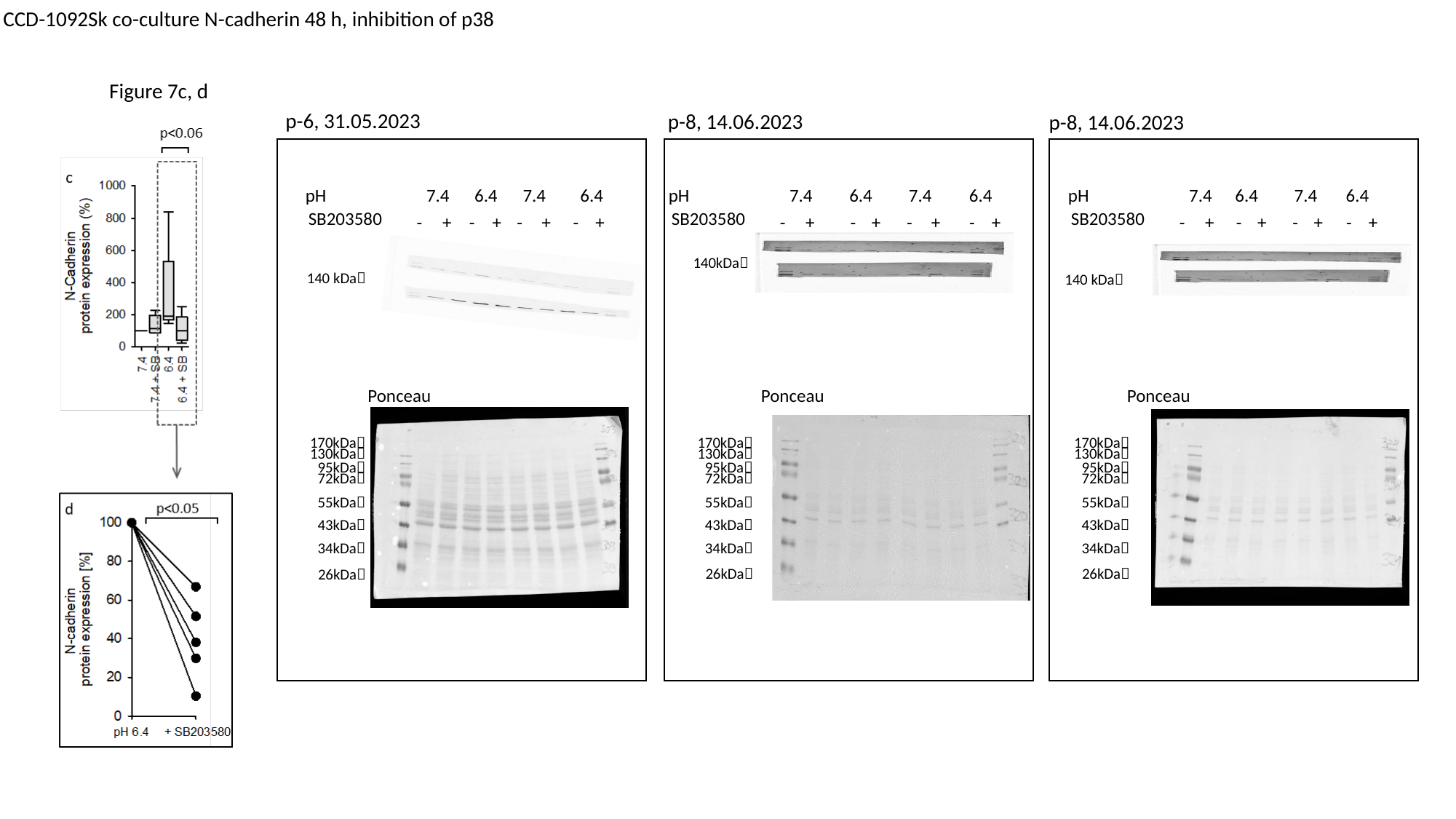

CCD-1092Sk co-culture N-cadherin 48 h, inhibition of p38
Figure 7c, d
p-6, 31.05.2023
p-8, 14.06.2023
p-8, 14.06.2023
pH
7.4
6.4
7.4
6.4
SB203580
+
-
+
-
+
-
+
-
pH
7.4
6.4
7.4
6.4
SB203580
+
-
+
-
+
-
+
-
pH
7.4
6.4
7.4
6.4
SB203580
+
-
+
-
+
-
+
-
140kDa
140 kDa
140 kDa
Ponceau
Ponceau
Ponceau
170kDa
130kDa
95kDa
72kDa
55kDa
43kDa
34kDa
26kDa
170kDa
130kDa
95kDa
72kDa
55kDa
43kDa
34kDa
26kDa
170kDa
130kDa
95kDa
72kDa
55kDa
43kDa
34kDa
26kDa

## Slide 64
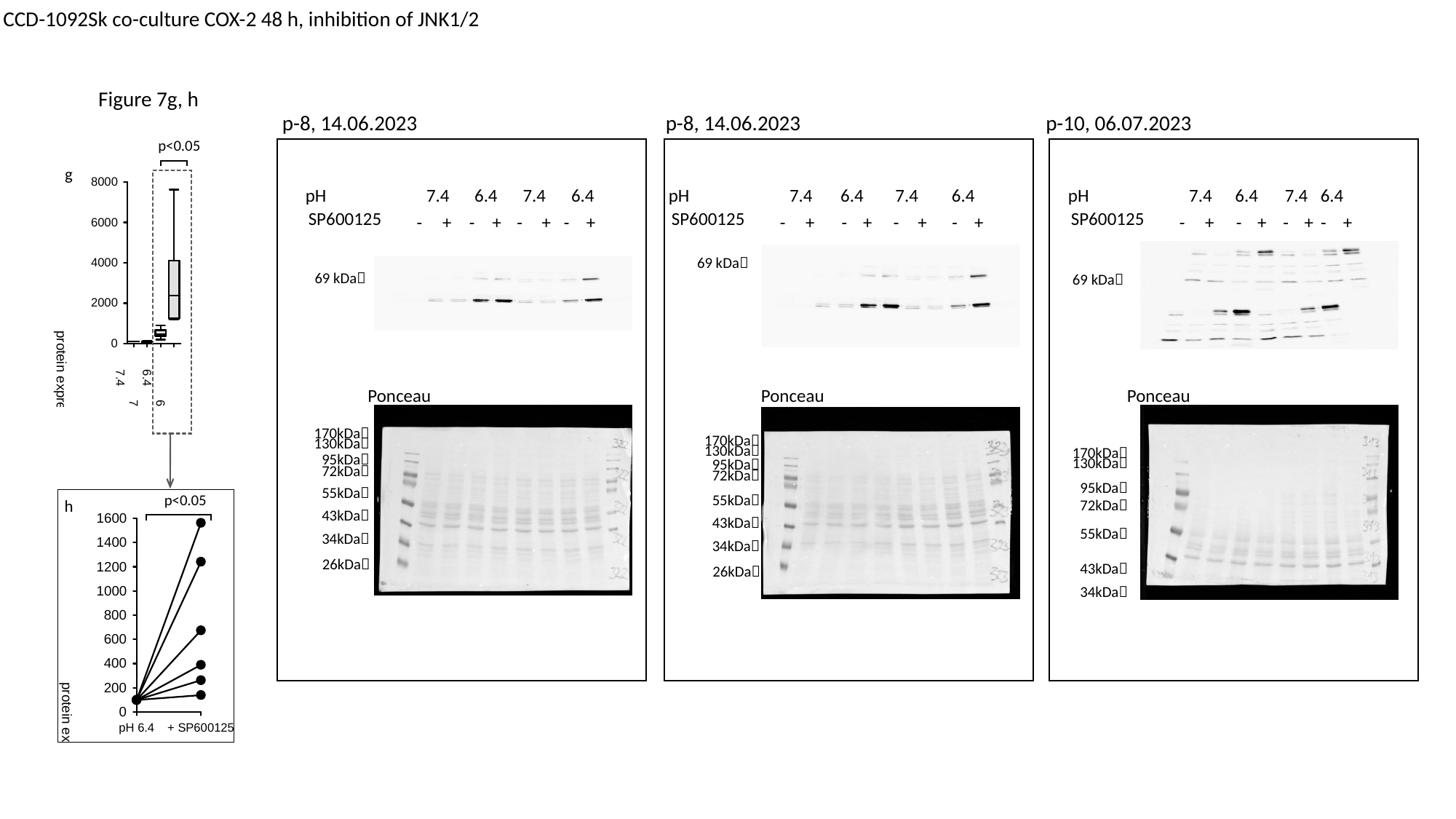

CCD-1092Sk co-culture COX-2 48 h, inhibition of JNK1/2
Figure 7g, h
p-8, 14.06.2023
p-10, 06.07.2023
p-8, 14.06.2023
p<0.05
g
pH
7.4
6.4
7.4
6.4
SP600125
+
-
+
-
+
-
+
-
pH
7.4
6.4
7.4
6.4
SP600125
+
-
+
-
+
-
+
-
pH
7.4
6.4
7.4
6.4
SP600125
+
-
+
-
+
-
+
-
69 kDa
69 kDa
69 kDa
Ponceau
Ponceau
Ponceau
170kDa
130kDa
95kDa
72kDa
55kDa
43kDa
34kDa
26kDa
170kDa
130kDa
95kDa
72kDa
55kDa
43kDa
34kDa
26kDa
170kDa
130kDa
95kDa
72kDa
55kDa
43kDa
34kDa
p<0.05
h

## Slide 65
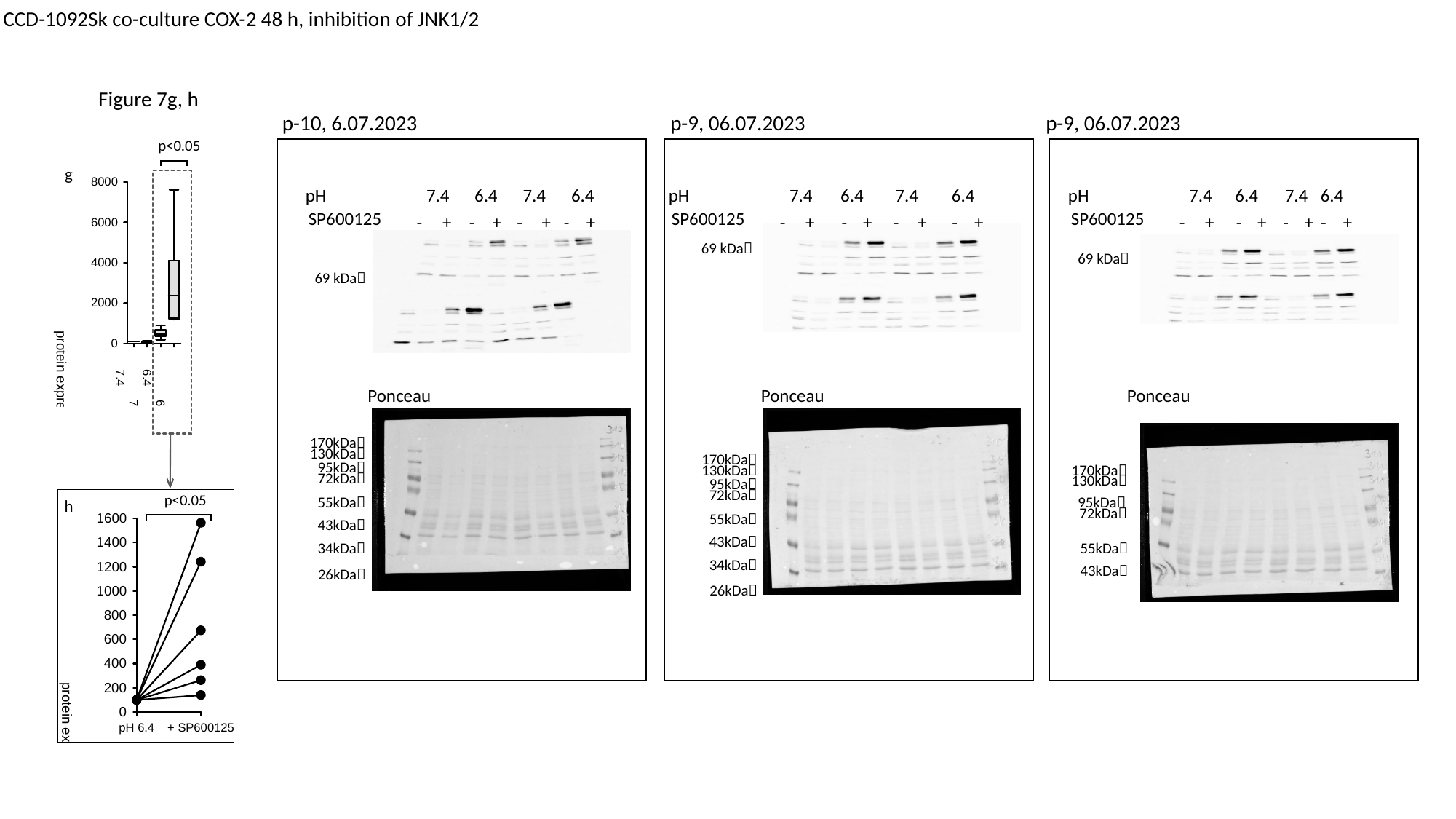

CCD-1092Sk co-culture COX-2 48 h, inhibition of JNK1/2
Figure 7g, h
p-9, 06.07.2023
p-10, 6.07.2023
p-9, 06.07.2023
p<0.05
g
pH
7.4
6.4
7.4
6.4
SP600125
+
-
+
-
+
-
+
-
pH
7.4
6.4
7.4
6.4
SP600125
+
-
+
-
+
-
+
-
pH
7.4
6.4
7.4
6.4
SP600125
+
-
+
-
+
-
+
-
69 kDa
69 kDa
69 kDa
Ponceau
Ponceau
Ponceau
170kDa
130kDa
95kDa
72kDa
55kDa
43kDa
34kDa
26kDa
170kDa
130kDa
95kDa
72kDa
55kDa
43kDa
34kDa
26kDa
170kDa
130kDa
95kDa
72kDa
55kDa
43kDa
p<0.05
h

## Slide 66
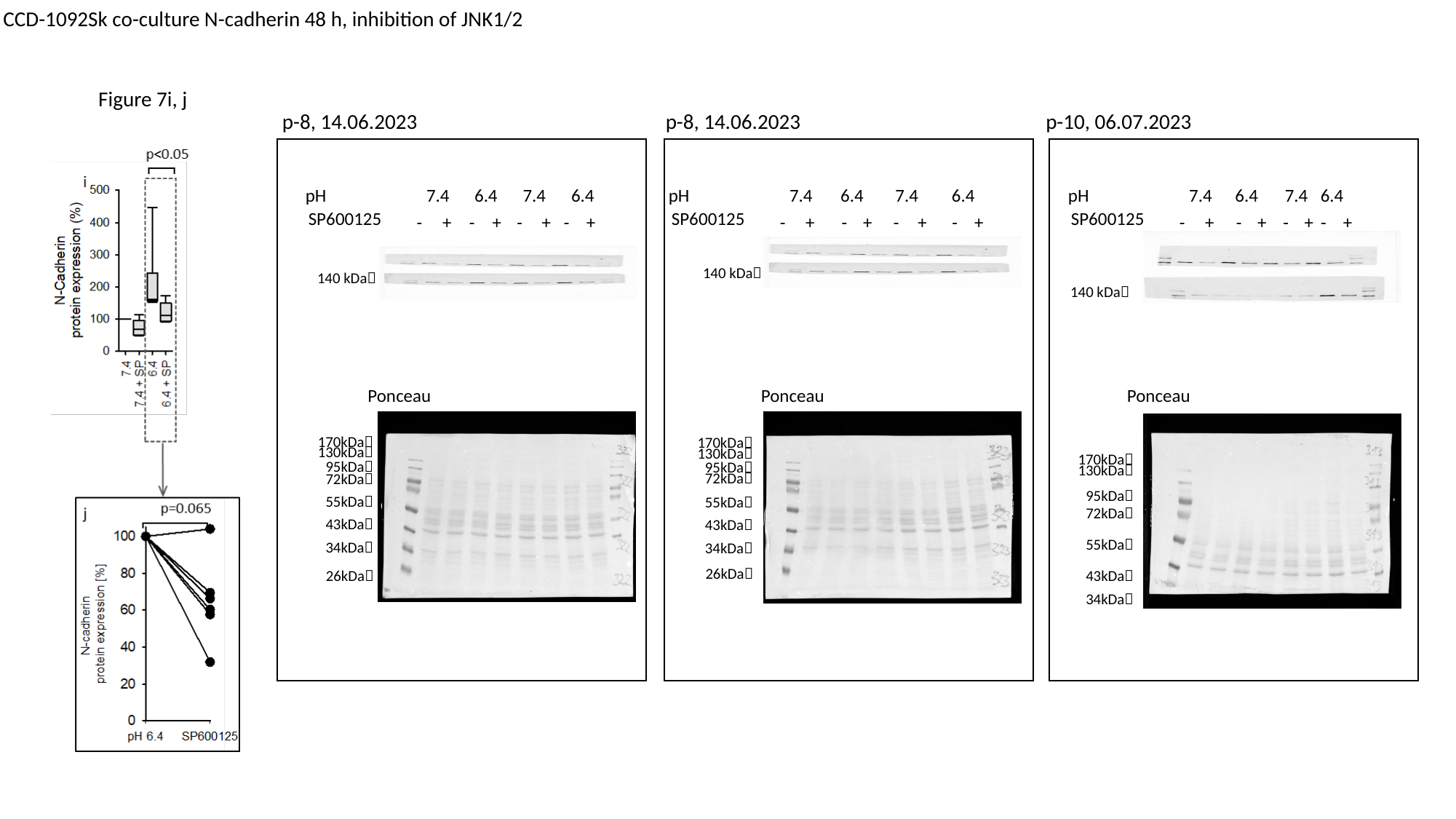

CCD-1092Sk co-culture N-cadherin 48 h, inhibition of JNK1/2
Figure 7i, j
p-8, 14.06.2023
p-10, 06.07.2023
p-8, 14.06.2023
pH
7.4
6.4
7.4
6.4
SP600125
+
-
+
-
+
-
+
-
pH
7.4
6.4
7.4
6.4
SP600125
+
-
+
-
+
-
+
-
pH
7.4
6.4
7.4
6.4
SP600125
+
-
+
-
+
-
+
-
140 kDa
140 kDa
140 kDa
Ponceau
Ponceau
Ponceau
170kDa
130kDa
95kDa
72kDa
55kDa
43kDa
34kDa
26kDa
170kDa
130kDa
95kDa
72kDa
55kDa
43kDa
34kDa
26kDa
170kDa
130kDa
95kDa
72kDa
55kDa
43kDa
34kDa

## Slide 67
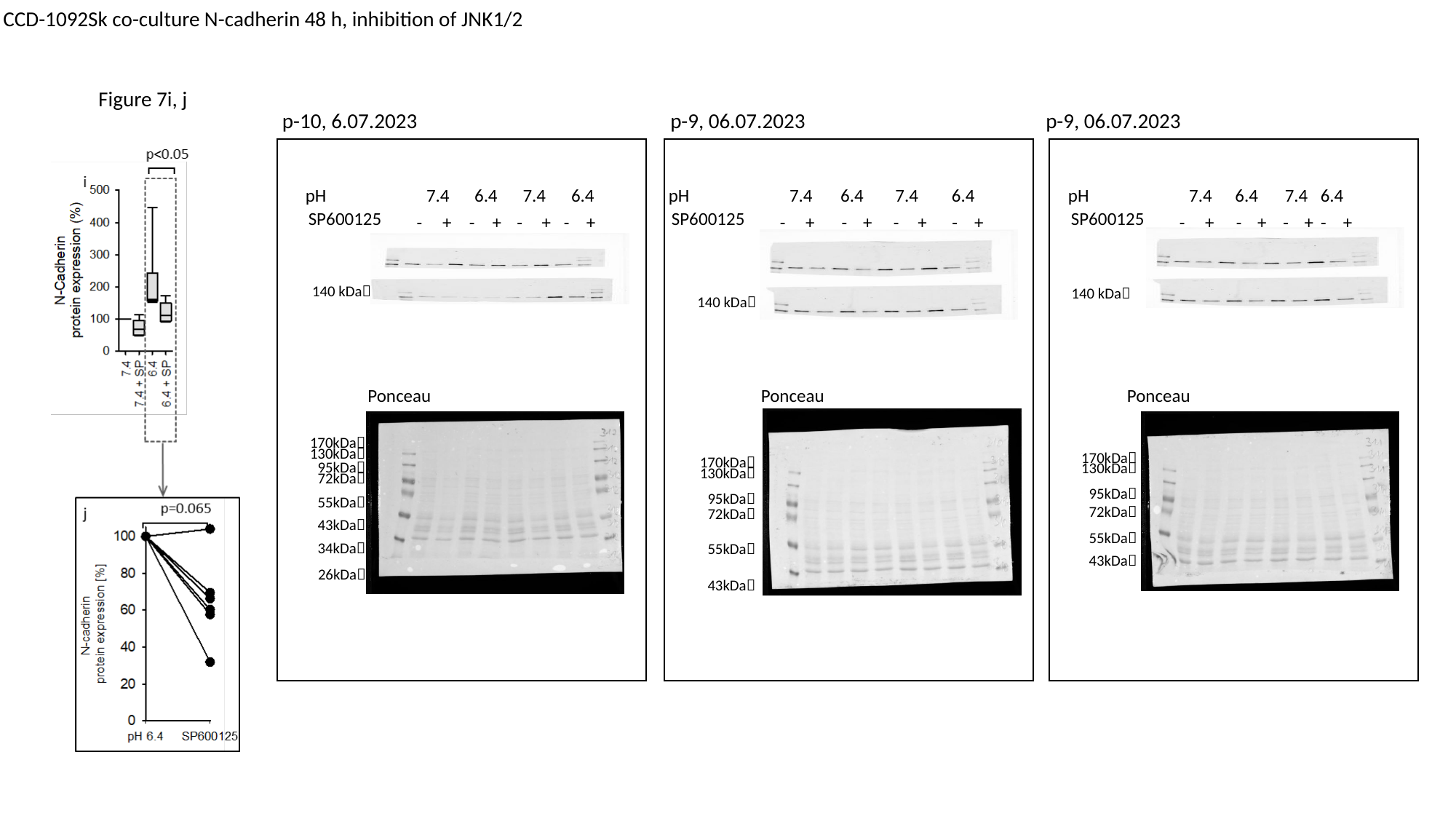

CCD-1092Sk co-culture N-cadherin 48 h, inhibition of JNK1/2
Figure 7i, j
p-9, 06.07.2023
p-10, 6.07.2023
p-9, 06.07.2023
pH
7.4
6.4
7.4
6.4
SP600125
+
-
+
-
+
-
+
-
pH
7.4
6.4
7.4
6.4
SP600125
+
-
+
-
+
-
+
-
pH
7.4
6.4
7.4
6.4
SP600125
+
-
+
-
+
-
+
-
140 kDa
140 kDa
140 kDa
Ponceau
Ponceau
Ponceau
170kDa
130kDa
95kDa
72kDa
55kDa
43kDa
34kDa
26kDa
170kDa
130kDa
95kDa
72kDa
55kDa
43kDa
170kDa
130kDa
95kDa
72kDa
55kDa
43kDa

## Slide 68
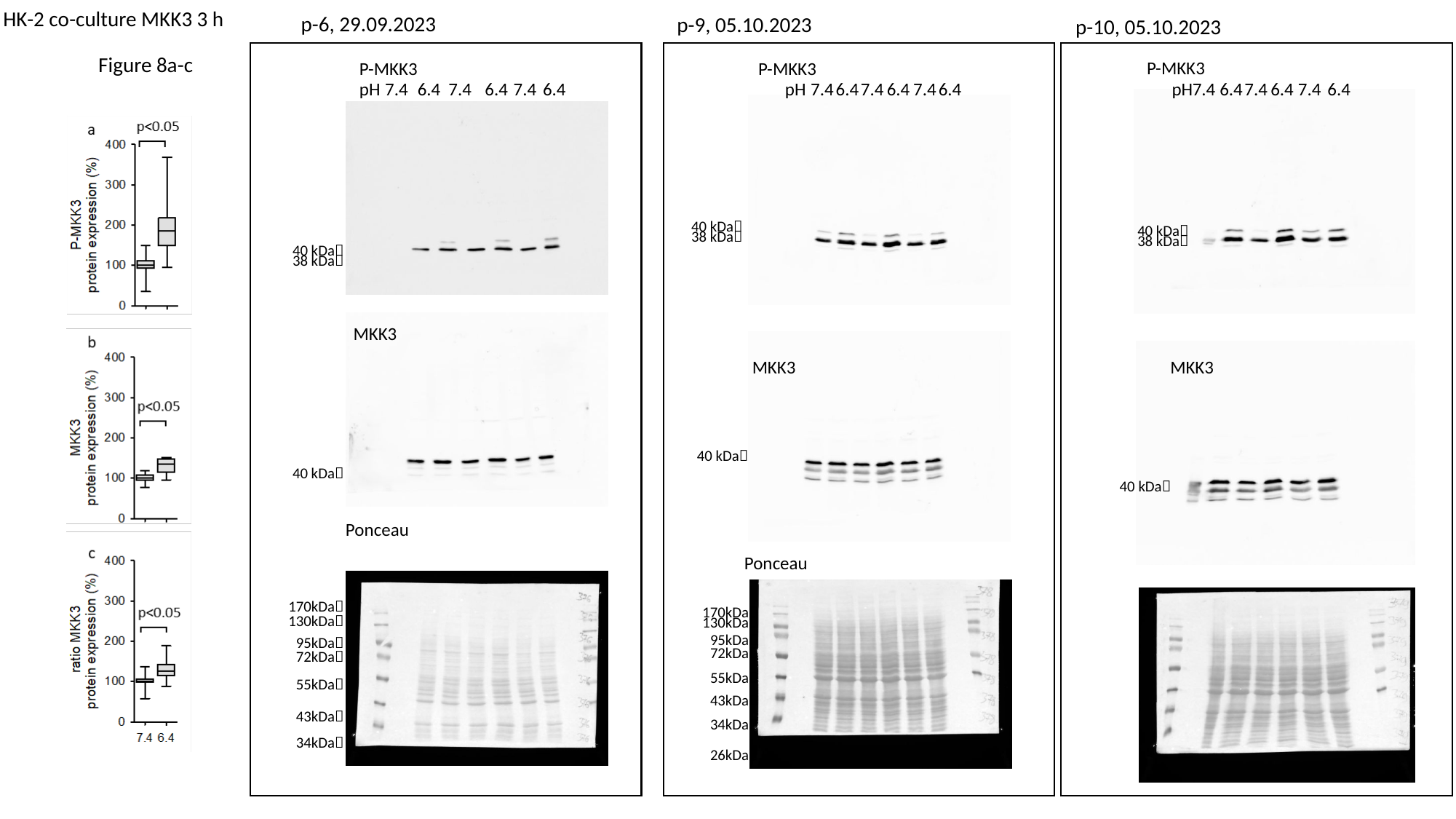

HK-2 co-culture MKK3 3 h
p-6, 29.09.2023
p-9, 05.10.2023
p-10, 05.10.2023
Figure 8a-c
P-MKK3
P-MKK3
P-MKK3
pH
7.4
6.4
7.4
6.4
7.4
6.4
pH
7.4
6.4
7.4
6.4
7.4
6.4
pH
7.4
6.4
7.4
6.4
7.4
6.4
40 kDa
40 kDa
38 kDa
38 kDa
40 kDa
38 kDa
MKK3
MKK3
MKK3
40 kDa
40 kDa
40 kDa
Ponceau
Ponceau
170kDa
130kDa
95kDa
72kDa
55kDa
43kDa
34kDa
170kDa
130kDa
95kDa
72kDa
55kDa
43kDa
34kDa
26kDa

## Slide 69
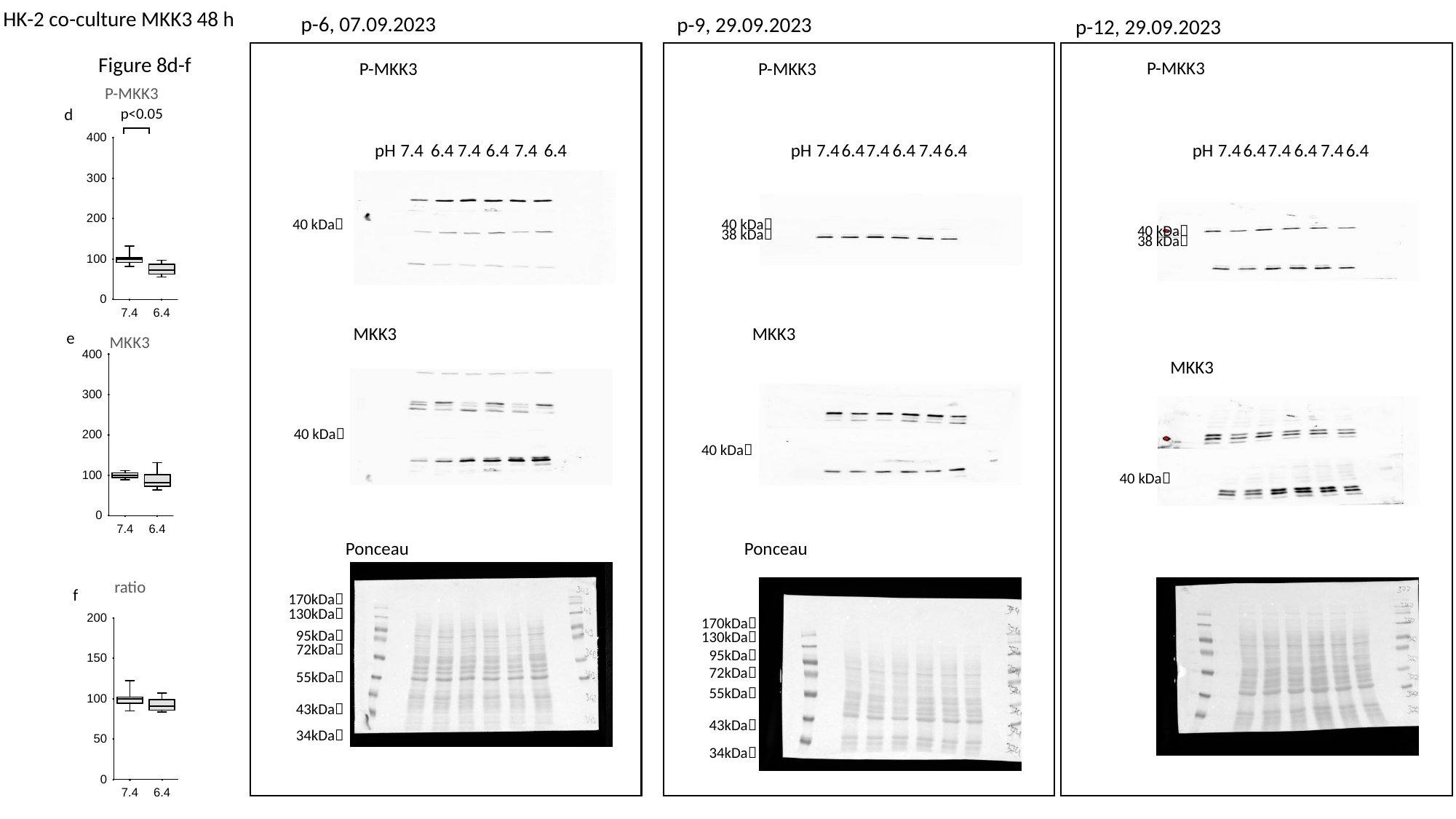

HK-2 co-culture MKK3 48 h
p-6, 07.09.2023
p-9, 29.09.2023
p-12, 29.09.2023
Figure 8d-f
P-MKK3
P-MKK3
P-MKK3
P-MKK3
p<0.05
d
pH
7.4
6.4
7.4
6.4
7.4
6.4
pH
7.4
6.4
7.4
6.4
7.4
6.4
pH
7.4
6.4
7.4
6.4
7.4
6.4
40 kDa
40 kDa
40 kDa
38 kDa
38 kDa
MKK3
MKK3
e
MKK3
MKK3
40 kDa
40 kDa
40 kDa
Ponceau
Ponceau
ratio
f
170kDa
130kDa
95kDa
72kDa
55kDa
43kDa
34kDa
170kDa
130kDa
95kDa
72kDa
55kDa
43kDa
34kDa

## Slide 70
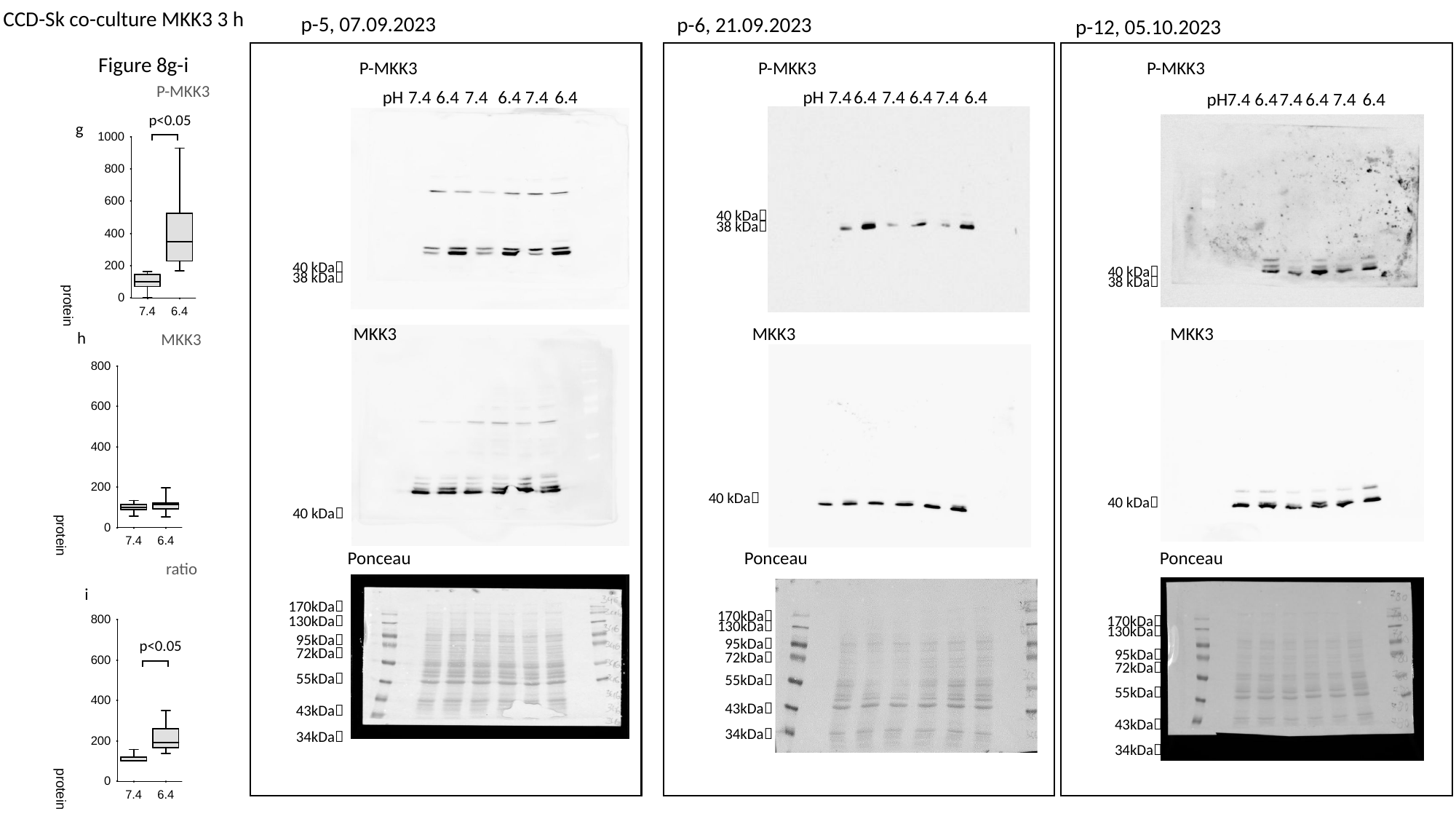

CCD-Sk co-culture MKK3 3 h
p-5, 07.09.2023
p-6, 21.09.2023
p-12, 05.10.2023
Figure 8g-i
P-MKK3
P-MKK3
P-MKK3
P-MKK3
pH
7.4
6.4
7.4
6.4
7.4
6.4
pH
7.4
6.4
7.4
6.4
7.4
6.4
pH
7.4
6.4
7.4
6.4
7.4
6.4
p<0.05
g
40 kDa
38 kDa
40 kDa
40 kDa
38 kDa
38 kDa
MKK3
MKK3
MKK3
h
MKK3
40 kDa
40 kDa
40 kDa
Ponceau
Ponceau
Ponceau
ratio
i
170kDa
130kDa
95kDa
72kDa
55kDa
43kDa
34kDa
170kDa
130kDa
95kDa
72kDa
55kDa
43kDa
34kDa
170kDa
130kDa
95kDa
72kDa
55kDa
43kDa
34kDa
p<0.05

## Slide 71
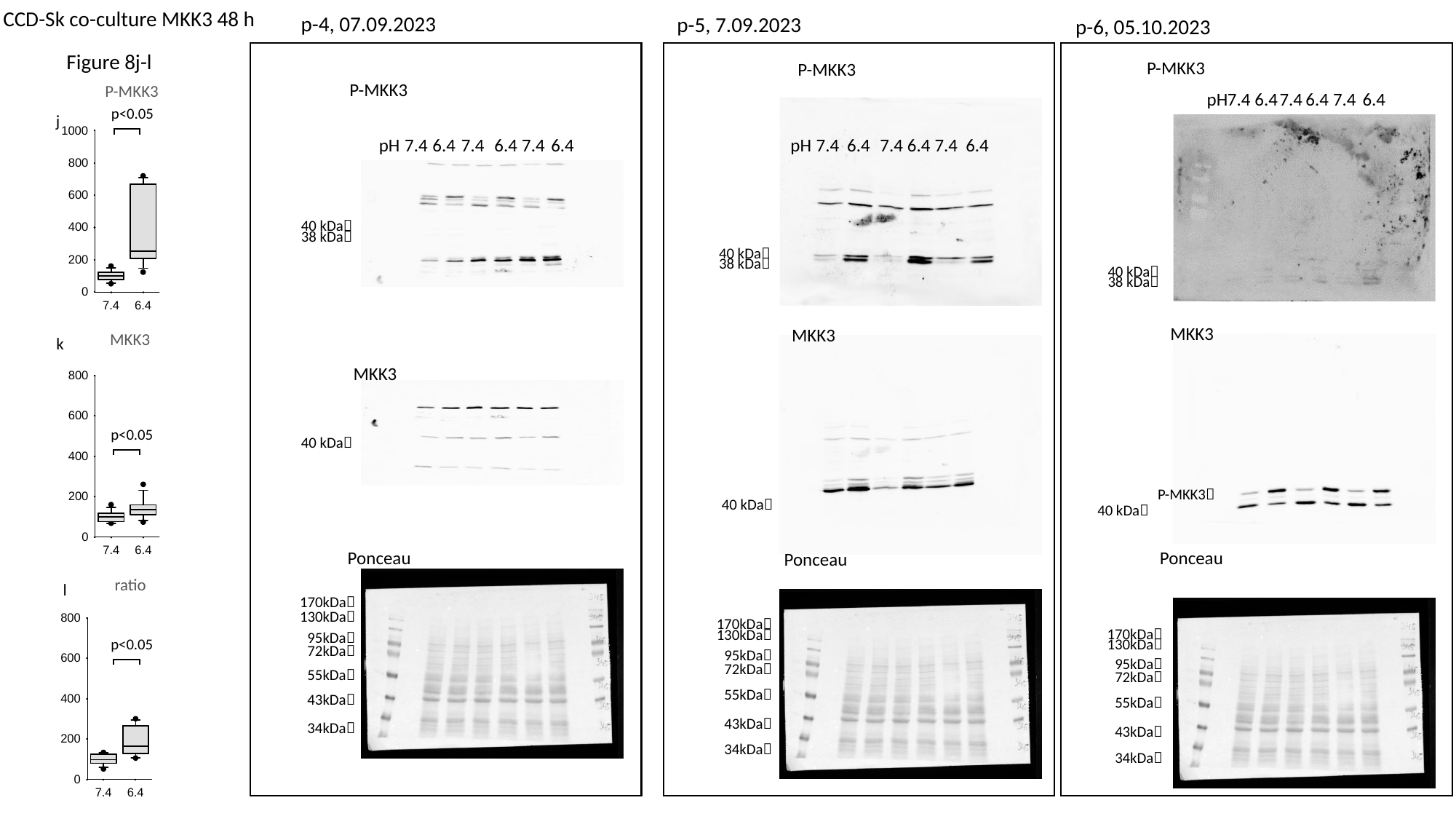

CCD-Sk co-culture MKK3 48 h
p-4, 07.09.2023
p-5, 7.09.2023
p-6, 05.10.2023
Figure 8j-l
P-MKK3
P-MKK3
P-MKK3
P-MKK3
pH
7.4
6.4
7.4
6.4
7.4
6.4
p<0.05
j
pH
7.4
6.4
7.4
6.4
7.4
6.4
pH
7.4
6.4
7.4
6.4
7.4
6.4
40 kDa
38 kDa
40 kDa
38 kDa
40 kDa
38 kDa
MKK3
MKK3
MKK3
k
MKK3
p<0.05
40 kDa
P-MKK3
40 kDa
40 kDa
Ponceau
Ponceau
Ponceau
ratio
l
170kDa
130kDa
95kDa
72kDa
55kDa
43kDa
34kDa
170kDa
130kDa
95kDa
72kDa
55kDa
43kDa
34kDa
170kDa
130kDa
95kDa
72kDa
55kDa
43kDa
34kDa
p<0.05

## Slide 72
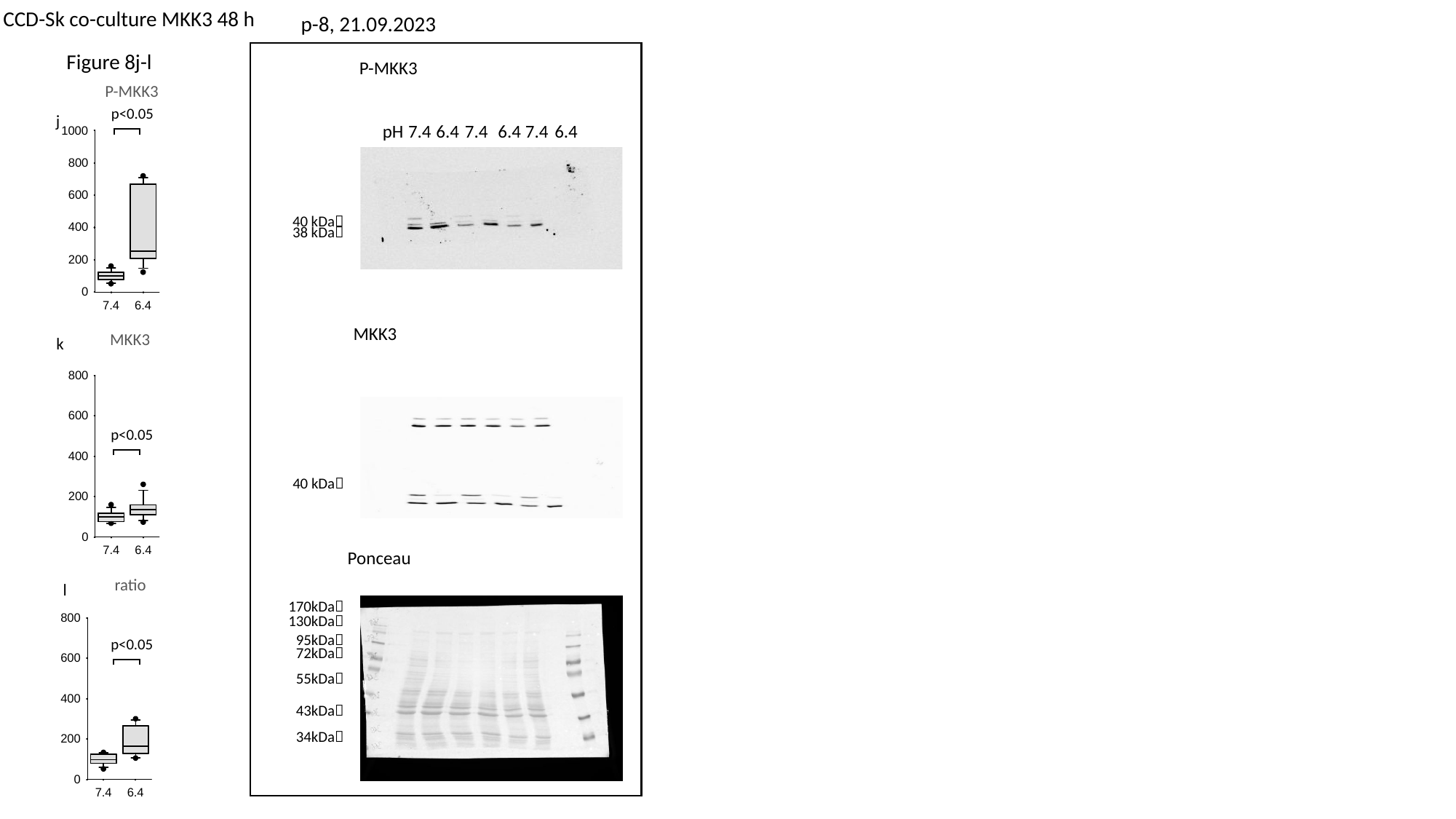

CCD-Sk co-culture MKK3 48 h
p-8, 21.09.2023
Figure 8j-l
P-MKK3
P-MKK3
p<0.05
j
pH
7.4
6.4
7.4
6.4
7.4
6.4
40 kDa
38 kDa
MKK3
MKK3
k
p<0.05
40 kDa
Ponceau
ratio
l
170kDa
130kDa
95kDa
72kDa
55kDa
43kDa
34kDa
p<0.05

## Slide 73
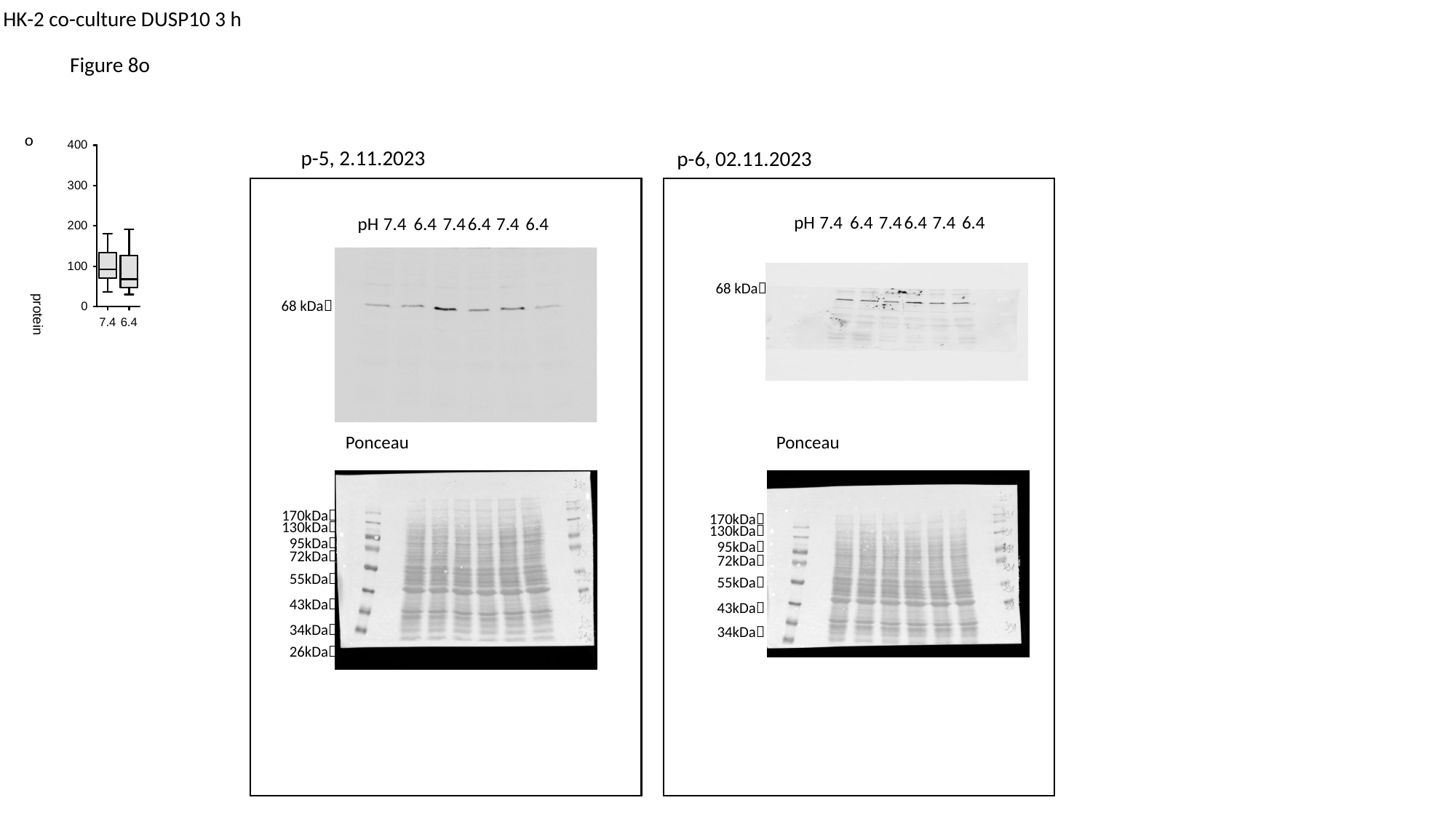

HK-2 co-culture DUSP10 3 h
Figure 8o
o
p-5, 2.11.2023
p-6, 02.11.2023
pH
7.4
6.4
7.4
6.4
7.4
6.4
pH
7.4
6.4
7.4
6.4
7.4
6.4
68 kDa
68 kDa
Ponceau
Ponceau
170kDa
130kDa
95kDa
72kDa
55kDa
43kDa
34kDa
26kDa
170kDa
130kDa
95kDa
72kDa
55kDa
43kDa
34kDa

## Slide 74
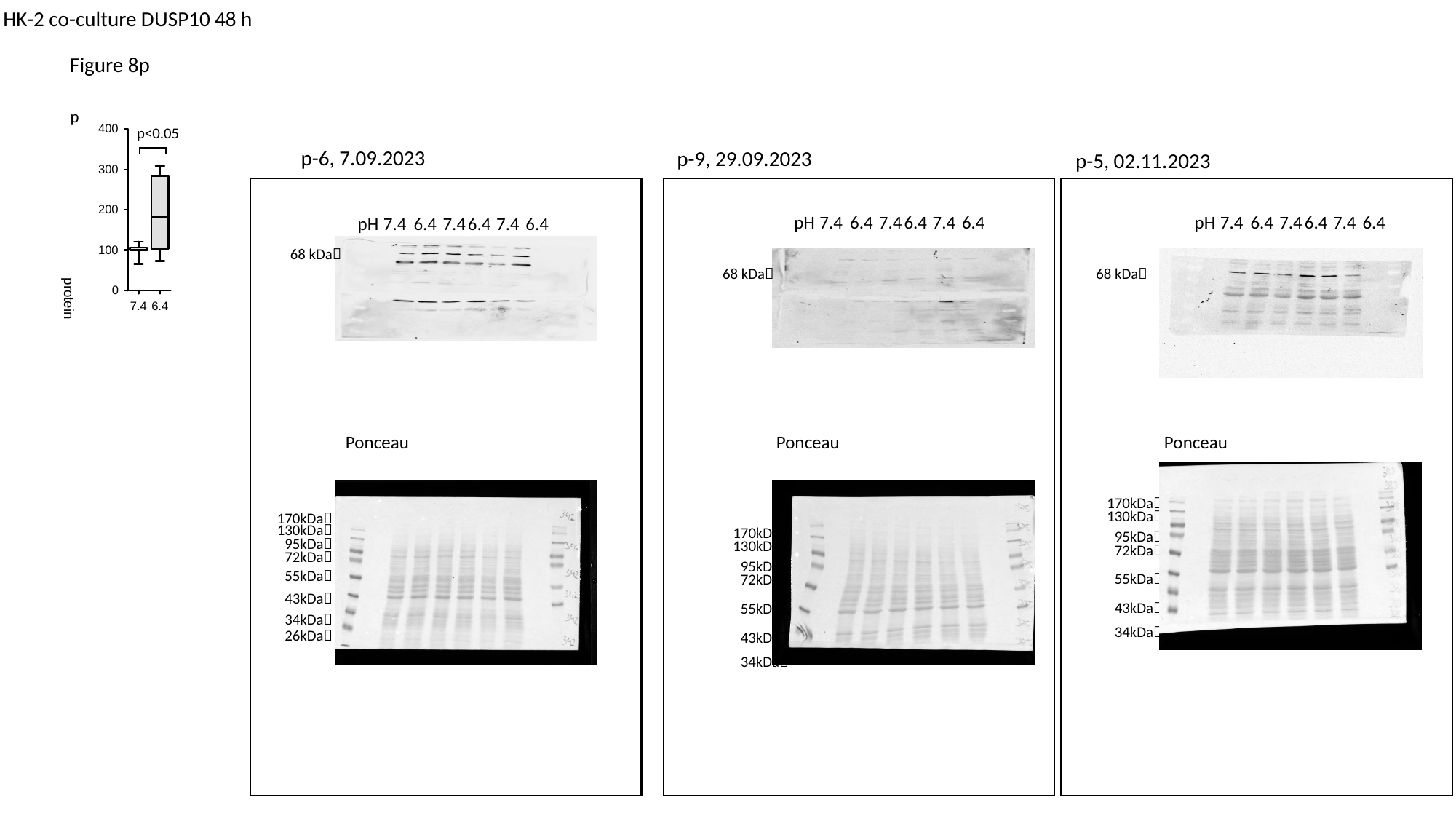

HK-2 co-culture DUSP10 48 h
Figure 8p
p
p<0.05
p-6, 7.09.2023
p-9, 29.09.2023
p-5, 02.11.2023
pH
7.4
6.4
7.4
6.4
7.4
6.4
pH
7.4
6.4
7.4
6.4
7.4
6.4
pH
7.4
6.4
7.4
6.4
7.4
6.4
68 kDa
68 kDa
68 kDa
Ponceau
Ponceau
Ponceau
170kDa
130kDa
95kDa
72kDa
55kDa
43kDa
34kDa
170kDa
130kDa
95kDa
72kDa
55kDa
43kDa
34kDa
26kDa
170kDa
130kDa
95kDa
72kDa
55kDa
43kDa
34kDa

## Slide 75
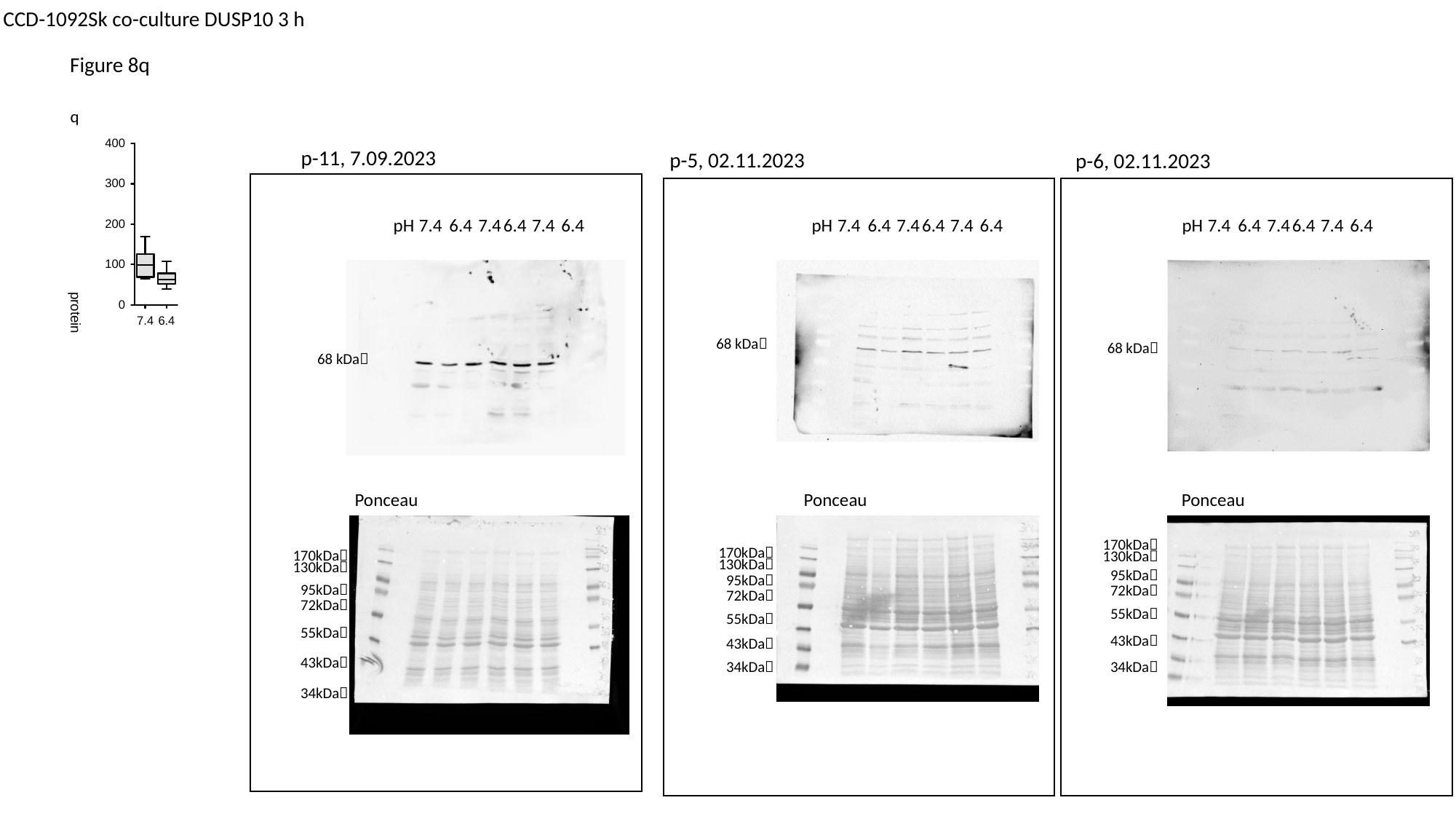

CCD-1092Sk co-culture DUSP10 3 h
Figure 8q
q
p-11, 7.09.2023
p-5, 02.11.2023
p-6, 02.11.2023
pH
7.4
6.4
7.4
6.4
7.4
6.4
pH
7.4
6.4
7.4
6.4
7.4
6.4
pH
7.4
6.4
7.4
6.4
7.4
6.4
68 kDa
68 kDa
68 kDa
Ponceau
Ponceau
Ponceau
170kDa
130kDa
95kDa
72kDa
55kDa
43kDa
34kDa
170kDa
130kDa
95kDa
72kDa
55kDa
43kDa
34kDa
170kDa
130kDa
95kDa
72kDa
55kDa
43kDa
34kDa

## Slide 76
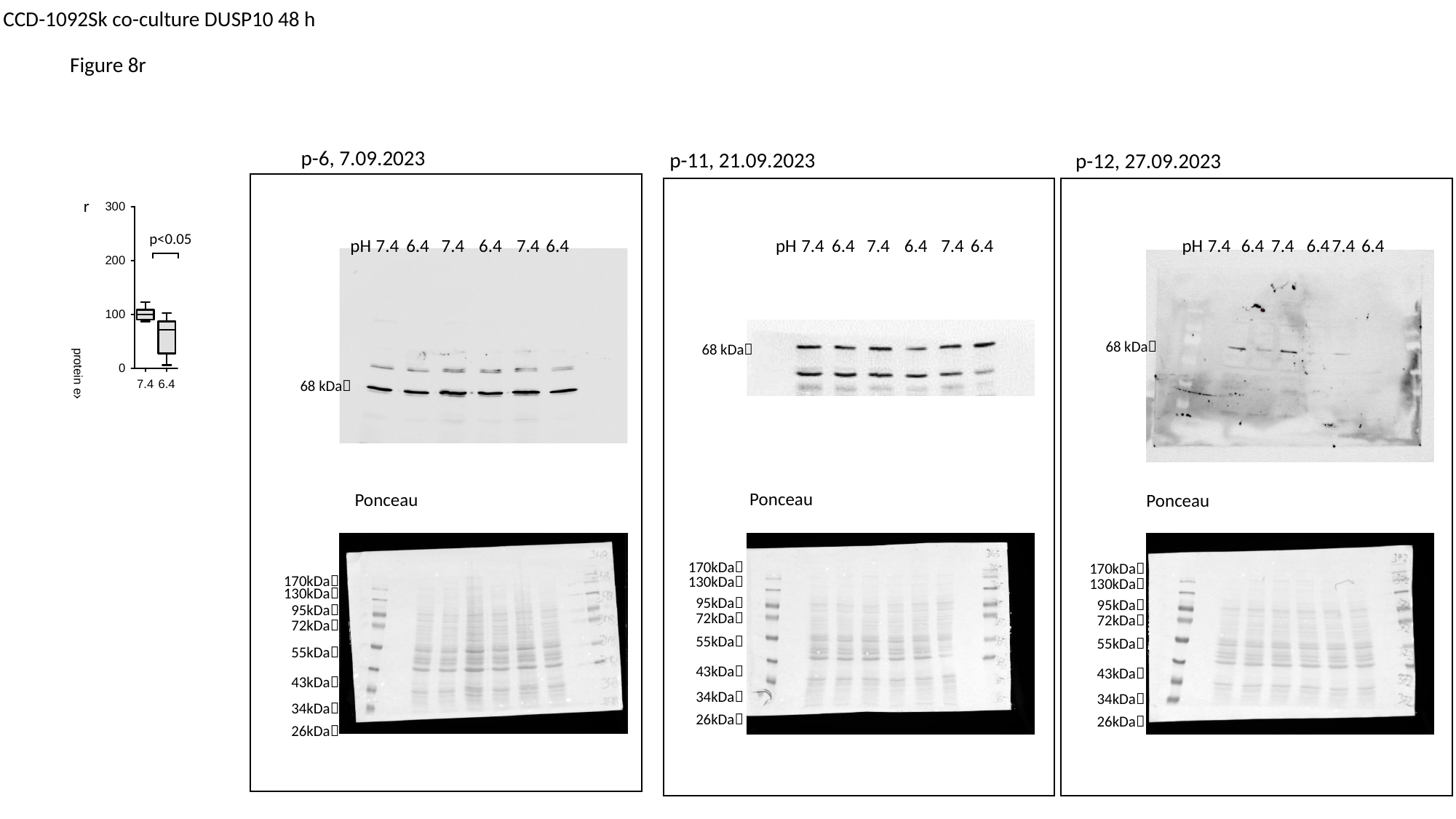

CCD-1092Sk co-culture DUSP10 48 h
Figure 8r
p-6, 7.09.2023
p-11, 21.09.2023
p-12, 27.09.2023
r
p<0.05
pH
7.4
6.4
7.4
6.4
7.4
6.4
pH
7.4
6.4
7.4
6.4
7.4
6.4
pH
7.4
6.4
7.4
6.4
7.4
6.4
68 kDa
68 kDa
68 kDa
Ponceau
Ponceau
Ponceau
170kDa
130kDa
95kDa
72kDa
55kDa
43kDa
34kDa
26kDa
170kDa
130kDa
95kDa
72kDa
55kDa
43kDa
34kDa
26kDa
170kDa
130kDa
95kDa
72kDa
55kDa
43kDa
34kDa
26kDa

## Slide 77
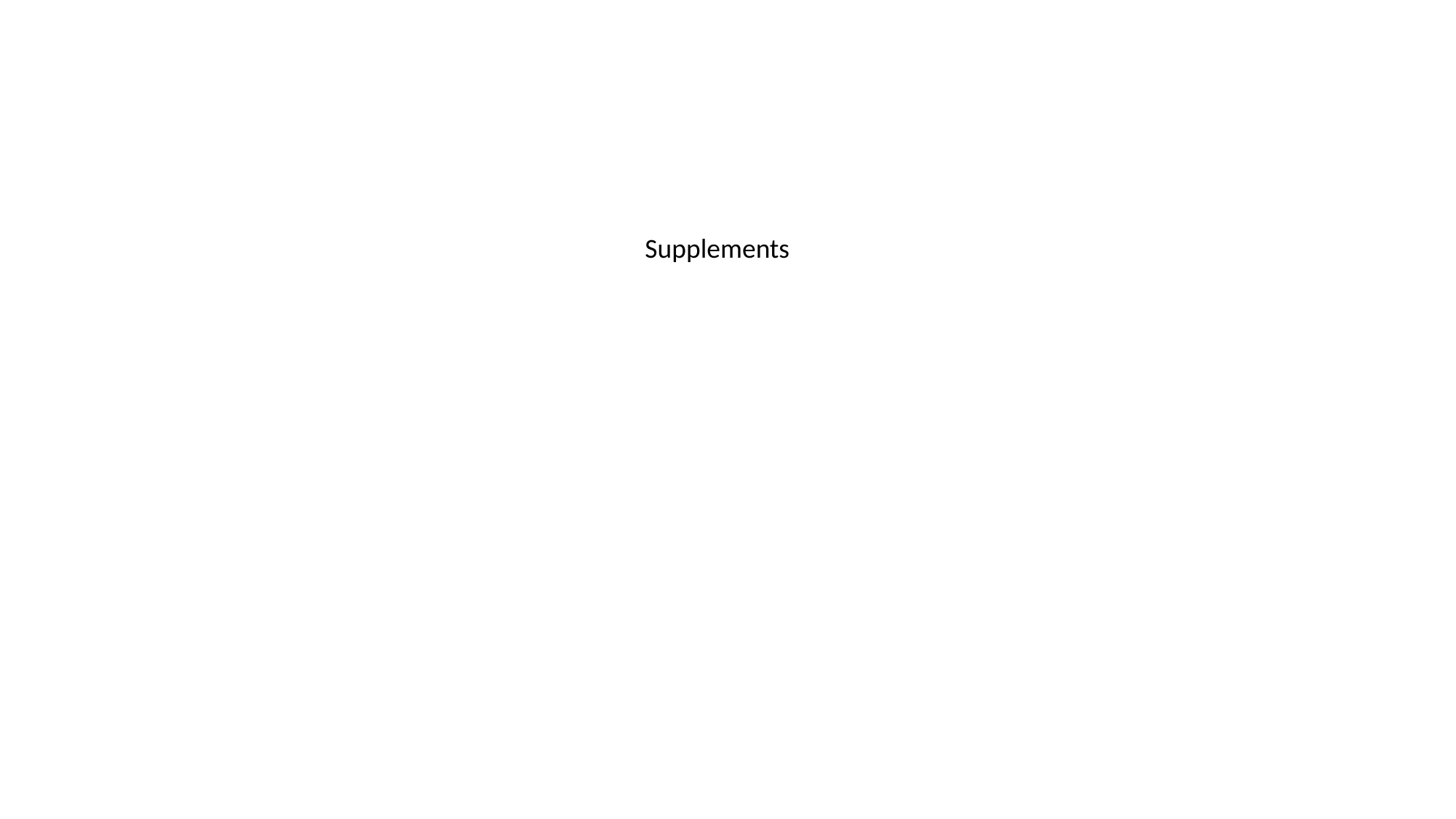

# Supplements

## Slide 78
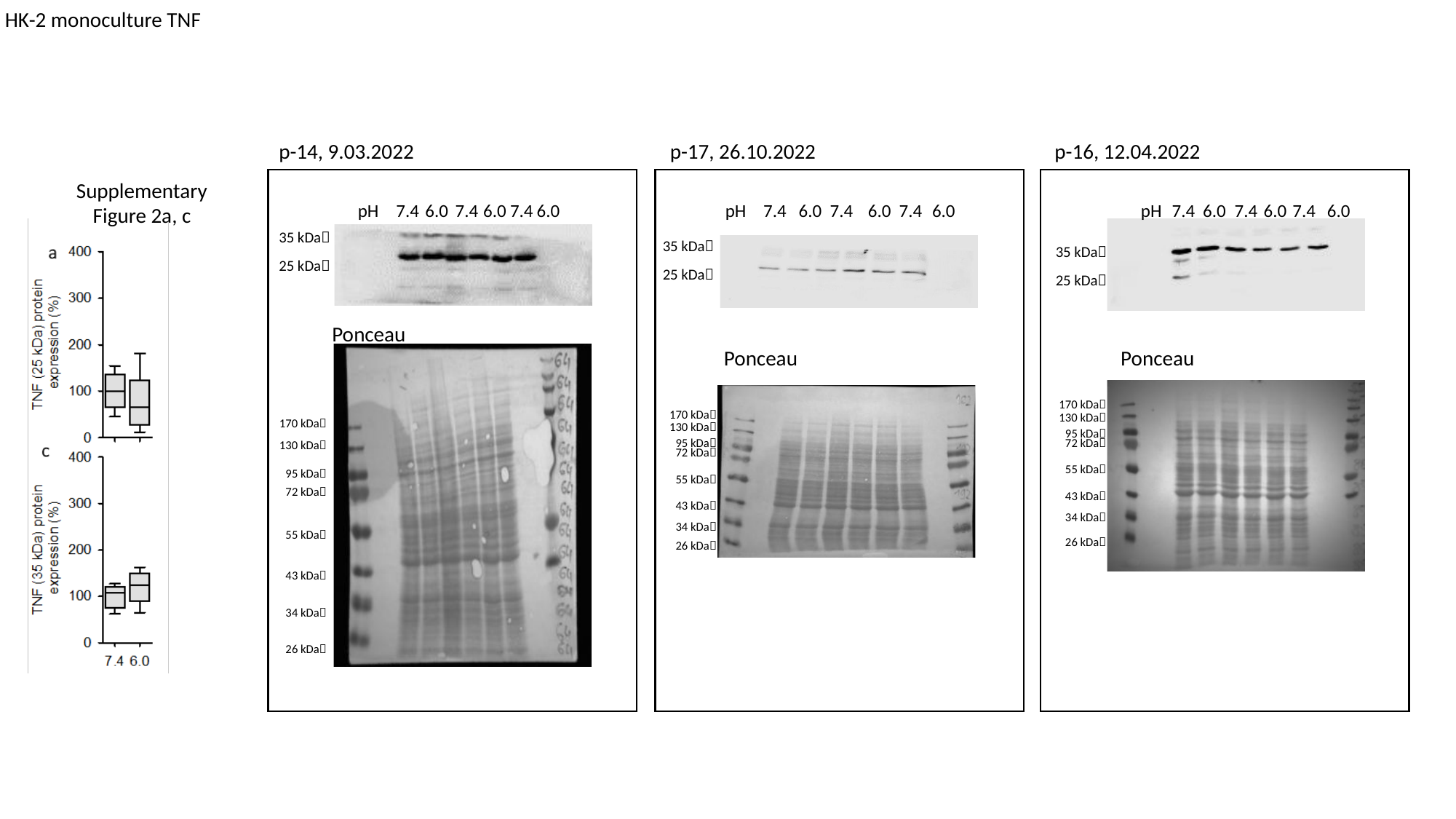

HK-2 monoculture TNF
p-14, 9.03.2022
p-17, 26.10.2022
p-16, 12.04.2022
Supplementary Figure 2a, c
pH
7.4
6.0
7.4
6.0
7.4
6.0
pH
7.4
6.0
7.4
6.0
7.4
6.0
pH
7.4
6.0
7.4
6.0
7.4
6.0
35 kDa
35 kDa
35 kDa
25 kDa
25 kDa
25 kDa
Ponceau
Ponceau
Ponceau
170 kDa
130 kDa
95 kDa
72 kDa
55 kDa
43 kDa
34 kDa
26 kDa
170 kDa
130 kDa
95 kDa
72 kDa
55 kDa
43 kDa
34 kDa
26 kDa
170 kDa
130 kDa
95 kDa
72 kDa
55 kDa
43 kDa
34 kDa
26 kDa

## Slide 79
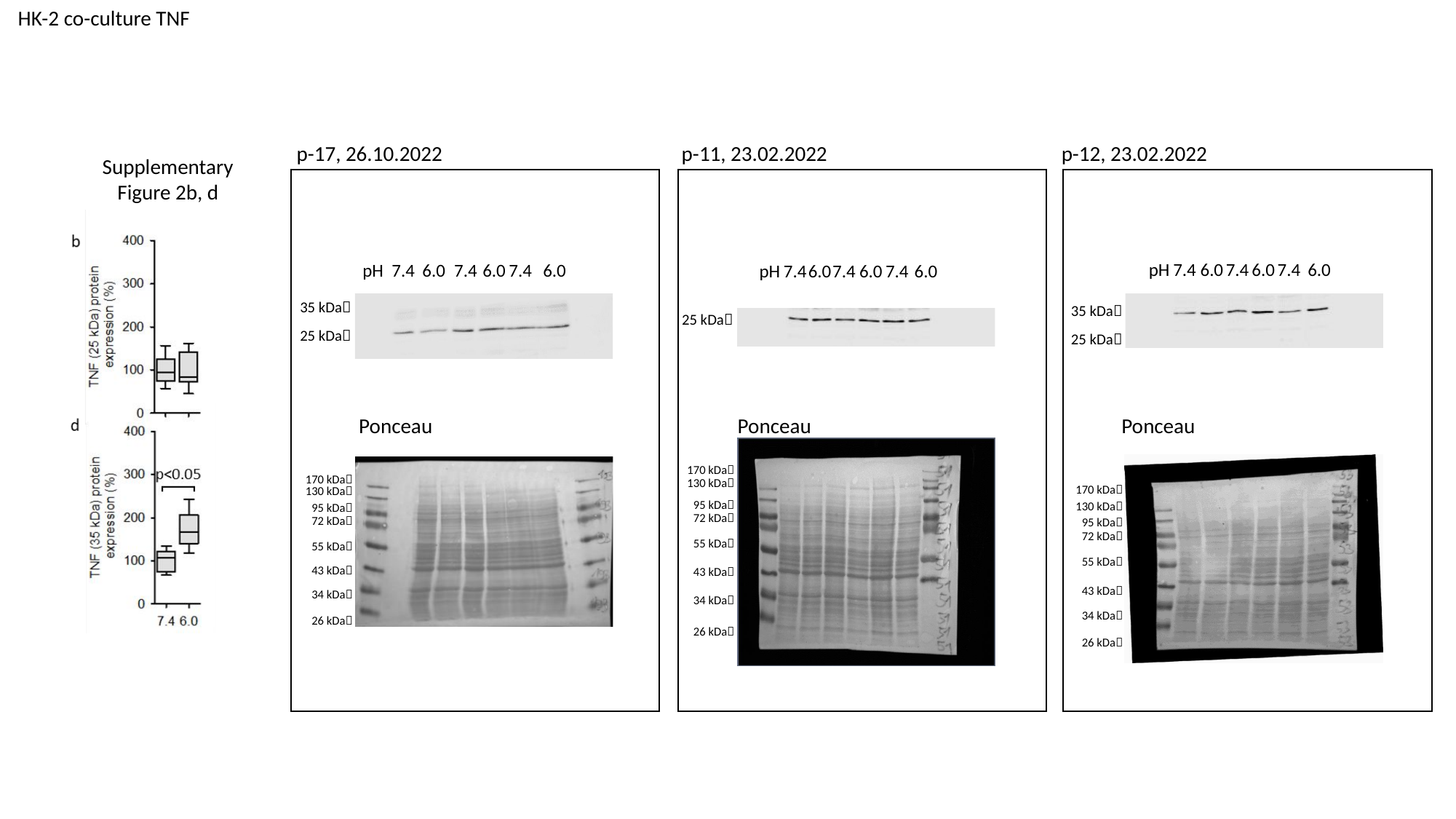

HK-2 co-culture TNF
p-17, 26.10.2022
p-11, 23.02.2022
p-12, 23.02.2022
Supplementary Figure 2b, d
pH
7.4
6.0
7.4
6.0
7.4
6.0
pH
7.4
6.0
7.4
6.0
7.4
6.0
pH
7.4
6.0
7.4
6.0
7.4
6.0
35 kDa
35 kDa
25 kDa
25 kDa
25 kDa
Ponceau
Ponceau
Ponceau
170 kDa
130 kDa
95 kDa
72 kDa
55 kDa
43 kDa
34 kDa
26 kDa
170 kDa
130 kDa
95 kDa
72 kDa
55 kDa
43 kDa
34 kDa
26 kDa
170 kDa
130 kDa
95 kDa
72 kDa
55 kDa
43 kDa
34 kDa
26 kDa

## Slide 80
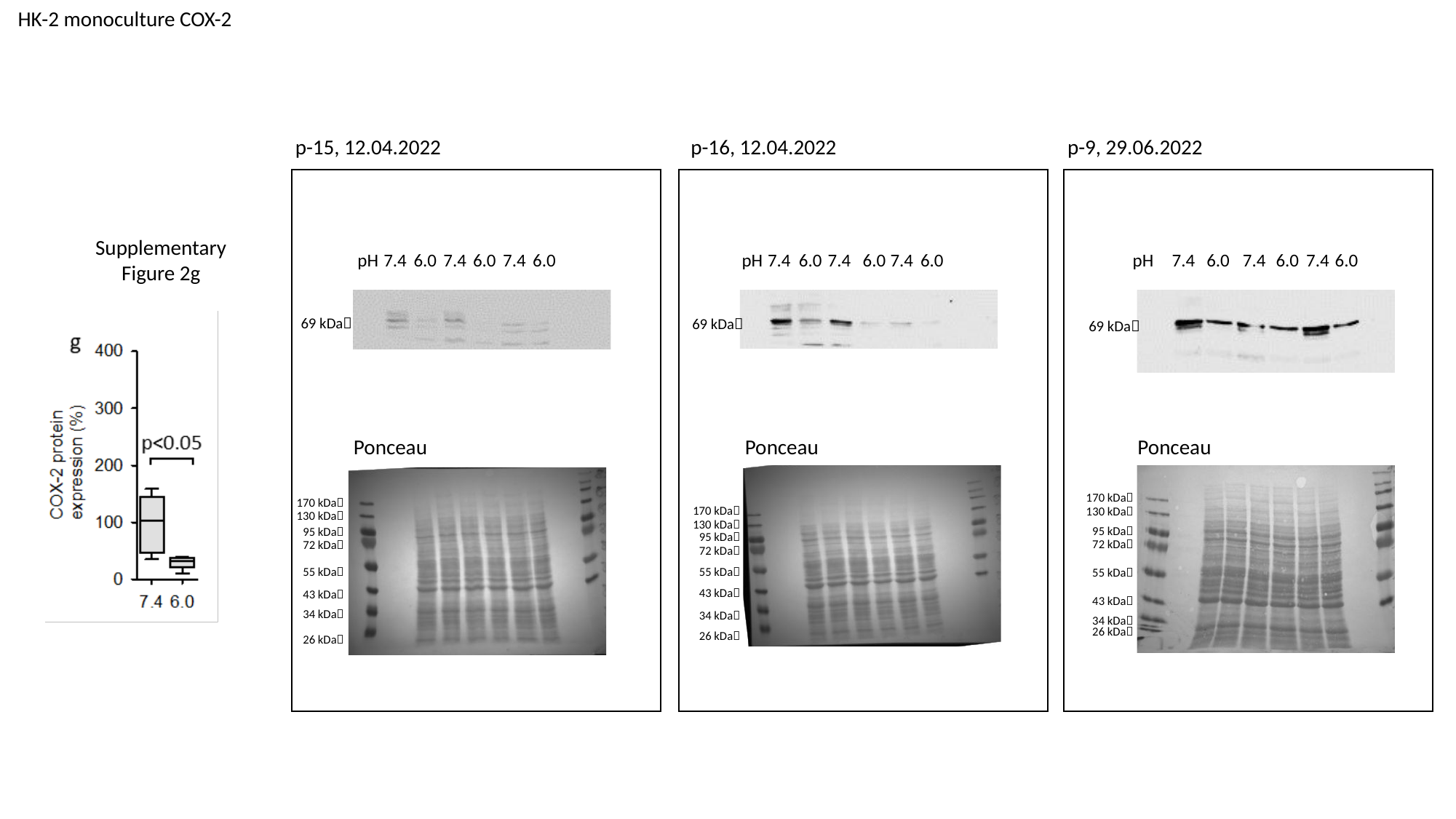

HK-2 monoculture COX-2
p-15, 12.04.2022
p-16, 12.04.2022
p-9, 29.06.2022
Supplementary Figure 2g
pH
7.4
6.0
7.4
6.0
7.4
6.0
pH
7.4
6.0
7.4
6.0
7.4
6.0
pH
7.4
6.0
7.4
6.0
7.4
6.0
69 kDa
69 kDa
69 kDa
Ponceau
Ponceau
Ponceau
170 kDa
130 kDa
95 kDa
72 kDa
55 kDa
43 kDa
34 kDa
26 kDa
170 kDa
130 kDa
95 kDa
72 kDa
55 kDa
43 kDa
34 kDa
26 kDa
170 kDa
130 kDa
95 kDa
72 kDa
55 kDa
43 kDa
34 kDa
26 kDa

## Slide 81
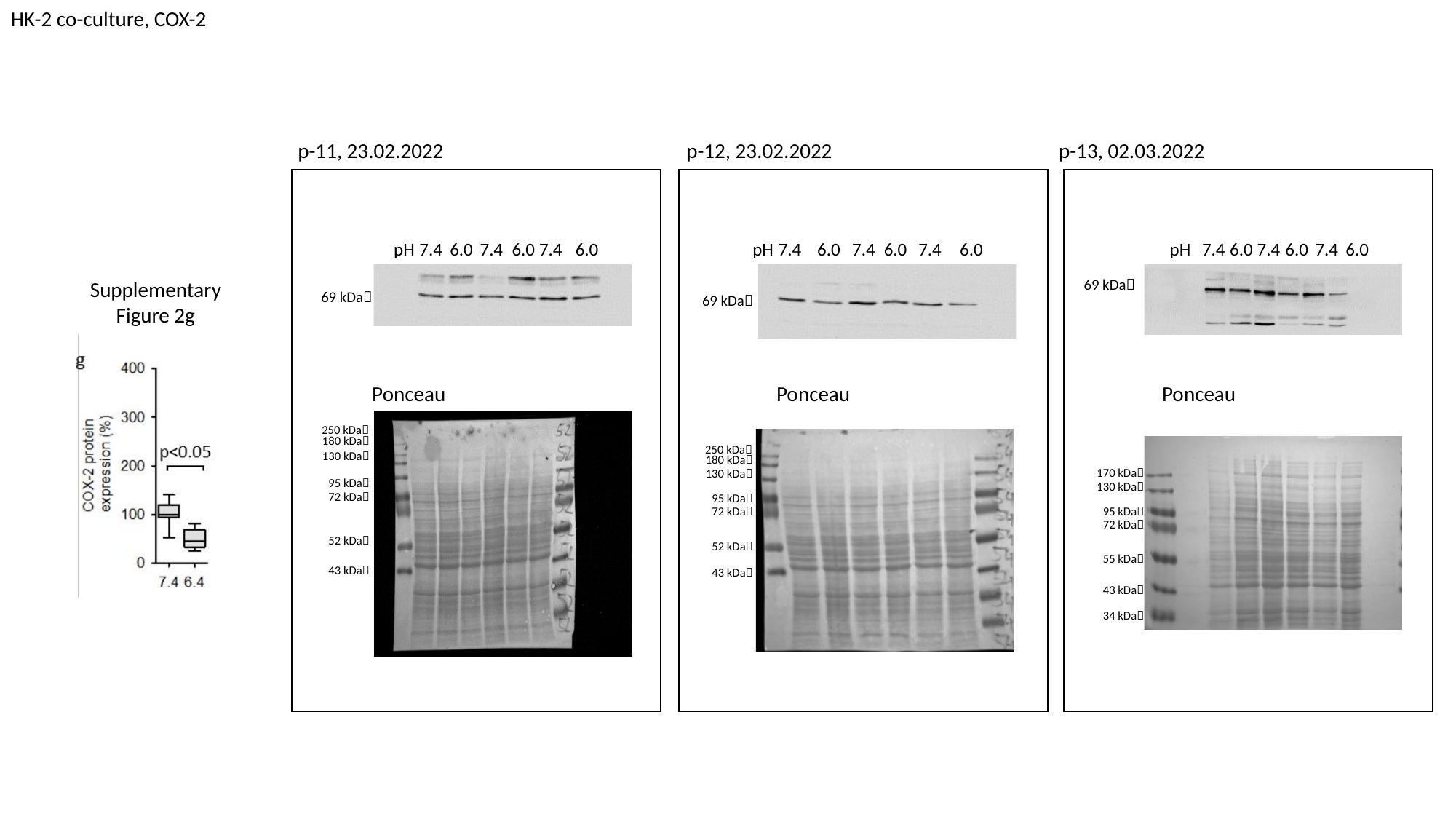

HK-2 co-culture, COX-2
p-11, 23.02.2022
p-12, 23.02.2022
p-13, 02.03.2022
pH
7.4
6.0
7.4
6.0
7.4
6.0
pH
7.4
6.0
7.4
6.0
7.4
6.0
pH
7.4
6.0
7.4
6.0
7.4
6.0
69 kDa
Supplementary Figure 2g
69 kDa
69 kDa
Ponceau
Ponceau
Ponceau
250 kDa
180 kDa
130 kDa
95 kDa
72 kDa
52 kDa
43 kDa
250 kDa
180 kDa
130 kDa
95 kDa
72 kDa
52 kDa
43 kDa
170 kDa
130 kDa
95 kDa
72 kDa
55 kDa
43 kDa
34 kDa

## Slide 82
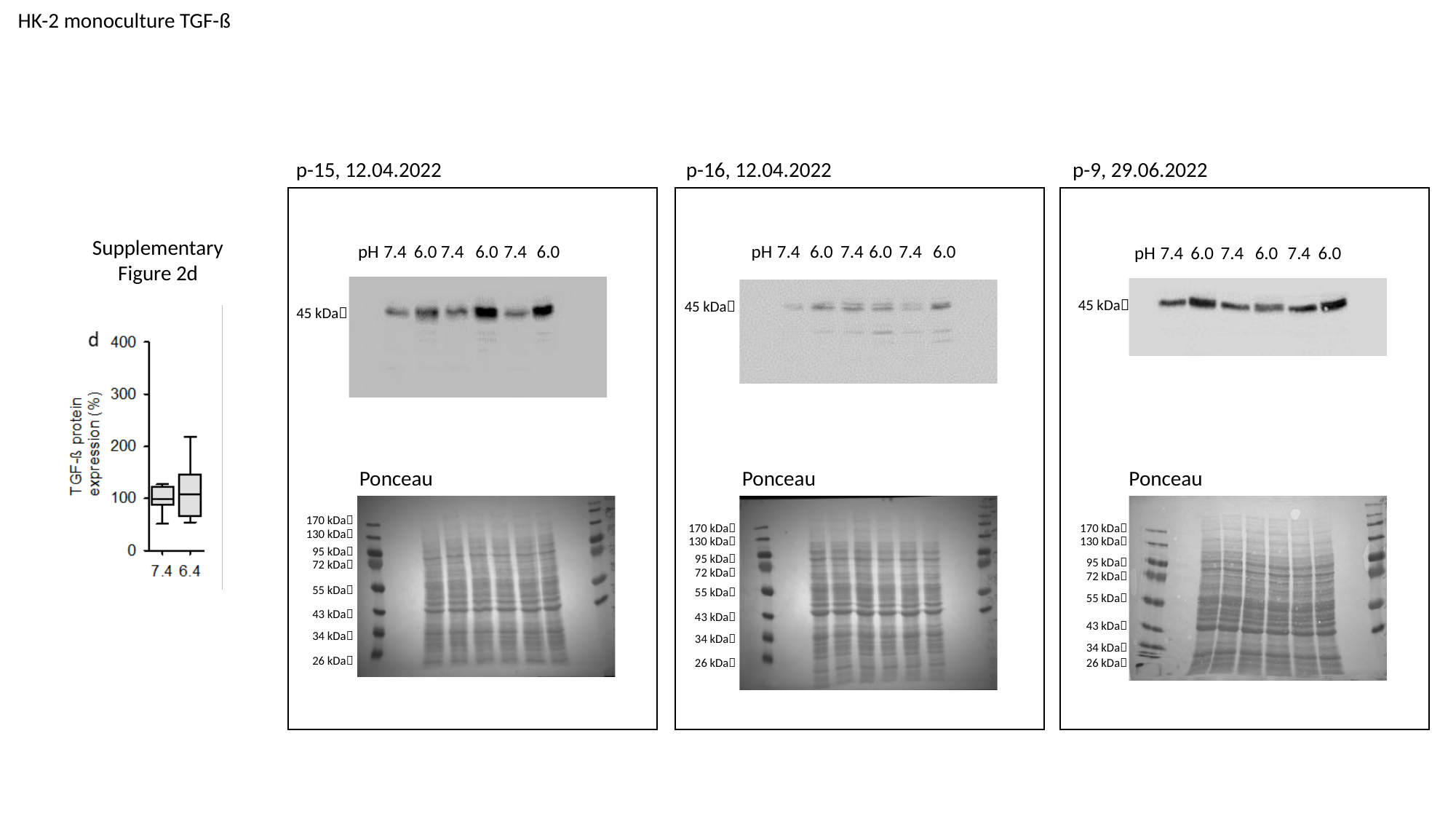

HK-2 monoculture TGF-ß
p-15, 12.04.2022
p-16, 12.04.2022
p-9, 29.06.2022
Supplementary Figure 2d
pH
7.4
6.0
7.4
6.0
7.4
6.0
pH
7.4
6.0
7.4
6.0
7.4
6.0
pH
7.4
6.0
7.4
6.0
7.4
6.0
45 kDa
45 kDa
45 kDa
Ponceau
Ponceau
Ponceau
170 kDa
130 kDa
95 kDa
72 kDa
55 kDa
43 kDa
34 kDa
26 kDa
170 kDa
130 kDa
95 kDa
72 kDa
55 kDa
43 kDa
34 kDa
26 kDa
170 kDa
130 kDa
95 kDa
72 kDa
55 kDa
43 kDa
34 kDa
26 kDa

## Slide 83
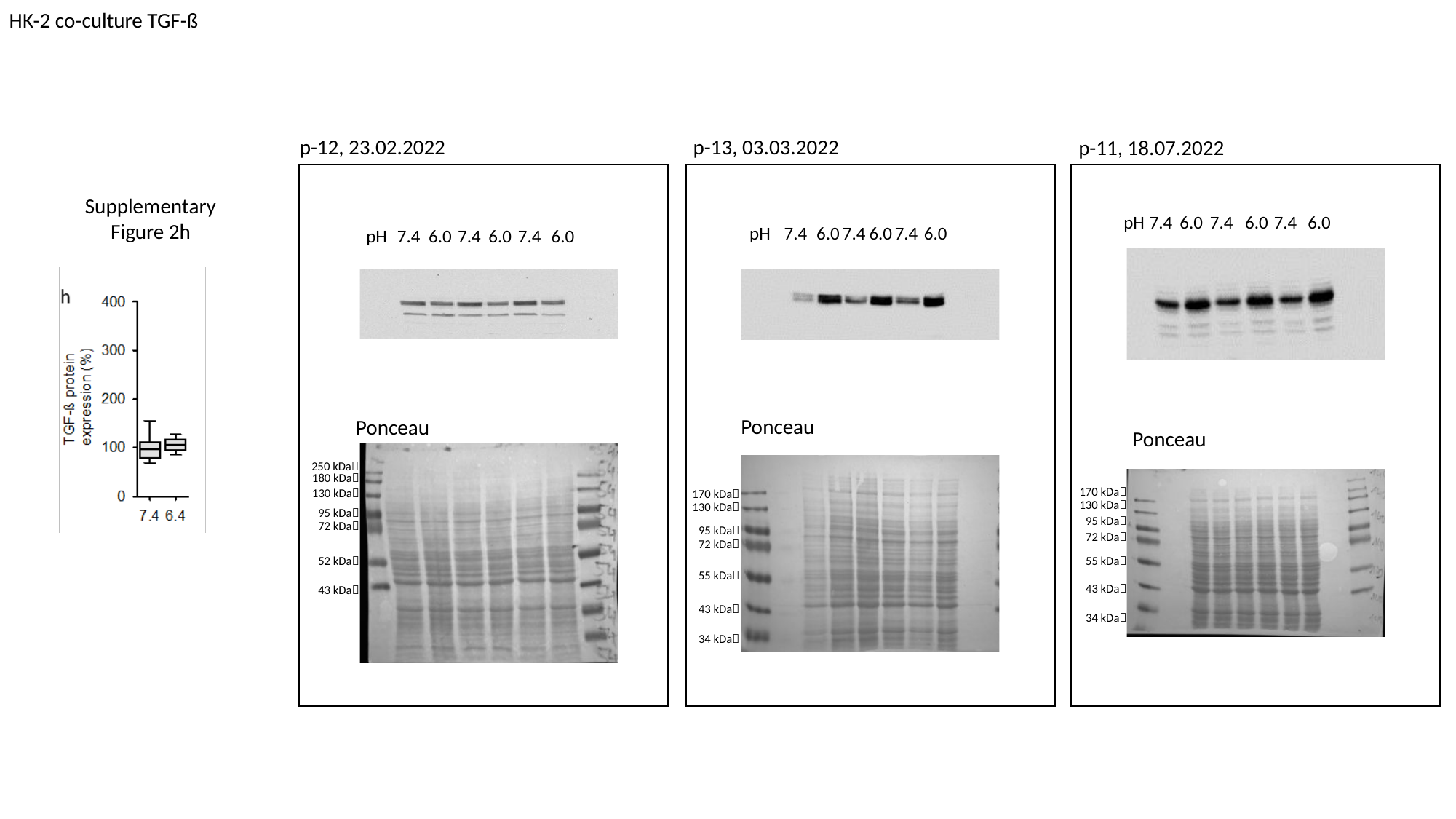

HK-2 co-culture TGF-ß
p-12, 23.02.2022
p-13, 03.03.2022
p-11, 18.07.2022
Supplementary Figure 2h
pH
7.4
6.0
7.4
6.0
7.4
6.0
pH
7.4
6.0
7.4
6.0
7.4
6.0
pH
7.4
6.0
7.4
6.0
7.4
6.0
Ponceau
Ponceau
Ponceau
250 kDa
180 kDa
130 kDa
95 kDa
72 kDa
52 kDa
43 kDa
170 kDa
130 kDa
95 kDa
72 kDa
55 kDa
43 kDa
34 kDa
170 kDa
130 kDa
95 kDa
72 kDa
55 kDa
43 kDa
34 kDa

## Slide 84
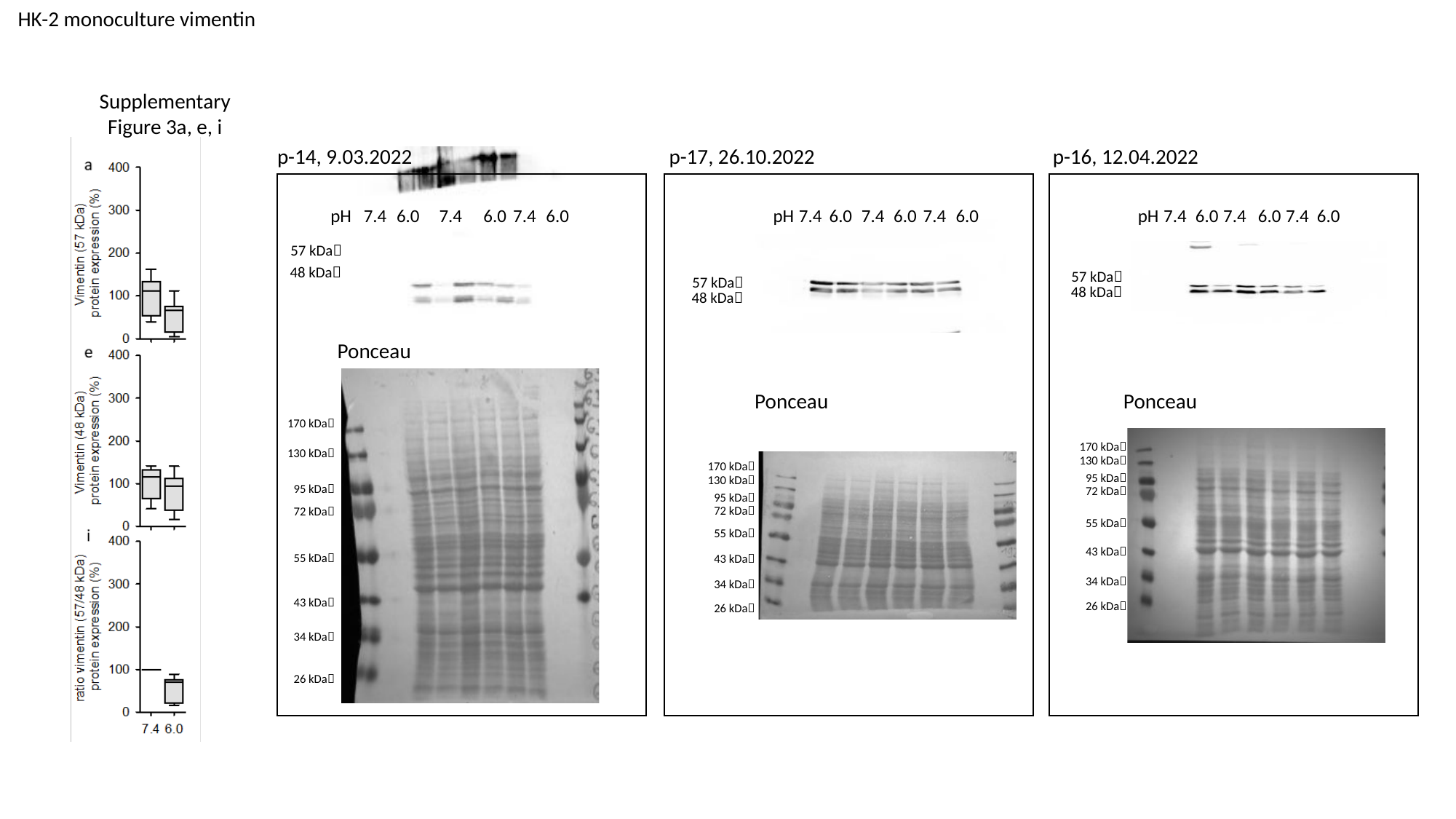

HK-2 monoculture vimentin
Supplementary Figure 3a, e, i
p-14, 9.03.2022
p-17, 26.10.2022
p-16, 12.04.2022
pH
7.4
6.0
7.4
6.0
7.4
6.0
pH
7.4
6.0
7.4
6.0
7.4
6.0
pH
7.4
6.0
7.4
6.0
7.4
6.0
57 kDa
48 kDa
57 kDa
57 kDa
48 kDa
48 kDa
Ponceau
Ponceau
Ponceau
170 kDa
130 kDa
95 kDa
72 kDa
55 kDa
43 kDa
34 kDa
26 kDa
170 kDa
130 kDa
95 kDa
72 kDa
55 kDa
43 kDa
34 kDa
26 kDa
170 kDa
130 kDa
95 kDa
72 kDa
55 kDa
43 kDa
34 kDa
26 kDa

## Slide 85
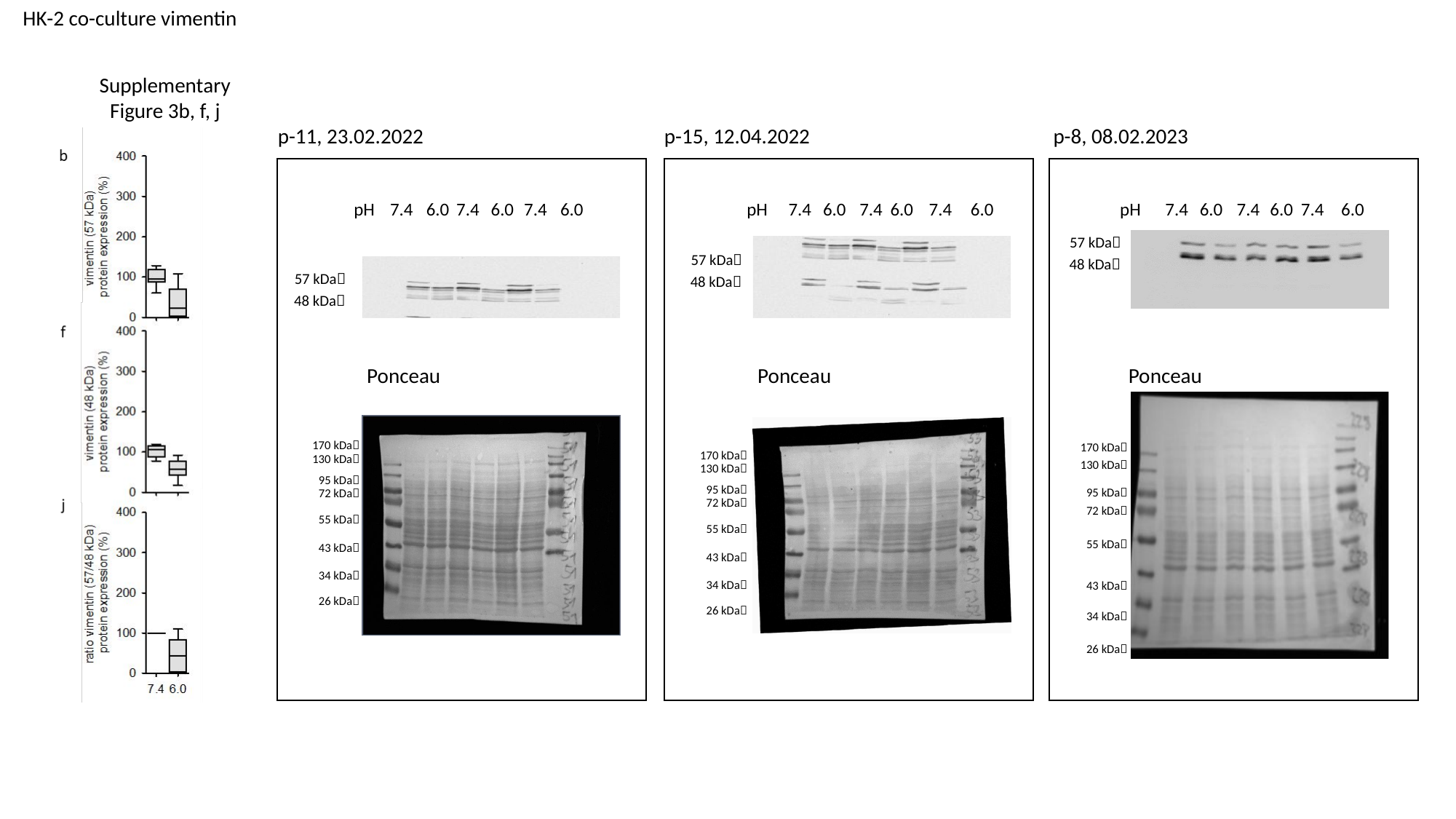

HK-2 co-culture vimentin
Supplementary Figure 3b, f, j
p-11, 23.02.2022
p-15, 12.04.2022
p-8, 08.02.2023
pH
7.4
6.0
7.4
6.0
7.4
6.0
pH
7.4
6.0
7.4
6.0
7.4
6.0
pH
7.4
6.0
7.4
6.0
7.4
6.0
57 kDa
57 kDa
48 kDa
57 kDa
48 kDa
48 kDa
Ponceau
Ponceau
Ponceau
170 kDa
130 kDa
95 kDa
72 kDa
55 kDa
43 kDa
34 kDa
26 kDa
170 kDa
130 kDa
95 kDa
72 kDa
55 kDa
43 kDa
34 kDa
26 kDa
170 kDa
130 kDa
95 kDa
72 kDa
55 kDa
43 kDa
34 kDa
26 kDa

## Slide 86
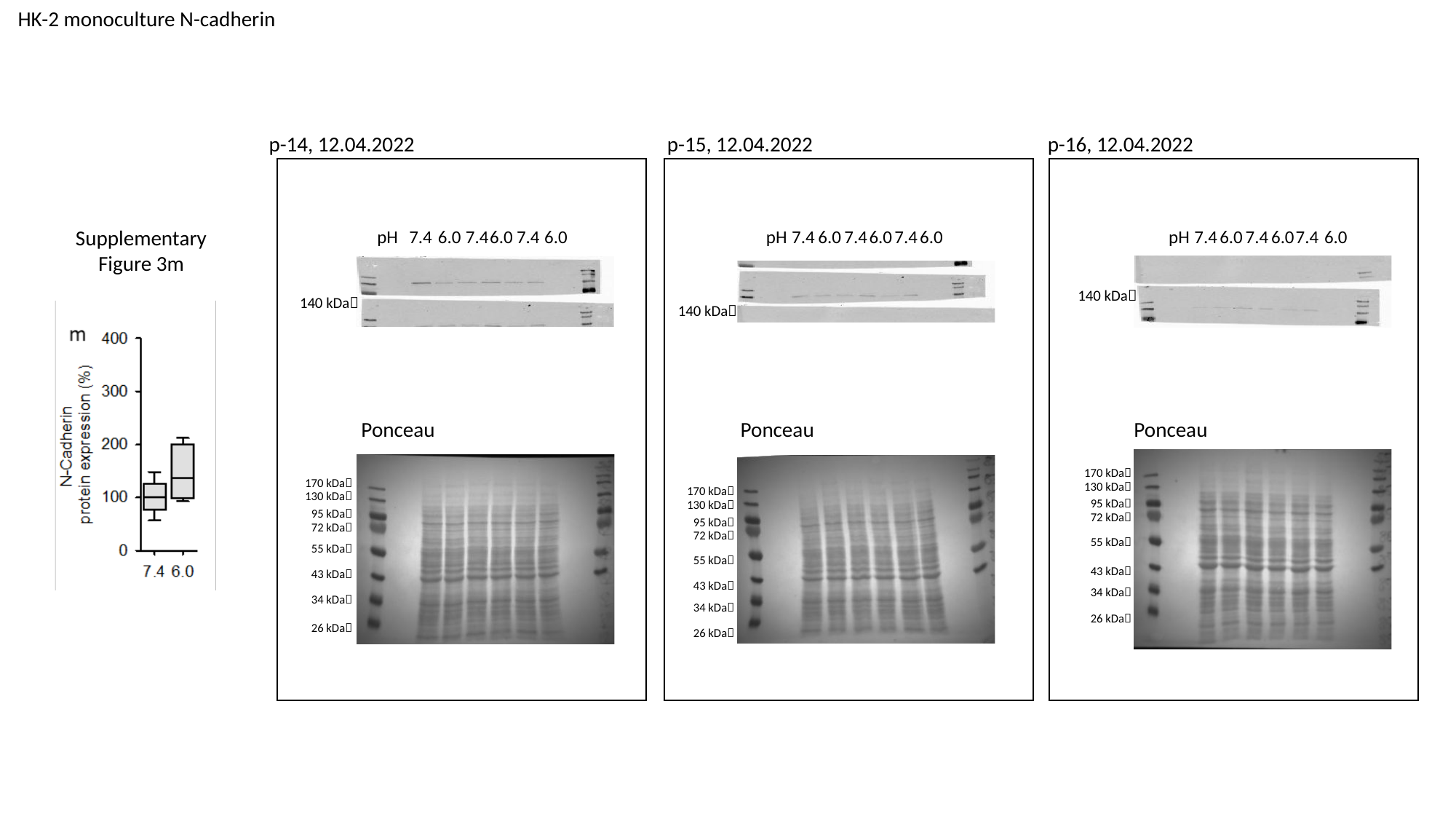

HK-2 monoculture N-cadherin
p-14, 12.04.2022
p-15, 12.04.2022
p-16, 12.04.2022
Supplementary Figure 3m
pH
7.4
6.0
7.4
6.0
7.4
6.0
pH
7.4
6.0
7.4
6.0
7.4
6.0
pH
7.4
6.0
7.4
6.0
7.4
6.0
140 kDa
140 kDa
140 kDa
Ponceau
Ponceau
Ponceau
170 kDa
130 kDa
95 kDa
72 kDa
55 kDa
43 kDa
34 kDa
26 kDa
170 kDa
130 kDa
95 kDa
72 kDa
55 kDa
43 kDa
34 kDa
26 kDa
170 kDa
130 kDa
95 kDa
72 kDa
55 kDa
43 kDa
34 kDa
26 kDa

## Slide 87
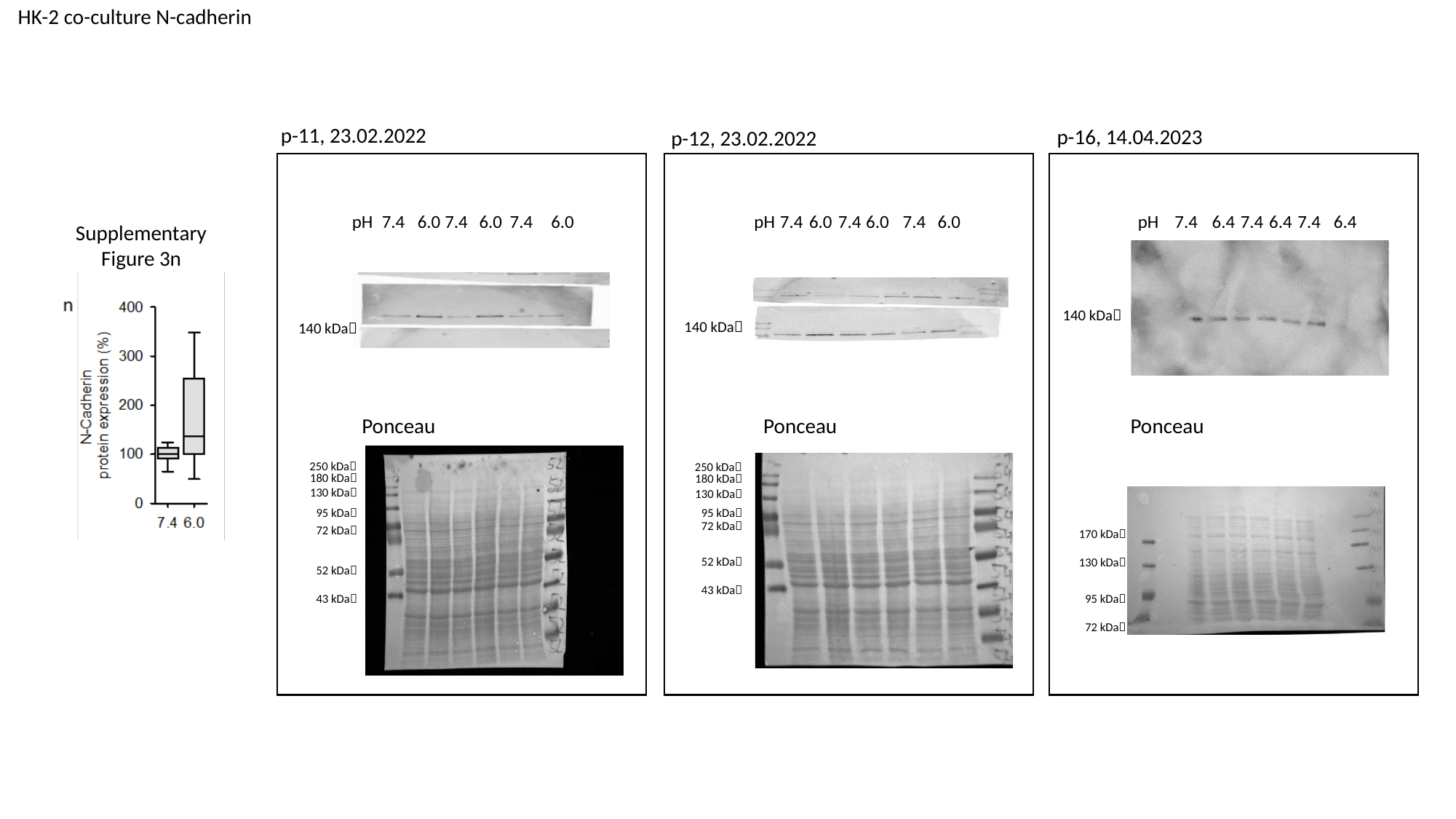

HK-2 co-culture N-cadherin
p-11, 23.02.2022
p-16, 14.04.2023
p-12, 23.02.2022
pH
7.4
6.0
7.4
6.0
7.4
6.0
pH
7.4
6.0
7.4
6.0
7.4
6.0
pH
7.4
6.4
7.4
6.4
7.4
6.4
Supplementary Figure 3n
140 kDa
140 kDa
140 kDa
Ponceau
Ponceau
Ponceau
250 kDa
180 kDa
130 kDa
95 kDa
72 kDa
52 kDa
43 kDa
250 kDa
180 kDa
130 kDa
95 kDa
72 kDa
52 kDa
43 kDa
170 kDa
130 kDa
95 kDa
72 kDa

## Slide 88
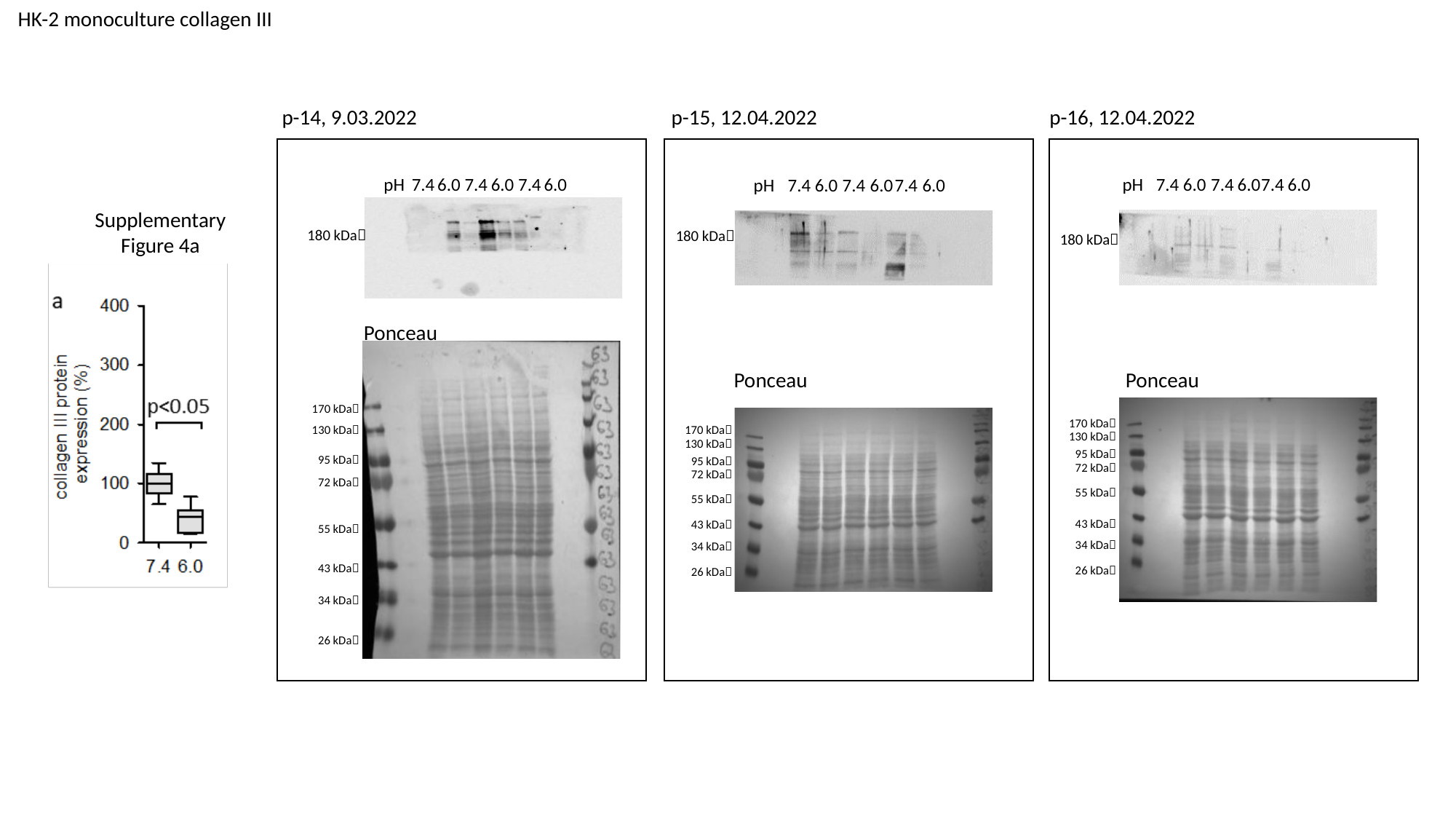

HK-2 monoculture collagen III
p-14, 9.03.2022
p-15, 12.04.2022
p-16, 12.04.2022
pH
7.4
6.0
7.4
6.0
7.4
6.0
pH
7.4
6.0
7.4
6.0
7.4
6.0
pH
7.4
6.0
7.4
6.0
7.4
6.0
Supplementary Figure 4a
180 kDa
180 kDa
180 kDa
Ponceau
Ponceau
Ponceau
170 kDa
130 kDa
95 kDa
72 kDa
55 kDa
43 kDa
34 kDa
26 kDa
170 kDa
130 kDa
95 kDa
72 kDa
55 kDa
43 kDa
34 kDa
26 kDa
170 kDa
130 kDa
95 kDa
72 kDa
55 kDa
43 kDa
34 kDa
26 kDa

## Slide 89
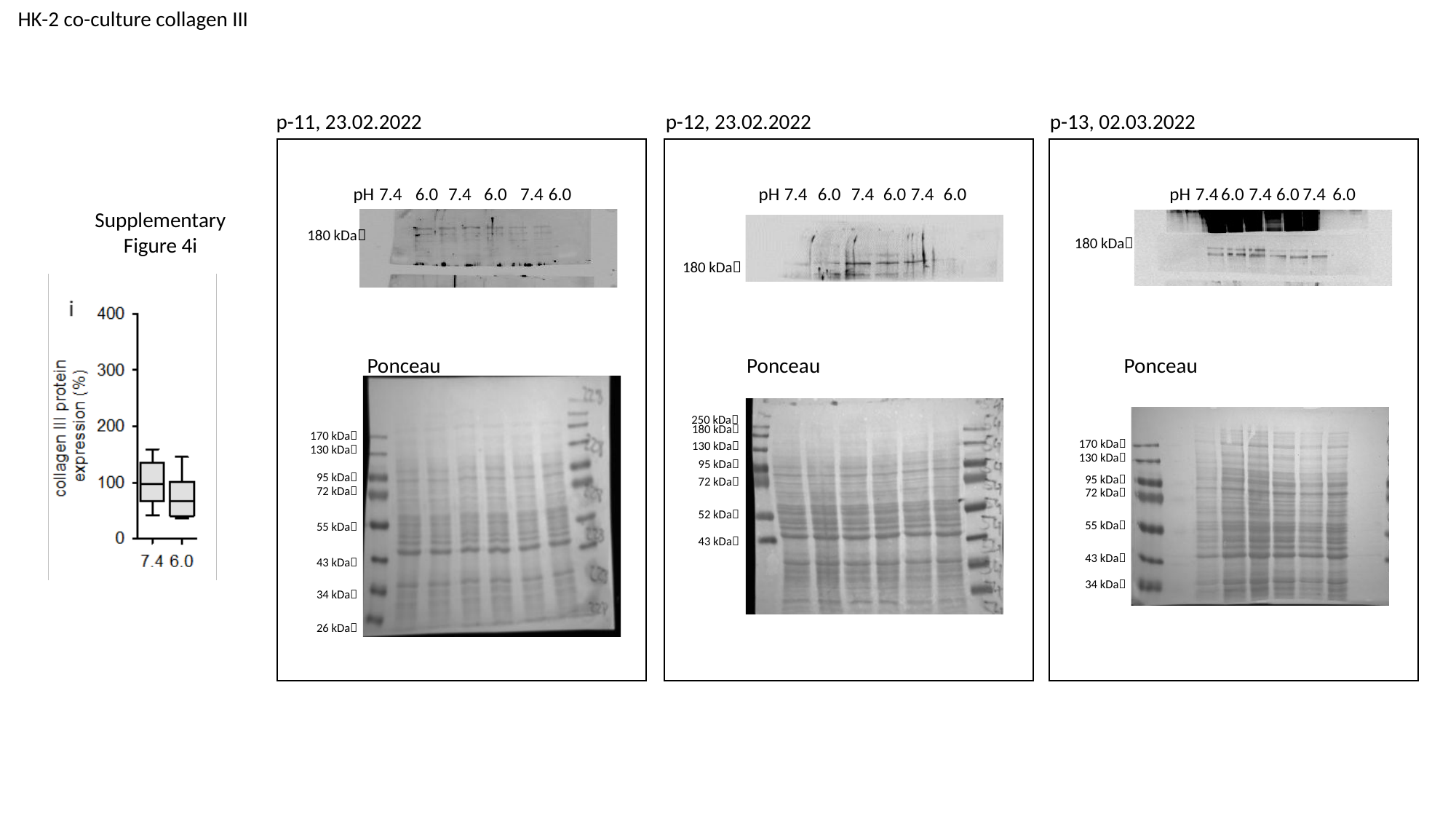

HK-2 co-culture collagen III
p-11, 23.02.2022
p-12, 23.02.2022
p-13, 02.03.2022
pH
7.4
6.0
7.4
6.0
7.4
6.0
pH
7.4
6.0
7.4
6.0
7.4
6.0
pH
7.4
6.0
7.4
6.0
7.4
6.0
Supplementary Figure 4i
180 kDa
180 kDa
180 kDa
Ponceau
Ponceau
Ponceau
250 kDa
180 kDa
130 kDa
95 kDa
72 kDa
52 kDa
43 kDa
170 kDa
130 kDa
95 kDa
72 kDa
55 kDa
43 kDa
34 kDa
26 kDa
170 kDa
130 kDa
95 kDa
72 kDa
55 kDa
43 kDa
34 kDa

## Slide 90
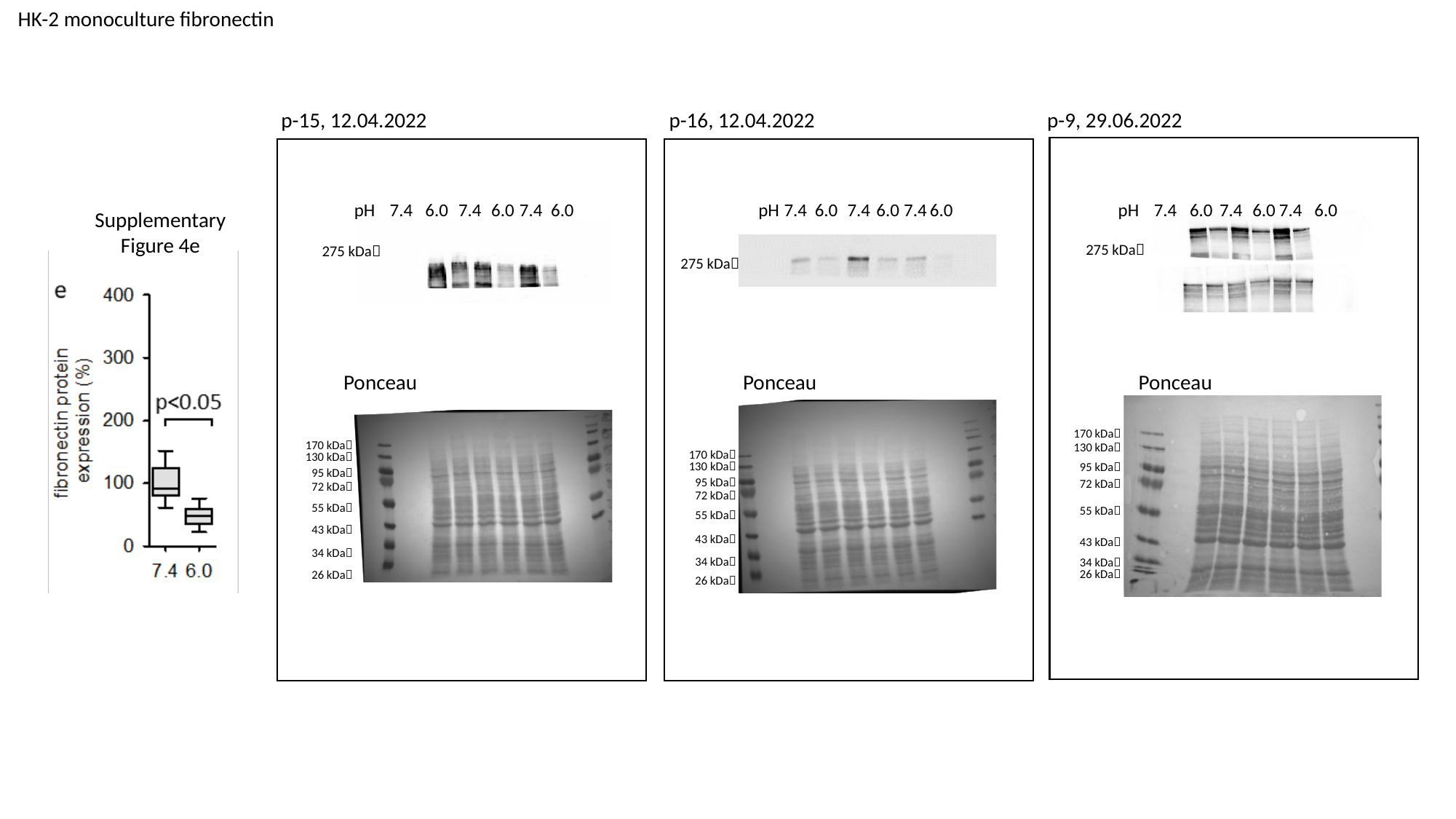

HK-2 monoculture fibronectin
p-15, 12.04.2022
p-16, 12.04.2022
p-9, 29.06.2022
pH
7.4
6.0
7.4
6.0
7.4
6.0
pH
7.4
6.0
7.4
6.0
7.4
6.0
pH
7.4
6.0
7.4
6.0
7.4
6.0
Supplementary Figure 4e
275 kDa
275 kDa
275 kDa
Ponceau
Ponceau
Ponceau
170 kDa
130 kDa
95 kDa
72 kDa
55 kDa
43 kDa
34 kDa
26 kDa
170 kDa
130 kDa
95 kDa
72 kDa
55 kDa
43 kDa
34 kDa
26 kDa
170 kDa
130 kDa
95 kDa
72 kDa
55 kDa
43 kDa
34 kDa
26 kDa

## Slide 91
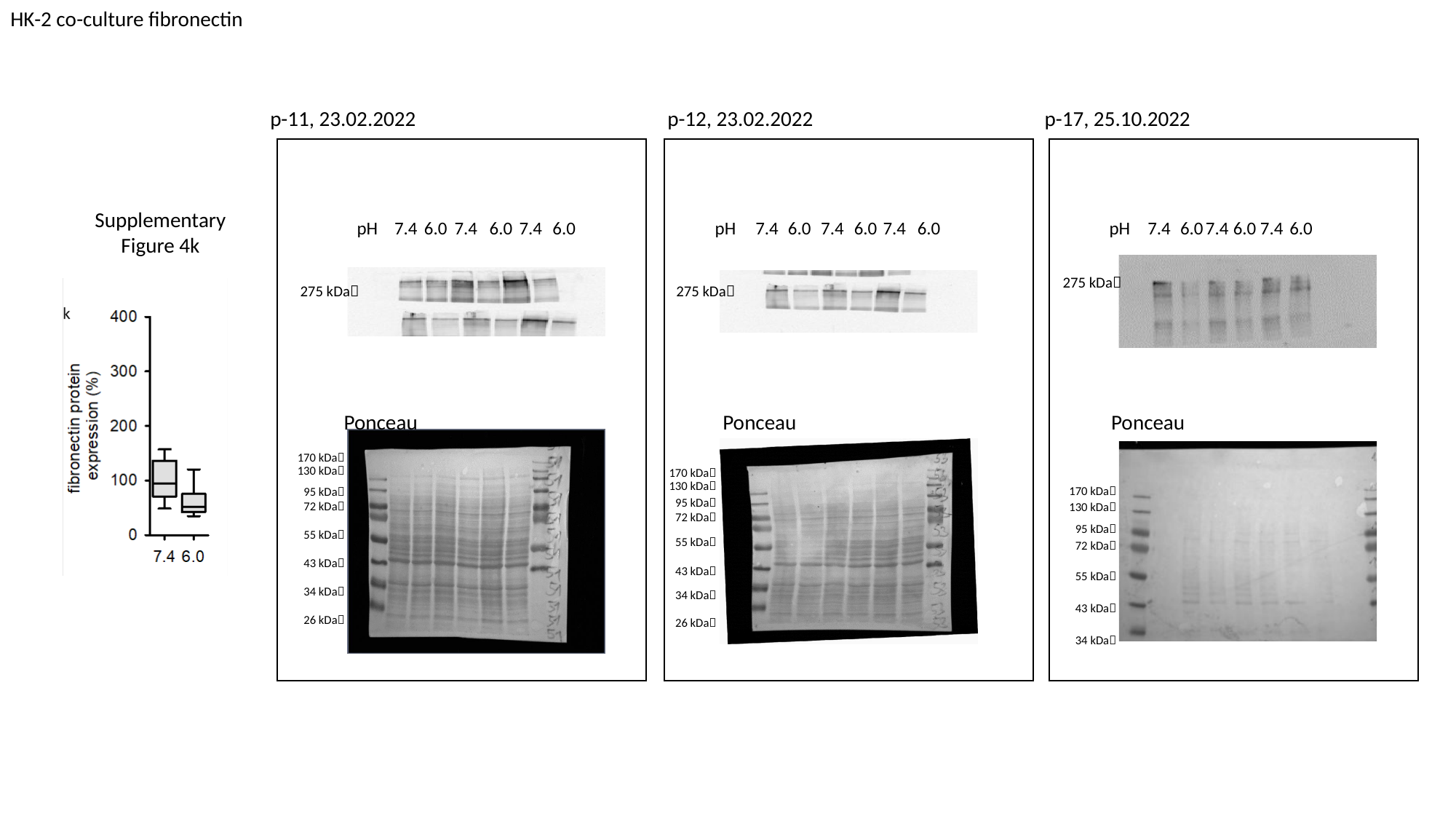

HK-2 co-culture fibronectin
p-11, 23.02.2022
p-12, 23.02.2022
p-17, 25.10.2022
Supplementary Figure 4k
pH
7.4
6.0
7.4
6.0
7.4
6.0
pH
7.4
6.0
7.4
6.0
7.4
6.0
pH
7.4
6.0
7.4
6.0
7.4
6.0
275 kDa
275 kDa
275 kDa
Ponceau
Ponceau
Ponceau
170 kDa
130 kDa
95 kDa
72 kDa
55 kDa
43 kDa
34 kDa
26 kDa
170 kDa
130 kDa
95 kDa
72 kDa
55 kDa
43 kDa
34 kDa
26 kDa
170 kDa
130 kDa
95 kDa
72 kDa
55 kDa
43 kDa
34 kDa

## Slide 92
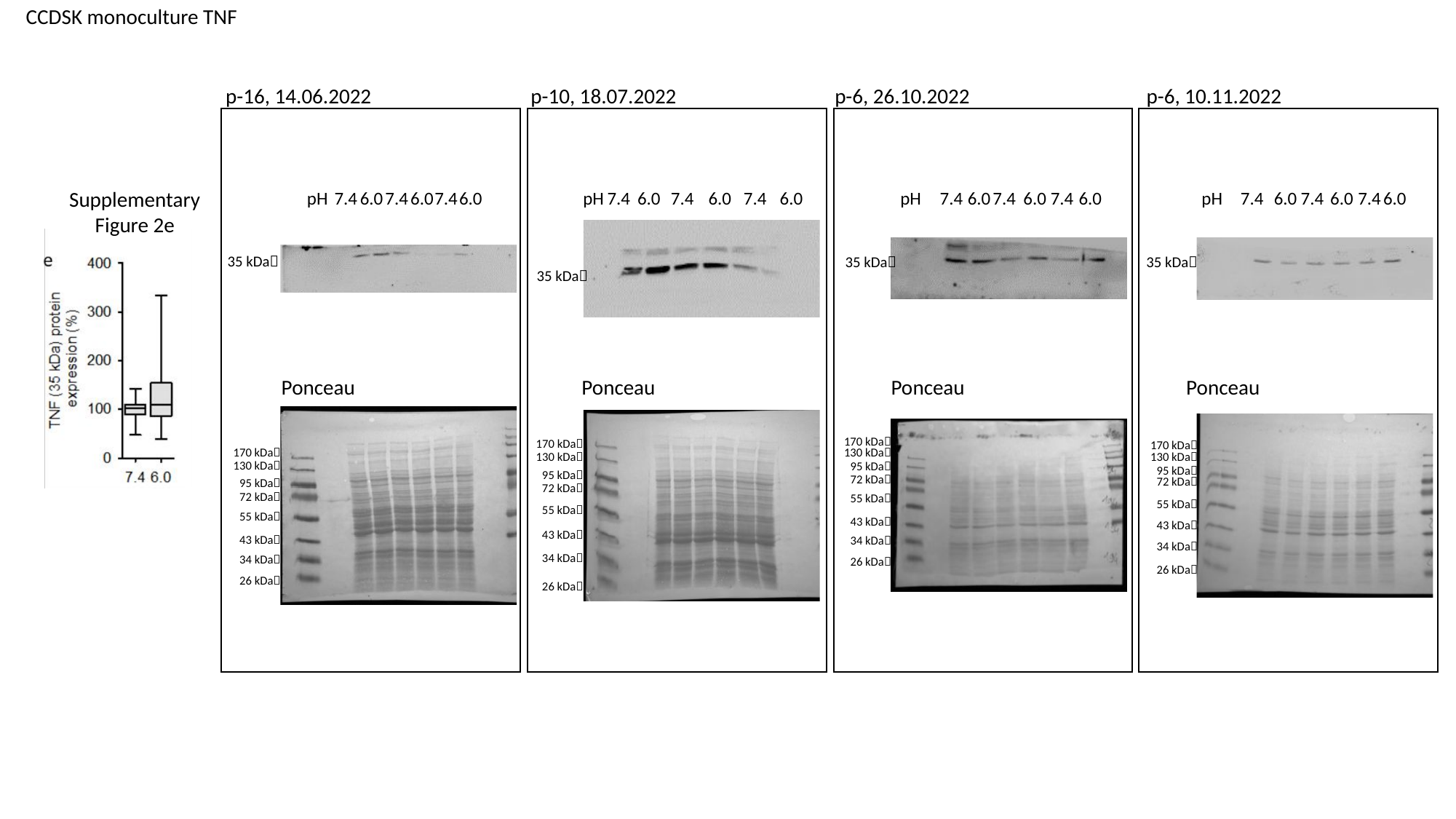

CCDSK monoculture TNF
p-16, 14.06.2022
p-10, 18.07.2022
p-6, 26.10.2022
p-6, 10.11.2022
Supplementary Figure 2e
pH
7.4
6.0
7.4
6.0
7.4
6.0
pH
7.4
6.0
7.4
6.0
7.4
6.0
pH
7.4
6.0
7.4
6.0
7.4
6.0
pH
7.4
6.0
7.4
6.0
7.4
6.0
35 kDa
35 kDa
35 kDa
35 kDa
Ponceau
Ponceau
Ponceau
Ponceau
170 kDa
130 kDa
95 kDa
72 kDa
55 kDa
43 kDa
34 kDa
26 kDa
170 kDa
130 kDa
95 kDa
72 kDa
55 kDa
43 kDa
34 kDa
26 kDa
170 kDa
130 kDa
95 kDa
72 kDa
55 kDa
43 kDa
34 kDa
26 kDa
170 kDa
130 kDa
95 kDa
72 kDa
55 kDa
43 kDa
34 kDa
26 kDa

## Slide 93
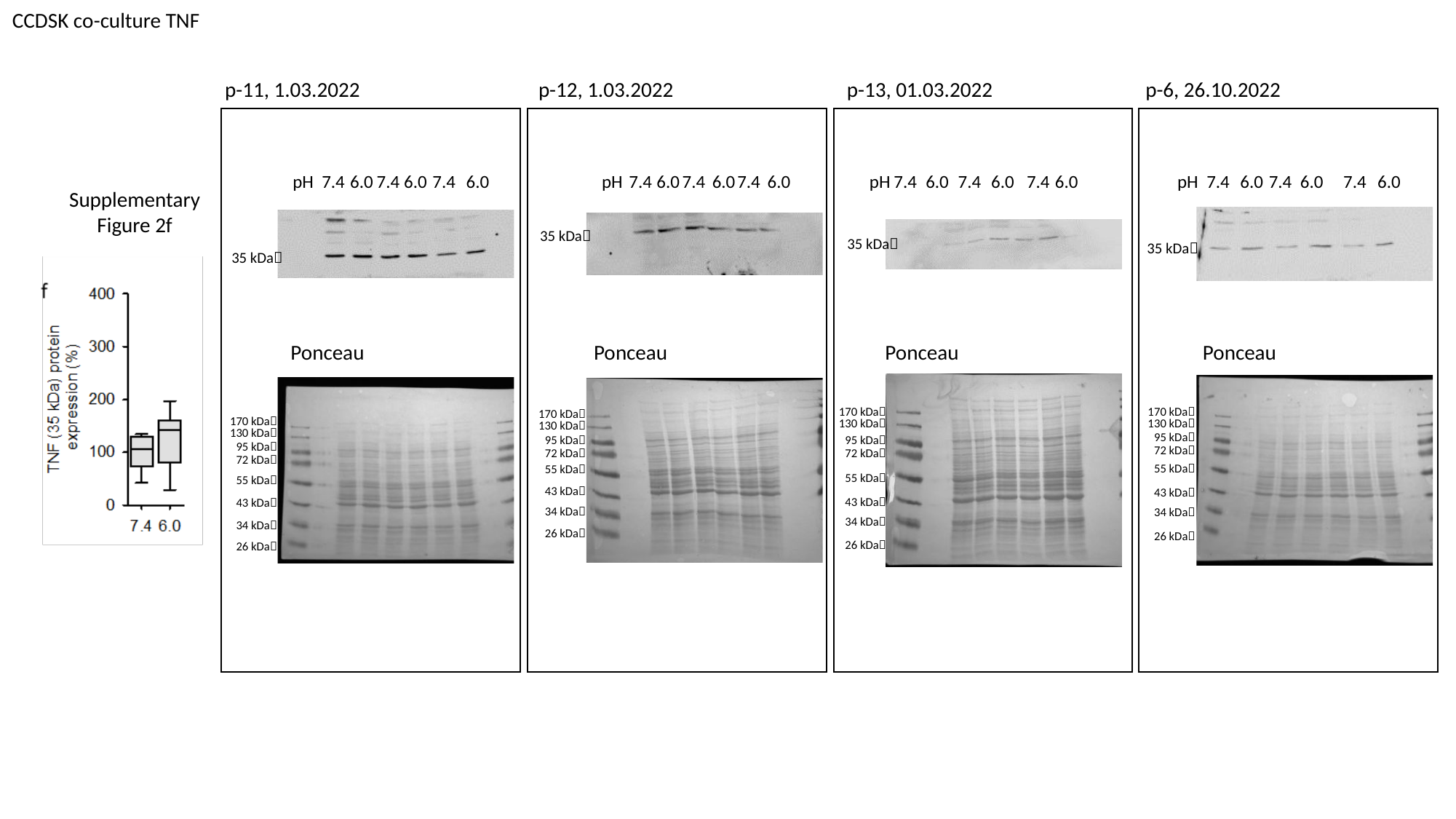

CCDSK co-culture TNF
p-11, 1.03.2022
p-12, 1.03.2022
p-13, 01.03.2022
p-6, 26.10.2022
pH
7.4
6.0
7.4
6.0
7.4
6.0
pH
7.4
6.0
7.4
6.0
7.4
6.0
pH
7.4
6.0
7.4
6.0
7.4
6.0
pH
7.4
6.0
7.4
6.0
7.4
6.0
Supplementary Figure 2f
35 kDa
35 kDa
35 kDa
35 kDa
Ponceau
Ponceau
Ponceau
Ponceau
170 kDa
130 kDa
95 kDa
72 kDa
55 kDa
43 kDa
34 kDa
26 kDa
170 kDa
130 kDa
95 kDa
72 kDa
55 kDa
43 kDa
34 kDa
26 kDa
170 kDa
130 kDa
95 kDa
72 kDa
55 kDa
43 kDa
34 kDa
26 kDa
170 kDa
130 kDa
95 kDa
72 kDa
55 kDa
43 kDa
34 kDa
26 kDa

## Slide 94
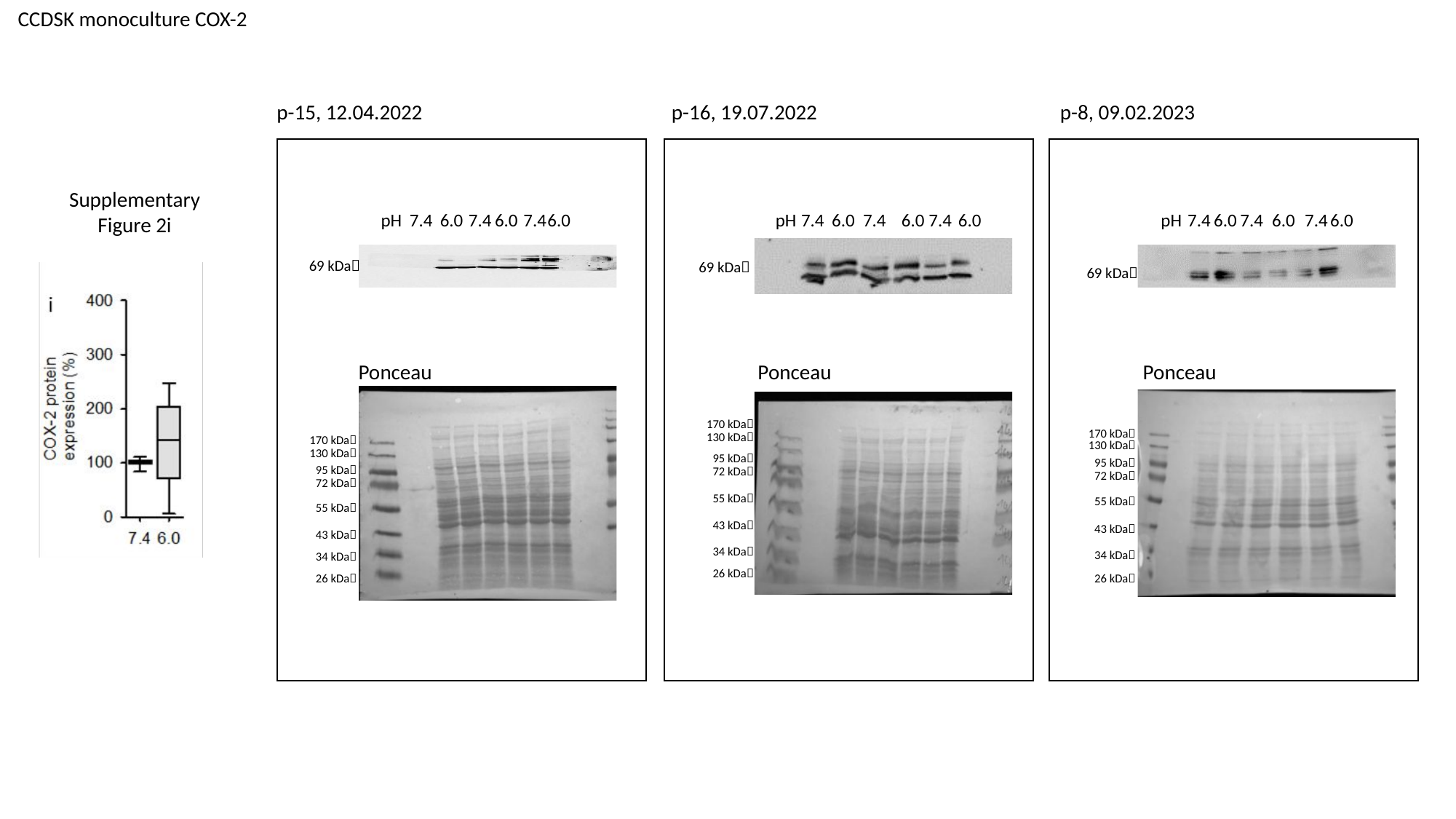

CCDSK monoculture COX-2
p-15, 12.04.2022
p-16, 19.07.2022
p-8, 09.02.2023
Supplementary Figure 2i
pH
7.4
6.0
7.4
6.0
7.4
6.0
pH
7.4
6.0
7.4
6.0
7.4
6.0
pH
7.4
6.0
7.4
6.0
7.4
6.0
69 kDa
69 kDa
69 kDa
Ponceau
Ponceau
Ponceau
170 kDa
130 kDa
95 kDa
72 kDa
55 kDa
43 kDa
34 kDa
26 kDa
170 kDa
130 kDa
95 kDa
72 kDa
55 kDa
43 kDa
34 kDa
26 kDa
170 kDa
130 kDa
95 kDa
72 kDa
55 kDa
43 kDa
34 kDa
26 kDa

## Slide 95
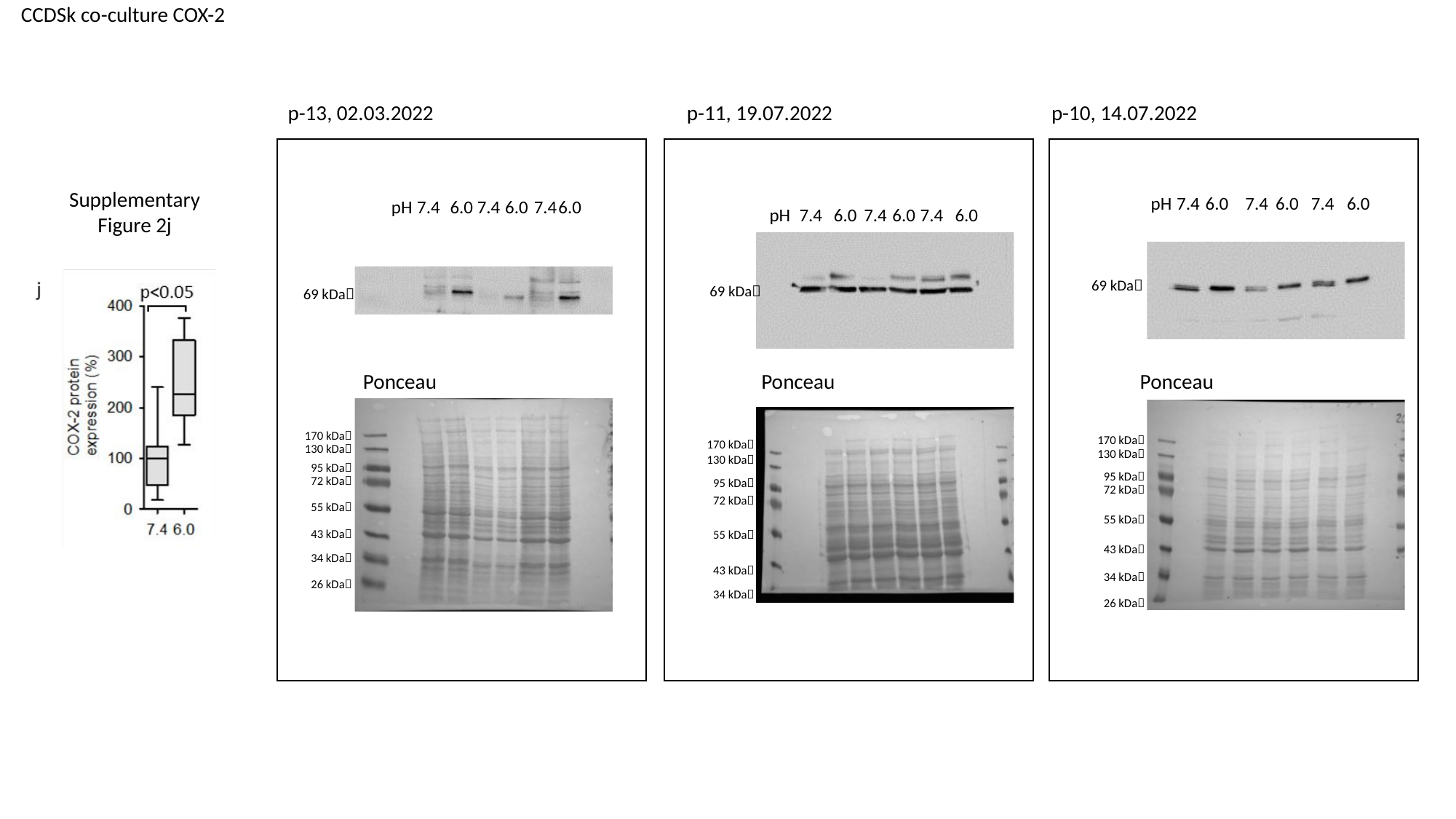

CCDSk co-culture COX-2
p-13, 02.03.2022
p-11, 19.07.2022
p-10, 14.07.2022
Supplementary Figure 2j
pH
7.4
6.0
7.4
6.0
7.4
6.0
pH
7.4
6.0
7.4
6.0
7.4
6.0
pH
7.4
6.0
7.4
6.0
7.4
6.0
69 kDa
69 kDa
69 kDa
Ponceau
Ponceau
Ponceau
170 kDa
130 kDa
95 kDa
72 kDa
55 kDa
43 kDa
34 kDa
26 kDa
170 kDa
130 kDa
95 kDa
72 kDa
55 kDa
43 kDa
34 kDa
26 kDa
170 kDa
130 kDa
95 kDa
72 kDa
55 kDa
43 kDa
34 kDa

## Slide 96
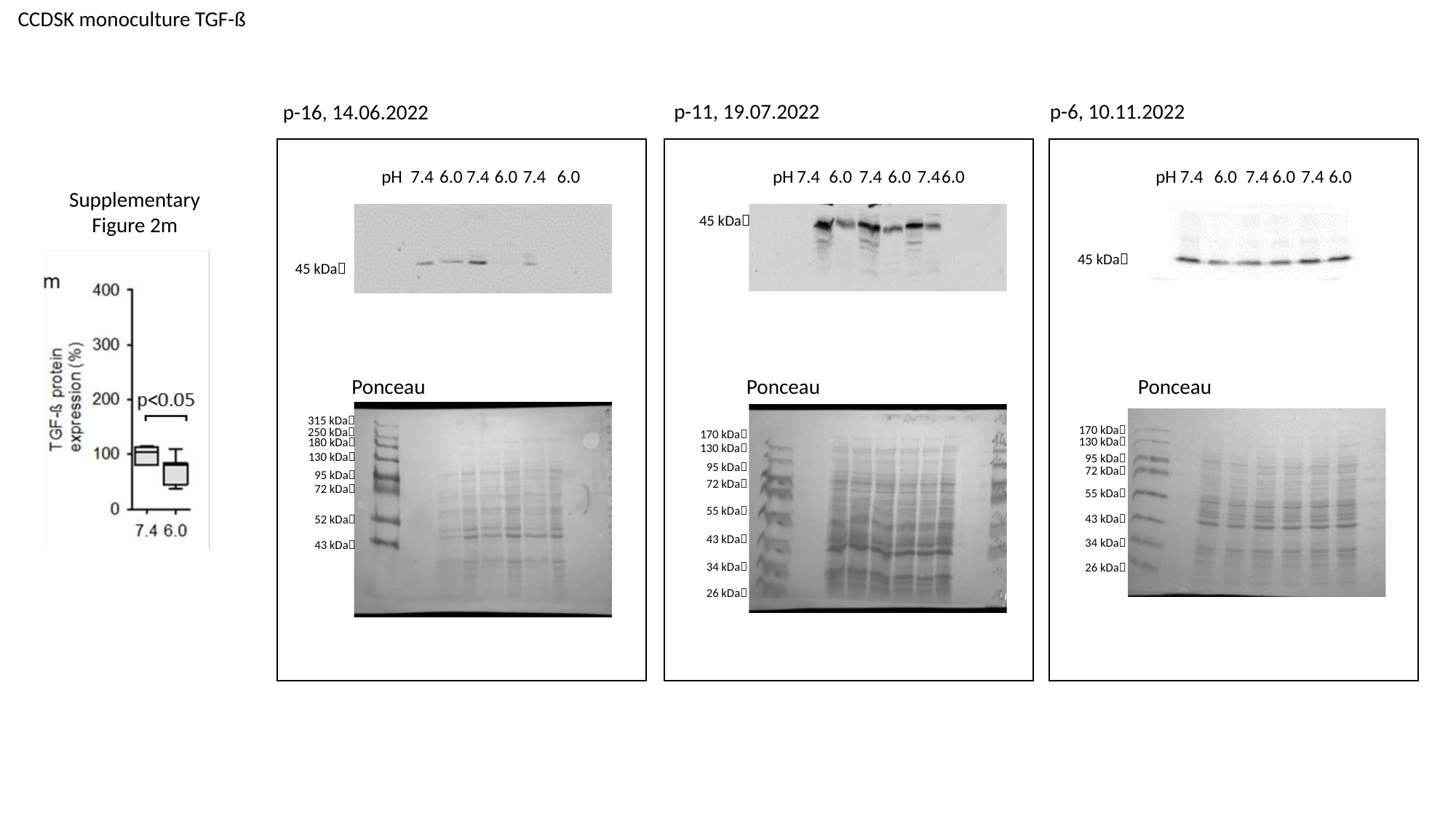

CCDSK monoculture TGF-ß
p-11, 19.07.2022
p-6, 10.11.2022
p-16, 14.06.2022
pH
7.4
6.0
7.4
6.0
7.4
6.0
pH
7.4
6.0
7.4
6.0
7.4
6.0
pH
7.4
6.0
7.4
6.0
7.4
6.0
Supplementary Figure 2m
45 kDa
45 kDa
45 kDa
Ponceau
Ponceau
Ponceau
315 kDa
250 kDa
180 kDa
130 kDa
95 kDa
72 kDa
52 kDa
43 kDa
170 kDa
130 kDa
95 kDa
72 kDa
55 kDa
43 kDa
34 kDa
26 kDa
170 kDa
130 kDa
95 kDa
72 kDa
55 kDa
43 kDa
34 kDa
26 kDa

## Slide 97
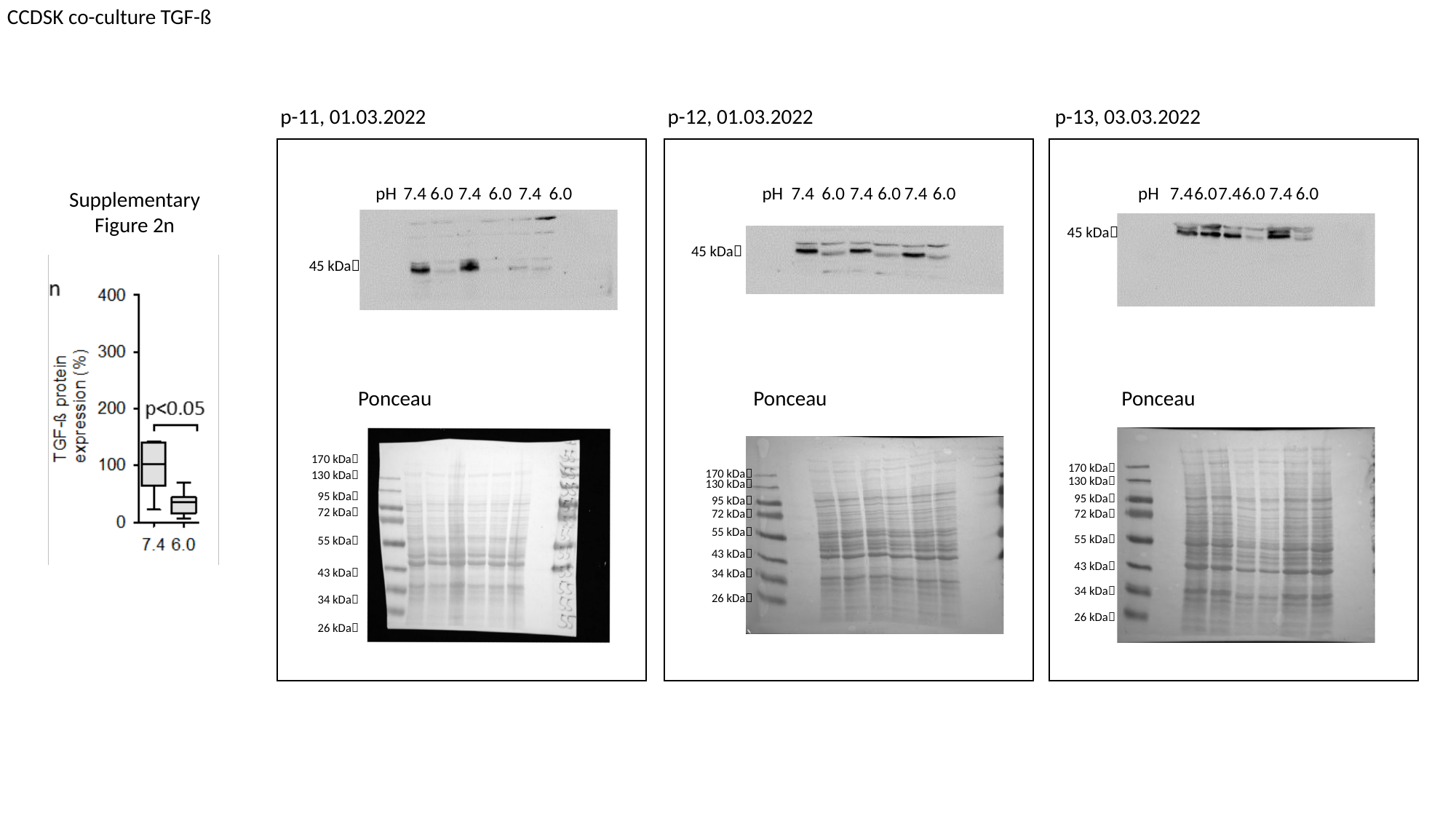

CCDSK co-culture TGF-ß
p-11, 01.03.2022
p-12, 01.03.2022
p-13, 03.03.2022
pH
7.4
6.0
7.4
6.0
7.4
6.0
pH
7.4
6.0
7.4
6.0
7.4
6.0
pH
7.4
6.0
7.4
6.0
7.4
6.0
Supplementary Figure 2n
45 kDa
45 kDa
45 kDa
Ponceau
Ponceau
Ponceau
170 kDa
130 kDa
95 kDa
72 kDa
55 kDa
43 kDa
34 kDa
26 kDa
170 kDa
130 kDa
95 kDa
72 kDa
55 kDa
43 kDa
34 kDa
26 kDa
170 kDa
130 kDa
95 kDa
72 kDa
55 kDa
43 kDa
34 kDa
26 kDa

## Slide 98
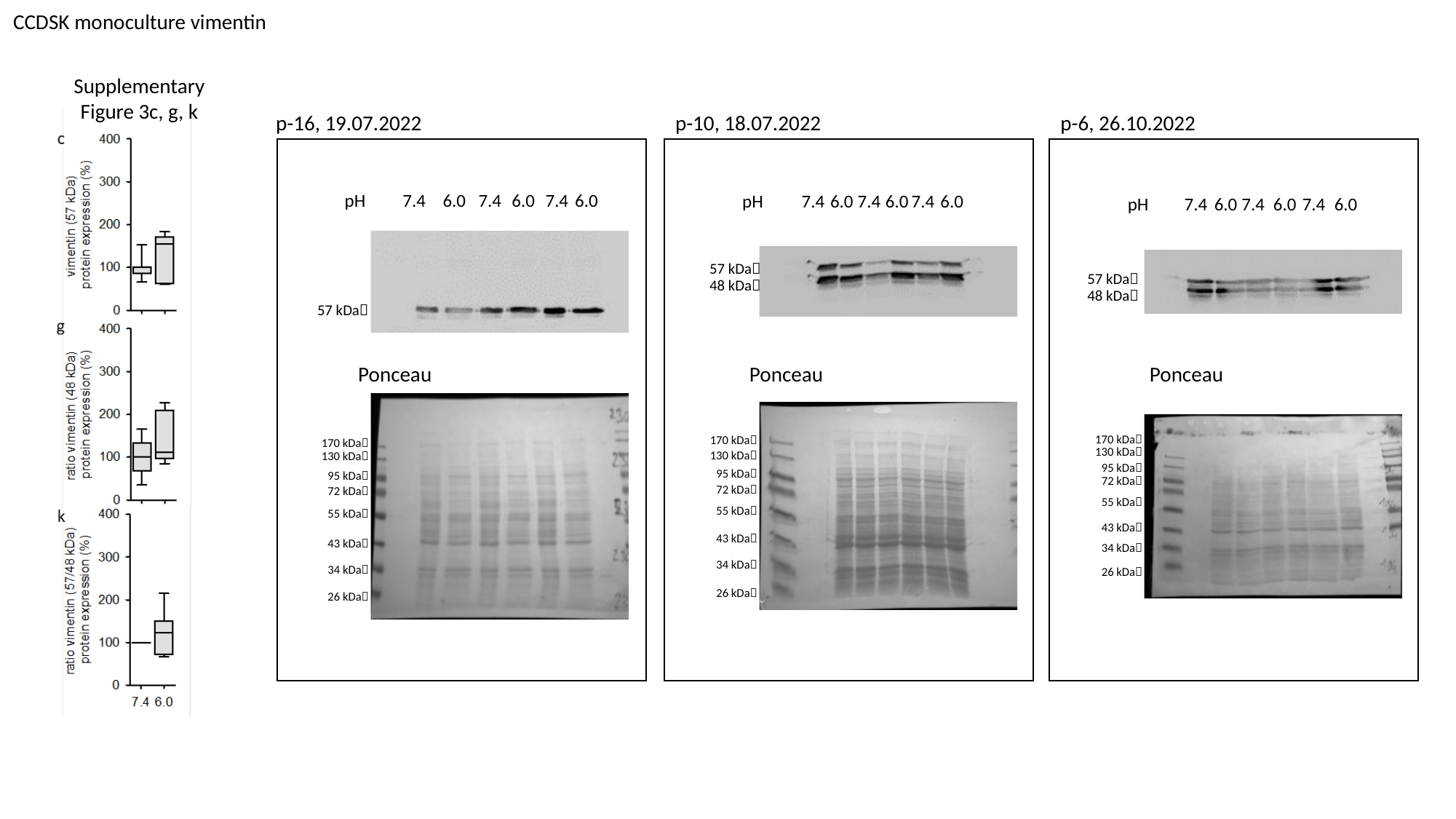

CCDSK monoculture vimentin
Supplementary Figure 3c, g, k
p-16, 19.07.2022
p-10, 18.07.2022
p-6, 26.10.2022
pH
7.4
6.0
7.4
6.0
7.4
6.0
pH
7.4
6.0
7.4
6.0
7.4
6.0
pH
7.4
6.0
7.4
6.0
7.4
6.0
57 kDa
57 kDa
48 kDa
48 kDa
57 kDa
Ponceau
Ponceau
Ponceau
170 kDa
130 kDa
95 kDa
72 kDa
55 kDa
43 kDa
34 kDa
26 kDa
170 kDa
130 kDa
95 kDa
72 kDa
55 kDa
43 kDa
34 kDa
26 kDa
170 kDa
130 kDa
95 kDa
72 kDa
55 kDa
43 kDa
34 kDa
26 kDa

## Slide 99
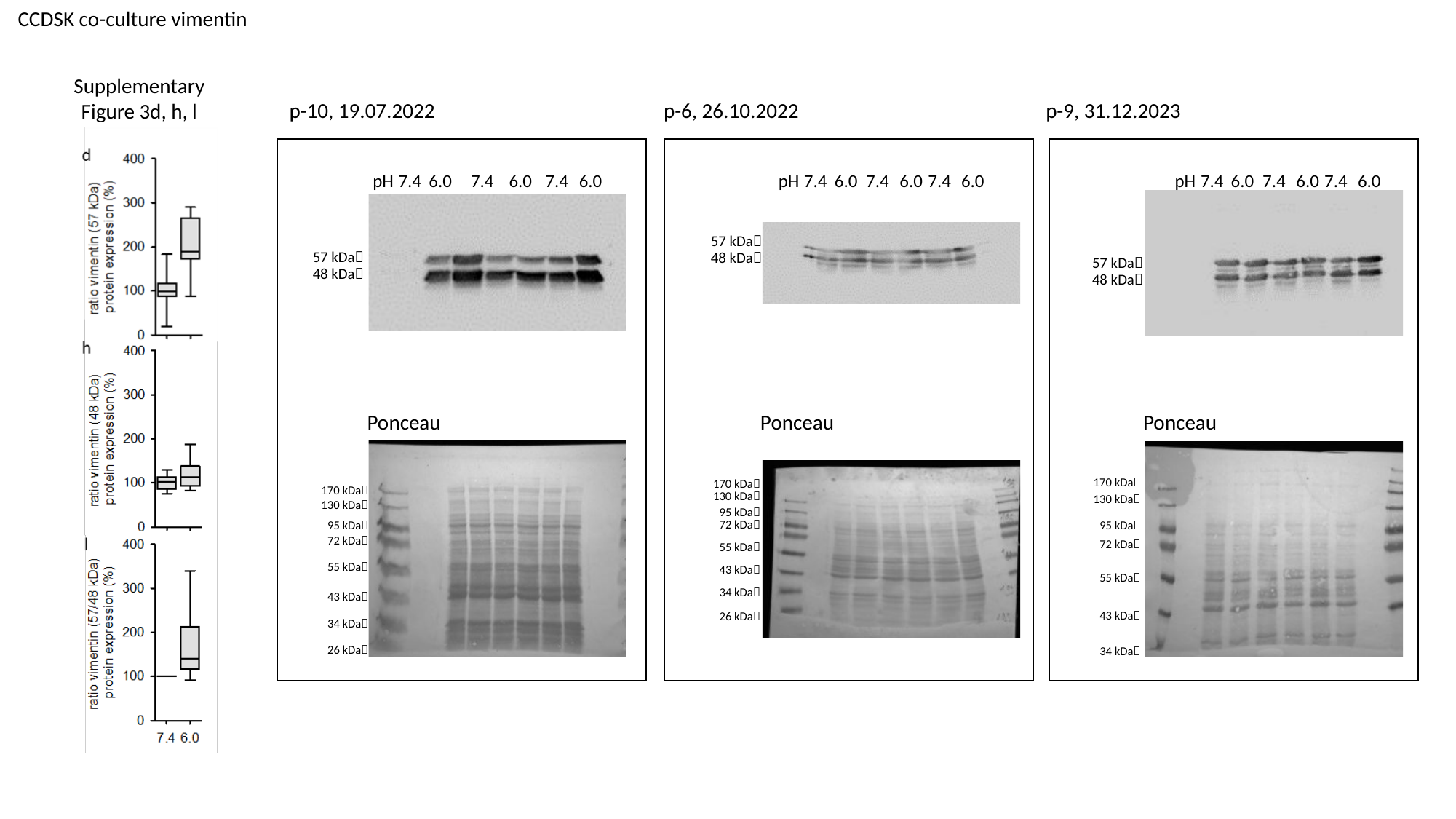

CCDSK co-culture vimentin
Supplementary Figure 3d, h, l
p-6, 26.10.2022
p-9, 31.12.2023
p-10, 19.07.2022
pH
7.4
6.0
7.4
6.0
7.4
6.0
pH
7.4
6.0
7.4
6.0
7.4
6.0
pH
7.4
6.0
7.4
6.0
7.4
6.0
57 kDa
57 kDa
48 kDa
57 kDa
48 kDa
48 kDa
Ponceau
Ponceau
Ponceau
170 kDa
130 kDa
95 kDa
72 kDa
55 kDa
43 kDa
34 kDa
170 kDa
130 kDa
95 kDa
72 kDa
55 kDa
43 kDa
34 kDa
26 kDa
170 kDa
130 kDa
95 kDa
72 kDa
55 kDa
43 kDa
34 kDa
26 kDa

## Slide 100
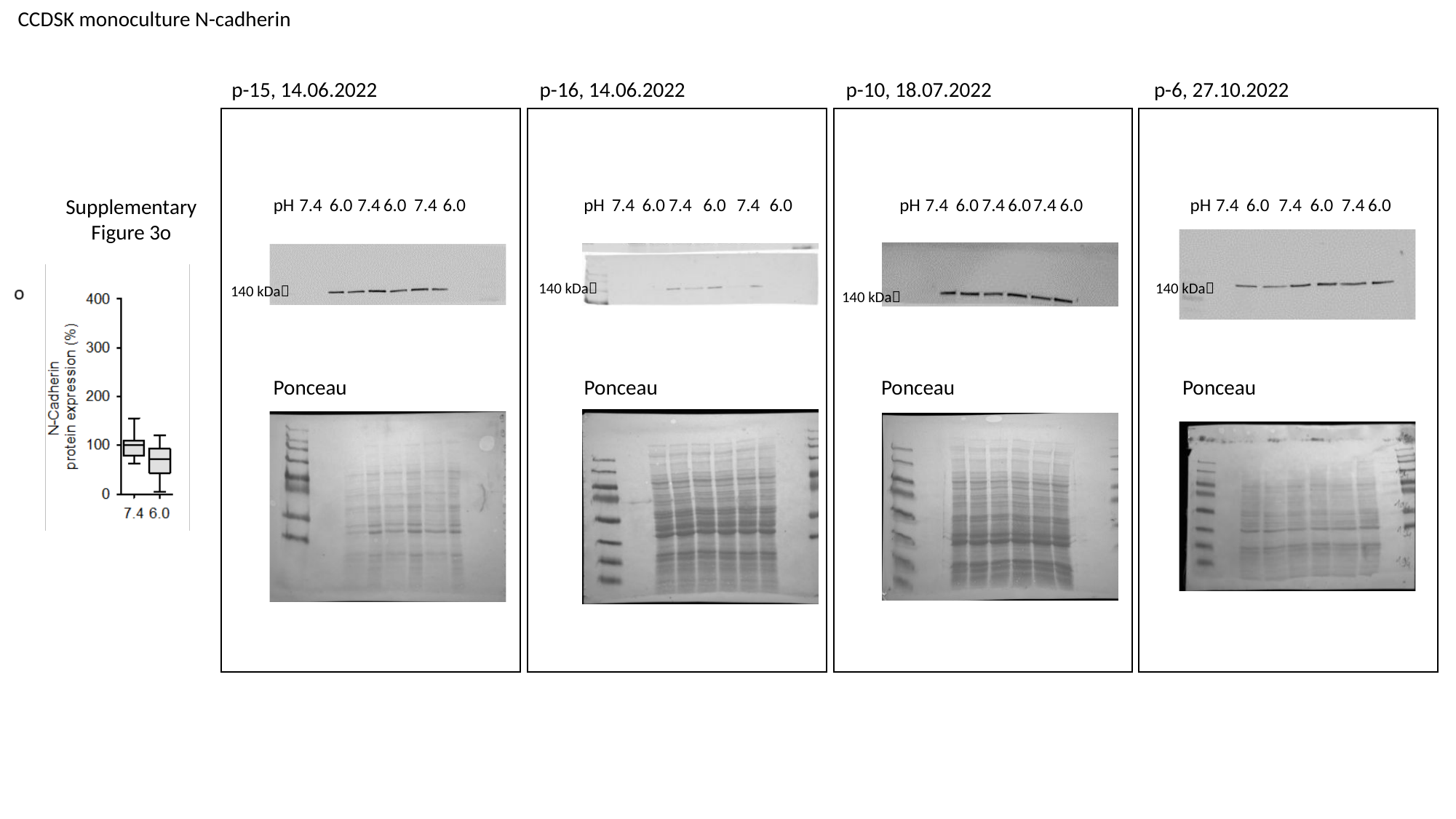

CCDSK monoculture N-cadherin
p-15, 14.06.2022
p-16, 14.06.2022
p-10, 18.07.2022
p-6, 27.10.2022
Supplementary Figure 3o
pH
7.4
6.0
7.4
6.0
7.4
6.0
pH
7.4
6.0
7.4
6.0
7.4
6.0
pH
7.4
6.0
7.4
6.0
7.4
6.0
pH
7.4
6.0
7.4
6.0
7.4
6.0
140 kDa
140 kDa
140 kDa
140 kDa
Ponceau
Ponceau
Ponceau
Ponceau

## Slide 101
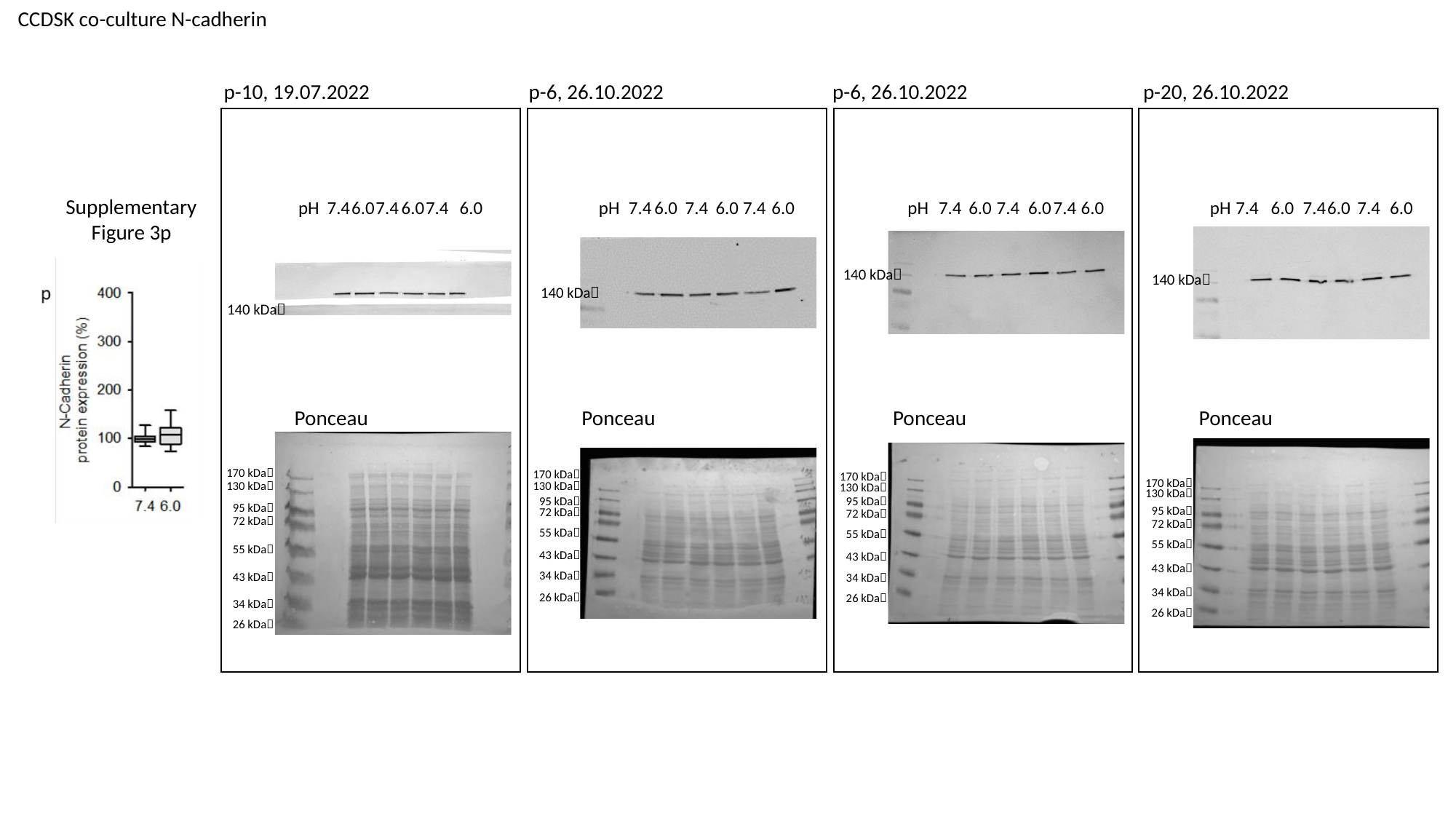

CCDSK co-culture N-cadherin
p-10, 19.07.2022
p-6, 26.10.2022
p-6, 26.10.2022
p-20, 26.10.2022
Supplementary Figure 3p
pH
7.4
6.0
7.4
6.0
7.4
6.0
pH
7.4
6.0
7.4
6.0
7.4
6.0
pH
7.4
6.0
7.4
6.0
7.4
6.0
pH
7.4
6.0
7.4
6.0
7.4
6.0
140 kDa
140 kDa
140 kDa
140 kDa
Ponceau
Ponceau
Ponceau
Ponceau
170 kDa
130 kDa
95 kDa
72 kDa
55 kDa
43 kDa
34 kDa
26 kDa
170 kDa
130 kDa
95 kDa
72 kDa
55 kDa
43 kDa
34 kDa
26 kDa
170 kDa
130 kDa
95 kDa
72 kDa
55 kDa
43 kDa
34 kDa
26 kDa
170 kDa
130 kDa
95 kDa
72 kDa
55 kDa
43 kDa
34 kDa
26 kDa

## Slide 102
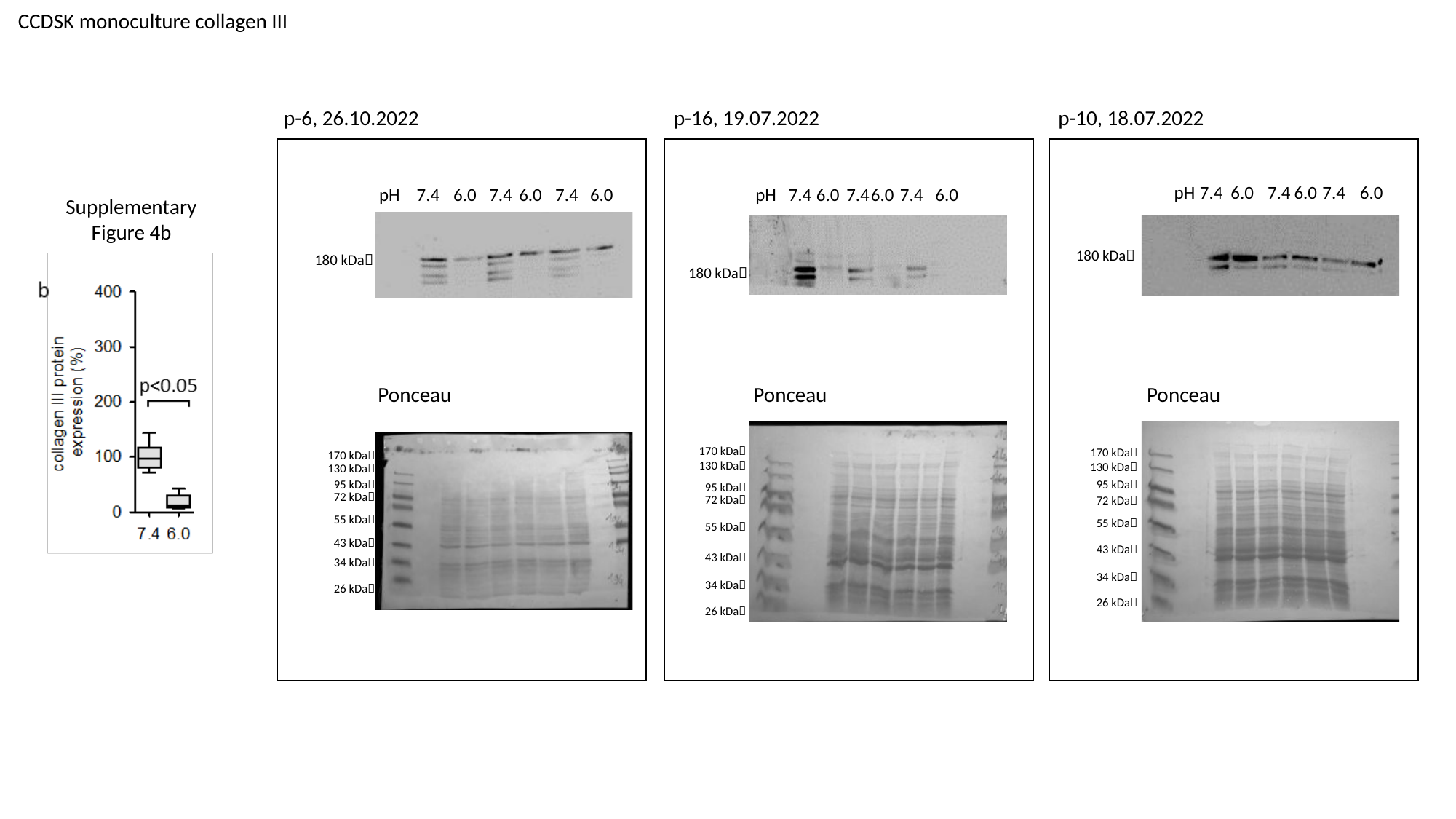

CCDSK monoculture collagen III
p-6, 26.10.2022
p-16, 19.07.2022
p-10, 18.07.2022
pH
7.4
6.0
7.4
6.0
7.4
6.0
pH
7.4
6.0
7.4
6.0
7.4
6.0
pH
7.4
6.0
7.4
6.0
7.4
6.0
Supplementary Figure 4b
180 kDa
180 kDa
180 kDa
Ponceau
Ponceau
Ponceau
170 kDa
130 kDa
95 kDa
72 kDa
55 kDa
43 kDa
34 kDa
26 kDa
170 kDa
130 kDa
95 kDa
72 kDa
55 kDa
43 kDa
34 kDa
26 kDa
170 kDa
130 kDa
95 kDa
72 kDa
55 kDa
43 kDa
34 kDa
26 kDa

## Slide 103
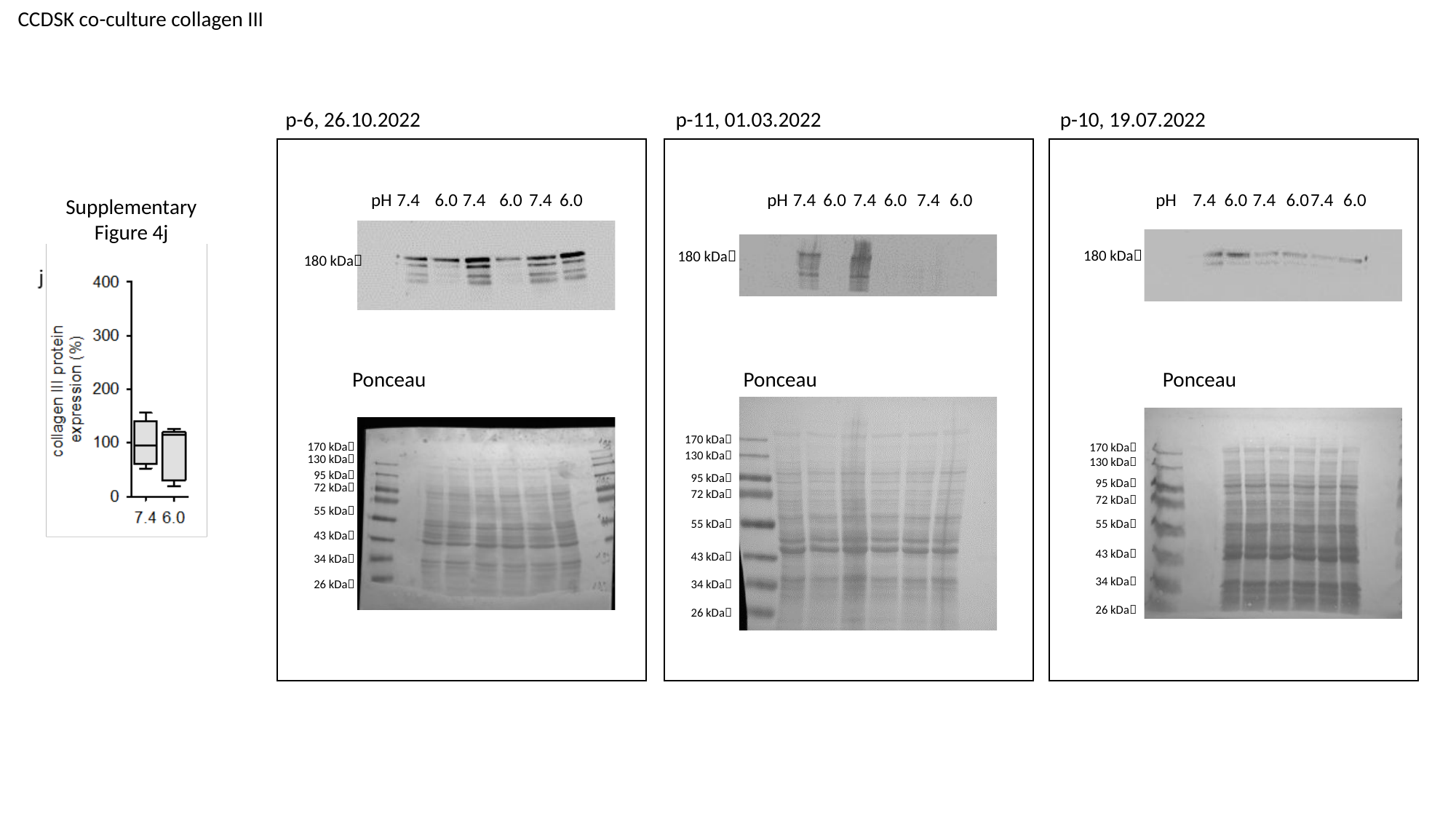

CCDSK co-culture collagen III
p-6, 26.10.2022
p-11, 01.03.2022
p-10, 19.07.2022
pH
7.4
6.0
7.4
6.0
7.4
6.0
pH
7.4
6.0
7.4
6.0
7.4
6.0
pH
7.4
6.0
7.4
6.0
7.4
6.0
Supplementary Figure 4j
180 kDa
180 kDa
180 kDa
Ponceau
Ponceau
Ponceau
170 kDa
130 kDa
95 kDa
72 kDa
55 kDa
43 kDa
34 kDa
26 kDa
170 kDa
130 kDa
95 kDa
72 kDa
55 kDa
43 kDa
34 kDa
26 kDa
170 kDa
130 kDa
95 kDa
72 kDa
55 kDa
43 kDa
34 kDa
26 kDa

## Slide 104
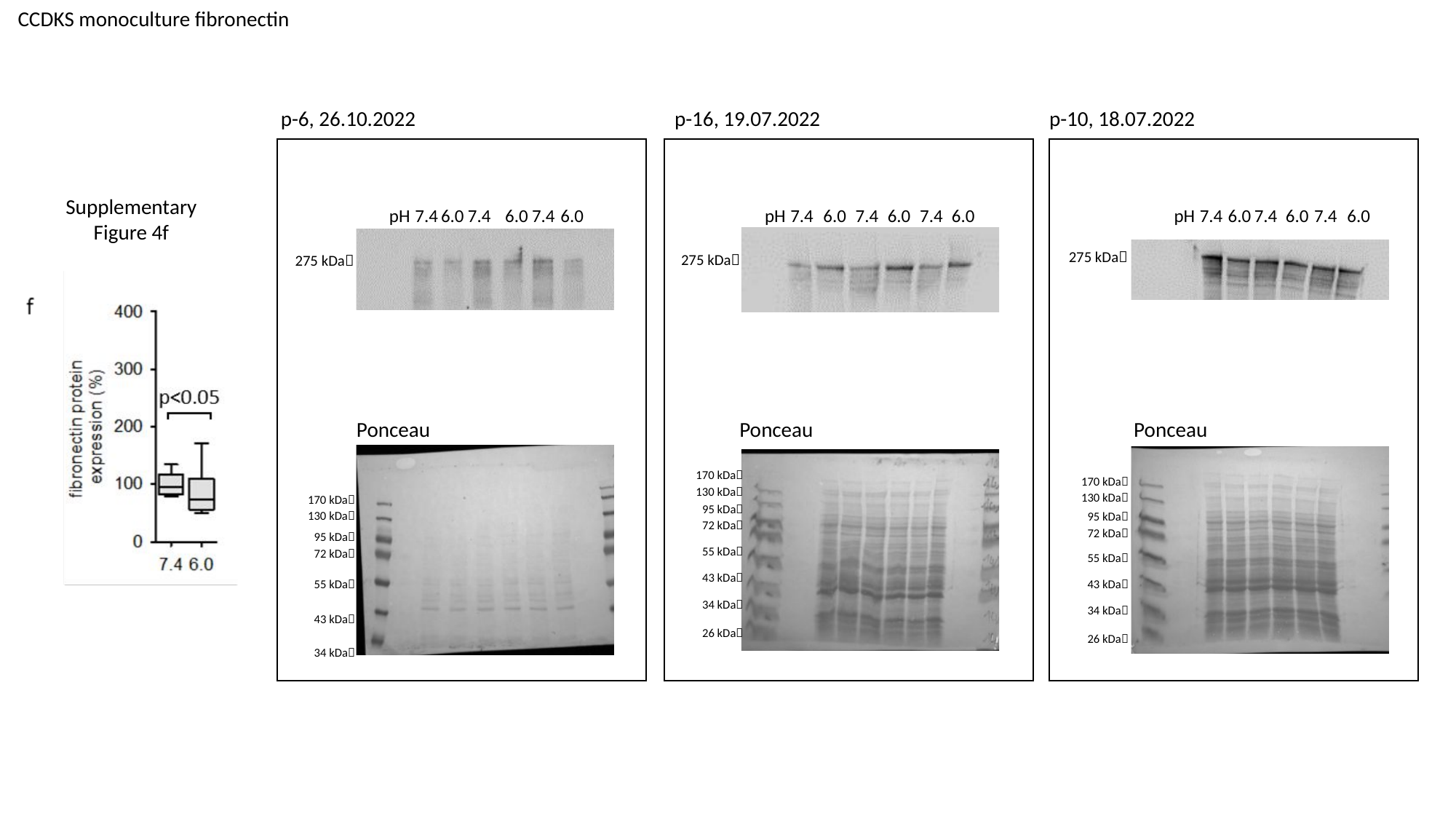

CCDKS monoculture fibronectin
p-6, 26.10.2022
p-16, 19.07.2022
p-10, 18.07.2022
Supplementary Figure 4f
pH
7.4
6.0
7.4
6.0
7.4
6.0
pH
7.4
6.0
7.4
6.0
7.4
6.0
pH
7.4
6.0
7.4
6.0
7.4
6.0
275 kDa
275 kDa
275 kDa
Ponceau
Ponceau
Ponceau
170 kDa
130 kDa
95 kDa
72 kDa
55 kDa
43 kDa
34 kDa
26 kDa
170 kDa
130 kDa
95 kDa
72 kDa
55 kDa
43 kDa
34 kDa
26 kDa
170 kDa
130 kDa
95 kDa
72 kDa
55 kDa
43 kDa
34 kDa

## Slide 105
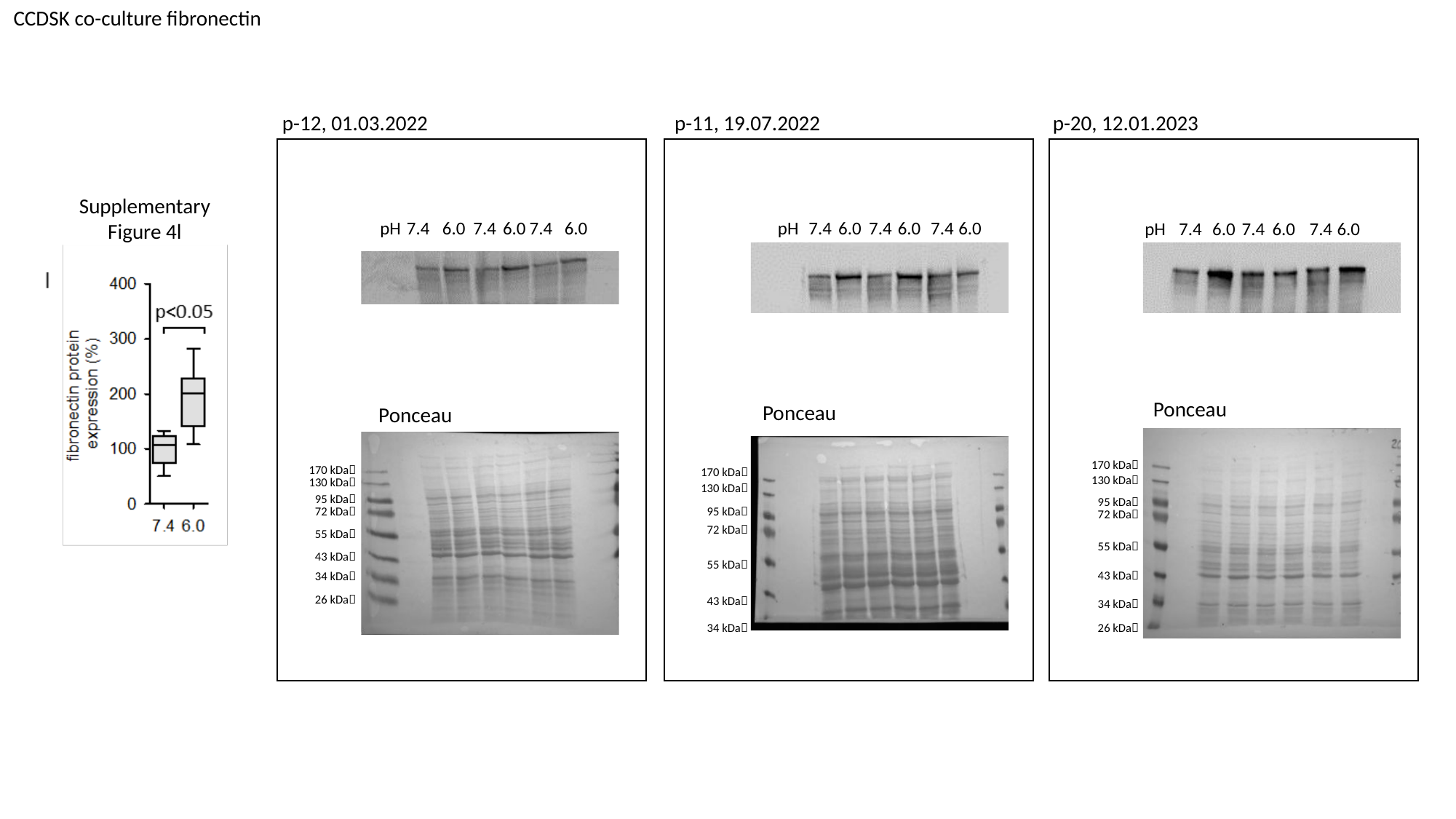

CCDSK co-culture fibronectin
p-12, 01.03.2022
p-11, 19.07.2022
p-20, 12.01.2023
Supplementary Figure 4l
pH
7.4
6.0
7.4
6.0
7.4
6.0
pH
7.4
6.0
7.4
6.0
7.4
6.0
pH
7.4
6.0
7.4
6.0
7.4
6.0
Ponceau
Ponceau
Ponceau
170 kDa
130 kDa
95 kDa
72 kDa
55 kDa
43 kDa
34 kDa
26 kDa
170 kDa
130 kDa
95 kDa
72 kDa
55 kDa
43 kDa
34 kDa
26 kDa
170 kDa
130 kDa
95 kDa
72 kDa
55 kDa
43 kDa
34 kDa
